# Novel Halolactones Derived from Vanillin: Design, Synthesis, Structural Characterization, and Evaluation of Antiproliferative and Hemolytic Activities

**DOI:** 10.3390/molecules30214180

**Published:** 2025-10-25

**Authors:** Anna Dunal, Witold Gładkowski, Ewa Dejnaka, Joanna Sulecka-Zadka, Aleksandra Pawlak, Aleksandra Włoch, Hanna Pruchnik, Gabriela Maciejewska

**Affiliations:** 1 Department of Food Chemistry and Biocatalysis, Wrocław University of Environmental and Life Sciences, Norwida 25, 50-375 Wrocław, Poland; 2 Department of Pharmacology and Toxicology, Faculty of Veterinary Medicine, Wrocław University of Environmental and Life Sciences, Norwida 31, 50-375 Wrocław, Poland; ewa.dejnaka@upwr.edu.pl (E.D.); joanna.sulecka-zadka@upwr.edu.pl (J.S.-Z.); aleksandra.pawlak@upwr.edu.pl (A.P.); 3 Department of Physics and Biophysics, Faculty of Biotechnology and Food Sciences, Wrocław University of Environmental and Life Sciences, Norwida 25, 50-375 Wrocław, Poland; aleksandra.wloch@upwr.edu.pl (A.W.); hanna.pruchnik@upwr.edu.pl (H.P.); 4 Omics Research Center, Wrocław Medical University, 50-368 Wrocław, Poland; gabriela.maciejewska@umw.edu.pl

**Keywords:** vanillin, lactones, aromatic ring, halolactonization, antiproliferative activity, hemolytic activity

## Abstract

A series of novel γ-halo-δ-lactones and δ-halo-γ-lactones bearing a phenolic ring at the β-position were synthesized from vanillin. The divergent seven-step synthetic route commenced with the benzyl protection of the hydroxy group of the starting material, followed by a four-step transformation that led to the corresponding β-aryl-γ,δ-unsaturated carboxylic acids. Subsequent halolactonization (iodo-, bromo-, and chlorolactonization), followed by selective benzyl deprotection, gave the target halolactones. The structures of all intermediates and final lactones were confirmed by comprehensive spectroscopic analyses, including NMR and HRMS. The resulting halolactones were evaluated for antiproliferative activity against two canine (CLBL-1, CLB70) and two human (T-24, CaCo-2) cancer cell lines, as well as non-cancerous mouse embryonic fibroblasts (NIH/3T3). Hemolytic assays were performed to assess toxicity against human red blood cells (RBCs). Among the tested lactones, the *trans*δ-iodo-γ-lactone was the most active, particularly against CLBL-1 and T-24 cells. All compounds demonstrated no inhibitory effects on normal fibroblasts and no hemolytic toxicity. This favorable selectivity profile positions this group of lactones, particularly *trans*-δ-iodo-γ-lactone, as a promising candidate for further development as potential anticancer agents.

## 1. Introduction

Five- and six-membered lactone rings predominate in nature due to their favorable thermodynamic stability [1]. Natural compounds containing lactone rings are produced mainly by plants [2,3,4], but also by insects [5], microorganisms [6], and marine organisms [7,8], where they function as secondary metabolites. This structurally diverse group of compounds exhibits a wide range of biological activities, including antibacterial [9,10,11,12], anti-inflammatory [13,14], antifeedant [15,16,17], and antifungal properties [18,19,20]. Due to their broad spectrum of bioactivities, lactones are applied in economically important sectors such as the food industry, medicine, and agriculture. Their multifunctional nature makes them attractive candidates for the development of bioactive agents and functional materials.

However, particular attention has been paid to their anticancer properties [21,22,23,24]. Numerous studies have been conducted to demonstrate the cytotoxic activity of lactones containing an aromatic substituent against cancer or normal cell lines [25,26,27,28,29,30]. Selected β-aryl-δ-halo-γ-lactones synthesized from simple aromatic aldehydes exhibit cytotoxicity against human (e.g., Jurkat, HL-60, AGS) and canine (e.g., D17, GL-1, CLBL-1) cancer cell lines [31,32,33,34]. The mechanism of action of these compounds involves the induction of apoptosis via a mitochondrial-dependent pathway and caspase activation, as demonstrated for cuminaldehyde-derived δ-iodo-γ-lactones [35]. Enantiomeric *trans*-β-aryl-δ-iodo-γ-lactones derived from 2,5-dimethylbenzaldehyde were shown to trigger cancer cell apoptosis by downregulating the anti-apoptotic proteins Bcl-2 and Bcl-xL. In the canine cell lines used in this study, the tested compounds also activated the receptor-mediated apoptotic pathway through fragmentation of the Bid protein, thereby enhancing their pro-apoptotic effects [36]. To gain deeper insight into their biophysical behavior and anticancer mechanisms, enantiomeric piperonal-derived *trans*-β-aryl-δ-iodo-γ-lactones have been extensively studied for their ability to induce apoptosis and interact with biological systems, including cancer cell membranes and biomacromolecules. Membrane interaction studies revealed a reduction in membrane fluidity, correlating with a marked induction of apoptosis by these lactones [37]. *Cis*-β-aryl-δ-iodo-γ-lactones containing unsubstituted or *p*-alkyl-substituted phenyl ring exhibited cytotoxic activity against HeLa (human cervix carcinoma) and MCF-7 (human breast adenocarcinoma) cancer cell lines, and significantly disrupted the antioxidative/oxidative balance in the NHDF (normal human dermal fibroblasts) cell line [38].

Continuing our studies on the biological activity of lactones bearing an aromatic ring, we designed and synthesized a series of novel β-arylhalolactones using vanillin (**1**) as the starting material. This naturally occurring aromatic aldehyde has a well-documented range of biological properties, including gastro- and cardioprotective, diuretic, anti-organ toxicity, antimicrobial, anti-inflammatory, neuroprotective, and anti-infective effects, among others [39,40,41]. Numerous research groups have also reported significant anticancer activity of vanillin (**1**) against various human cancer cell lines, like MCF-7 (breast cancer) [42], HepG2 (hepatocellular carcinoma) [43], HT-29 (colorectal adenocarcinoma) [44], NCI-H460 (non-small cell lung carcinoma) [45], as well as A2058 (malignant melanoma) cell lines [46].

Due to the presence of various reactive functional groups, vanillin (**1**) is also a versatile building block for the synthesis of novel derivatives, including a wide range of heterocyclic systems such as pyrimidines, quinoxalines, imidazoles, and thiazoles, which are highly relevant in medicinal and pharmaceutical chemistry due to a broad spectrum of pharmacological activities [47]. Vanillin-derived hydrazones exhibit antimicrobial, anticancer, and enzyme inhibition properties, making them promising candidates for therapeutic applications [48]. Additionally, derivatives bearing tacrine or naphthalimido moieties have been developed as multi-target agents for Alzheimer′s disease therapy. These compounds act as antioxidants and inhibit acetylcholinesterase (AChE) as well as β-amyloid peptide aggregation, both of which are key pathological features of Alzheimer′s disease [49]. Other vanillin-based derivatives have been designed as dual inhibitors targeting both AChE and butyrylcholinesterase (BuChE), thereby enhancing their potential efficacy in neurodegenerative conditions [50]. Regarding anticancer activity, certain vanillin derivatives—such as compounds based on the SBE13 scaffold, significantly outperform the parent compound by potently inducing apoptosis and reducing Plk1 kinase activity in HeLa cells, indicating strong antiproliferative effects [51]. This further emphasizes the potential of vanillin as a versatile scaffold for designing targeted anticancer therapeutics.

The presence of a phenolic fragment is an advantageous factor influencing the potential of vanillin-derived halolactones as a promising bioactive compound with different therapeutic properties, including cytotoxic, antioxidant, and anti-inflammatory, which can be of high relevance in oncology. Here, we would like to present the synthesis and studies on the cytotoxicity of new halolactones obtained from vanillin (**1**) against different cancer cell lines. Due to the frequently observed high toxicity of anticancer agents against healthy cells, we also decided to investigate the hemolytic properties of the synthesized halolactones against human red blood cells (RBCs). Owing to their simple structure and crucial physiological functions, these cells are a simple and well-established model to evaluate general hemolytic activity, as erythrocyte membranes are highly sensitive to chemically induced damage and serve as a convenient indicator of membrane-disruptive properties of bioactive compounds.

## 2. Results and Discussion

### 2.1. Synthesis of Vanillin-Derived Halolactones

The presence of a reactive phenolic group in the benzene ring of starting compound dictated our synthetic strategy, which included the benzyl protection of vanillin (**1**), the synthesis of the key intermediate γ,δ-unsaturated carboxylic acid **6**, and subsequent halolactonization, followed by a final deprotection step to obtain the target lactones. The use of benzyl protection was essential to prevent side reactions involving the phenolic OH group during the multistep synthesis.

#### 2.1.1. Synthesis of α,β-Unsaturated Carboxylic Acid **6**

**Scheme 1:**
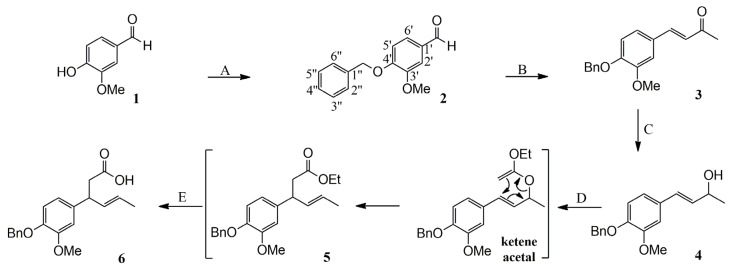
Synthesis of unsaturated γ,δ-carboxylic acid intermediate **6** from vanillin (**1**). *Reagents and conditions:* (A) BnBr, K_2_CO_3_, EtOH, r.t., 48 h (88%) (B) acetone, 10% NaOH, r.t., 24 h (84%) (C) MeOH, NaBH_4_, ice bath 1 h, then r.t., 23 h (82%) (D) triethyl orthoacetate, propionic acid, 138 °C, 6 h (47%) (E) 10% NaOH, EtOH, reflux, 5 h (82%).

The five-step synthesis of γ,δ-unsaturated carboxylic acid **6** is shown in Scheme 1. This synthetic pathway was designed based on the procedure used for the synthesis of β-aryl-δ-halo-γ-lactones and their δ-lactone isomers from other aromatic aldehydes, namely benzaldehyde, *p*-methylbenzaldehyde and cuminaldehyde [32].

Benzylation of vanillin (**1**) was carried out using an ethanolic solution of benzyl bromide in the presence of potassium carbonate. Benzylvanillin **2** [52] was obtained in an 88% yield after crystallization from ethanol.

In the next step, benzylvanillin **2** was converted to known [53] α,β-unsaturated ketone **3** in high yield (84%) via Claisen–Schmidt condensation with acetone under alkaline conditions. The coupling constant between the olefinic protons observed in the ^1^H NMR spectrum (*J* = 16.2 Hz) confirmed the *E*-configuration of the C3–C4 double bond.

Reduction of ketone **3** by sodium borohydride afforded exclusively the corresponding allylic alcohol **4** and no traces of saturated alcohol was detected by TLC and GC analysis directly after separation of the product from the reaction mixture. Spectroscopic data confirmed the selective reduction of the carbonyl group, indicated by a multiplet at 4.47 ppm from H-2 and a singlet corresponding to the hydroxyl group at 1.64 ppm, as well as a strong band at 3384 cm^−1^ attributed to O–H stretching vibrations. The retention of the *E*-configured double bond was confirmed by the coupling constant between the H-3 and H-4 protons (*J* = 15.8 Hz).

Alcohol **4** was subjected to the Johnson-Claisen rearrangement using triethyl orthoacetate in the presence of a catalytic amount of propionic acid at 138 °C. The mechanism involves initial etherification of the allylic alcohol with triethyl orthoacetate, followed by elimination of ethanol to form a ketene acetal intermediate (Scheme 1). Upon heating, this intermediate undergoes a [3.3] sigmatropic rearrangement to yield the γ,δ-unsaturated ester **5**. Consistently with the mechanism of Johnson-Claisen rearrangement, *E* configuration of double bond in ester **5** was retained, as indicated by the coupling constant (*J* = 15.3 Hz) between olefinic protons H-4 and H-5. Ester **5**, after two-step purification (by gravity chromatography followed by flash chromatography) was hydrolyzed in a 10% ethanolic NaOH solution under reflux to afford the γ,δ-unsaturated carboxylic acid **6**. Besides benzylvanillin (**2**) and ketone **3**, other compounds (**4**,**5** and **6**) depicted in Scheme 1 were not previously published and their spectroscopic data are presented in Section 3.5, Section 3.6 and Section 3.7.

#### 2.1.2. Halolactonization of γ,δ-Unsaturated Carboxylic Acid **6**

**Scheme 2:**
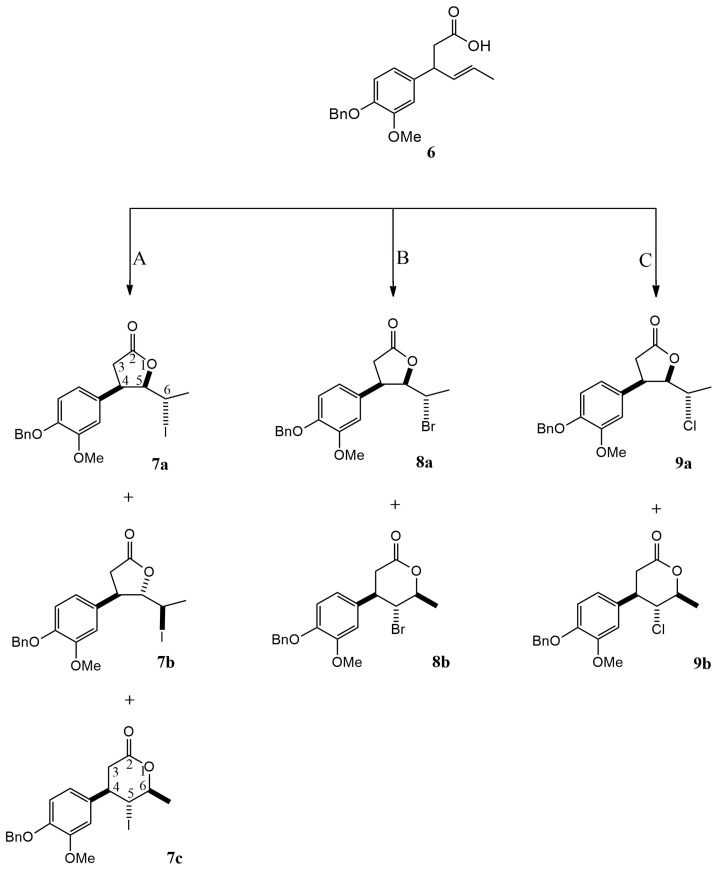
Halolactonization of acid **6** (only isolated products are shown). *Reagents and conditions:* (A) I_2_/KI, Et_2_O, NaHCO_3_, r.t., 20 h (B) NBS, acetic acid, THF, r.t., 24 h (C) NCS, acetic acid, THF, r.t., 24 h.

Acid **6** was subjected to the halolactonization, leading to the formation of novel iodo-, bromo-, and chlorolactones as illustrated in Scheme 2.

The iodolactonization reaction was carried out using iodine, potassium iodide, in Et_2_O/NaHCO_3_ mixture. After the reaction, three products were successfully isolated and purified by flash chromatography.

The major isolated products were two diastereomeric γ-lactones, **7a** and **7b**, formed via 5-exo cyclization. Analysis of their ^1^H NMR spectra revealed significant differences in the chemical shifts and multiplicities of the H-4, H-5, and H-6 protons. Comparison with the data reported by Gładkowski et al. [32] enabled the assignment of the relative configurations at C-4 and C-5 in the γ-lactone ring of both compounds. In the cited work, the relative stereochemistry of analogous lactones bearing an unsubstituted phenyl ring was unambiguously determined by X-ray crystallography, and their NMR data closely matched those observed here.

**Scheme 3:**
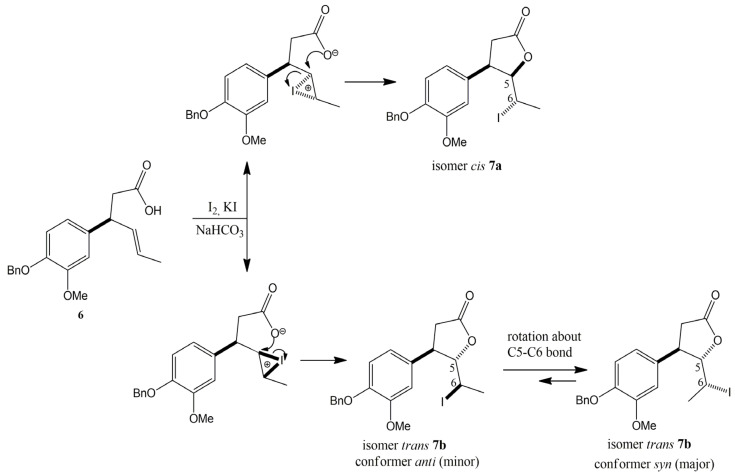
Iodolactonization of acid **6** and formation of *cis*- and *trans*-γ-lactones (**7a** and **7b**).

For example, in the case of the *trans*-δ-iodo-γ-lactone **7b**, a characteristic triplet at 4.23 ppm (*J* = 5.3 Hz) assigned to H-5 was observed, in contrast to the doublet of doublets at 4.78 ppm found in the *cis* isomer **7a**. The H-6 proton signal in the *cis* isomer **7a** appeared significantly upfield (3.47 ppm) compared to that in the *trans* isomer **7b** (4.36 ppm), attributable to the positioning of H-6 within the shielding cone of the aromatic ring. The coupling constant between H-6 and H-5 (*J* = 10.9 Hz) observed in **7a** indicates an antiperiplanar orientation of the C5–H5 and C6–H6 bonds, which is consistent with the previously reported crystal structure of an analogous *cis*-β-phenyl-δ-iodo-γ-lactone exhibiting a torsion angle of −179.14° [32]. This stereochemical outcome is a consequence of the iodolactonization mechanism, wherein the carboxylate ion attacks the C-5 carbon from the face opposite to the three-membered iodonium ion, resulting in both *cis* and *trans* isomers (Scheme 3).

For the *trans*-δ-iodo-γ-lactone **7b**, additional minor signals in the ^1^H and ^13^C NMR spectra reflect a conformational equilibrium between two conformers: a predominant *syn* conformer, in which the C–I and C–O bonds adopt a gauche conformation, and a minor *anti* conformer, with these bonds in an antiperiplanar arrangement. The *syn* conformer forms through rotation about the C5–C6 bond to minimize steric repulsion between the phenyl ring and iodine. Similar observations were reported by Gładkowski et al. for analogous *trans*-δ-iodo-γ-lactone bearing an unsubstituted phenyl ring [32]. Additionally, the coupling constant between H-5 and H-6 in isomer **7b** *(J* = 5.3 Hz) was significantly lower than that observed for the *cis* isomer **7a**, reflecting the differing torsion angles between these protons. This interpretation was also fully supported by X-ray crystallographic analysis of the analogous *trans* lactone [32].

In the case of the minor isolated product of 5-*endo* iodolactonization, γ-iodo-δ-lactone **7c**, the most informative signal was the triplet from the H-5 proton at 4.00 ppm with the coupling constant *J* = 10.6 Hz. This value unequivocally indicated the pseudoaxial orientations of H-4, H-5 and H-6 protons and a *trans* orientation of the iodine at C-5 relative to both the benzene ring at C-4 and the methyl group at C-6.

Bromo- and chlorolactonization of acid **6** were carried out using *N*-bromosuccinimide (NBS) and *N*-chlorosuccinimide (NCS), respectively, in anhydrous tetrahydrofuran with a catalytic amount of acetic acid. Both *cis*-δ-bromo-γ-lactone **8a** and *cis*-δ-chloro-γ-lactone **9a**, as well as their corresponding six-membered analogues **8b**, **9b**, were isolated from the product mixtures. However, attempts to isolate the pure stereoisomers of the *trans*-γ-lactones were unsuccessful.

^1^H NMR data of isolated bromo- and chlorolactones showed a high degree of resemblance in the shapes and coupling constant values of the signals from protons H-4, H-5, and H-6 compared to the corresponding signals observed in the spectra of the analogous iodolactones **7a** and **7c**. Among the halogens, the highest deshielding effect for protons H-5 and H-6 was observed for iodine, followed by bromine and chlorine, which is consistent with research data for analogous β-aryl-δ-halo-γ-lactones and β-aryl-γ-halo-δ-lactones [32].

#### 2.1.3. Benzyl Deprotection of Halolactones **7a**–**c**, **8a**,**b**, **9a**,**b**

The final stage of the synthesis involved the removal of the benzyl protecting group. To identify a selective and efficient method, various deprotection strategies were tested using *cis*-δ-iodo-γ-lactone **7a** as a model substrate, including catalytic hydrogenolysis over Pd/C [54], reaction with 33% HBr in acetic acid [55], treatment with concentrated HCl under reflux [56] or the use of aqueous NaBrO_3_ and Na_2_S_2_O_3_ solutions [57]. Unfortunately, these procedures led to the inseparable mixture of products or isomerization of starting *cis* iodolactone **7a** to the *trans* stereoisomer. Ultimately, the only effective deprotection method involved the use of anhydrous FeCl_3_ in dry dichloromethane at room temperature under a nitrogen atmosphere [58]. This approach enabled successful removal of the benzyl group, though the initial isolated yield of the final lactone **10a** after flash chromatography was only 10%, likely due to complexation of the phenolic product with Fe^3+^ ions. To improve the yield, during the work-up procedure the reaction mixture was washed with 1% phosphoric(V) acid, following the protocol described by Giri et al. for FeCl_3_-mediated deprotection of Boc-protected amino acids and peptides [59]. This modification increased the isolated yield of **10a** to 28%, significantly enhancing the efficiency of the deprotection process. 

**Scheme 4:**
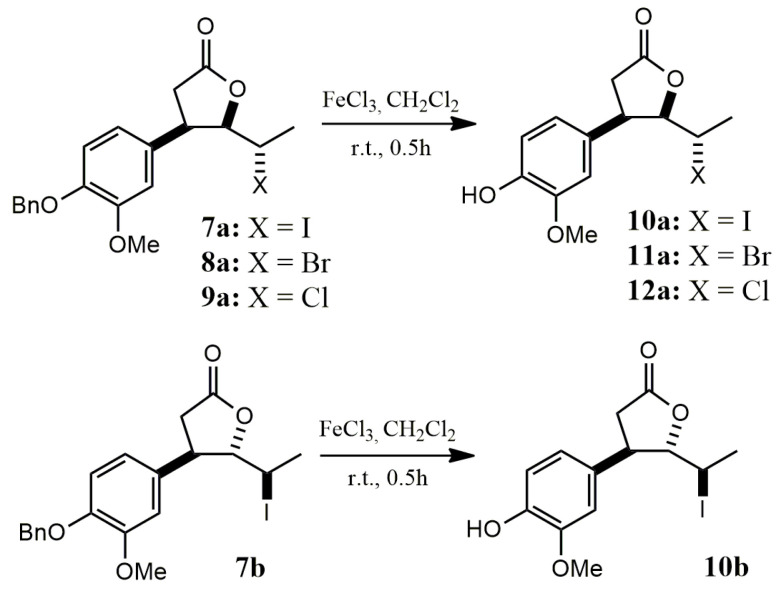
Debenzylation of *cis*-δ-halo-γ-lactones **7a**–**9a** and *trans*-δ-iodo-γ-lactone **7b**.

Following this procedure, successful debenzylation of *cis* δ-halo-γ-lactones **7a**–**9a** and *trans*-δ-iodo-γ-lactone **7b** was carried out, affording the corresponding deprotected lactones **10a**–**12a** and **10b**, respectively (Scheme 4). The selective removal of the benzyl group was confirmed by the disappearance of the aromatic proton signals in the range of 7.31–7.47 ppm and the singlet at 5.14–5.15 ppm corresponding to the benzylic methylene protons in the ^1^H NMR spectra. In place of these, a new singlet appeared at approximately 5.61–5.62 ppm, along with a broad band in the IR spectrum in the range of 3333–3422 cm^−1^, corresponding to O–H stretching vibrations confirming the presence of a free hydroxy group. Moreover, the successful deprotection was additionally evidenced by the expected molecular masses of the target compounds determined by HRMS analysis. Together, these results provide strong evidence for the successful removal of the benzyl protecting group under the optimized reaction conditions.

**Scheme 5:**
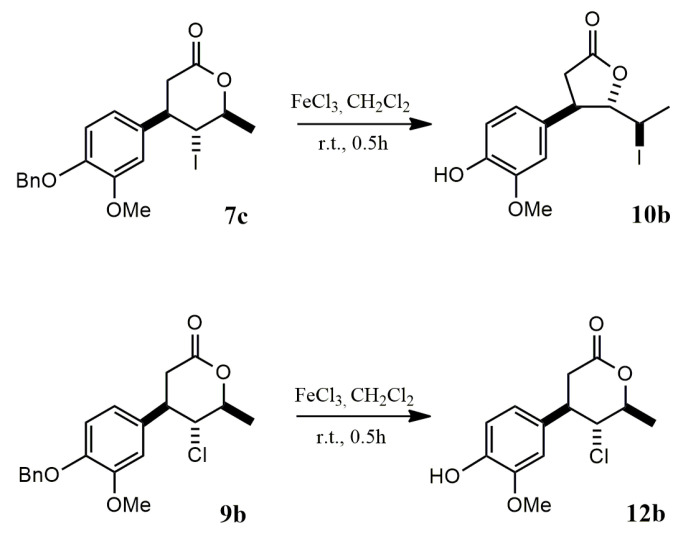
Debenzylation of γ-halo-δ-lactones **7c** and **9b**.

An analogous procedure was applied for the deprotection of γ-halo-δ-lactones **7c**, **8b**, and **9b**. Among these, only in the case of γ-chloro-δ-lactone **9b** a successful deprotection was achieved, and the expected δ-lactone **12b** was obtained as the sole product. Interestingly, NMR analysis of the product obtained from the deprotection of γ-iodo-δ-lactone **7c** clearly confirmed the structure of *trans*-δ-iodo-γ-lactone **10b**, suggesting an intramolecular rearrangement through a translactonization process (Scheme 5). In contrast, for the bromo analogue **8b**, TLC and GC analyses revealed the slow formation of two inseparable products, indicating a less selective reaction course.

**Scheme 6:**
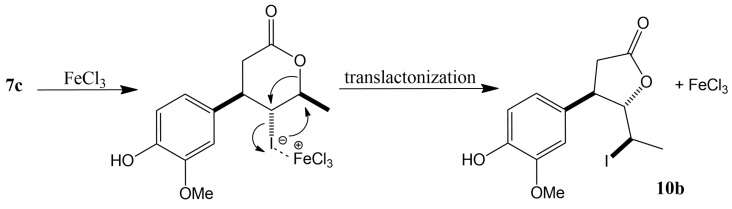
Proposed translactonization mechanism leading to the formation of *trans*-δ-iodo-γ-lactone **10b** during FeCl_3_-mediated debenzylation of γ-iodo-δ-lactone **7c**.

The proposed mechanism of the translactonization process, which occurs during FeCl_3_-mediated debenzylation of γ-iodo-δ-lactone **7c**, is illustrated in Scheme 6.

The rearrangement appears to proceed in a single step via intramolecular nucleophilic substitution, facilitated by FeCl_3_. Acting as a Lewis acid, FeCl_3_ promotes the transposition of the iodine atom from the C-5 to the C-6 position, accompanied by a simultaneous nucleophilic attack of the lactone oxygen on the C-5 carbon, resulting in the formation of a five-membered ring. The exclusive formation of the *trans* isomer strongly suggests that the substitution follows an S_N_2-type mechanism, in which the oxygen nucleophile approaches from the side opposite to the leaving iodine atom.

Translactonization converting δ-lactones into γ-lactones has been previously reported in the literature, both under chemical and microbiological halolactonization conditions. Kamizela et al. [25] observed the unexpected formation of δ-hydroxy-γ-lactones among the products of iodolactonization of 3-methyl-5-arylpent-4-enoic acids using I_2_/KI in a biphasic NaHCO_3_/Et_2_O aqueous system. This phenomenon was attributed to the rearrangement of initially formed γ-iodo-δ-lactones. In that case, the reaction was proposed to proceed via an S_N_1-like mechanism, as evidenced by the formation of both *cis* and *trans* isomers of the δ-hydroxy-γ-lactones. The process involved a dehalogenation-lactonization sequence with two simultaneous nucleophilic substitutions: replacement of the iodine atom at C-5 by the lactone oxygen, and nucleophilic attack of water at C-6 [25]. Interestingly, in one of our previous studies, a similar rearrangement was observed during the biotransformation of a chalcone-derived γ-bromo-δ-lactone in the culture of *Penicillium frequentans* AM 359. In contrast to the chemically induced rearrangement, the microbial process proceeded via an S_N_2 mechanism, which led exclusively to the formation of a single *trans* isomer of the hydroxylactone [60].

The translactonization of γ-halo-δ-lactones reported previously typically involved the participation of water, leading to the formation of δ-hydroxy-γ-lactones. The FeCl_3_-mediated debenzylation of γ-iodo-δ-lactone **7c** reported herein was carried out under strictly anhydrous conditions, favoring an intramolecular S_N_2-type reaction and leading to the selective formation of the *trans*-δ-iodo-γ-lactone. To the best of our knowledge, this type of rearrangement has not been previously reported and may be attributed to the higher thermodynamic stability of five-membered lactone rings compared with six-membered ones. With regard to the relative reactivity of halogens in the presence of FeCl_3_, the order can be classified as I > Br > Cl. This trend corresponds to the bond strength between the carbon and halogen atoms, iodine forms the weakest bond, making γ-iodo-δ-lactones less stable and more susceptible to rearrangement. Consequently, no translactonization was observed for γ-chloro-δ-lactone **9b**, and the process occurred only very slowly for its bromo analogue **8b**. Even after 2 h of reaction, TLC analysis revealed the presence of two distinct products, corresponding to the unrearranged δ-lactone and the newly formed γ-lactone.

### 2.2. Antiproliferative Activity

One of a widely used cytostatic agents in anticancer therapy and in vitro studies is doxorubicin. Sensitivity to doxorubicin varies among cancer cell lines. Previous studies found that after 48 h of incubation, the IC_50_ values were 0.06 μM for the T-24 cell line [61], 0.07 µM for the CLB-70 line [62] and 2.41 µg/mL for the Caco-2 line [63]. For 72 h of incubation, the IC_50_ for CLBL-1 was below 0.09 µM [64]. These results show a heterogeneous response linked to tumor origin, biological traits, and incubation time. Despite its effectiveness, use of doxorubicin is limited by systemic toxicity, especially to healthy cells. Therefore, it is crucial to develop new compounds that are active but non-toxic to healthy cells. 

To evaluate their antiproliferative potential, synthesized vanillin-derived halolactones **10a**,**b**, **11a** and **12a**,**b** as well as starting vanillin (**1**) were subjected to in vitro MTT assay against a panel of selected cancer cell lines: two canine hematopoietic cancer cell lines: canine B-cell lymphoma (CLBL-1) and chronic B-cell leukemia (CLB70), and two human cancer cell lines: bladder carcinoma (T-24) and colorectal adenocarcinoma (CaCo-2). The normal cell line of mouse embryonic fibroblasts (NIH/3T3) was also used in these studies. The results, expressed as IC_50_ values are shown in Table 1. In order to determine the preferential cytotoxicity of the tested compounds against cancer cell lines over normal cell line, the Selectivity Index (SI) was also calculated.

All tested compounds exhibited measurable antiproliferative activity against the canine cancer cell lines, whereas only three compounds showed notable effects against the human cancer cell lines (T-24 or CaCo-2). This observation suggests a higher susceptibility of hematopoietic canine cancer lines compared to the relatively higher resistance displayed by the human cancer cells against the tested compounds.

The highest antiproliferative activity was observed for *trans*-δ-iodo-γ-lactone **10b** against the CLBL-1 cell line, with an IC_50_ value of 46.3 µM, and for vanillin (**1**) against the T-24 cell line (IC_50_ = 48.5 µM). Moderate activity was also noted for *cis*-δ-bromo-γ-lactone **11a** against CLBL-1 (IC_50_ = 63.2 μM) and for compound **10b** against T-24 (IC_50_ = 63.4 μM). In all other cases, the tested compounds exhibited lower activity, with IC_50_ values exceeding 70 µM. Importantly, none of the compounds showed cytotoxic effects against the normal NIH/3T3 fibroblast cell line, indicating a degree of selectivity against cancer cells. To quantify this selectivity, the Selectivity Index [65] as calculated for tested compounds. As shown in Table 1, all compounds exhibited SI values higher than 1, confirming their preferential cytotoxicity toward cancer cells over normal fibroblasts. Notably, the highest SI for the CLBL-1 line was calculated for compound **10b**, supporting its potential as a selective anticancer agent.

Comparing the antiproliferative activity of vanillin (**1**) with its halolactone derivatives against the CLBL-1 cell line, the *trans*-δ-iodo-γ-lactone **10b** and *cis*-δ-bromo-γ-lactone **11a** exhibited notably higher activity. In assays with CaCo-2 cells, vanillin (**1**) was inactive, whereas *cis*-δ-bromo-γ-lactone **11a**, *cis*-δ-chloro-γ-lactone**12a**, and γ-chloro-δ-lactone **12b** demonstrated moderate activity. Conversely, vanillin (**1**) proved to be the most active compound against the T-24 cell line. For the CLB70 line, the antiproliferative activities of vanillin (**1**) and all tested vanillin-derived lactones were comparable.

When evaluating the impact of the halogen substituent on the biological activity of the lactones, iodolactones **10a** and **10b** demonstrated activity against the T-24 bladder carcinoma line and their bromo- and chlorolactone counterparts were inactive (**11a**, **12a** and **12b**). Conversely, for the CaCo-2 colorectal adenocarcinoma line, the trend was reversed: bromo- and chlorolactones inhibited the proliferation unlike iodolactones. Comparing the antiproliferative activity of *cis* isomers of δ-halo-γ-lactones against the CLBL-1 cell line, the highest activity was observed for bromolactone **11a**. In contrast, the activity of all tested *cis*-δ-halo-γ-lactones (**10a**, **11a**, and **12a**) against the CLB70 cell line was comparable. These results clearly indicate that the influence of the halogen substituent on the antiproliferative activity of lactones is strongly dependent on the specific cancer cell line. Similar observations were reported in our previous studies. For instance, when studying a series of racemic halolactones bearing phenyl, *p*-methylphenyl, and *p*-isopropylphenyl substituents at the β-position of the lactone ring, bromolactones exhibited higher antiproliferative activity compared with their iodo- and chloro- analogues against the Jurkat cell line (human T-cell leukemia) [32]. In another study, β-aryl-δ-iodo-γ-lactones containing a 2′,5′-dimethylphenyl substituent showed higher activity against the CLBL-1 and D17 (canine osteosarcoma) cell lines, but lower activity against GL-1 (B-cell leukemia) and Jurkat cell lines when compared with their bromo analogs [34].

Considering the effect of the spatial structure of iodolactones on their antiproliferative properties, it is evident that *trans*-δ-iodo-γ-lactone **10b** exhibited considerably higher activity than its *cis* isomer **10a** against the CLBL-1 and T-24 cell lines, whereas the activities of both isomers against the CLB-70 and CaCo-2 lines were comparable. A higher anti-proliferative activity of *trans* isomers of β-aryl-δ-iodo-γ-lactones, bearing 2,5-dimethylphenyl and 1,3-benzodioxole substituents, compared with their *cis* isomers was also reported against Jurkat, GL-1, CLBL-1, and D17 cell lines [33].

In summary, among all tested halolactones, the most promising antiproliferative properties were exhibited by *trans*-δ-iodo-γ-lactone **10b**. It showed the highest activity against the CLBL-1 and T-24 cell lines, as well as activity against the CLB-70 cell line comparable to that of the other tested halolactones.

In our previous investigation we synthesized and studied antiproliferative activity of a series of halolactones containing an aromatic ring at the β-position derived from simple aromatic aldehydes. Iodolactones derived from 2,5-dimethylbenzaldehyde, cuminaldehyde, and piperonal were particularly active against hematopoietic cancer lines: Jurkat, D17, GL-1, and CLBL-1 [33]. The stereoisomers of cuminaldehyde-derived iodolactones presented higher activity against canine lymphoma/leukemia than against mammary tumor cell lines [35]. Bromolactones with 2,5-dimethylphenyl ring exhibited activity also against CLB70 line [34]. The antiproliferative activity of vanillin-derived compound **10b** reported here against the CLBL-1 cell line (IC_50_ = 46.3 μM) was found to be higher or comparable to some of previously studied analogs. Similar activity was observed for the *cis*-(4*R*,5*R*,6*S*) and *trans*-(4*R*,5*S*,6*R*) cuminaldehyde-derived δ-iodo-γ-lactones, with IC_50_ values ranging from 44 to 45 µM [35]. Notably, lactone **10b** demonstrated higher activity than the piperone-derived enantiomer *cis*-(4*R*,5*R*,6*S*) of δ-iodo-γ-lactone with an IC_50_ value of 68.6 µM [35]. Comparison of compound **10b** with bromolactones bearing a 2,5-dimethylphenyl substituent at the β-position revealed markedly superior activity for **10b**. Among the six bromolactones tested, IC_50_ values ranged from 48 to 155 µM [33], indicating that compound **10b** exhibits a more favorable antiproliferative profile against the CLBL-1 cell line. In assays against the CLB70 cell line, *trans*-δ-iodo-γ-lactone **10b** showed higher activity than the enantiomer (4*R*,5*R*,6*S*) of the γ-bromo-δ-lactone derived from 2,5-dimethylbenzaldehyde, which showed an IC_50_ of 105.7 µM.

It is also worth mentioning that structurally related compounds, such as δ-aryl-γ-halo-δ-lactones synthesized from aryl bromides and 3-methylcrotonaldehyde [26], or *trans*-crotonaldehyde [25], exhibited cytotoxic effects not only against some cancer cell lines but also against the L929 mouse fibroblast normal cell line. In contrast, the vanillin-derived halolactones reported here showed the antiproliferative activity against cancer cells without affecting the normal NIH/3T3 fibroblast cell line. This favorable selectivity profile makes this group of lactones, particularly lactone **10b**, a promising candidate for further development as a safe anticancer agent.

### 2.3. Cytotoxicity Against Red Blood Cells (RBCs)

The next stage of this study focused on determining the toxicity of the tested lactones and vanillin (**1**) against human red blood cells (RBCs) across a wide concentration range (0 to 200 μM) and at different incubation times (2 h, 24 h and 72 h).

**Figure 1:**
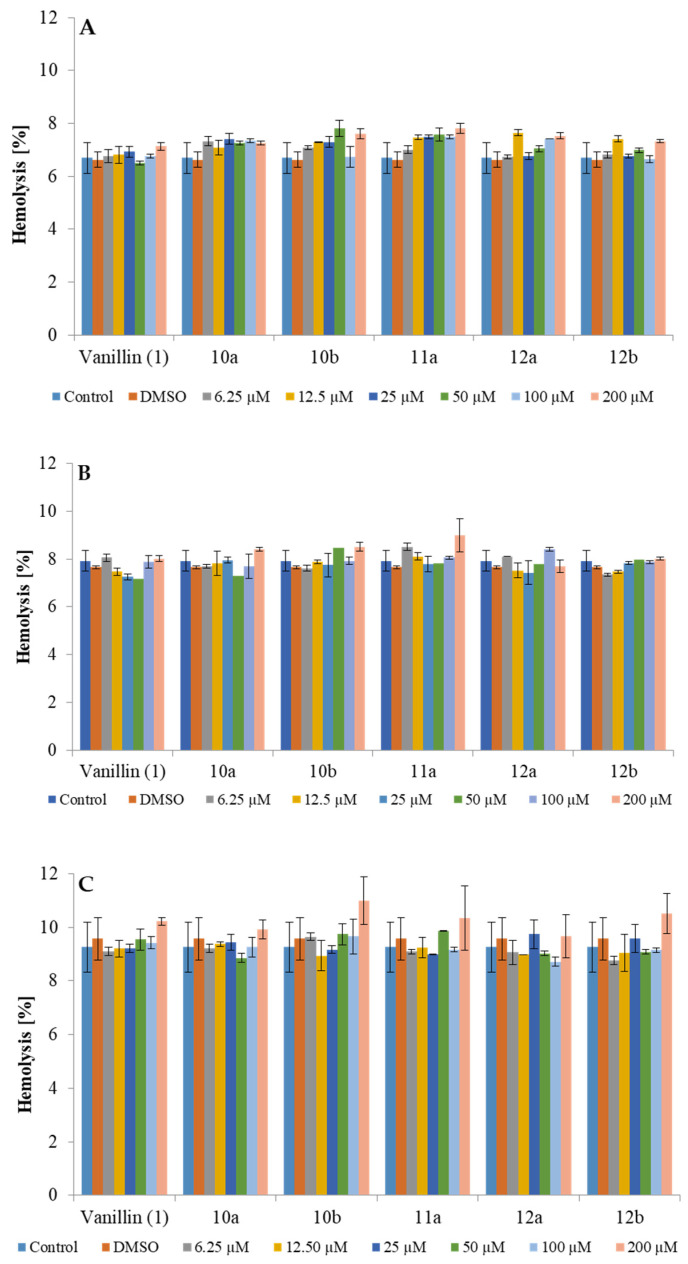
Percentage of hemolysis after 2 h (**A**), 24 h (**B**), 72 (**C**) in the presence of the compounds. Error bars represent mean ± SD (n = 3).

The results of these analyses are presented as the percentage of hemolysis relative to the control sample without the addition of the tested compound and the sample containing only the solvent—DMSO, allowing a precise assessment of the compounds′ impact. Detailed results are shown in Figure 1A–C.

The results showed that incubation of red blood cells with vanillin (**1**) and the tested lactones **10a**,**b**, **11a**, **12a**,**b** for 2 h showed hemolysis between 6.7% and 7.8%, regardless of the concentration, with hemolysis levels for DMSO and control sample 6.7% and 6.6%, respectively (Figure 1A).

A slight increase in the percentage of hemolysis was observed after 24 h of incubation, with values ranging from 7.3% to 9.0%, especially for **10a** and **11a** at the highest concentrations. However, these hemolysis levels were comparable to those of the control and DMSO groups, which demonstrated values of 7.7% and 7.9%, respectively (Figure 1B).

After 72 h of incubation, hemolysis increased slightly in all samples to above 9%. At the highest concentration, three lactones—**10b**, **11a**, and **12b** induced slightly higher hemolysis than the other compounds and the control sample, with values of 11.0%, 10.3%, and 10.5%, respectively. The results obtained indicate a slight hemolytic effect associated with the prolonged incubation and very high concentration (200 μM) of the tested compounds. Nevertheless, the percentage of hemolysis increased by less than 2% compared to the control and DMSO (Figure 1C).

The results of this study demonstrate that over a wide range of concentrations (exceeding those used in cell line studies), both vanillin and all tested vanillin-derived halolactones did not exhibit a significant increase in hemolysis compared to the control and DMSO after 2 and 24 h of exposure. Although prolonged exposure to high concentrations caused minor hemolytic effects for some halolactones, the values remained only slightly elevated relative to the control and DMSO, suggesting that the tested compounds should be considered non-toxic to erythrocytes. It should also be emphasized that hemolysis values for both DMSO and the control remained at the same level, confirming that the solvent itself had no impact on erythrocyte integrity.

The hemolytic activity profile of the tested lactones observed in this study is strongly supported by previous investigations of structurally related compounds, which likewise demonstrated no-toxic effects on erythrocyte membranes. Włoch et al. examined racemic β-aryl-δ-iodo-γ-lactones with various aromatic substituents and showed compounds bearing either a methyl group or no substituent on the benzene ring, did not induce hemolysis in erythrocytes even after 48 h of incubation [38]. In another study, Włoch et al. also evaluated the toxicity of enantiomeric piperonal-derived *trans*-β-aryl-δ-iodo-γ-lactones against human red blood cells (RBCs) across a wide range of concentrations and various incubation times [37]. In this case, as well, the results demonstrated a lack of toxicity at the tested concentrations and extended incubation periods. However, it should be noted that in both cited studies the concentration range of tested compounds (up to 100 µM) was significantly lower than in the present work (up to 200 µM). Furthermore, the incubation time in our study was longer (up to 72 h) compared with those previous reports (up to 48 h).

## 3. Materials and Methods

### 3.1. Chemicals

Vanillin (99% purity), benzyl bromide (98% purity), anhydrous methylene chloride (≥99.8% purity), anhydrous iron(III) chloride (98% purity), *N*-bromosuccinimide (NBS) (95% purity), *N*-chlorosuccinimide (NCS) (98% purity), sodium borohydride (≥96% purity), anhydrous tetrahydrofuran (THF) (≥99% purity), triethyl orthoacetate (97% purity), were purchased from Merck (Darmstadt, Germany). Other common reagents and solvents were purchased from Chempur (Piekary Śląskie, Poland or Idalia Radom, Poland).

### 3.2. Analysis and Purification

The reaction progress was monitored by Thin Layer Chromatography (TLC) using 0.2 mm aluminum plates coated with silica gel 60 F254 (Merck, Darmstadt, Germany). Chromatograms were visualized by spraying the plates with a 1% solution of Ce(SO_4_)_2_ and 2% H_3_[P(Mo_3_O_10_)_4_] in 10% H_2_SO_4_, followed by heating the plates at 120–200 °C.

Gas Chromatography (GC) analysis was conducted using an Agilent Technologies 6890N (Santa Clara, CA, USA) instrument equipped with an autosampler, split injection (50:1), and an FID detector, employing a DB5-HT column (Agilent, Santa Clara, CA, USA), polyimide-coated fused silica tubing, 30 m × 0.25 mm × 0.10 μm with hydrogen as the carrier gas. For analysis of lactones **10a**,**b**, **11a**, **12a**,**b**, the following temperature program was used: injector and detector (FID) temperatures 300 °C, the column temperature was programmed from 100 °C to 200 °C at a rate of 20 °C/min, followed by a ramp from 200 °C to 300 °C at 30 °C/min, with a hold at 300 °C for 8 min. For intermediate product analysis **1**–**5**, the initial column temperature was 100 °C, followed by a temperature increase from 100 °C to 200 °C at a rate of 20 °C/min, then from 200 °C to 300 °C at a rate of 30 °C/min, and finally a 1 min hold at 300 °C.

Nuclear Magnetic Resonance (NMR) spectra, including ^1^H NMR, ^13^C NMR, ^13^C DEPT 135, COSY, HMQC and HMBC were recorded using either a Jeol 400 MHz Year Hold Magnet spectrometer (Jeol Ltd., Tokyo, Japan) or NMR Avance III HD 600 MHz spectrometer (Bruker Biospin GmbH, Rheinstetten, Germany). They are available in the Supplementary Materials (Figures S1–S102). Samples were dissolved in CDCl_3_ (≥99%) or CD_3_OD (≥99%) and chemical shifts were referenced to the residual solvent signal (δ_H_ = 7.26, δ_C_ = 77.00 or δ_H_ = 3.31, δ_C_ = 49.00).

Infrared (IR) spectra were acquired using a Nicolet iS10 FTIR Spectrometer (Thermo Fisher Scientific™, Waltham, MA, USA), equipped with a monolithic diamond ATR crystal attachment. All recorded IR spectra are available in the Supplementary Materials (Figures S103–S119).

High Resolution Mass Spectra (HRMS) were recorded on either a Bruker Daltonics ESI-Q-TOF maXis impact mass spectrometer (Bruker, Billerica, MA, USA) or a Waters Xevo G2 mass spectrometer (Waters, Milford, MA, USA), with positive or negative electrospray ionization (ESI) techniques. All recorded HRMS spectra are available in the Supplementary Materials (Figures S120–S136).

Flash chromatography was conducted using the puriFlash^®^ SX520 Plus system (Interchim, Montluçon, France), which includes a gradient pump, UV detector, and fraction collector. Samples were dry loaded on a pre-column (puriFlash^®^) and the compounds were separated on puriFlash^®^ SIHP F0012 or F0040 30 µm columns by gradient elution with hexane/ethyl acetate mixtures (flow rate: 26 mL/min; pressure: 15 mbar).

The melting points (uncorrected) were determined on the Boetius apparatus (Nagema, Dresden, Germany).

### 3.3. Preparation of Benzylvanillin (***2***)

Vanillin (**1**) (5.00 g, 33.86 mmol), potassium carbonate (5.26 g, 38.06 mmol), and benzyl bromide (4.90 mL, 41.27 mmol) were dissolved in ethanol (150 mL) and stirred at room temperature for 48 h. The reaction mixture was then filtered to remove solid impurities, and the solvent was evaporated under reduced pressure. The resulting residue was treated with 30 mL of 5% aqueous NaOH and the product was extracted with methylene chloride (3 × 30 mL). The combined organic layers were dried over anhydrous MgSO_4_, filtered, and the solvent was removed under reduced pressure. The crude product was dissolved in hot ethanol and recrystallized by cooling at –20 °C for 72 h. The resulting crystals were collected by vacuum filtration using a Büchner funnel, washed with cold ethanol, and dried to afford pure benzylvanillin (**2**).

Yield 7.01 g (88%); light yellow crystals; mp 58–60 °C (lit. 61–63 °C ([52]); R_f_ = 0.45 (hexane/ethyl acetate 3:1, *v*/*v*). Physical and spectral data (Supplementary Materials, page 74) are consistent with reference [52].

### 3.4. Preparation of Ketone ***3*** via Claisen–Schmidt Condensation

A solution of benzylvanillin (**2**) (7.01 g, 28.96 mmol) in acetone (230 mL) was stirred in a water bath at 10 °C. A 10% aqueous NaOH solution (20 mL) was added dropwise to the reaction mixture. The reaction was stirred at room temperature for 24 h, after which the mixture was acidified to pH = 2 using 1 M HCl. The resulting product was extracted with methylene chloride (3 × 40 mL). The combined organic layers were washed with brine, dried over anhydrous MgSO_4_, and filtered. The solvent was removed under reduced pressure, and the crude product was purified by flash chromatography (gradient elution from hexane to hexane/ethyl acetate 7:1, *v*/*v*) to afford pure (*E*)-4-(4′-(benzyloxy-3′-methoxyphenyl)but-3-en-2-one (**3**).

Yield 6.86 g (84%); yellow crystals; mp 91–93 °C (lit. 92–93 °C ([53]); R_f_ = 0.21 (hexane/ethyl acetate 4:1, *v*/*v*). Spectral data (Supplementary Materials, page 74) were reported in accordance with those of a reference [53].

### 3.5. Preparation of Allylic Alcohol ***4***

Ketone **3** (6.86 g, 24.32 mmol) was dissolved in methanol (200 mL), and the solution was cooled in an ice bath. An aqueous solution of NaBH_4_ (2.05 g in 12 mL H_2_O) was added dropwise to the stirred reaction mixture. The reaction was stirred for 1 h in the ice bath and then for 23 h at room temperature. After this time, hot water (150 mL) was added, and the product was extracted with methylene chloride (3 × 30 mL). The organic layer was washed with brine, dried over anhydrous MgSO_4_, and filtered. The solvent was removed under reduced pressure to afford pure (*E*)-4-(4′-benzyloxy-3′-methoxyphenyl)but-3-en-2-ol (**4**), which was characterized by the following physical and spectral data:

Yield 5.67 g (82%); white solid; mp 55–57 °C; R_f_ = 0.25 (hexane/acetone 3:1, *v*/*v*); ^1^H NMR (400 MHz, CDCl_3_): δ 1.37 (d, *J* = 6.4 Hz, 3H, CH_3_-1), 1.64 (s, 1H, -OH), 3.91 (s, 3H, -OCH_3_), 4.47 (m, 1H, H-2), 5.16 (s, 2H, -OCH_2_Ph), 6.13 (dd, *J* = 15.8 Hz and 6.6 Hz, 1H, H-3), 6.48 (d, *J* = 15.8 Hz, 1H, H-4), 6.82 (d, *J* = 8.2 Hz, 1H, H-5′), 6.85 (dd, *J* = 8.2 and 1.6 Hz, 1H, H-6′), 6.96 (d, *J* = 1.6 Hz, 1H, H-2′), 7.30 (m, 1H, H-4″), 7.33–7.39 (m, 2H, H-3″, H-5″), 7.40–7.46 (m, 2H, H-2″, H-6″); ^13^C NMR (100 MHz, CDCl_3_): δ 23.44 (C-1), 55.91 (OCH_3_), 69.02 (C-2), 70.92 (-OCH_2_Ph), 109.25 (C-2′), 113.80 (C-5′), 119.52 (C-6′), 127.19 (C-2″, C-6″), 127.81 (C-4″), 128.52 (C-3″, C-5″), 129.19 (C-4), 130.15 (C-1′), 131.75 (C-3), 136.99 (C-1″), 147.91 (C-4′), 149.62 (C-3′); IR (ATR): ν_max_ = 3384, 1513, 1264, 1139, 969, 798, 858, 744, 699 cm^−1^; HRMS (ESI): *m*/*z* calcd for C_18_H_20_O_3_ [M+Na]^+^: 307.1305; found: 307.1317.

### 3.6. Preparation of Ester ***5*** by Johnson-Claisen Rearrangement

A mixture of alcohol **4** (5.67 g, 19.94 mmol), triethyl orthoacetate (230 mL, 1.27 mol), and a few drops of propionic acid was heated at 138 °C for 6 h with continuous distillation of ethanol. Upon completion of the reaction (monitored by TLC), the mixture was subjected to silica gel column chromatography to remove excess triethyl orthoacetate (eluent: hexane/acetone 10:1, *v*/*v*). The crude product was further purified by flash chromatography (gradient elution from hexane to hexane/ethyl acetate 19:1, *v*/*v*) to afford pure (*E*)-3-(4′-benzyloxy-3′-methoxyphenyl)hex-4-enoate (**5**), which was characterized by the following physical and spectral data:

Yield 3.32 (47%); light yellow oily liquid; R_f_ = 0.64 (hexane/ethyl acetate (3:1, *v*/*v*); ^1^H NMR (400 MHz, CDCl_3_): δ 1.17 (t, *J* = 7.1 Hz, 3H, -OCH_2_CH_3_), 1.65 (d, *J* = 6.0 Hz, 3H, CH_3_-6), 2.62 (dd, *J* = 14.8 and 7.5 Hz, 1H, one of CH_2_-2), 2.66 (dd, *J* = 14.8 and 8.0 Hz, 1H, one of CH_2_-2), 3.73 (m, 1H, H-3), 3.88 (s, 3H, -OCH_3_), 4.04–4.10 (two q, *J* = 7.1 Hz, 2H, -OCH_2_CH_3_), 5.12 (s, 2H, -OCH_2_Ph), 5.48 (dq, *J* = 15.3 and 6.0 Hz, 1H, H-5), 5.57 (ddq, *J* = 15.3, 7.1 and 1.1 Hz, 1H, H-4), 6.68 (dd, *J* = 8.2 and 2.0 Hz 1H, H-6′), 6.75 (d, *J* = 2.0 Hz, 1H, H-2′), 6.81 (d, *J* = 8.2 Hz, H-5′), 7.29 (m, 1H, H-4″), 7.32–7.39 (m, 2H, H-3″, H-5″), 7.40–7.45 (m, 2H, H-2″, H-6″); ^13^C NMR (100 MHz, CDCl_3_): δ 14.19 (-OCH_2_CH_3_), 17.89 (C-6), 41.18 (C-2), 44.57 (C-3), 55.98 (-OCH_3_), 60.26 (-OCH_2_CH_3_), 71.07 (-OCH_2_Ph), 111.38 (C-2′), 114.06 (C-5′), 119.12 (C-6′), 125.42 (C-5), 127.22 (C-2″, C-6″), 127.73 (C-4″), 128.47 (C-3″, C-5″), 133.17 (C-4), 136.61 (C-1′), 137.32 (C-1″), 146.75 (C-4′), 149.53 (C-3′), 172.03 (C-1); IR (ATR): ν_max_ = 1731, 1511, 1259, 1137, 1025, 967, 852, 802, 734, 696 cm^−1^; HRMS (ESI): *m*/*z* calcd for C_22_H_26_O_4_ [M+Na]^+^: 377.1723; found: 377.1740.

### 3.7. Preparation of Acid ***6***

Ester **5** (3.32 g, 9.37 mmol) was heated under reflux for 5 h in a 10% ethanol solution of NaOH (230 mL) containing a few mL of water. After evaporating the solvent under reduced pressure, the residue was diluted with water, and organic impurities were removed by extraction with diethyl ether (3 × 30 mL). The aqueous layer was acidified with 1M HCl, and the product was extracted with methylene chloride (3 × 30 mL). The combined organic layers were washed with brine, dried over anhydrous MgSO_4_, and the solvent was evaporated in *vacuo* to afford pure (*E*)-3-(4′-benzyloxy-3′-methoxyphenyl)hex-4-enoic acid (**6**) with the following physical and spectral data: 

Yield 2.51 g (82%); white solid; mp 95–98 °C; R_f_ = 0.05 (hexane/ethyl acetate 3:1, *v*/*v*); ^1^H NMR (400 MHz, CDCl_3_): δ 1.66 (d, *J* = 5.8 Hz, 3H, CH_3_-6), 2.68 (dd, *J* = 15.6 and 7.4 Hz, 1H, one of CH_2_-2), 2.72 (dd, *J* = 15.6 and 8.0 Hz, 1H, one of CH_2_-2), 3.72 (m, 1H, H-3), 3.87 (s, 3H, -OCH_3_), 5.12 (s, 2H, -OCH_2_Ph), 5.50 (dq, *J* = 15.4 and 5.8 Hz, 1H, H-5), 5.57 (ddq, *J* = 15.4, 6.8 and 1.2 Hz, 1H, H-4), 6.68 (dd, *J* = 8.2 and 2.0 Hz 1H, H-6′), 6.74 (d, *J* = 2.0 Hz, 1H, H-2′), 6.82 (d, *J* = 8.2 Hz, H-5′), 7.29 (m, 1H, H-4″), 7.33–7.39 (m, 2H, H-3″, H-5″), 7.40–7.45 (m, 2H, H-2″, H-6″); ^13^C NMR (100 MHz, CDCl_3_): δ 17.91 (C-6), 40.67 (C-2), 44.06 (C-3), 55.98 (-OCH_3_), 71.05 (-OCH_2_Ph), 111.36 (C-2′), 114.02 (C-5′), 119.06 (C-6′), 125.68 (C-5), 127.23 (C-2″, C-6″), 127.76 (C-4″), 128.49 (C-3″, C-5″), 132.88 (C-4), 136.25 (C-1′), 137.26 (C-1″), 146.86 (C-4′), 149.54 (C-3′), 177.57 (C-1); IR (ATR): ν_max_ = 2500–3500, 1702, 1518, 1236, 1142, 965, 855, 791, 744, 696 cm^−1^; HRMS (ESI): *m*/*z* calcd for C_20_H_22_O_4_ [M+Na]^+^: 349.1410; found: 349.1413.

### 3.8. Preparation of Iodolactones ***7a**–**c***

A solution of acid **6** (2.69 g, 7.70 mmol) in diethyl ether (80 mL) and saturated NaHCO_3_ solution (80 mL) was stirred for 1 h at room temperature. Then, a solution of I_2_ (12.70 g, 50.01 mmol) and KI (3.97 g, 23.92 mmol) in water (20 mL) was added dropwise to the stirred mixture until a stable brown color was observed. After 19 h, the reaction mixture was diluted with diethyl ether and washed with aqueous Na_2_S_2_O_3_. The aqueous phase was shaken with diethyl ether (3 × 30 mL), and the combined organic layers were washed with saturated NaHCO_3_, brine, then dried over anhydrous MgSO_4_. After evaporation of the solvent under reduced pressure, the mixture of iodolactones was separated by flash chromatography (gradient elution from hexane to hexane/ethyl acetate 5:1, *v*/*v*). Physicochemical and spectral data of the isolated iodolactones **7a–c** are given below:

#### 3.8.1. *Cis*-4-(4′-Benzyloxy-3′-methoxyphenyl)-5-(1-iodoethyl)dihydrofuran-2-One (**7a**)

Yield 1.81 g (52%); white solid; mp 116–118 °C; R_f_ = 0.27 (hexane/ethyl acetate 4:1, *v*/*v*); ^1^H NMR (400 MHz, CDCl_3_): δ 2.01 (d, *J* = 6.8 Hz, 3H, CH_3_-7), 2.70 (d, *J* = 17.5 Hz, 1H, one of CH_2_-3), 3.10 (dd, *J* = 17.5 and 8.4 Hz, 1H, one of CH_2_-3), 3.47 (dq, *J* = 10.9 and 6.8 Hz, 1H, H-6), 3.84 (dd, *J* = 8.4 and 5.0 Hz, 1H, H-4), 3.89 (s, 3H, -OCH_3_), 4.78 (dd, *J* = 10.9 and 5.0 Hz, 1H, H-5), 5.14 (s, 2H, -OCH_2_Ph), 6.71 (dd, *J* = 8.3 and 2.1 Hz, 1H, H-6′), 6.80–6.86 (m, 2H, H-2′, H-5′), 7.31 (m, 1H, H-4″), 7.34–7.40 (m, 2H, H-3″, H-5″), 7.41–7.46 (m, 2H, H-2″, H-6″); ^13^C NMR (100 MHz, CDCl_3_): δ 23.70 (C-6), 25.53 (C-7), 38.83 (C-3), 44.54 (C-4), 56.04 (-OCH_3_), 70.98 (-OCH_2_Ph), 87.87 (C-5), 112.80 (C-2′), 113.87 (C-5′), 120.26 (C-6′), 127.31 (C-2″, C-6″), 127.93 (C-4″), 128.57 (C-3″, C-5″), 130.05 (C-1′), 136.86 (C-1″), 147.81 (C-4′), 149.31 (C-3′), 176.64 (C-2); IR (ATR): ν_max_ = 1789, 1518, 1239, 1148, 1012, 954, 857, 751, 700, 538 cm^−1^; HRMS (ESI): *m*/*z* calcd for C_20_H_21_IO_4_ [M+Na]^+^ 475.0377; found: 475.0390.

#### 3.8.2. *Trans*-4-(4′-Benzyloxy-3′-methoxyphenyl)-5-(1-iodoethyl)dihydrofuran-2-one (**7b**)

Yield 0.87 g (25%); white solid; mp 80–82 °C; R_f_ = 0.16 (hexane/ethyl acetate 4:1, *v*/*v*); ^1^H NMR (400 MHz, CDCl_3_): major signals from *syn* conformer: δ 1.86 (d, *J* = 7.1 Hz, 3H, CH_3_-7), 2.64 (dd, *J* = 18.4 and 6.6 Hz, one of CH_2_-3), 3.12 (dd, *J* = 18.4 and 10.0 Hz, 1H, one of CH_2_-3), 3.56 (ddd, *J* = 10.0, 6.6 and 5.3 Hz, 1H, H-4), 3.89 (s, 3H, -OCH_3_), 4.23 (t, *J* = 5.3 Hz, 1H, H-5), 4.36 (qd, *J* = 7.1 and 5.3 Hz, 1H, H-6), 5.14 (s, 2H, -OCH_2_Ph), 6.73 (dd, *J* = 8.2 and 2.1 Hz, 1H, H-6′), 6.75 (d, *J* = 2.1 Hz, 1H, H-2′), 6.85 (d, *J* = 8.2 Hz, 1H, H-5′), 7.31 (m, 1H, H-4″), 7.34–7.40 (m, 2H, H-3″, H-5″), 7.41–7.45 (m, 2H, H-2″, H-6″); ^13^C NMR (100 MHz, CDCl_3_): δ 23.44 (C-7), 28.45 (C-6), 37.67 (C-3), 45.17 (C-4), 56.14 (-OCH_3_), 71.04 (-OCH_2_Ph), 89.56 (C-5), 110.54 (C-2′), 114.35 (C-5′), 119.06 (C-6′), 127.23 (C-2″, C-6″), 127.93 (C-4″), 128.57 (C-3″, C-5″), 134.31 (C-1′), 136.85 (C-1″), 147.69 (C-4′), 150.15 (C-3′), 174.88 (C-2); minor signals from *anti* conformer: ^1^H NMR: δ 1.97 (d, *J* = 7.1 Hz, 3H, CH_3_-7), 2.76 (dd, *J* = 18.1 and 9.5 Hz, one of CH_2_-3), 3.07 (dd, *J* = 18.1 Hz and 9.5 Hz, 1H, one of CH_2_-3), 3.46 (td, *J* = 9.5 and 7.1 Hz, 1H, H-4), 3.73 (dd, *J* = 7.1 and 2.4 Hz, 1H, H-5), 3.90 (s, 3H, -OCH_3_), 5.30 (s, 2H, -OCH_2_Ph), 6.85 (d, *J* = 8.1 Hz, 1H, H-5′); ^13^C NMR: δ 25.42 (C-7), 29.02 (C-6), 37.04 (C-3), 47.41 (C-4), 89.27 (C-5), 110.70 (C-2′), 119.26 (C-6′), 132.07 (C-1′), 174.80 (C-2); IR (ATR): ν_max_ = 1786, 1517, 1454, 1254, 1142, 1030, 970, 848, 804, 746, 697, 644, 536 cm^−1^; HRMS (ESI): *m*/*z* calcd for C_20_H_21_IO_4_ [M+Na]^+^: 475.0377; found: 475.0378.

#### 3.8.3. 4-*r*-(4′-Benzyloxy-3′-methoxyphenyl)-5-*t*-iodo-6-*c*-methyltetrahydropyran-2-one (**7c**)

Yield 0.38 g (11%); white solid; mp 109–112 °C; R_f_ = 0.15 (hexane/ethyl acetate 4:1, *v*/*v*); ^1^H NMR (400 MHz, CDCl_3_): δ 1.74 (d, *J* = 6.2 Hz, 3H, CH_3_-7), 2.65 (dd, *J* = 17.6 and 10.6 Hz, 1H, one of CH_2_-3), 2.98 (dd, *J* = 17.6 and 6.6 Hz, 1H, one of CH_2_-3), 3.43 (td, *J* = 10.6 and 6.6 Hz, 1H, H-4), 3.90 (s, 3H, -OCH_3_), 4.00 (t, *J* = 10.6 Hz, H-5), 4.75 (dq, *J* = 10.6 and 6.2 Hz, 1H, H-6), 5.15 (s, 2H, -OCH_2_Ph), 6.65 (dd, *J* = 8.0 and 2.2 Hz, 1H, H-6′), 6.67 (d, *J* = 2.2 Hz, 1H, H-2′), 6.87 (d, *J* = 8.0 Hz, 1H, H-5′), 7.31 (m, 1H, H-4″), 7.35–7.40 (m, 2H, H-3″, H-5″), 7.42–7.46 (m, 2H, H-2″, H-6″); ^13^C NMR (100 MHz, CDCl_3_): δ 22.30 (C-7), 34.90 (C-5), 37.72 (C-3), 48.22 (C-4), 56.10 (-OCH_3_), 70.99 (-OCH_2_Ph), 81.00 (C-6), 110.46 (C-2′), 113.99 (C-5′), 118.90 (C-6′), 127.27 (C-2″, C-6″), 127.90 (C-4″), 128.56 (C-3″, C-5″), 135.42 (C-1′), 136.90 (C-1″), 147.79 (C-4′), 149.84 (C-3′), 169.73 (C-2); IR (ATR): ν_max_ = 1714, 1518, 1377, 1261, 1141, 1029, 971, 851, 810, 740, 699, 574 cm^−1^; HRMS (ESI): *m*/*z* calcd for C_20_H_21_IO_4_ [M+Na]^+^: 475.0377; found: 475.0383.

### 3.9. Preparation of Bromolactones ***8a**,**b***

A solution of acid **6** (2.51 g, 7.70 mmol) and *N*-bromosuccinimide (1.89 g, 10.62 mmol) in THF (80 mL), with a few drops of acetic acid, was stirred at room temperature for 24 h. The mixture was then diluted with diethyl ether (40 mL) and washed with saturated NaHCO_3_ solution and brine. The organic layer was dried over anhydrous MgSO_4_. After solvent evaporation under reduced pressure, the product mixture was purified by flash chromatography (gradient elution from hexane to hexane/ethyl acetate 5:1, *v*/*v*). Two bromolactones, **8a** and **8b**, were isolated and characterized by the following data:

#### 3.9.1. *Cis*-4-(4′-Benzyloxy-3′-methoxyphenyl)-5-(1-bromoethyl)dihydrofuran-2-one (**8a**)

Yield 1.12 g (35%); white solid; mp 125–128 °C; R_f_ = 0.39 (hexane/chloroform/methanol 8:2:1, *v*/*v*); ^1^H NMR (400 MHz, CDCl_3_): δ 1.76 (d, *J* = 6.6 Hz, 3H, CH_3_-7), 2.71 (d, *J* = 17.5 Hz, 1H, one of CH_2_-3), 3.09 (dd, *J* = 17.5 and 8.4 Hz, 1H, one of CH_2_-3), 3.45 (dq, *J* = 10.4 and 6.6 Hz, 1H, H-6), 3.81 (dd, *J* = 8.4 and 5.1 Hz, 1H, H-4), 3.87 (s, 3H, -OCH_3_), 4.64 (dd, *J* = 10.4 and 5.1 Hz, 1H, H-5), 5.14 (s, 2H, -OCH_2_Ph), 6.68 (dd, *J* = 8.3 and 2.1 Hz, 1H, H-6′), 6.79 (d, *J* = 2.1 Hz, 1H, H-2′), 6.84 (d, *J* = 8.3 Hz, 1H, H-5′), 7.31 (m, 1H, H-4″), 7.34–7.40 (m, 2H, H-3″, H-5″), 7.41–7.47 (m, 2H, H-2″, H-6″); ^13^C NMR (100 MHz, CDCl_3_): δ 23.26 (C-7), 38.29 (C-3), 43.64 (C-4), 45.54 (C-6), 56.02 (-OCH_3_), 70.97 (-OCH_2_Ph), 86.71 (C-5), 112.58 (C-2′), 113.83 (C-5′), 120.10 (C-6′), 127.30 (C-2″, C-6″), 127.93 (C-4″), 128.57 (C-3″, C-5″), 130.19 (C-1′), 136.87 (C-1″), 147.82 (C-4′), 149.34 (C-3′), 176.42 (C-2); IR (ATR): ν_max_ = 178, 1518, 1254, 1146, 1015, 966, 780, 747, 699 cm^−1^; HRMS (ESI): *m*/*z* calcd for C_20_H_21_BrO_4_ [M+Na]^+^: 427.0515; found: 427.0522.

#### 3.9.2. 4-*r*-(4′-Benzyloxy-3′-methoxyphenyl)-5-*t*-bromo-6-*c*-methyltetrahydropyran-2-one (**8b**)

Yield 0.26 g (8%); white solid; mp 132–134 °C; R_f_ = 0.33 (hexane/chloroform/methanol 8:2:1, *v*/*v*); ^1^H NMR (400 MHz, CDCl_3_): δ 1.65 (d, *J* = 6.2 Hz, 3H, CH_3_-7), 2.70 (dd, *J* = 17.6 and 9.4 Hz, 1H, one of CH_2_-3), 3.05 (dd, *J* = 17.6 and 6.9 Hz, 1H, one of CH_2_-3), 3.40 (td, *J* = 9.4 and 6.9 Hz, 1H, H-4), 3.90 (s, 3H, -OCH_3_), 3.91 (t, *J* = 9.4 Hz, H-5), 4.63 (dq, *J* = 9.4 and 6.2 Hz, 1H, H-6), 5.14 (s, 2H, -OCH_2_Ph), 6.68 (dd, *J* = 8.1 and 2.1 Hz, 1H, H-6′), 6.71 (d, *J* = 2.1 Hz, 1H, H-2′), 6.87 (d, *J* = 8.1 Hz, 1H, H-5′), 7.31 (m, 1H, H-4″), 7.34–7.40 (m, 2H, H-3″, H-5″), 7.41–7.46 (m, 2H, H-2″, H-6″); ^13^C NMR (100 MHz, CDCl_3_): δ 20.62 (C-7), 37.64 (C-3), 46.78 (C-4), 54.51 (C-5), 56.10 (-OCH_3_), 71.00 (-OCH_2_Ph), 79.59 (C-6), 110.66 (C-2′), 114.05 (C-5′), 119.00 (C-6′), 127.25 (C-2″, C-6″), 127.90 (C-4″), 128.57 (C-3″, C-5″), 133.91 (C-1′), 136.93 (C-1″), 147.81 (C-4′), 149.88 (C-3′), 169.45 (C-2); IR (ATR): ν_max_ = 1721, 1517, 1375, 1225, 1141, 1001, 972, 804, 758, 704 cm^−1^; HRMS (ESI): *m*/*z* calcd for C_20_H_21_BrO_4_ [M+Na]^+^: 427.0515; found: 427.0517.

### 3.10. Preparation of Chlorolactones ***9a,b***

A solution of acid **6** (2.51 g, 7.70 mmol) and *N*-chlorosuccinimide (2.32 g, 17.37 mmol) in THF (80 mL), with a few drops of acetic acid, was stirred at room temperature for 24 h. The work-up procedure and isolation described in Section 3.9 were applied to isolate chlorolactones **9a** and **9b**, which were characterized by the following physical and spectral data:

#### 3.10.1. *Cis*-4-(4′-Benzyloxy-3′-methoxyphenyl)-5-(1-chloroethyl)dihydrofuran-2-one (**9a**)

Yield 0.28 g (10%); white solid; mp 81–84 °C; R_f_ = 0.37 (hexane/chloroform/methanol 8:2:1, *v*/*v*); ^1^H NMR (400 MHz, CDCl_3_): δ 1.56 (d, *J* = 6.5 Hz, 3H, CH_3_-7), 2.71 (d, *J* = 17.6 and 0.8 Hz, 1H, one of CH_2_-3), 3.07 (dd, *J* = 17.6 and 8.4 Hz, 1H, one of CH_2_-3), 3.43 (dq, *J* = 10.1 and 6.5 Hz, 1H, H-6), 3.80 (dd, *J* = 8.4 and 5.2 Hz, 1H, H-4), 3.87 (s, 3H, -OCH_3_), 4.49 (dd, *J* = 10.1 and 5.2 Hz, 1H, H-5), 5.14 (s, 2H, -OCH_2_Ph), 6.66 (dd, *J* = 8.3 and 2.2 Hz, 1H, H-6′), 6.76 (d, *J* = 2.2 Hz, 1H, H-2′), 6.84 (d, *J* = 8.3 Hz, 1H, H-5′), 7.31 (m, 1H, H-4″), 7.35–7.40 (m, 2H, H-3″, H-5″), 7.42–7.46 (m, 2H, H-2″, H-6″); ^13^C NMR (100 MHz, CDCl_3_): δ 22.38 (C-7), 37.94 (C-3), 43.13 (C-4), 53.93 (C-6), 56.02 (-OCH_3_), 70.97 (-OCH_2_Ph), 86.58 (C-5), 112.42 (C-2′), 113.82 (C-5′), 120.03 (C-6′), 127.29 (C-2″, C-6″), 127.92 (C-4″), 128.57 (C-3″, C-5″), 130.30 (C-1′), 136.87 (C-1″), 147.81 (C-4′), 149.37 (C-3′), 176.34 (C-2); IR (ATR): ν_max_ = 1789, 1516, 1454, 1238, 1144, 1019, 973, 780, 745, 698, 680 cm^−1^; HRMS (ESI): *m*/*z* calcd for C_20_H_21_O_4_Cl [M+H]^+^: 361.1207; found: 361.1206.

#### 3.10.2. 4-*r*-(4′-Benzyloxy-3′-methoxyphenyl)-5-*t*-chloro-6-*c*-methyltetrahydropyran-2-one (**9b**)

Yield 0.5 g (18%); white solid; mp 93–96 °C; R_f_ = 0.24 (hexane/chloroform/methanol 8:2:1, *v*/*v*); ^1^H NMR (400 MHz, CDCl_3_): δ 1.60 (d, *J* = 6.2 Hz, 3H, CH_3_-7), 2.72 (dd, *J* = 17.7 and 9.5 Hz, 1H, one of CH_2_-3), 3.06 (dd, *J* = 17.7 and 7.0 Hz, 1H, one of CH_2_-3), 3.29 (td, *J* = 9.5 and 7.0 Hz, 1H, H-4), 3.83 (t, *J* = 10.0 Hz, H-5), 3.90 (s, 3H, -OCH_3_), 4.50 (dq, *J* = 10.0 and 6.2 Hz, 1H, H-6), 5.14 (s, 2H, -OCH_2_Ph), 6.70 (dd, *J* = 8.1 and 2.2 Hz, 1H, H-6′), 6.72 (d, *J* = 2.2 Hz, 1H, H-2′), 6.87 (d, *J* = 8.1 Hz, 1H, H-5′), 7.31 (m, 1H, H-4″), 7.35–7.40 (m, 2H, H-3″, H-5″), 7.42–7.46 (m, 2H, H-2″, H-6″); ^13^C NMR (100 MHz, CDCl_3_): δ 19.66 (C-7), 37.21 (C-3), 46.31 (C-4), 56.09 (-OCH_3_), 62.47 (C-5), 71.00 (-OCH_2_Ph), 79.35 (C-6), 110.78 (C-2′), 114.08 (C-5′), 119.10 (C-6′), 127.24 (C-2″, C-6″), 127.90 (C-4″), 128.57 (C-3″, C-5″), 133.13 (C-1′), 136.93 (C-1″), 147.82 (C-4′), 149.89 (C-3′), 169.33 (C-2); IR (ATR): ν_max_ = 1732, 1511, 1258, 1219, 1139, 1016, 973, 796, 738, 696, 642 cm^−1^; HRMS (ESI): *m*/*z* calcd for C_20_H_21_O_4_Cl [M+H]^+^: 361.1207; found: 361.1205.

### 3.11. General Procedure for Benzyl Deprotection of Halolactones ***7a**–**c***, ***8a***,***b*** and ***9a**,**b***

Lactone **7**–**9** (1 equiv) and 10 mL of anhydrous dichloromethane were placed in a two-necked flask equipped with a nitrogen-filled balloon. The solution was stirred on a magnetic stirrer and after complete dissolution of the substrate, anhydrous FeCl_3_ (3 equiv) was added. The mixture was further stirred at room temperature. After complete reaction of the substrate (30 min), the reaction mixture was filtered under reduced pressure through a Schott funnel, transferred to a separatory funnel, and the filtrate was washed with 1% orthophosphoric acid solution (3 × 10 mL). After phase separation, the aqueous layer was further washed with methylene chloride (3 × 15 mL). All combined organic layers were washed with saturated NaCl solution and subsequently dried over anhydrous MgSO_4_. After filtration, the solvent was evaporated under reduced pressure using a rotary evaporator. The concentrated crude product was purified by flash chromatography. Gradient elution from hexane to hexane/ethyl acetate 9:2 (*v*/*v*) was used for lactones **10a**, **10b**, **12a**, and **12b**, while for lactone **11a** a gradient from hexane to hexane/ethyl acetate 3:1 (*v*/*v*) was applied. Reaction yields and the physical and spectral data of the deprotected halolactones are as follows:

#### 3.11.1. *Cis*-4-(4′-Hydroxy-3′-methoxyphenyl)-5-(1-iodoethyl)dihydrofuran-2-one (**10a**)

Obtained from lactone **7a** (1.81 g, 4 mmol) in yield 0.41 g (28%); white solid; mp 56–59 °C; R_f_ = 0.24 (hexane/ethyl acetate 3:1, *v*/*v*), ^1^H NMR (400 MHz, CDCl_3_): δ 2.01 (d, *J* = 6.8 Hz, 3H, CH_3_-7), 2.70 (d, *J* = 17.5 Hz, 1H, one of CH_2_-3), 3.11 (dd, *J* = 17.5 and 8.4 Hz, 1H, one of CH_2_-3), 3.48 (dq, *J* = 10.9 and 6.8 Hz, 1H, H-6), 3.83 (dd, *J* = 8.4 and 5.0 Hz, 1H, H-4), 3.89 (s, 3H, -OCH_3_), 4.78 (dd, *J* = 10.9 and 5.0 Hz, 1H, H-5), 5.62 (s, 1H, -OH), 6.74 (dd, *J* = 8.1 and 2.0 Hz, 1H, H-6′), 6.78 (d, *J* = 2.0 Hz, 1H, H-2′), 6.86 (d, *J* = 8.1 Hz, 1H, H-5′); ^13^C NMR (100 MHz, CDCl_3_): δ 23.72 (C-6), 25.53 (C-7), 38.91 (C-3), 44.66 (C-4), 56.01 (-OCH_3_), 87.93 (C-5), 111.45 (C-2′), 114.64 (C-5′), 121.20 (C-6′), 128.94 (C-1′), 145.33 (C-4′), 146.34 (C-3′), 176.67 (C-2); IR (ATR): ν_max_ = 3367, 1762, 1518, 1178, 1129, 1033, 966, 846, 597, 527 cm^−1^; HRMS (ESI): *m*/*z* calcd for C_13_H_15_O_4_I [M+H]^+^: 363.0093; found: 363.0094.

#### 3.11.2. *Trans*-4-(4′-Hydroxy-3′-methoxyphenyl)-5-(1-iodoethyl)dihydrofuran-2-one (**10b**)

Obtained from lactone **7b** (0.87 g, 2.40 mmol) in yield 0.11 g (16%) and from lactone **7c** (0.38 g, 1.05 mmol) in yield 0.05 g (18%); white solid; mp 39–41 °C; R_f_ = 0.16 (hexane/ethyl acetate 3:1, *v*/*v*); ^1^H NMR (600 MHz, CDCl_3_): δ 1.87 (d, *J* = 7.1 Hz, 3H, CH_3_-7), 2.64 (dd, *J* = 18.4 and 6.6 Hz, one of CH_2_-3), 3.13 (dd, *J* = 18.4 and 10.1 Hz, 1H, one of CH_2_-3), 3.56 (ddd, *J* = 10.1, 6.6 and 5.3 Hz, 1H, H-4), 3.90 (s, 3H, -OCH_3_), 4.23 (t, *J* = 5.3 Hz, 1H, H-5), 4.37 (qd, *J* = 7.1 and 5.3 Hz, 1H, H-6), 5.61 (s, 1H, -OH), 6.71 (d, *J* = 2.1 Hz, 1H, H-2′), 6.76 (dd, *J* = 8.1 and 2.1 Hz, 1H, H-6′), 6.89 (d, *J* = 8.1 Hz, 1H, H-5′); ^13^C NMR (150 MHz, CDCl_3_): δ 23.48 (C-7), 28.38 (C-6), 37.81 (C-3), 45.26 (C-4), 56.05 (-OCH_3_), 89.70 (C-5), 109.25 (C-2′), 115.00 (C-5′), 119.91 (C-6′), 133.26 (C-1′), 145.24 (C-4′), 147.07 (C-3′), 174.89 (C-2); IR (ATR): ν_max_ = 3406, 1770, 1516, 1269, 1148, 1026, 969, 816, 757, 596 cm^−1^; HRMS (ESI): *m*/*z* calcd for C_13_H_15_O_4_I [M+H]^+^: 363.0093; found: 363.0094.

#### 3.11.3. *Cis*-5-(1-Bromoethyl)-4-(4′-hydroxy-3′-methoxyphenyl)dihydrofuran-2-one (**11a**)

Obtained from lactone **8a** (1.12 g, 3.55 mmol) in yield 0.24 g (28%); white solid; mp 43–46 °C; R_f_ = 0.27 (hexane/chloroform/methanol 8:2:1, *v*/*v*); Rt = 6.84 min; ^1^H NMR (600 MHz, CDCl_3_): δ 1.76 (d, *J* = 6.6 Hz, 3H, CH_3_-7), 2.71 (d, *J* = 17.3 Hz, 1H, one of CH_2_-3), 3.10 (dd, *J* = 17.3 and 8.5 Hz, 1H, one of CH_2_-3), 3.46 (dq, *J* = 10.4 and 6.6 Hz, 1H, H-6), 3.81 (dd, *J* = 8.5 and 5.1 Hz, 1H, H-4), 3.88 (s, 3H, -OCH_3_), 4.64 (dd, *J* = 10.4 and 5.1 Hz, 1H, H-5), 5.62 (s, 1H, -OH), 6.71 (dd, *J* = 8.1 and 2.1 Hz, 1H, H-6′), 6.73 (d, *J* = 2.1 Hz, 1H, H-2′), 6.87 (d, *J* = 8.1 Hz, 1H, H-5′); ^13^C NMR (150 MHz, CDCl_3_): δ 23.26 (C-7), 38.36 (C-3), 43.74 (C-4), 45.56 (C-6), 55.97 (-OCH_3_), 86.76 (C-5), 111.23 (C-2′), 114.59 (C-5′), 121.00 (C-6′), 129.08 (C-1′), 145.32 (C-4′), 146.34 (C-3′), 176.47 (C-2); IR (ATR): ν_max_ = 3333, 1737, 1519, 1132, 967, 777, 703, 624 cm^−1^; HRMS (ESI): *m*/*z* calcd for C_13_H_15_O_4_Br [M+H]^+^: 315,0232; found: 315.0230.

#### 3.11.4. *Cis*-5-(1-Chloroethyl)-4-(4′-hydroxy-3′-methoxyphenyl)dihydrofuran-2-one (**12a**)

Obtained from lactone **9a** (0.28 g, 0.78 mmol) in yield 0.06 g (28%); white solid; mp 86–89 °C; R_f_ = 0.13 (hexane/chloroform/methanol 8:2:1, *v*/*v*); ^1^H NMR (600 MHz, CDCl_3_): δ 1.57 (d, *J* = 6.5 Hz, 3H, CH_3_-7), 2.72 (d, *J* = 17.6 Hz, 1H, one of CH_2_-3), 3.08 (dd, *J* = 17.6 and 8.5 Hz, 1H, one of CH_2_-3), 3.45 (dq, *J* = 10.1 and 6.5 Hz, 1H, H-6), 3.80 (dd, *J* = 8.5 and 5.2 Hz, 1H, H-4), 3.88 (s, 3H, -OCH_3_), 4.50 (dd, *J* = 10.1 and 5.2 Hz, 1H, H-5), 5.61 (s, 1H, -OH), 6.69–6.70 (two m, 2H, H-2′ and H-6′), 6.88 (d, *J* = 8.4 Hz, 1H, H-5′); ^13^C NMR (150 MHz, CDCl_3_): δ 22.38 (C-7), 38.02 (C-3), 43.30 (C-4), 53.96 (C-6), 55.97 (-OCH_3_), 86.65 (C-5), 111.10 (C-6′), 114.60 (C-5′), 120.96 (C-2′), 129.21 (C-1′), 145.35 (C-4′), 146.41 (C-3′), 176.33 (C-2); IR (ATR): ν_max_ = 3422, 1763, 1517, 1266, 1188, 1128, 1018, 974, 838, 785, 632, 600 cm^−1^; HRMS (ESI): *m*/*z* calcd for C_13_H_15_O_4_Cl [M-H]^−^: 269.0581; found: 269.0573.

#### 3.11.5. 5-*t*-Chloro-4-*r*-(4′-Hydroxy-3′-methoxyphenyl)-6-*c*-methyltetrahydropyran-2-one (**12b**)

Obtained from lactone **9b** (0.50 g, 1.39 mmol) in yield 0.07 g (19%); white solid; mp 52–55 °C; R_f_ = 0.06 (hexane/chloroform/methanol 8:2:1, *v*/*v*); ^1^H NMR (600 MHz, CDCl_3_): δ 1.60 (d, *J* = 6.2 Hz, 3H, CH_3_-7), 2.73 (dd, *J* = 17.8 and 10 Hz, 1H, one of CH_2_-3), 3.06 (dd, *J* = 17.8 and 7.0 Hz, 1H, one of CH_2_-3), 3.27 (td, *J* = 10.0 and 7.0 Hz, 1H, H-4), 3.83 (t, *J* = 10.0 Hz, H-5), 3.90 (s, 3H, -OCH_3_), 4.50 (dq, *J* = 10.0 and 6.2 Hz, 1H, H-6), 5.62 (s, 1H, -OH), 6.68 (d, *J* = 2.0 Hz, 1H, H-2′), 6.72 (dd, *J* = 8.1 and 2.0 Hz, 1H, H-6′), 6.91 (d, *J* = 8.1 Hz, 1H, H-5′); ^13^C NMR (150 MHz, CDCl_3_): δ 19.76 (C-7), 37.33 (C-3), 46.44 (C-4), 55.97 (-OCH_3_), 62.64 (C-5), 79.37 (C-6), 109.66 (C-2′), 115.04(C-5′), 119.84 (C-6′), 132.08 (C-1′), 145.30 (C-4′), 146.83 (C-3′), 169.30 (C-2); IR (ATR): ν_max_ = 3394, 1728, 1518, 1215, 1054, 1030, 975, 735, 643, 578 cm^−1^; HRMS (ESI): *m*/*z* calcd for C_13_H_15_O_4_Cl [M+H]^+^: 271.0737; found: 271.0740.

### 3.12. Antiproliferative Activity

#### 3.12.1. Chemicals for Biological Tests

The RPMI-1640 (Roswell Park Memorial Institute), McCoy′s 5A, Eagle′s Minimum Essential Medium (EMEM), Dulbecco′s Modified Eagle′s Medium High Glucose (DMEM HG), L-glutamine (L-Glu), DMSO (biological grade), 3-(4,5-dimethylthiazol-2-yl)-2,5-diphenyltetrazolium bromide (MTT) were purchased from Sigma-Aldrich^®^ (Steinheim, Germany). The Fetal Bovine Serum (FBS) and advanced RPMI medium were purchased from Gibco/ThermoFisher (Waltham, MA, USA). Antibiotics penicillin-streptomycin (PS) was purchased from Corning (Manassas, VA**,** USA). The T-75 cell culture flasks (EasYFlask Nunclon Delta Surface) and 96-well plates were purchased from NUNC (Kamstrupvej, Denmark).

#### 3.12.2. Cell Lines and Cell Cultures

The CLB70 cell line (canine chronic B-cell lymphocytic leukemia) was established at the Department of Pharmacology and Toxicology, Wrocław University of Environmental and Life Sciences [62]. The CLBL-1 cell line (canine B-cell lymphoma) was kindly provided by Dr. Barbara C. Rütgen from the Institute of Immunology, Department of Pathobiology, University of Veterinary Medicine, Vienna [66]. The T-24 (human bladder carcinoma), Caco-2 (human colorectal adenocarcinoma), and NIH/3T3 (mouse embryonic fibroblasts) cell lines were purchased from the American Type Culture Collection (ATCC, Rockville, MD, USA).

Cell cultures were maintained in their respective growth media, with fetal bovine serum (FBS) concentrations ranging from 10% to 20%, depending on the cell line. Penicillin-streptomycin (PS) and L-glutamine (L-Glu) were consistently added at 1% each to all media. CLB70 cells were cultured in RPMI ADVANCE medium supplemented with 20% FBS, while CLBL-1 cells were maintained in RPMI-1640 medium with 20% FBS. T-24 cells were cultured in McCoy′s 5A medium with 10% FBS, and Caco-2 cells in Eagle′s Minimum Essential Medium (EMEM) with 20% FBS. NIH/3T3 cells were maintained in high-glucose DMEM (DMEM HG) with 10% FBS. All cell cultures were grown in T-75 flasks under a humidified atmosphere at 37 °C and 5% CO_2_ and passaged every 3 days to maintain optimal cell density (70–80% confluence).

#### 3.12.3. MTT Assay

Stock solutions used in the experiments were freshly prepared for each trial by dissolving the tested compounds in biological-grade DMSO. These stock solutions were then diluted with the culture medium appropriate for each specific cell line to achieve the desired final concentrations, which ranged from 3.125 to 100 µM. The final DMSO concentration in each well was maintained below 1%, a level generally considered non-toxic to cells [67]. The MTT assay was employed to evaluate the antiproliferative and cytotoxic effects of the compounds on cancer cell lines, following a previously described protocol with minor modifications [32]. Cells were seeded in 96-well plates at the following densities: 1.5 × 10^4^ cells/mL (NIH/3T3), 2 × 10^4^ cells/mL (T-24, Caco-2), and 3 × 10^5^ cells/mL (CLB70, CLBL-1). Each plate included control wells with untreated cells and wells with vehicle controls (culture medium specific to each cell line).

After 72 h of treatment with the tested compounds, 10 µL of 3-(4,5-dimethylthiazol-2-yl)-2,5-diphenyltetrazolium bromide (MTT) solution (5 mg/mL) was added to each well. The cells were subsequently incubated with MTT for an additional 3 h to allow the formation of formazan crystals. Following this, 40 µL of lysis buffer (composed of 46 g sodium dodecyl sulfate, 150 mL dimethylformamide, and 183.2 mL Milli-Q water) was added to each well to dissolve the formazan. After a 24 h incubation at room temperature, the optical density (OD) of the resulting formazan product was measured using a spectrophotometric microplate reader (Spark, Tecan, Männedorf, Switzerland) at 570 nm. To correct for background absorbance and minimize potential measurement artifacts, a reference wavelength of 630 nm, at which formazan exhibits negligible absorbance, was used.

The results were determined from three independent experiments (four wells each) and presented as mean IC_50_ value ± SD from at least four independent experiments, each performed in quadruplicate. IC_50_ values (the concentration of the compound required to inhibit 50% of cell growth) were calculated from dose–response curves.

### 3.13. Cytotoxicity Against Red Blood Cells (RBCs)

To evaluate the hemolytic activity and cytotoxicity of the tested compounds, a quantitative hemolysis assay was conducted using human red blood cells (RBCs), following the method described by Pruchnik et al., with minor modifications [68].

RBCs were obtained from healthy donors through the Blood Donation Center in Wrocław. In accordance with Polish law, the use of erythrocytes from blood centers for experimental purposes does not require approval from an ethics committee. The collected RBCs were gently resuspended and washed three times with cold isotonic phosphate-buffered saline (PBS, pH 7.4) to remove plasma and cellular debris.

All tested compounds were dissolved in biological-grade DMSO. A series of working solutions was prepared by diluting the stock solutions to final concentrations of 6.25, 12.50, 25, 50, 100, and 200 μM. The DMSO content in all experimental and control samples did not exceed 1% (*v*/*v*). Control samples contained no tested compounds, while vehicle control samples contained DMSO at the same concentration as the treated samples. Positive control samples (A_100_%) were prepared by inducing complete hemolysis with Milli-Q water. All experiments were performed in triplicate, with three independent replicates for each condition.

The erythrocyte suspension was adjusted to a final hematocrit of 1.2% in all test tubes, with a total sample volume of 1 mL. Samples were incubated at 37 °C under static conditions for 2, 24, and 72 h to assess both short- and long-term cytotoxic effects.

Following incubation, all samples were centrifuged at 5000 rpm for 3 min at room temperature. The supernatants were carefully transferred to a 96-well plate, and the absorbance of free hemoglobin was measured at 540 nm using a UV-Vis microplate spectrophotometer (EPOCH, BioTek, Santa Clara, CA, USA). The degree of in vitro hemolysis was calculated based on the amount of released hemoglobin relative to that in the completely hemolyzed positive control sample, according to the following formula:

$$H\left[\%\right]=\frac{A_{s}}{A_{100\%}}\times 100\%,$$

where *H* [%] is the percentage of erythrocyte hemolysis, *A_s_* is absorbance of hemoglobin in supernatant at 540 nm, and *A*_100%_ refers to the absorbance of hemoglobin in the supernatant following complete hemolysis of the erythrocytes.

## 4. Conclusions

This study presents the design, synthesis, and biological evaluation of five novel vanillin-derived γ-halo-δ-lactones and δ-halo-γ-lactones bearing a phenolic substituent at the β-position, obtained via a seven-step synthetic pathway. All compounds exhibited antiproliferative activity against canine cell lines (CLBL-1, CLB70). Iodolactones **10a** and **10b** demonstrated activity against the human T-24 cell line, whereas bromolactone **11a** and chlorolactones **12a** and **12b** were active against the Caco-2 cancer cell line. Importantly, all lactones showed no cytotoxic effects on normal mouse fibroblasts (NIH/3T3). Hemolysis assays further confirmed their safety profile, indicating no toxicity against human red blood cells (RBCs) and preservation of erythrocyte membrane integrity. Among the tested compounds, *trans*-δ-iodo-γ-lactone **10b** showed the most promising biological activity.

To further elucidate the anticancer potential of this compound, we plan to develop a chemoenzymatic synthetic pathway to obtain both enantiomers of lactone **10b** and determine which enantiomer is responsible for the antiproliferative activity. Subsequently, the active enantiomer will be thoroughly investigated to elucidate its mechanism of action in detail. In particular, we will assess its ability to induce programmed cell death through apoptosis-related pathways, including caspase activation and mitochondrial membrane potential disruption. In parallel, we will examine DNA damage induction using standard assays such as γH2AX staining. Considering the chemical structure of vanillin-derived lactones and their reported bioactivity, we will also explore potential mechanisms, including cell cycle arrest and oxidative stress modulation, that contribute to the compound′s anticancer effects. These studies will provide mechanistic insights that support further preclinical development. Understanding these fundamental aspects will be critical to rationally guide further optimization and application. Additionally, all vanillin-derived halolactones will be subjected to further studies to evaluate their potential antioxidant and anti-inflammatory activities, as the presence of a phenolic fragment in their structures suggests broader, multi-target biological relevance.

Subsequently, efforts will focus on the design and synthesis of phospholipid-based liposomal nanocarriers incorporating the most biologically active lactones to enhance their targeted delivery and therapeutic efficacy against cancer cells.

## Figures and Tables

**Table 1:** Antiproliferative activity and selectivity index (SI) of vanillin (**1**) and vanillin-derived halolactones (**10a,b**, **11a**, **12a,b**) against CLBL-1, CLB70, T-24, CaCo-2 and NIH/3T3 cell lines after 72 h of exposure.

Compound	Cell Line								
	CLBL-1		CLB70		T-24		CaCo-2		NIH/3T3
	IC_50_ [µM]	SI	IC_50_ [µM]	SI	IC_50_ [µM]	SI	IC_50_ [µM]	SI	IC_50_ [µM]
**1**	72.4 ± 9.4 ^1^	>1.4 ^2^	72.8 ± 2.3	>1.4	48.5 ± 13.2	>2.1	>100	-	>100
**10a**	73.6 ± 8.8	>1.4	69.7 ± 13.7	>1.4	98.1 ± 10.4	>1.0	>100	-	>100
**10b**	46.3 ± 4.1	>2.2	71.5 ± 7.5	>1.4	63.4 ± 6.2	>1.6	>100	-	>100
**11a**	63.2 ± 1.6	>1.6	76.2 ± 5.3	>1.3	>100	-	76.4 ± 10.0	>1.3	>100
**12a**	76.7 ± 11.5	>1.3	75.2 ± 6.3	>1.3	>100	-	88.1 ± 4.4	>1.1	>100
**12b**	85.7 ± 15.7	>1.2	71.2 ± 11.6	>1.4	>100	-	72.5 ± 19.8	>1.4	>100

^1^ Data represent mean values ± SD from at least four independent experiments, 3 wells each. ^2^ SI calculated as the ratio of the IC_50_ value obtained for the normal cell line (NIH/3T3) to that measured for the corresponding cancer cell line.

## Data Availability

The datasets used and/or analyzed during the current study are available from the corresponding author upon reasonable request.

## Supplementary Materials

The following supporting information can be downloaded at: https://www.mdpi.com/article/10.3390/molecules30214180/s1, Figure S1: ^1^H NMR spectrum of benzylvanillin (**2**)**;** Figure S2: ^13^C NMR spectrum of benzylvanillin (**2**); Figure S3: DEPT 135 NMR spectrum of benzylvanillin (**2**); Figure S4: COSY spectrum of benzylvanillin (**2**); Figure S5: HMQC spectrum of benzylvanillin (**2**); Figure S6: HMBC spectrum of benzylvanillin (**2**); Figure S7: ^1^H NMR spectrum of (*E*)-4-(4′-(benzyloxy-3′-methoxyphenyl)but-3-en-2-one (**3**); Figure S8: ^13^C NMR spectrum of (*E*)-4-(4′-(benzyloxy-3′-methoxyphenyl)but-3-en-2-one (**3**); Figure S9: DEPT 135 NMR spectrum of (*E*)-4-(4′-(benzyloxy-3′-methoxyphenyl)but-3-en-2-one (**3**); Figure S10: COSY spectrum of (*E*)-4-(4′-(benzyloxy-3′-methoxyphenyl)but-3-en-2-one (**3**); Figure S11: HMQC spectrum of (*E*)-4-(4′-(benzyloxy-3′-methoxyphenyl)but-3-en-2-one (**3**); Figure S12: HMBC spectrum of (*E*)-4-(4′-(benzyloxy-3′-methoxyphenyl)but-3-en-2-one (**3**); Figure S13: ^1^H NMR spectrum of (*E*)-4-(4′-benzyloxy-3′-methoxyphenyl)but-3-en-2-ol (**4**); Figure S14: ^13^C NMR spectrum of (*E*)-4-(4′-benzyloxy-3′-methoxyphenyl)but-3-en-2-ol (**4**); Figure S15: DEPT 135 NMR spectrum of (*E*)-4-(4′-benzyloxy-3′-methoxyphenyl)but-3-en-2-ol (**4**); Figure S16: COSY spectrum of (*E*)-4-(4′-benzyloxy-3′-methoxyphenyl)but-3-en-2-ol (**4**); Figure S17: HMQC spectrum of (*E*)-4-(4′-benzyloxy-3′-methoxyphenyl)but-3-en-2-ol (**4**); Figure S18: HMBC spectrum of (*E*)-4-(4′-benzyloxy-3′-methoxyphenyl)but-3-en-2-ol (**4**); Figure S19: ^1^H NMR spectrum of (*E*)-3-(4′-benzyloxy-3′-methoxyphenyl)hex-4-enoate (**5**); Figure S20: ^13^C NMR spectrum of (*E*)-3-(4′-benzyloxy-3′-methoxyphenyl)hex-4-enoate (**5**); Figure S21: DEPT 135 NMR spectrum of (*E*)-3-(4′-benzyloxy-3′-methoxyphenyl)hex-4-enoate (**5**); Figure S22: COSY spectrum of (*E*)-3-(4′-benzyloxy-3′-methoxyphenyl)hex-4-enoate (**5**); Figure S23: HMQC spectrum of (*E*)-3-(4′-benzyloxy-3′-methoxyphenyl)hex-4-enoate (**5**); Figure S24: HMBC spectrum of (*E*)-3-(4′-benzyloxy-3′-methoxyphenyl)hex-4-enoate (**5**); Figure S25: ^1^H NMR spectrum of (*E*)-3-(4′-benzyloxy-3′-methoxyphenyl)hex-4-enoic acid (**6**); Figure S26: ^13^C NMR spectrum of (*E*)-3-(4′-benzyloxy-3′-methoxyphenyl)hex-4-enoic acid (**6**); Figure S27: DEPT 135 NMR spectrum of (*E*)-3-(4′-benzyloxy-3′-methoxyphenyl)hex-4-enoic acid (**6**); Figure S28: COSY spectrum of (*E*)-3-(4′-benzyloxy-3′-methoxyphenyl)hex-4-enoic acid (**6**); Figure S29: HMQC spectrum of (*E*)-3-(4′-benzyloxy-3′-methoxyphenyl)hex-4-enoic acid (**6**); Figure S30: HMBC spectrum of (*E*)-3-(4′-benzyloxy-3′-methoxyphenyl)hex-4-enoic acid (**6**); Figure S31: ^1^H NMR spectrum of *cis*-4-(4′-benzyloxy-3′-methoxyphenyl)-5-(1-iodoethyl)dihydrofuran-2-one (**7a**); Figure S32: ^13^C NMR spectrum of *cis*-4-(4′-benzyloxy-3′-methoxyphenyl)-5-(1-iodoethyl)dihydrofuran-2-one (**7a**); Figure S33: DEPT NMR spectrum of *cis*-4-(4′-benzyloxy-3′-methoxyphenyl)-5-(1-iodoethyl)dihydrofuran-2-one (**7a**); Figure S34: COSY spectrum of *cis*-4-(4′-benzyloxy-3′-methoxyphenyl)-5-(1-iodoethyl)dihydrofuran-2-one (**7a**); Figure S35: HMQC spectrum of *cis*-4-(4′-benzyloxy-3′-methoxyphenyl)-5-(1-iodoethyl)dihydrofuran-2-one (**7a**); Figure S36: HMBC spectrum of *cis*-4-(4′-benzyloxy-3′-methoxyphenyl)-5-(1-iodoethyl)dihydrofuran-2-one (**7a**); Figure S37: ^1^H NMR spectrum of *trans*-4-(4′-benzyloxy-3′-methoxyphenyl)-5-(1-iodoethyl)dihydrofuran-2-one (**7b**); Figure S38: ^13^C NMR spectrum of *trans*-4-(4′-benzyloxy-3′-methoxyphenyl)-5-(1-iodoethyl)dihydrofuran-2-one (**7b**); Figure S39: DEPT 135 NMR spectrum of *trans*-4-(4′-benzyloxy-3′-methoxyphenyl)-5-(1-iodoethyl)dihydrofuran-2-one (**7b**); Figure S40: COSY spectrum of *trans*-4-(4′-benzyloxy-3′-methoxyphenyl)-5-(1-iodoethyl)dihydrofuran-2-one (**7b**); Figure S41: HMQC spectrum of *trans*-4-(4′-benzyloxy-3′-methoxyphenyl)-5-(1-iodoethyl)dihydrofuran-2-one (**7b**); Figure S42: HMBC spectrum of *trans*-4-(4′-benzyloxy-3′-methoxyphenyl)-5-(1-iodoethyl)dihydrofuran-2-one (**7b**); Figure S43: ^1^H NMR spectrum of 4-*r*-(4′-benzyloxy-3′-methoxyphenyl)-5-*t*-iodo-6-*c*-methyltetrahydropyran-2-one (**7c**); Figure S44: ^13^C NMR spectrum of 4-*r*-(4′-benzyloxy-3′-methoxyphenyl)-5-*t*-iodo-6-*c*-methyltetrahydropyran-2-one (**7c**); Figure S45: DEPT 135 NMR spectrum of 4-*r*-(4′-benzyloxy-3′-methoxyphenyl)-5-*t*-iodo-6-*c*-methyltetrahydropyran-2-one (**7c**); Figure S46: COSY spectrum of 4-*r*-(4′-benzyloxy-3′-methoxyphenyl)-5-*t*-iodo-6-*c*-methyltetrahydropyran-2-one (**7c**); Figure S47: HMQC spectrum of 4-*r*-(4′-benzyloxy-3′-methoxyphenyl)-5-*t*-iodo-6-*c*-methyltetrahydropyran-2-one (**7c**); Figure S48: HMBC spectrum of 4-*r*-(4′-benzyloxy-3′-methoxyphenyl)-5-*t*-iodo-6-*c*-methyltetrahydropyran-2-one (**7c**); Figure S49: ^1^H NMR spectrum of *cis*-4-(4′-benzyloxy-3′-methoxyphenyl)-5-(1-bromoethyl)dihydrofuran-2-one (**8a**); Figure S50: ^13^C NMR spectrum of *cis*-4-(4′-benzyloxy-3′-methoxyphenyl)-5-(1-bromoethyl)dihydrofuran-2-one (**8a**); Figure S51: DEPT 135 NMR spectrum of *cis*-4-(4′-benzyloxy-3′-methoxyphenyl)-5-(1-bromoethyl)dihydrofuran-2-one (**8a**); Figure S52: COSY spectrum of *cis*-4-(4′-benzyloxy-3′-methoxyphenyl)-5-(1-bromoethyl)dihydrofuran-2-one (**8a**); Figure S53: HMQC spectrum of *cis*-4-(4′-benzyloxy-3′-methoxyphenyl)-5-(1-bromoethyl)dihydrofuran-2-one (**8a**); Figure S54: HMBC spectrum of *cis*-4-(4′-benzyloxy-3′-methoxyphenyl)-5-(1-bromoethyl)dihydrofuran-2-one (**8a**); Figure S55: ^1^H NMR spectrum of 4-*r*-(4′-benzyloxy-3′-methoxyphenyl)-5-*t*-bromo-6-*c*-methyltetrahydropyran-2-one (**8b**); Figure S56: ^13^C NMR spectrum of 4-*r*-(4′-benzyloxy-3′-methoxyphenyl)-5-*t*-bromo-6-*c*-methyltetrahydropyran-2-one (**8b**); Figure S57: DEPT 135 NMR spectrum of 4-*r*-(4′-benzyloxy-3′-methoxyphenyl)-5-*t*-bromo-6-*c*-methyltetrahydropyran-2-one (**8b**); Figure S58: COSY spectrum of 4-*r*-(4′-benzyloxy-3′-methoxyphenyl)-5-*t*-bromo-6-*c*-methyltetrahydropyran-2-one (**8b**); Figure S59: HMQC spectrum of 4-*r*-(4′-benzyloxy-3′-methoxyphenyl)-5-*t*-bromo-6-*c*-methyltetrahydropyran-2-one (**8b**); Figure S60: HMBC spectrum of 4-*r*-(4′-benzyloxy-3′-methoxyphenyl)-5-*t*-bromo-6-*c*-methyltetrahydropyran-2-one (**8b**); Figure S61: ^1^H NMR spectrum of *cis*-4-(4′-benzyloxy-3′-methoxyphenyl)-5-(1-chloroethyl)dihydrofuran-2-one (**9a**); Figure S62: ^13^C NMR spectrum of *cis*-4-(4′-benzyloxy-3′-methoxyphenyl)-5-(1-chloroethyl)dihydrofuran-2-one (**9a**); Figure S63: DEPT 135 NMR spectrum of *cis*-4-(4′-benzyloxy-3′-methoxyphenyl)-5-(1-chloroethyl)dihydrofuran-2-one (**9a**); Figure S64: COSY spectrum of *cis*-4-(4′-benzyloxy-3′-methoxyphenyl)-5-(1-chloroethyl)dihydrofuran-2-one (**9a**); Figure S65: HMQC spectrum of *cis*-4-(4′-benzyloxy-3′-methoxyphenyl)-5-(1-chloroethyl)dihydrofuran-2-one (**9a**); Figure S66: HMBC spectrum of *cis*-4-(4′-benzyloxy-3′-methoxyphenyl)-5-(1-chloroethyl)dihydrofuran-2-one (**9a**); Figure S67: ^1^H NMR spectrum of 4-*r*-(4′-benzyloxy-3′-methoxyphenyl)-5-*t*-chloro-6-*c*-methyltetrahydropyran-2-one (**9b**); Figure S68: ^13^CNMR spectrum of 4-*r*-(4′-benzyloxy-3′-methoxyphenyl)-5-*t*-chloro-6-*c*-methyltetrahydropyran-2-one (**9b**); Figure S69: DEPT 135 NMR spectrum of 4-*r*-(4′-benzyloxy-3′-methoxyphenyl)-5-*t*-chloro-6-*c*-methyltetrahydropyran-2-one (**9b**); Figure S70: COSY spectrum of 4-*r*-(4′-benzyloxy-3′-methoxyphenyl)-5-*t*-chloro-6-*c*-methyltetrahydropyran-2-one (**9b**); Figure S71: HMQC spectrum of 4-*r*-(4′-benzyloxy-3′-methoxyphenyl)-5-*t*-chloro-6-*c*-methyltetrahydropyran-2-one (**9b**); Figure S72: HMBC spectrum of 4-*r*-(4′-benzyloxy-3′-methoxyphenyl)-5-*t*-chloro-6-*c*-methyltetrahydropyran-2-one (**9b**); Figure S73: ^1^H NMR spectrum of *cis*-4-(4′-hydroxy-3′-methoxyphenyl)-5-(1-iodoethyl)dihydrofuran-2-one (**10a**); Figure S74: ^13^C NMR spectrum of *cis*-4-(4′-hydroxy-3′-methoxyphenyl)-5-(1-iodoethyl)dihydrofuran-2-one (**10a**); Figure S75: DEPT 135 NMR spectrum of *cis*-4-(4′-hydroxy-3′-methoxyphenyl)-5-(1-iodoethyl)dihydrofuran-2-one (**10a**); Figure S76: COSY spectrum of *cis*-4-(4′-hydroxy-3′-methoxyphenyl)-5-(1-iodoethyl)dihydrofuran-2-one (**10a**); Figure S77: HMQC spectrum of *cis*-4-(4′-hydroxy-3′-methoxyphenyl)-5-(1-iodoethyl)dihydrofuran-2-one (**10a**); Figure S78: HMBC spectrum of *cis*-4-(4′-hydroxy-3′-methoxyphenyl)-5-(1-iodoethyl)dihydrofuran-2-one (**10a**); Figure S79: ^1^H NMR spectrum of *trans*-4-(4′-hydroxy-3′-methoxyphenyl)-5-(1-iodoethyl)dihydrofuran-2-one (**10b**); Figure S80: ^13^C NMR spectrum of *trans*-4-(4′-hydroxy-3′-methoxyphenyl)-5-(1-iodoethyl)dihydrofuran-2-one (**10b**); Figure S81: DEPT 135 NMR spectrum of *trans*-4-(4′-hydroxy-3′-methoxyphenyl)-5-(1-iodoethyl)dihydrofuran-2-one (**10b**); Figure S82: COSY spectrum of *trans*-4-(4′-hydroxy-3′-methoxyphenyl)-5-(1-iodoethyl)dihydrofuran-2-one (**10b**); Figure S83: HMQC spectrum of *trans*-4-(4′-hydroxy-3′-methoxyphenyl)-5-(1-iodoethyl)dihydrofuran-2-one (**10b**); Figure S84: HMBC spectrum of *trans*-4-(4′-hydroxy-3′-methoxyphenyl)-5-(1-iodoethyl)dihydrofuran-2-one (**10b**); Figure S85: ^1^H NMR spectrum of *cis*-5-(1-bromoethyl)-4-(4′-hydroxy-3′-methoxyphenyl)dihydrofuran-2-one (**11a**); Figure S86: ^13^C NMR spectrum of *cis*-5-(1-bromoethyl)-4-(4′-hydroxy-3′-methoxyphenyl)dihydrofuran-2-one (**11a**); Figure S87: DEPT 135 NMR spectrum of *cis*-5-(1-bromoethyl)-4-(4′-hydroxy-3′-methoxyphenyl)dihydrofuran-2-one (**11a**); Figure S88: COSY spectrum of *cis*-5-(1-bromoethyl)-4-(4′-hydroxy-3′-methoxyphenyl)dihydrofuran-2-one (**11a**); Figure S89: HMQC spectrum of *cis*-5-(1-bromoethyl)-4-(4′-hydroxy-3′-methoxyphenyl)dihydrofuran-2-one (**11a**); Figure S90: HMBC spectrum of *cis*-5-(1-bromoethyl)-4-(4′-hydroxy-3′-methoxyphenyl)dihydrofuran-2-one (**11a**); Figure S91: ^1^H NMR spectrum of *cis*-5-(1-chloroethyl)-4-(4′-hydroxy-3′-methoxyphenyl)dihydrofuran-2-one (**12a**); Figure S92: ^13^C NMR spectrum of *cis*-5-(1-chloroethyl)-4-(4′-hydroxy-3′-methoxyphenyl)dihydrofuran-2-one (**12a**); Figure S93: DEPT 135 NMR spectrum of *cis*-5-(1-chloroethyl)-4-(4′-hydroxy-3′-methoxyphenyl)dihydrofuran-2-one (**12a**); Figure S94: COSY spectrum of *cis*-5-(1-chloroethyl)-4-(4′-hydroxy-3′-methoxyphenyl)dihydrofuran-2-one (**12a**); Figure S95: HMQC spectrum of *cis*-5-(1-chloroethyl)-4-(4′-hydroxy-3′-methoxyphenyl)dihydrofuran-2-one (**12a**); Figure S96: HMBC spectrum of *cis*-5-(1-chloroethyl)-4-(4′-hydroxy-3′-methoxyphenyl)dihydrofuran-2-one (**12a**); Figure S97: ^1^H NMR spectrum of 5-*t*-chloro-4-*r*-(4′-hydroxy-3′-methoxyphenyl)-6-*c*-methyltetrahydropyran-2-one (**12b**); Figure S98: ^13^C NMR spectrum of 5-*t*-chloro-4-*r*-(4′-hydroxy-3′-methoxyphenyl)-6-*c*-methyltetrahydropyran-2-one (**12b**); Figure S99: DEPT 135 NMR spectrum of 5-*t*-chloro-4-*r*-(4′-hydroxy-3′-methoxyphenyl)-6-*c*-methyltetrahydropyran-2-one (**12b**); Figure S100: COSY spectrum of 5-*t*-chloro-4-*r*-(4′-hydroxy-3′-methoxyphenyl)-6-*c*-methyltetrahydropyran-2-one (**12b**); Figure S101: HMQC spectrum of 5-*t*-chloro-4-*r*-(4′-hydroxy-3′-methoxyphenyl)-6-*c*-methyltetrahydropyran-2-one (**12b**); Figure S102: HMBC spectrum of 5-*t*-chloro-4-*r*-(4′-hydroxy-3′-methoxyphenyl)-6-*c*-methyltetrahydropyran-2-one **(12b)**; Figure S103: IR spectrum of benzylvanillin (**2**); Figure S104: IR spectrum of (*E*)-4-(4′-(benzyloxy-3′-methoxyphenyl)but-3-en-2-one (**3**); Figure S105: IR spectrum of (*E*)-4-(4′-benzyloxy-3′-methoxyphenyl)but-3-en-2-ol (**4**); Figure S106: IR spectrum of (*E*)-3-(4′-benzyloxy-3′-methoxyphenyl)hex-4-enoate (**5**); Figure S107: IR spectrum of (*E*)-3-(4′-benzyloxy-3′-methoxyphenyl)hex-4-enoic acid (**6**); Figure S108: IR spectrum of *cis*-4-(4′-benzyloxy-3′-methoxyphenyl)-5-(1-iodoethyl)dihydrofuran-2-one (**7a**); Figure S109: IR spectrum of *trans*-4-(4′-benzyloxy-3′-methoxyphenyl)-5-(1-iodoethyl)dihydrofuran-2-one (**7b**); Figure S110: IR spectrum of 4-*r*-(4′-benzyloxy-3′-methoxyphenyl)-5-*t*-iodo-6-*c*-methyltetrahydropyran-2-one (**7c**); Figure S111: IR spectrum of *cis*-4-(4′-benzyloxy-3′-methoxyphenyl)-5-(1-bromoethyl)dihydrofuran-2-one (**8a**); Figure S112: IR spectrum of 4-*r*-(4′-benzyloxy-3′-methoxyphenyl)-5-*t*-bromo-6-*c*-methyltetrahydropyran-2-one (**8b**); Figure S113: IR spectrum of *cis*-4-(4′-benzyloxy-3′-methoxyphenyl)-5-(1-chloroethyl)dihydrofuran-2-one (**9a**); Figure S114: IR spectrum of 4-*r*-(4′-benzyloxy-3′-methoxyphenyl)-5-*t*-chloro-6-*c*-methyltetrahydropyran-2-one (**9b**); Figure S115: IR spectrum of *cis*-4-(4′-hydroxy-3′-methoxyphenyl)-5-(1-iodoethyl)dihydrofuran-2-one (**10a**); Figure S116: IR spectrum of *trans*-4-(4′-hydroxy-3′-methoxyphenyl)-5-(1-iodoethyl)dihydrofuran-2-one (**10b**); Figure S117: IR spectrum of *cis*-5-(1-bromoethyl)-4-(4′-hydroxy-3′-methoxyphenyl)dihydrofuran-2-one (**11a**); Figure S118: IR spectrum of *cis*-5-(1-chloroethyl)-4-(4′-hydroxy-3′-methoxyphenyl)dihydrofuran-2-one (**12a**); Figure S119: IR spectrum of 5-*t*-chloro-4-*r*-(4′-hydroxy-3′-methoxyphenyl)-6-*c*-methyltetrahydropyran-2-one (**12b**); Figure S120: HRMS spectrum of benzylvanillin (**2**); Figure S121: HRMS spectrum of (E)-4-(4′-(benzyloxy-3′-methoxyphenyl)but-3-en-2-one (**3**); Figure S122: HRMS spectrum of (E)-4-(4′-benzyloxy-3′-methoxyphenyl)but-3-en-2-ol (**4**); Figure S123: HRMS spectrum of (E)-3-(4′-benzyloxy-3′-methoxyphenyl)hex-4-enoate (**5**); Figure S124: HRMS spectrum of (E)-3-(4′-benzyloxy-3′-methoxyphenyl)hex-4-enoic acid (**6**); Figure S125: HRMS spectrum of *cis*-4-(4′-benzyloxy-3′-methoxyphenyl)-5-(1-iodoethyl)dihydrofuran-2-one (**7a**); Figure S126: HRMS spectrum of *trans*-4-(4′-benzyloxy-3′-methoxyphenyl)-5-(1-iodoethyl)dihydrofuran-2-one (**7b**); Figure S127: HRMS spectrum of 4-r-(4′-benzyloxy-3′-methoxyphenyl)-5-t-iodo-6-c-methyltetrahydropyran-2-one (**7c**); Figure S128: HRMS spectrum of *cis*-4-(4′-benzyloxy-3′-methoxyphenyl)-5-(1-bromoethyl)dihydrofuran-2-one (**8a**); Figure S129: HRMS spectrum of 4-r-(4′-benzyloxy-3′-methoxyphenyl)-5-t-bromo-6-c-methyltetrahydropyran-2-one (**8b**); Figure S130: HRMS spectrum of *cis*-4-(4′-benzyloxy-3′-methoxyphenyl)-5-(1-chloroethyl)dihydrofuran-2-one (**9a**); Figure S131: HRMS spectrum of 4-r-(4′-benzyloxy-3′-methoxyphenyl)-5-t-chloro-6-c-methyltetrahydropyran-2-one (**9b**); Figure S132: HRMS spectrum of *cis*-4-(4′-hydroxy-3′-methoxyphenyl)-5-(1-iodoethyl)dihydrofuran-2-one (**10a**); Figure S133: HRMS spectrum of *trans*-4-(4′-hydroxy-3′-methoxyphenyl)-5-(1-iodoethyl)dihydrofuran-2-one (**10b**); Figure S134: HRMS spectrum of *cis*-5-(1-bromoethyl)-4-(4′-hydroxy-3′-methoxyphenyl)dihydrofuran-2-one (**11a**); Figure S135: HRMS spectrum of *cis*-5-(1-chloroethyl)-4-(4′-hydroxy-3′-methoxyphenyl)dihydrofuran-2-one (**12a**); Figure S136: HRMS spectrum of 5-t-chloro-4-r-(4′-hydroxy-3′-methoxyphenyl)-6-c-methyltetrahydropyran-2-one (**12b**); Page. 74: Spectral data of benzylvanillin (**2**); Page. 74: Spectral data of (*E*)-4-(4′-(benzyloxy-3′-methoxyphenyl)but-3-en-2-one (**3**).
