# Supplementary material for: Novel Halolactones Derived from Vanillin: Design, Synthesis, Structural Characterization, and Evaluation of Antiproliferative and Hemolytic Activities
Source: Molecules. 2025 Oct 25;30(21):4180. doi: 10.3390/molecules30214180 (PMC12609600; doi:10.3390/molecules30214180)
Supplement: Supplementary file 1 [file molecules-30-04180-s001.zip › molecules-3910490-supplementary.pdf]

*Supplementary Information*

# Novel Halolactones Derived from Vanillin: Design, Synthesis, Structural Characterization, and Evaluation of Antiproliferative and Hemolytic Activities

Anna Dunal[1,*], Witold Gładkowski[1,*], Ewa Dejnaka[2], Joanna Sulecka-Zadka[2], Aleksandra Pawlak[2], Aleksandra Włoch[3], Hanna Pruchnik[3], Gabriela Maciejewska[4]

[1] Department of Food Chemistry and Biocatalysis, Wrocław University of Environmental and Life Sciences, Norwida 25, 50-375 Wrocław, Poland; witold.gladkowski@upwr.edu.pl

[2] Department of Pharmacology and Toxicology, Faculty of Veterinary Medicine, Wrocław University of Environmental and Life Sciences, Norwida 31, 50-375, Wrocław, Poland; joanna.sulecka-zadka@upwr.edu.pl (J.S-Z.); aleksandra.pawlak@upwr.edu.pl (A.P.); ewa.dejnaka@upwr.edu.pl (E.D.)

[3] Department of Physics and Biophysics, Faculty of Biotechnology and Food Sciences, Wrocław University of Environmental and Life Sciences, Norwida 25, 50-375, Wrocław, Poland; aleksandra.wloch@upwr.edu.pl (A.W.); hanna.pruchnik@upwr.edu.pl (H.P.)

[4] Omics Research Center, Wrocław Medical University, 50-368 Wrocław, Poland; gabriela.maciejewska@umw.edu.pl

* Correspondence: anna.dunal@upwr.edu.pl;

# Table of content


| | Pages |
|---|---|
| $^{1}H$ NMR spectrum of benzylvanillin **(2)** (Fig.S1) | 7 |
| $^{13}C$ NMR spectrum of benzylvanillin **(2)** (Fig.S2) | 7 |
| DEPT 135 NMR spectrum of benzylvanillin **(2)** (Fig.S3) | 8 |
| COSY spectrum of benzylvanillin **(2)** (Fig.S4) | 8 |
| HMQC spectrum of benzylvanillin **(2)** (Fig.S5) | 9 |
| HMBC spectrum of benzylvanillin **(2)** (Fig.S6) | 9 |
| $^{1}H$ NMR spectrum of (*E*)-4-(4'-(benzyloxy-3'-methoxyphenyl)but-3-en-2-one **(3)** (Fig.S7) | 10 |
| $^{13}C$ NMR spectrum of (*E*)-4-(4'-(benzyloxy-3'-methoxyphenyl)but-3-en-2-one **(3)** (Fig.S8) | 10 |
| DEPT 135 NMR spectrum of (*E*)-4-(4'-(benzyloxy-3'-methoxyphenyl)but-3-en-2-one **(3)** (Fig.S9) | 11 |
| COSY spectrum of (*E*)-4-(4'-(benzyloxy-3'-methoxyphenyl)but-3-en-2-one **(3)** (Fig.S10) | 11 |
| HMQC spectrum of (*E*)-4-(4'-(benzyloxy-3'-methoxyphenyl)but-3-en-2-one **(3)** (Fig.S11) | 12 |
| HMBC spectrum of (*E*)-4-(4'-(benzyloxy-3'-methoxyphenyl)but-3-en-2-one **(3)** (Fig.S12) | 12 |
| $^{1}H$ NMR spectrum of (*E*)-4-(4'-benzyloxy-3'-methoxyphenyl)but-3-en-2-ol **(4)** (Fig.S13) | 13 |
| $^{13}C$ NMR spectrum of (*E*)-4-(4'-benzyloxy-3'-methoxyphenyl)but-3-en-2-ol **(4)** (Fig.S14) | 13 |
| DEPT 135 NMR spectrum of (*E*)-4-(4'-benzyloxy-3'-methoxyphenyl)but-3-en-2-ol **(4)** (Fig.S15) | 14 |
| COSY spectrum of (*E*)-4-(4'-benzyloxy-3'-methoxyphenyl)but-3-en-2-ol **(4)** (Fig.S16) | 14 |
| HMQC spectrum of (*E*)-4-(4'-benzyloxy-3'-methoxyphenyl)but-3-en-2-ol **(4)** (Fig.S17) | 15 |
| HMBC spectrum of (*E*)-4-(4'-benzyloxy-3'-methoxyphenyl)but-3-en-2-ol **(4)** (Fig.S18) | 15 |
| $^{1}H$ NMR spectrum of (*E*)-3-(4'-benzyloxy-3'-methoxyphenyl)hex-4-enoate **(5)** (Fig.S19) | 16 |
| $^{13}C$ NMR spectrum of (*E*)-3-(4'-benzyloxy-3'-methoxyphenyl)hex-4-enoate **(5)** (Fig.S20) | 16 |
| DEPT 135 NMR spectrum of (*E*)-3-(4'-benzyloxy-3'-methoxyphenyl)hex-4-enoate **(5)** (Fig.S21) | 17 |
| COSY spectrum of (*E*)-3-(4'-benzyloxy-3'-methoxyphenyl)hex-4-enoate **(5)** (Fig.S22) | 17 |
| HMQC spectrum of (*E*)-3-(4'-benzyloxy-3'-methoxyphenyl)hex-4-enoate **(5)** (Fig.S23) | 18 |
| HMBC spectrum of (*E*)-3-(4'-benzyloxy-3'-methoxyphenyl)hex-4-enoate **(5)** (Fig.S24) | 18 |
| $^{1}H$ NMR spectrum of (*E*)-3-(4'-benzyloxy-3'-methoxyphenyl)hex-4-enoic acid **(6)** (Fig.S25) | 19 |
| $^{13}C$ NMR spectrum of (*E*)-3-(4'-benzyloxy-3'-methoxyphenyl)hex-4-enoic acid **(6)** (Fig.S26) | 19 |
| DEPT 135 NMR spectrum of (*E*)-3-(4'-benzyloxy-3'-methoxyphenyl)hex-4-enoic acid **(6)** (Fig.S27) | 20 |
| COSY spectrum of (*E*)-3-(4'-benzyloxy-3'-methoxyphenyl)hex-4-enoic acid **(6)** (Fig.S28) | 20 |
| HMQC spectrum of (*E*)-3-(4'-benzyloxy-3'-methoxyphenyl)hex-4-enoic acid **(6)** (Fig.S29) | 21 |
| HMBC spectrum of (*E*)-3-(4'-benzyloxy-3'-methoxyphenyl)hex-4-enoic acid **(6)** (Fig.S30) | 21 |

| | |
|---|---|
| $^{1}$H NMR spectrum of *cis*-4-(4'-benzyloxy-3'-methoxyphenyl)-5-(1-iodoethyl)dihydrofuran-2-one **(7a)** (Fig.S31) | 22 |
| $^{13}$C NMR spectrum of *cis*-4-(4'-benzyloxy-3'-methoxyphenyl)-5-(1-iodoethyl)dihydrofuran-2-one **(7a)** (Fig.S32) | 22 |
| DEPT NMR spectrum of *cis*-4-(4'-benzyloxy-3'-methoxyphenyl)-5-(1-iodoethyl)dihydrofuran-2-one **(7a)** (Fig.S33) | 23 |
| COSY spectrum of *cis*-4-(4'-benzyloxy-3'-methoxyphenyl)-5-(1-iodoethyl)dihydrofuran-2-one **(7a)** (Fig.S34) | 23 |
| HMQC spectrum of *cis*-4-(4'-benzyloxy-3'-methoxyphenyl)-5-(1-iodoethyl)dihydrofuran-2-one **(7a)** (Fig.S35) | 24 |
| HMBC spectrum of *cis*-4-(4'-benzyloxy-3'-methoxyphenyl)-5-(1-iodoethyl)dihydrofuran-2-one **(7a)** (Fig.S36) | 24 |
| $^{1}$H NMR spectrum of *trans*-4-(4'-benzyloxy-3'-methoxyphenyl)-5-(1-iodoethyl)dihydrofuran-2-one **(7b)** (Fig.S37) | 25 |
| $^{13}$C NMR spectrum of *trans*-4-(4'-benzyloxy-3'-methoxyphenyl)-5-(1-iodoethyl)dihydrofuran-2-one **(7b)** (Fig.S38) | 25 |
| DEPT 135 NMR spectrum of *trans*-4-(4'-benzyloxy-3'-methoxyphenyl)-5-(1-iodoethyl)dihydrofuran-2-one **(7b)** (Fig.S39) | 26 |
| COSY spectrum of *trans*-4-(4'-benzyloxy-3'-methoxyphenyl)-5-(1-iodoethyl)dihydrofuran-2-one **(7b)** (Fig.S40) | 26 |
| HMQC spectrum of *trans*-4-(4'-benzyloxy-3'-methoxyphenyl)-5-(1-iodoethyl)dihydrofuran-2-one **(7b)** (Fig.S41) | 27 |
| HMBC spectrum of *trans*-4-(4'-benzyloxy-3'-methoxyphenyl)-5-(1-iodoethyl)dihydrofuran-2-one **(7b)** (Fig.S42) | 27 |
| $^{1}$H NMR spectrum of 4-*r*-(4'-benzyloxy-3'-methoxyphenyl)-5-*t*-iodo-6-*c*-methyltetrahydropyran-2-one **(7c)** (Fig.S43) | 28 |
| $^{13}$C NMR spectrum of 4-*r*-(4'-benzyloxy-3'-methoxyphenyl)-5-*t*-iodo-6-*c*-methyltetrahydropyran-2-one **(7c)** (Fig.S44) | 28 |
| DEPT 135 NMR spectrum of 4-*r*-(4'-benzyloxy-3'-methoxyphenyl)-5-*t*-iodo-6-*c*-methyltetrahydropyran-2-one **(7c)** (Fig.S45) | 29 |
| COSY spectrum of 4-*r*-(4'-benzyloxy-3'-methoxyphenyl)-5-*t*-iodo-6-*c*-methyltetrahydropyran-2-one **(7c)** (Fig.S46) | 29 |
| HMQC spectrum of 4-*r*-(4'-benzyloxy-3'-methoxyphenyl)-5-*t*-iodo-6-*c*-methyltetrahydropyran-2-one **(7c)** (Fig.S47) | 30 |
| HMBC spectrum of 4-*r*-(4'-benzyloxy-3'-methoxyphenyl)-5-*t*-iodo-6-*c*-methyltetrahydropyran-2-one **(7c)** (Fig.S48) | 30 |
| $^{1}$H NMR spectrum of *cis*-4-(4'-benzyloxy-3'-methoxyphenyl)-5-(1-bromoethyl)dihydrofuran-2-one **(8a)** (Fig.S49) | 31 |
| $^{13}$C NMR spectrum of *cis*-4-(4'-benzyloxy-3'-methoxyphenyl)-5-(1-bromoethyl)dihydrofuran-2-one **(8a)** (Fig.S50) | 31 |
| DEPT 135 NMR spectrum of *cis*-4-(4'-benzyloxy-3'-methoxyphenyl)-5-(1-bromoethyl)dihydrofuran-2-one **(8a)** (Fig.S51) | 32 |
| COSY spectrum of *cis*-4-(4'-benzyloxy-3'-methoxyphenyl)-5-(1-bromoethyl)dihydrofuran-2-one **(8a)** (Fig.S52) | 32 |
| HMQC spectrum of *cis*-4-(4'-benzyloxy-3'-methoxyphenyl)-5-(1-bromoethyl)dihydrofuran-2-one **(8a)** (Fig.S53) | 33 |
| HMBC spectrum of *cis*-4-(4'-benzyloxy-3'-methoxyphenyl)-5-(1-bromoethyl)dihydrofuran-2-one **(8a)** (Fig.S54) | 33 |
| $^{1}$H NMR spectrum of 4-*r*-(4'-benzyloxy-3'-methoxyphenyl)-5-*t*-bromo-6-*c*-methyltetrahydropyran-2-one **(8b)** (Fig.S55) | 34 |
| $^{13}$C NMR spectrum of 4-*r*-(4'-benzyloxy-3'-methoxyphenyl)-5-*t*-bromo-6-*c*-methyltetrahydropyran-2-one **(8b)** (Fig.S56) | 34 |
| DEPT 135 NMR spectrum of 4-*r*-(4'-benzyloxy-3'-methoxyphenyl)-5-*t*-bromo-6-*c*-methyltetrahydropyran-2-one **(8b)** (Fig.S57) | 35 |
| COSY spectrum of 4-*r*-(4'-benzyloxy-3'-methoxyphenyl)-5-*t*-bromo-6-*c*-methyltetrahydropyran-2-one **(8b)** (Fig.S58) | 35 |
| HMQC spectrum of 4-*r*-(4'-benzyloxy-3'-methoxyphenyl)-5-*t*-bromo-6-*c*-methyltetrahydropyran-2-one **(8b)** (Fig.S59) | 36 |
| HMBC spectrum of 4-*r*-(4'-benzyloxy-3'-methoxyphenyl)-5-*t*-bromo-6-*c*-methyltetrahydropyran-2-one **(8b)** (Fig.S60) | 36 |
| $^{1}$H NMR spectrum of *cis*-4-(4'-benzyloxy-3'-methoxyphenyl)-5-(1-chloroethyl)dihydrofuran-2-one **(9a)** (Fig.S61) | 37 |
| $^{13}$C NMR spectrum of *cis*-4-(4'-benzyloxy-3'-methoxyphenyl)-5-(1-chloroethyl)dihydrofuran-2-one **(9a)** (Fig.S62) | 37 |

| | |
|---|---|
| DEPT 135 NMR spectrum of *cis*-4-(4'-benzyloxy-3'-methoxyphenyl)-5-(1-chloroethyl)dihydrofuran-2-one **(9a)** (Fig.S63) | 38 |
| COSY spectrum of *cis*-4-(4'-benzyloxy-3'-methoxyphenyl)-5-(1-chloroethyl)dihydrofuran-2-one **(9a)** (Fig.S64) | 38 |
| HMQC spectrum of *cis*-4-(4'-benzyloxy-3'-methoxyphenyl)-5-(1-chloroethyl)dihydrofuran-2-one **(9a)** (Fig.S65) | 39 |
| HMBC spectrum of *cis*-4-(4'-benzyloxy-3'-methoxyphenyl)-5-(1-chloroethyl)dihydrofuran-2-one **(9a)** (Fig.S66) | 39 |
| $^1$H NMR spectrum of 4-*r*-(4'-benzyloxy-3'-methoxyphenyl)-5-*t*-chloro-6-*c*-methyltetrahydropyran-2-one **(9b)** (Fig.S67) | 40 |
| $^{13}$CNMR spectrum of 4-*r*-(4'-benzyloxy-3'-methoxyphenyl)-5-*t*-chloro-6-*c*-methyltetrahydropyran-2-one **(9b)** (Fig.S68) | 40 |
| DEPT 135 NMR spectrum of 4-*r*-(4'-benzyloxy-3'-methoxyphenyl)-5-*t*-chloro-6-*c*-methyltetrahydropyran-2-one **(9b)** (Fig.S69) | 41 |
| COSY spectrum of 4-*r*-(4'-benzyloxy-3'-methoxyphenyl)-5-*t*-chloro-6-*c*-methyltetrahydropyran-2-one **(9b)** (Fig.S70) | 41 |
| HMQC spectrum of 4-*r*-(4'-benzyloxy-3'-methoxyphenyl)-5-*t*-chloro-6-*c*-methyltetrahydropyran-2-one **(9b)** (Fig.S71) | 42 |
| HMBC spectrum of 4-*r*-(4'-benzyloxy-3'-methoxyphenyl)-5-*t*-chloro-6-*c*-methyltetrahydropyran-2-one **(9b)** (Fig.S72) | 42 |
| $^1$H NMR spectrum of *cis*-4-(4'-hydroxy-3'-methoxyphenyl)-5-(1-iodoethyl)dihydrofuran-2-one **(10a)** (Fig.S73) | 43 |
| $^{13}$C NMR spectrum of *cis*-4-(4'-hydroxy-3'-methoxyphenyl)-5-(1-iodoethyl)dihydrofuran-2-one **(10a)** (Fig.S74) | 43 |
| DEPT 135 NMR spectrum of *cis*-4-(4'-hydroxy-3'-methoxyphenyl)-5-(1-iodoethyl)dihydrofuran-2-one **(10a)** (Fig.S75) | 44 |
| COSY spectrum of *cis*-4-(4'-hydroxy-3'-methoxyphenyl)-5-(1-iodoethyl)dihydrofuran-2-one **(10a)** (Fig.S76) | 44 |
| HMQC spectrum of *cis*-4-(4'-hydroxy-3'-methoxyphenyl)-5-(1-iodoethyl)dihydrofuran-2-one **(10a)** (Fig.S77) | 45 |
| HMBC spectrum of *cis*-4-(4'-hydroxy-3'-methoxyphenyl)-5-(1-iodoethyl)dihydrofuran-2-one **(10a)** (Fig.S78) | 45 |
| $^1$H NMR spectrum of *trans*-4-(4'-hydroxy-3'-methoxyphenyl)-5-(1-iodoethyl)dihydrofuran-2-one **(10b)** (Fig.S79) | 46 |
| $^{13}$C NMR spectrum of *trans*-4-(4'-hydroxy-3'-methoxyphenyl)-5-(1-iodoethyl)dihydrofuran-2-one **(10b)** (Fig.S80) | 46 |
| DEPT 135 NMR spectrum of *trans*-4-(4'-hydroxy-3'-methoxyphenyl)-5-(1-iodoethyl)dihydrofuran-2-one **(10b)** (Fig.S81) | 47 |
| COSY spectrum of *trans*-4-(4'-hydroxy-3'-methoxyphenyl)-5-(1-iodoethyl)dihydrofuran-2-one **(10b)** (Fig.S82) | 47 |
| HMQC spectrum of *trans*-4-(4'-hydroxy-3'-methoxyphenyl)-5-(1-iodoethyl)dihydrofuran-2-one **(10b)** (Fig.S83) | 48 |
| HMBC spectrum of *trans*-4-(4'-hydroxy-3'-methoxyphenyl)-5-(1-iodoethyl)dihydrofuran-2-one **(10b)** (Fig.S84) | 48 |
| $^1$H NMR spectrum of *cis*-5-(1-bromoethyl)-4-(4'-hydroxy-3'-methoxyphenyl)dihydrofuran-2-one **(11a)** (Fig.S85) | 49 |
| $^{13}$C NMR spectrum of *cis*-5-(1-bromoethyl)-4-(4'-hydroxy-3'-methoxyphenyl)dihydrofuran-2-one **(11a)** (Fig.S86) | 49 |
| DEPT 135 NMR spectrum of *cis*-5-(1-bromoethyl)-4-(4'-hydroxy-3'-methoxyphenyl)dihydrofuran-2-one **(11a)** (Fig.S87) | 50 |
| COSY spectrum of *cis*-5-(1-bromoethyl)-4-(4'-hydroxy-3'-methoxyphenyl)dihydrofuran-2-one **(11a)** (Fig.S88) | 50 |
| HMQC spectrum of *cis*-5-(1-bromoethyl)-4-(4'-hydroxy-3'-methoxyphenyl)dihydrofuran-2-one **(11a)** (Fig.S89) | 51 |
| HMBC spectrum of *cis*-5-(1-bromoethyl)-4-(4'-hydroxy-3'-methoxyphenyl)dihydrofuran-2-one **(11a)** (Fig.S90) | 51 |
| $^1$H NMR spectrum of *cis*-5-(1-chloroethyl)-4-(4'-hydroxy-3'-methoxyphenyl)dihydrofuran-2-one **(12a)** (Fig.S91) | 52 |

| | |
|---|---|
| $^{13}$C NMR spectrum of *cis*-5-(1-chloroethyl)-4-(4'-hydroxy-3'-methoxyphenyl)dihydrofuran-2-one **(12a)** (Fig.S92) | 52 |
| DEPT 135 NMR spectrum of *cis*-5-(1-chloroethyl)-4-(4'-hydroxy-3'-methoxyphenyl)dihydrofuran-2-one **(12a)** (Fig.S93) | 53 |
| COSY spectrum of *cis*-5-(1-chloroethyl)-4-(4'-hydroxy-3'-methoxyphenyl)dihydrofuran-2-one **(12a)** (Fig.S94) | 53 |
| HMQC spectrum of *cis*-5-(1-chloroethyl)-4-(4'-hydroxy-3'-methoxyphenyl)dihydrofuran-2-one **(12a)** (Fig.S95) | 54 |
| HMBC spectrum of *cis*-5-(1-chloroethyl)-4-(4'-hydroxy-3'-methoxyphenyl)dihydrofuran-2-one **(12a)** (Fig.S96) | 54 |
| $^{1}$H NMR spectrum of 5-*t*-chloro-4-*r*-(4'-hydroxy-3'-methoxyphenyl)-6-*c*-methyltetrahydropyran-2-one **(12b)** (Fig.S97) | 55 |
| $^{13}$C NMR spectrum of 5-*t*-chloro-4-*r*-(4'-hydroxy-3'-methoxyphenyl)-6-*c*-methyltetrahydropyran-2-one **(12b)** (Fig.S98) | 55 |
| DEPT 135 NMR spectrum of 5-*t*-chloro-4-*r*-(4'-hydroxy-3'-methoxyphenyl)-6-*c*-methyltetrahydropyran-2-one **(12b)** (Fig.S99) | 56 |
| COSY spectrum of 5-*t*-chloro-4-*r*-(4'-hydroxy-3'-methoxyphenyl)-6-*c*-methyltetrahydropyran-2-one **(12b)** (Fig.S100) | 56 |
| HMQC spectrum of 5-*t*-chloro-4-*r*-(4'-hydroxy-3'-methoxyphenyl)-6-*c*-methyltetrahydropyran-2-one **(12b)** (Fig.S101) | 57 |
| HMBC spectrum of 5-*t*-chloro-4-*r*-(4'-hydroxy-3'-methoxyphenyl)-6-*c*-methyltetrahydropyran-2-one **(12b)** (Fig.S102) | 57 |
| IR spectrum of benzylvanillin **(2)** (Fig.S103) | 58 |
| IR spectrum of (*E*)-4-(4'-(benzyloxy-3'-methoxyphenyl)but-3-en-2-one **(3)** (Fig.S104) | 58 |
| IR spectrum of (*E*)-4-(4'-benzyloxy-3'-methoxyphenyl)but-3-en-2-ol **(4)** (Fig.S105) | 59 |
| IR spectrum of (*E*)-3-(4'-benzyloxy-3'-methoxyphenyl)hex-4-enoate **(5)** (Fig.S106) | 59 |
| IR spectrum of (*E*)-3-(4'-benzyloxy-3'-methoxyphenyl)hex-4-enoic acid **(6)** (Fig.S107) | 60 |
| IR spectrum of *cis*-4-(4'-benzyloxy-3'-methoxyphenyl)-5-(1-iodoethyl)dihydrofuran-2-one **(7a)** (Fig.S108) | 60 |
| IR spectrum of *trans*-4-(4'-benzyloxy-3'-methoxyphenyl)-5-(1-iodoethyl)dihydrofuran-2-one **(7b)** (Fig.S109) | 61 |
| IR spectrum of 4-*r*-(4'-benzyloxy-3'-methoxyphenyl)-5-*t*-iodo-6-*c*-methyltetrahydropyran-2-one **(7c)** (Fig.S110) | 61 |
| IR spectrum of *cis*-4-(4'-benzyloxy-3'-methoxyphenyl)-5-(1-bromoethyl)dihydrofuran-2-one **(8a)** (Fig.S111) | 62 |
| IR spectrum of 4-*r*-(4'-benzyloxy-3'-methoxyphenyl)-5-*t*-bromo-6-*c*-methyltetrahydropyran-2-one **(8b)** (Fig.S112) | 62 |
| IR spectrum of *cis*-4-(4'-benzyloxy-3'-methoxyphenyl)-5-(1-chloroethyl)dihydrofuran-2-one **(9a)** (Fig.S113) | 63 |
| IR spectrum of 4-*r*-(4'-benzyloxy-3'-methoxyphenyl)-5-*t*-chloro-6-*c*-methyltetrahydropyran-2-one **(9b)** (Fig.S114) | 63 |
| IR spectrum of *cis*-4-(4'-hydroxy-3'-methoxyphenyl)-5-(1-iodoethyl)dihydrofuran-2-one **(10a)** (Fig.S115) | 64 |
| IR spectrum of *trans*-4-(4'-hydroxy-3'-methoxyphenyl)-5-(1-iodoethyl)dihydrofuran-2-one **(10b)** (Fig.S116) | 64 |
| IR spectrum of *cis*-5-(1-bromoethyl)-4-(4'-hydroxy-3'-methoxyphenyl)dihydrofuran-2-one **(11a)** (Fig.S117) | 65 |
| IR spectrum of *cis*-5-(1-chloroethyl)-4-(4'-hydroxy-3'-methoxyphenyl)dihydrofuran-2-one **(12a)** (Fig.S118) | 65 |
| IR spectrum of 5-*t*-chloro-4-*r*-(4'-hydroxy-3'-methoxyphenyl)-6-*c*-methyltetrahydropyran-2-one **(12b)** (Fig.S119) | 66 |
| HRMS spectrum of benzylvanillin **(2)** (Fig.S120) | 66 |
| HRMS spectrum of (*E*)-4-(4'-(benzyloxy-3'-methoxyphenyl)but-3-en-2-one **(3)** (Fig.S121) | 67 |

| | |
|---|---|
| HRMS spectrum of (*E*)-4-(4'-benzyloxy-3'-methoxyphenyl)but-3-en-2-ol **(4)** (Fig.S122) | 67 |
| HRMS spectrum of (*E*)-3-(4'-benzyloxy-3'-methoxyphenyl)hex-4-enoate **(5)** (Fig.S123) | 67 |
| HRMS spectrum of (*E*)-3-(4'-benzyloxy-3'-methoxyphenyl)hex-4-enoic acid **(6)** (Fig.S124) | 68 |
| HRMS spectrum of *cis*-4-(4'-benzyloxy-3'-methoxyphenyl)-5-(1-iodoethyl)dihydrofuran-2-one **(7a)** (Fig.S125) | 68 |
| HRMS spectrum of *trans*-4-(4'-benzyloxy-3'-methoxyphenyl)-5-(1-iodoethyl)dihydrofuran-2-one **(7b)** (Fig.S126) | 68 |
| HRMS spectrum of 4-*r*-(4'-benzyloxy-3'-methoxyphenyl)-5-*t*-iodo-6-*c*-methyltetrahydropyran-2-one **(7c)** (Fig.S127) | 69 |
| HRMS spectrum of *cis*-4-(4'-benzyloxy-3'-methoxyphenyl)-5-(1-bromoethyl)dihydrofuran-2-one **(8a)** (Fig.S128) | 69 |
| HRMS spectrum of 4-*r*-(4'-benzyloxy-3'-methoxyphenyl)-5-*t*-bromo-6-*c*-methyltetrahydropyran-2-one **(8b)** (Fig.S129) | 69 |
| HRMS spectrum of *cis*-4-(4'-benzyloxy-3'-methoxyphenyl)-5-(1-chloroethyl)dihydrofuran-2-one **(9a)** (Fig.S130) | 70 |
| HRMS spectrum of 4-*r*-(4'-benzyloxy-3'-methoxyphenyl)-5-*t*-chloro-6-*c*-methyltetrahydropyran-2-one **(9b)** (Fig.S131) | 70 |
| HRMS spectrum of *cis*-4-(4'-hydroxy-3'-methoxyphenyl)-5-(1-iodoethyl)dihydrofuran-2-one **(10a)** (Fig.S132) | 71 |
| HRMS spectrum of *trans*-4-(4'-hydroxy-3'-methoxyphenyl)-5-(1-iodoethyl)dihydrofuran-2-one **(10b)** (Fig.S133) | 71 |
| HRMS spectrum of *cis*-5-(1-bromoethyl)-4-(4'-hydroxy-3'-methoxyphenyl)dihydrofuran-2-one **(11a)** (Fig.S134) | 72 |
| HRMS spectrum of *cis*-5-(1-chloroethyl)-4-(4'-hydroxy-3'-methoxyphenyl)dihydrofuran-2-one **(12a)** (Fig.S135) | 72 |
| HRMS spectrum of 5-*t*-chloro-4-*r*-(4'-hydroxy-3'-methoxyphenyl)-6-*c*-methyltetrahydropyran-2-one **(12b)** (Fig.S136) | 73 |
| Spectral data of benzylvanillin (**2**) | 74 |
| Spectral data of (*E*)-4-(4'-(benzyloxy-3'-methoxyphenyl)but-3-en-2-one (**3**) | 74 |

# NMR SPECTRA:

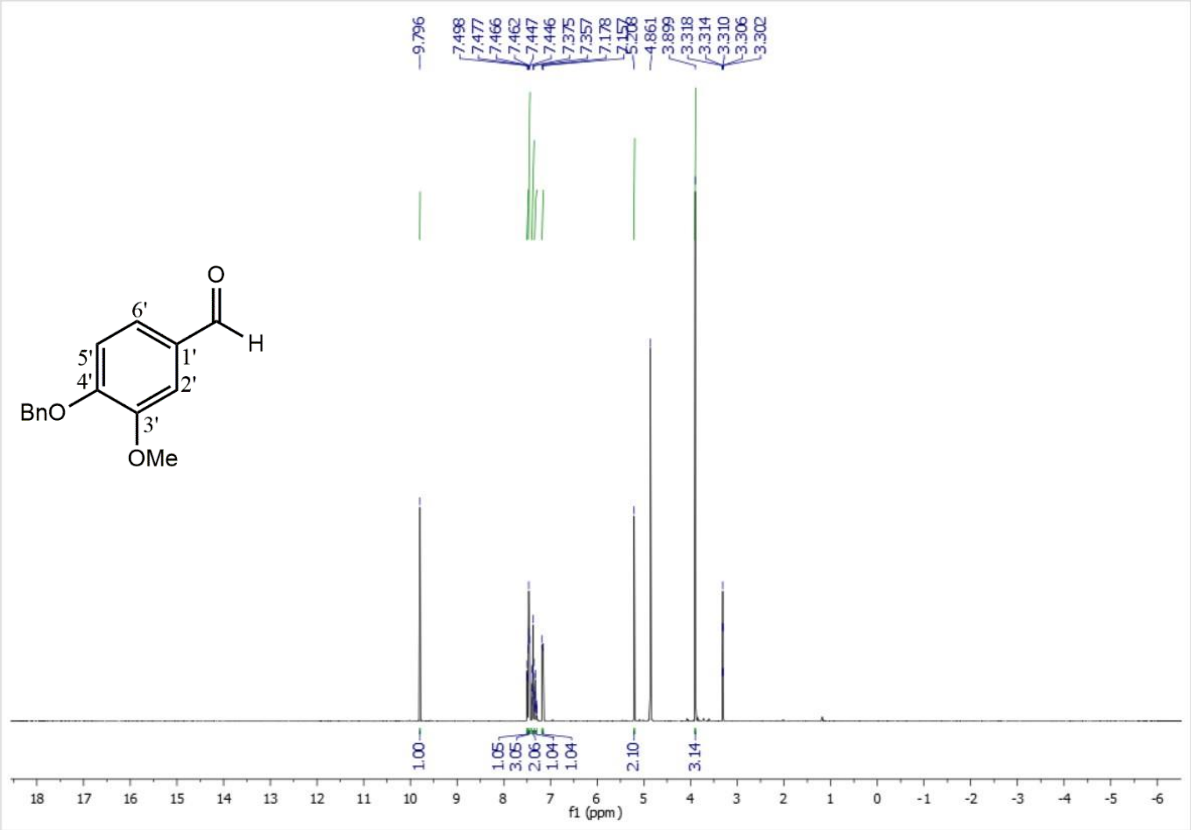


**Figure S1.** $^{1}$H NMR spectrum of benzylvanillin **(2)**

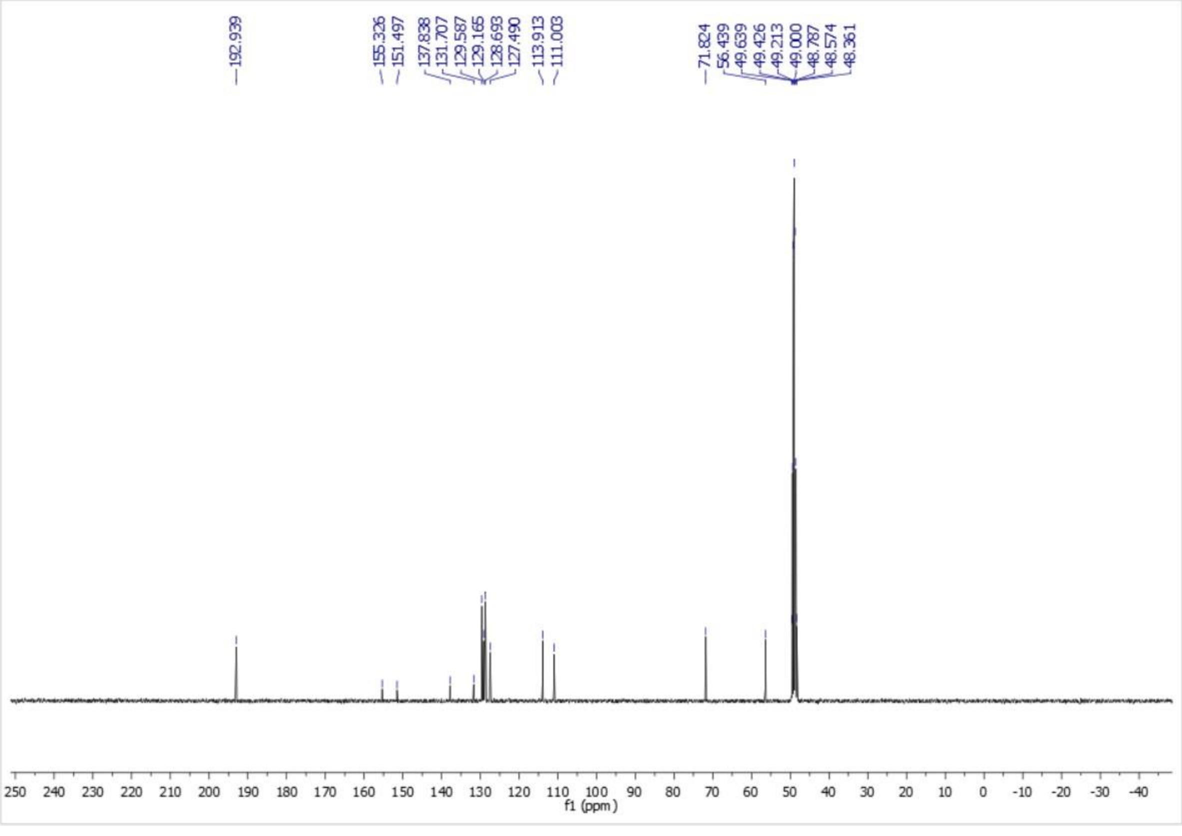

**Figure S2.** $^{13}C$ NMR spectrum of benzylvanillin **(2)**

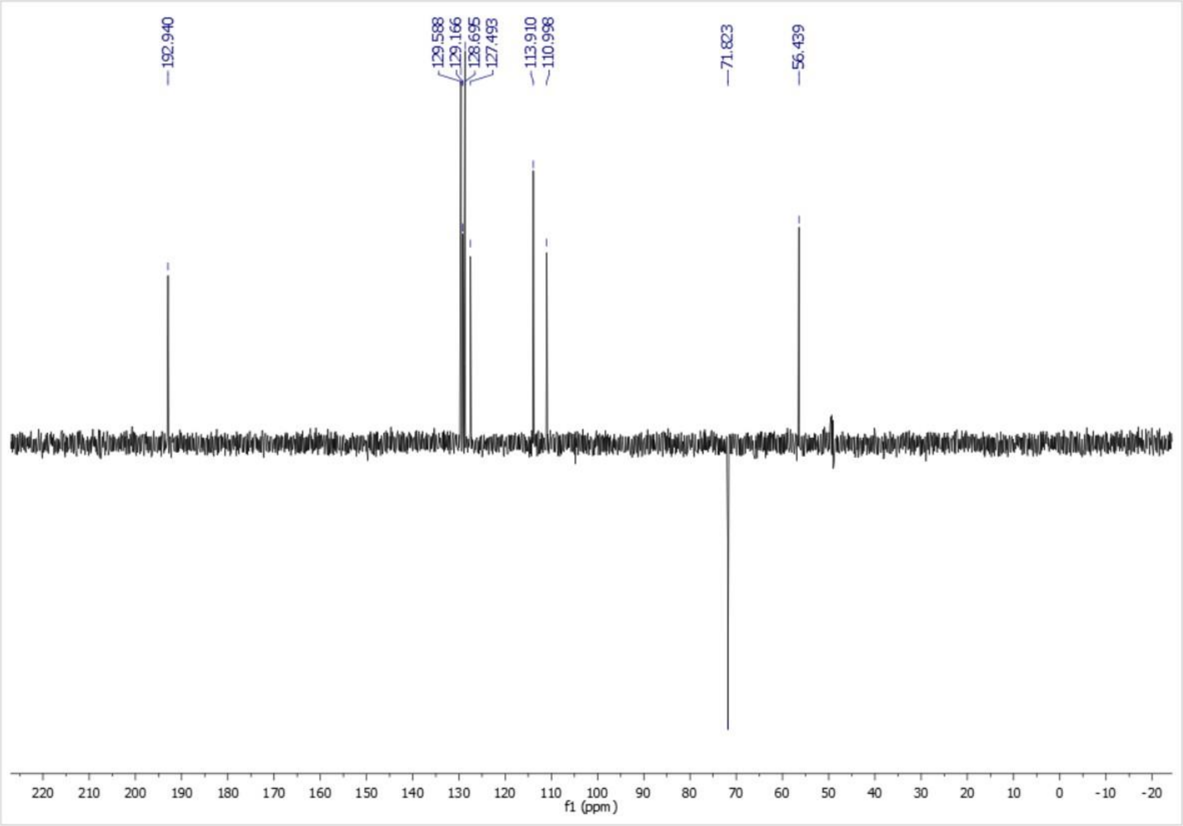


**Figure S3.** DEPT 135 NMR spectrum of benzylvanillin **(2)**

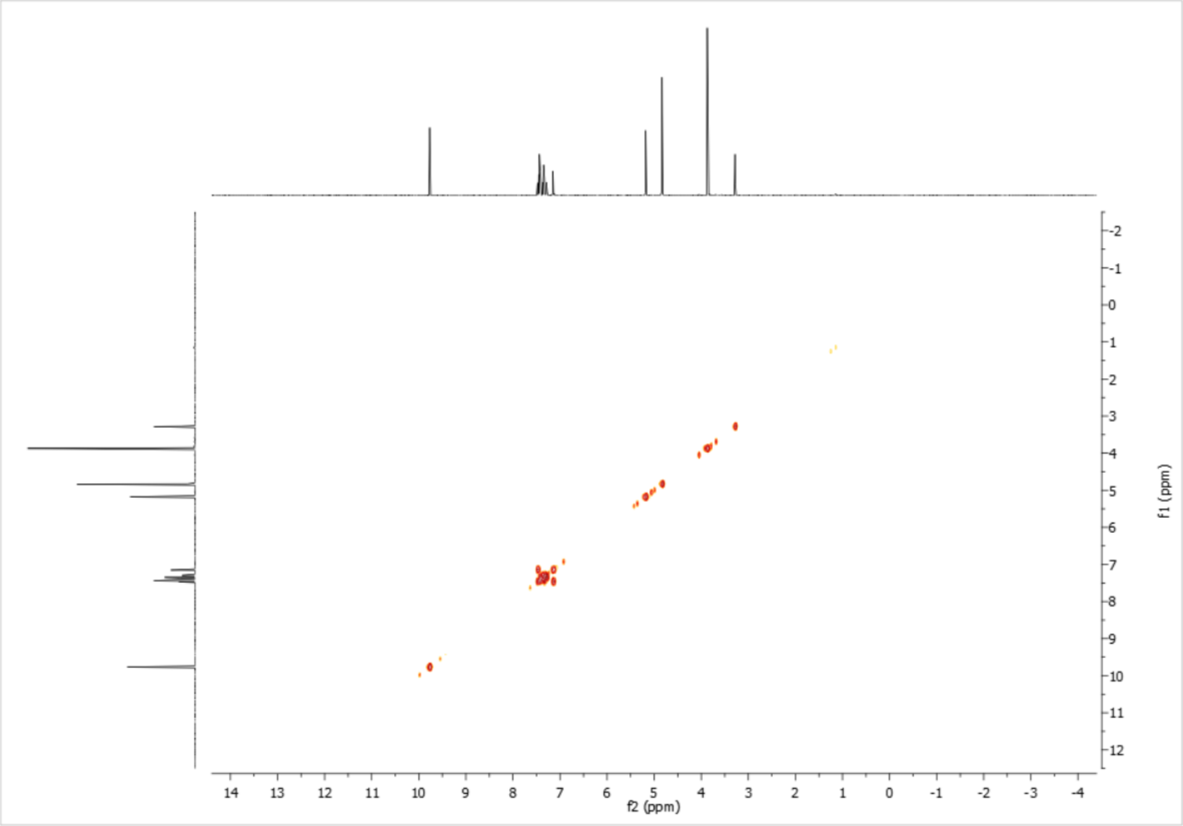


**Figure S4.** COSY spectrum of benzylvanillin **(2)**

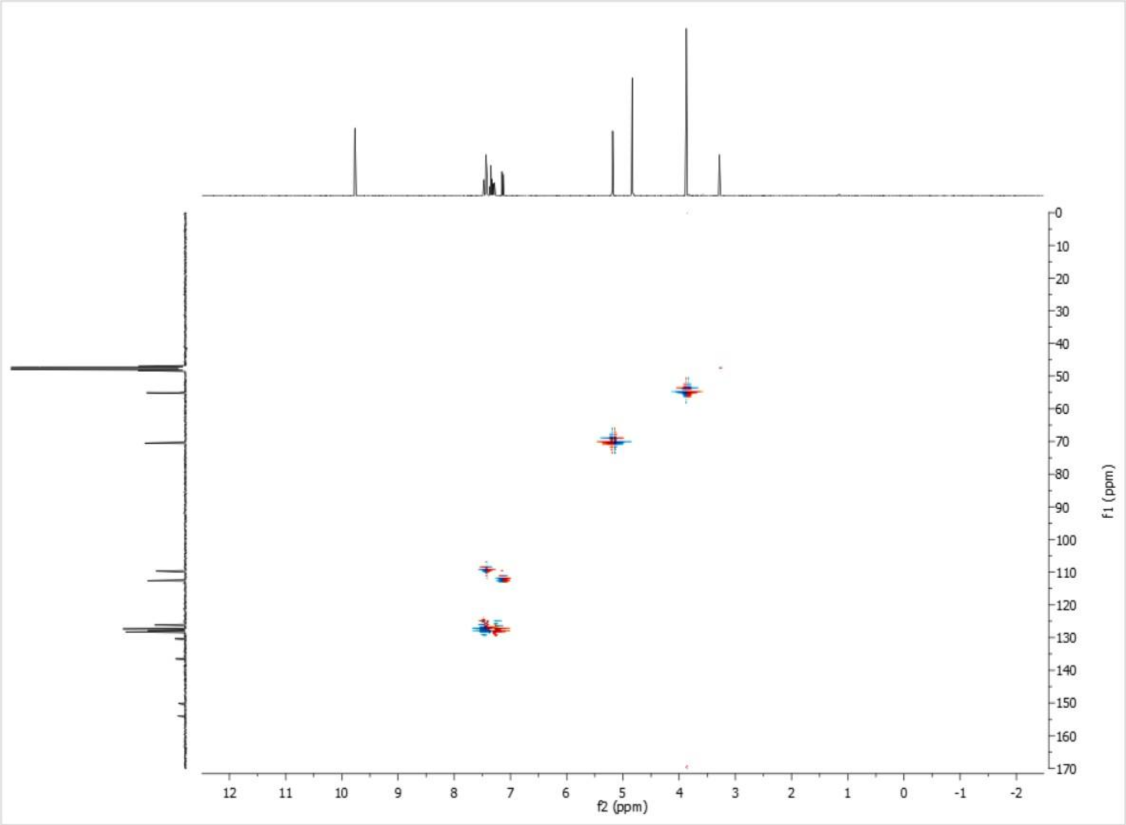


**Figure S5.** HMQC spectrum of benzylvanillin **(2)**

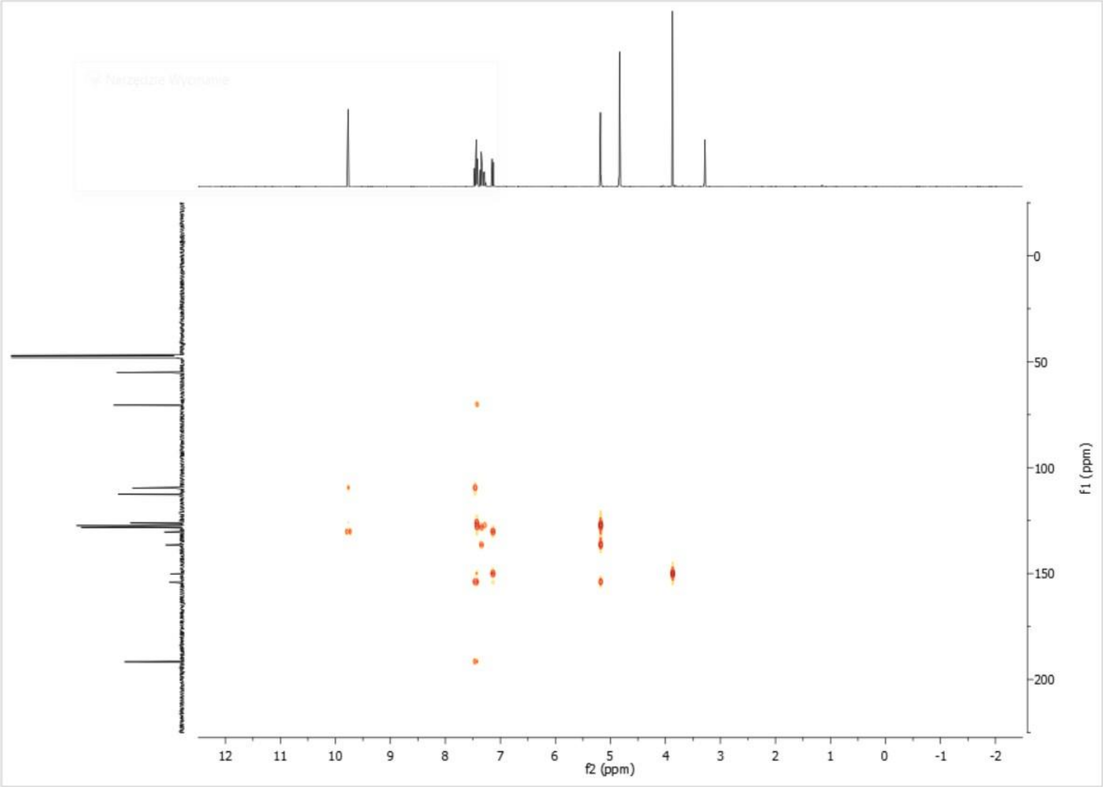


**Figure S6.** HMBC spectrum of benzylvanillin one **(2)**

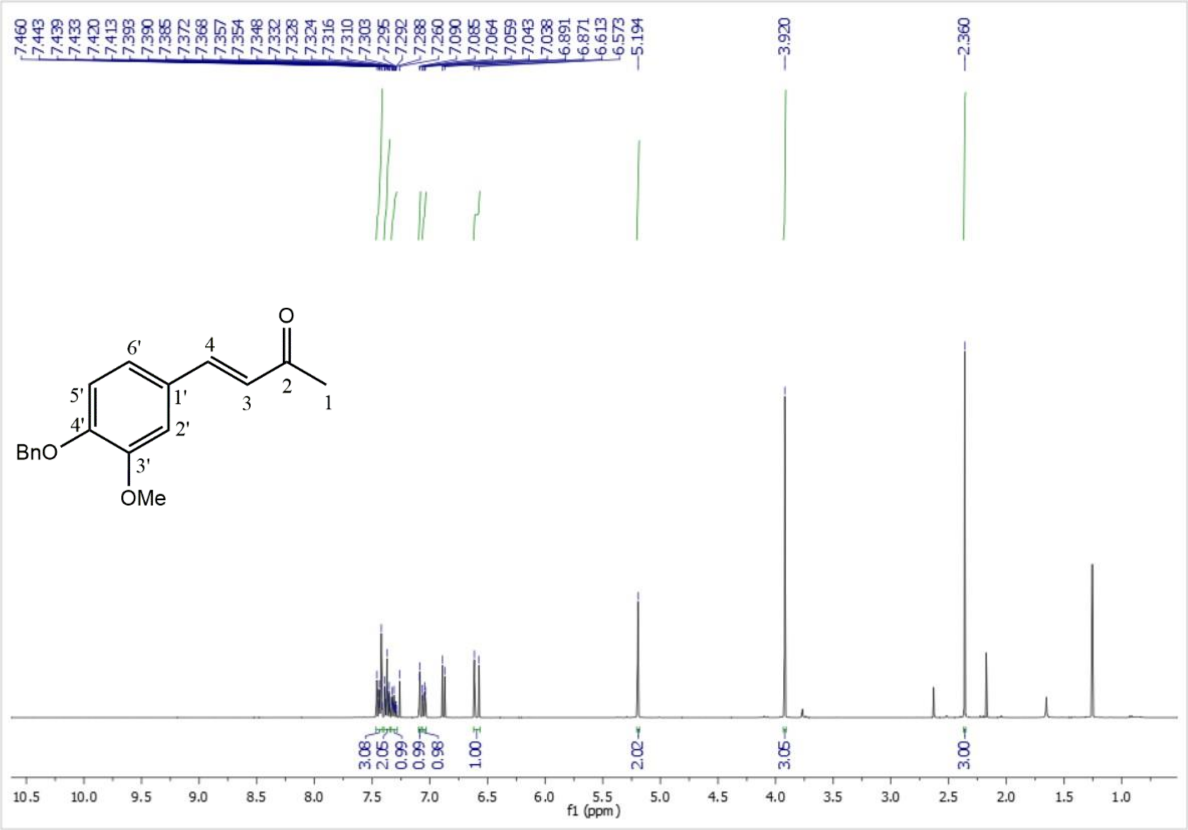

**Figure S7.** $^{1}$H NMR spectrum of (*E*)-4-(4'-(benzyloxy-3'-methoxyphenyl)but-3-en-2-one **(3)**

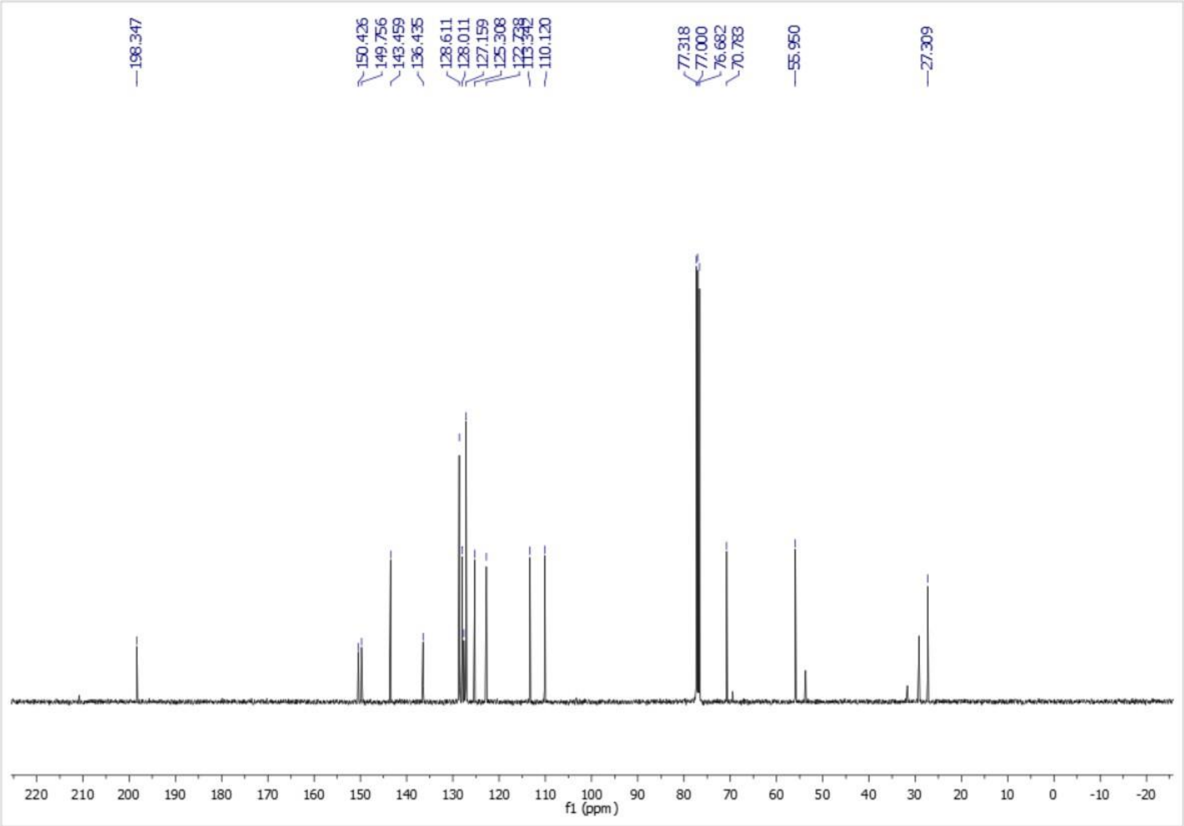

**Figure S8.** $^{13}$C NMR spectrum of (*E*)-4-(4'-(benzyloxy-3'-methoxyphenyl)but-3-en-2-one **(3)**

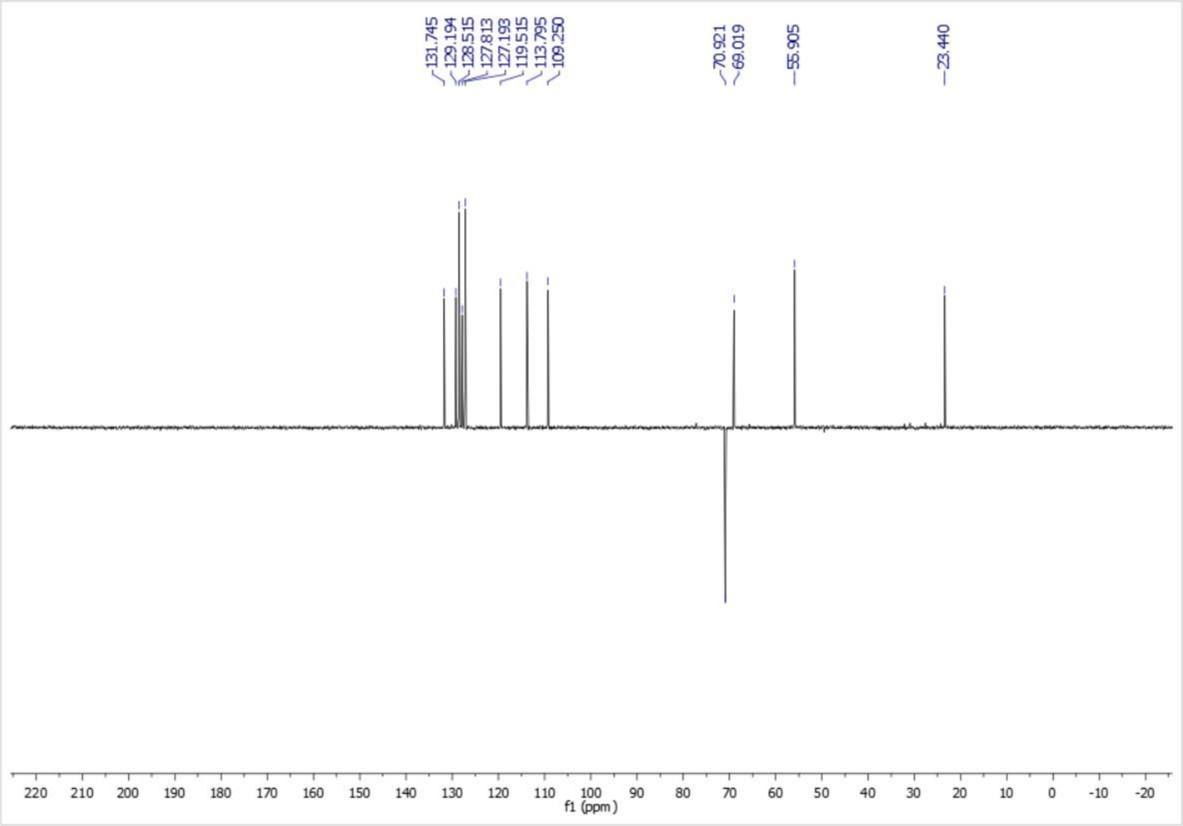


**Figure S9.** DEPT 135 NMR spectrum of (*E*)-4-(4'-(benzyloxy-3'-methoxyphenyl)but-3-en-2-one **(3)**

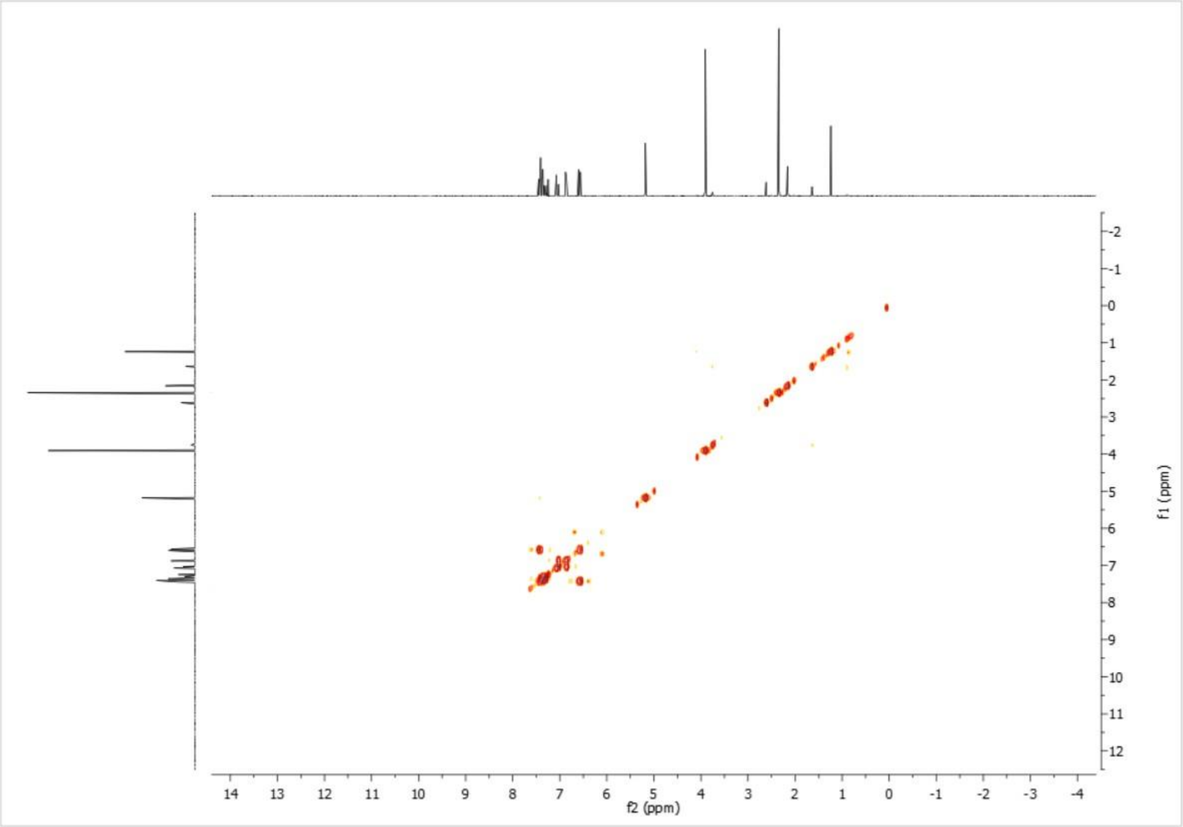


**Figure S10.** COSY spectrum of (*E*)-4-(4'-(benzyloxy-3'-methoxyphenyl)but-3-en-2-one **(3)**

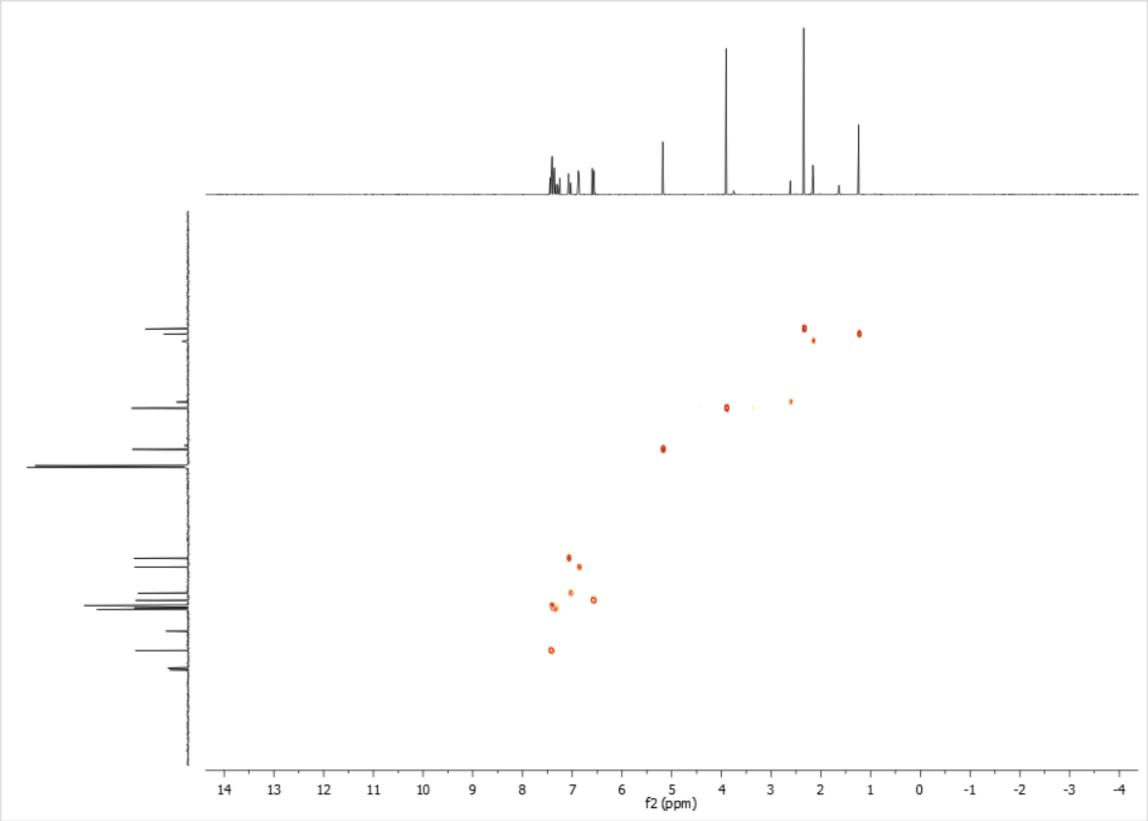


**Figure S11.** HMQC spectrum of (*E*)-4-(4'-(benzyloxy-3'-methoxyphenyl)but-3-en-2-one **(3)**

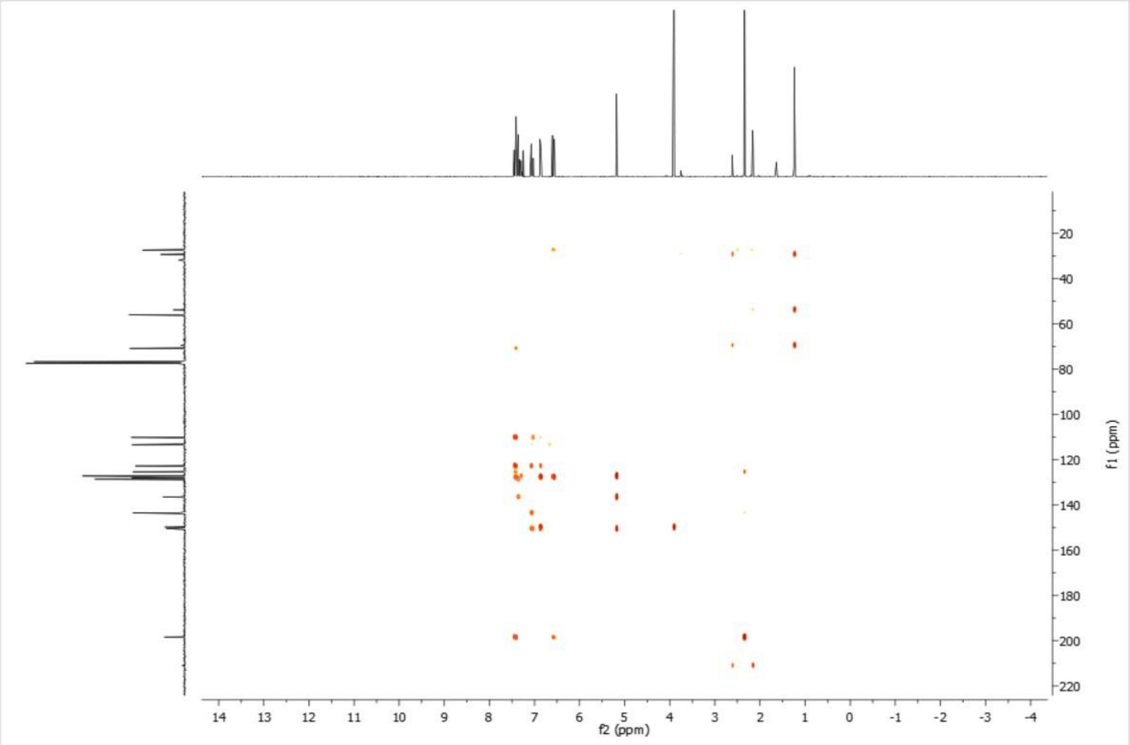


**Figure S12.** HMBC spectrum of (*E*)-4-(4'-(benzyloxy-3'-methoxyphenyl)but-3-en-2-one **(3)**

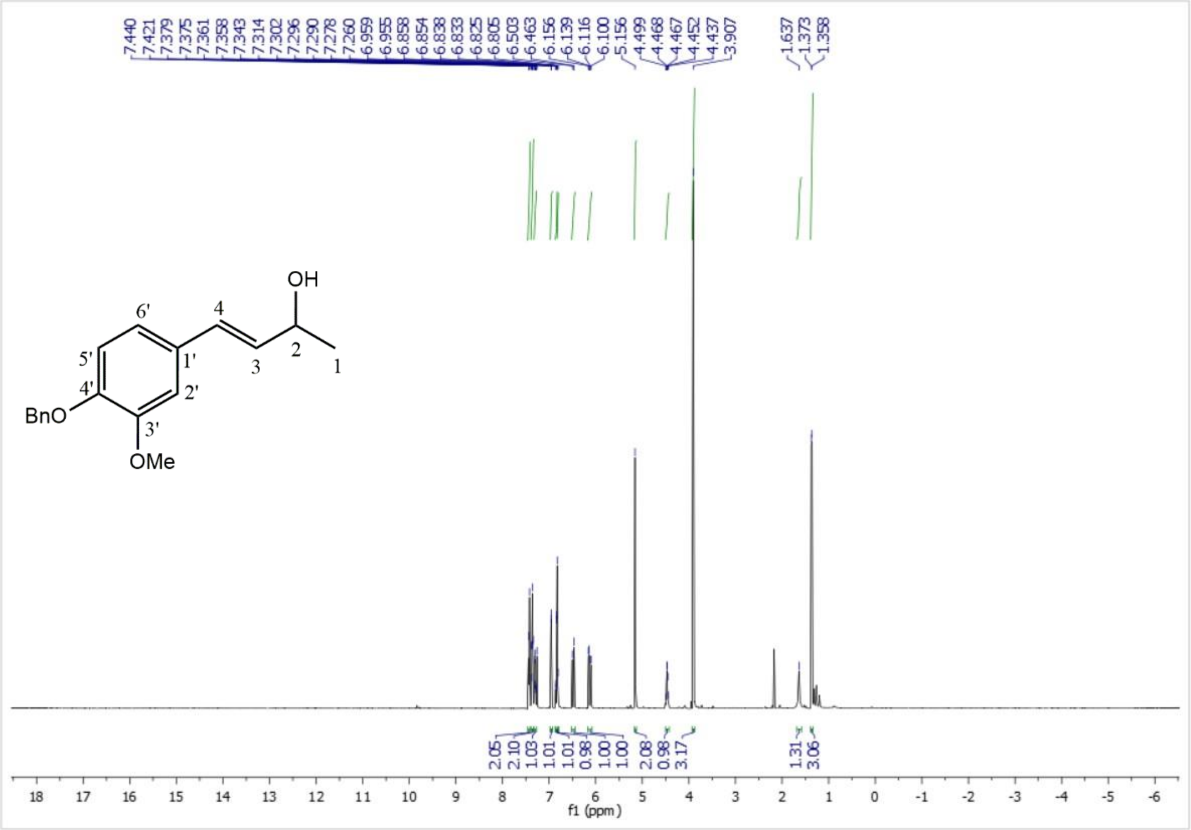

**Figure S13.** $^{1}$H NMR spectrum of ($E$)-4-(4'-benzyloxy-3'-methoxyphenyl)but-3-en-2-ol **(4)**

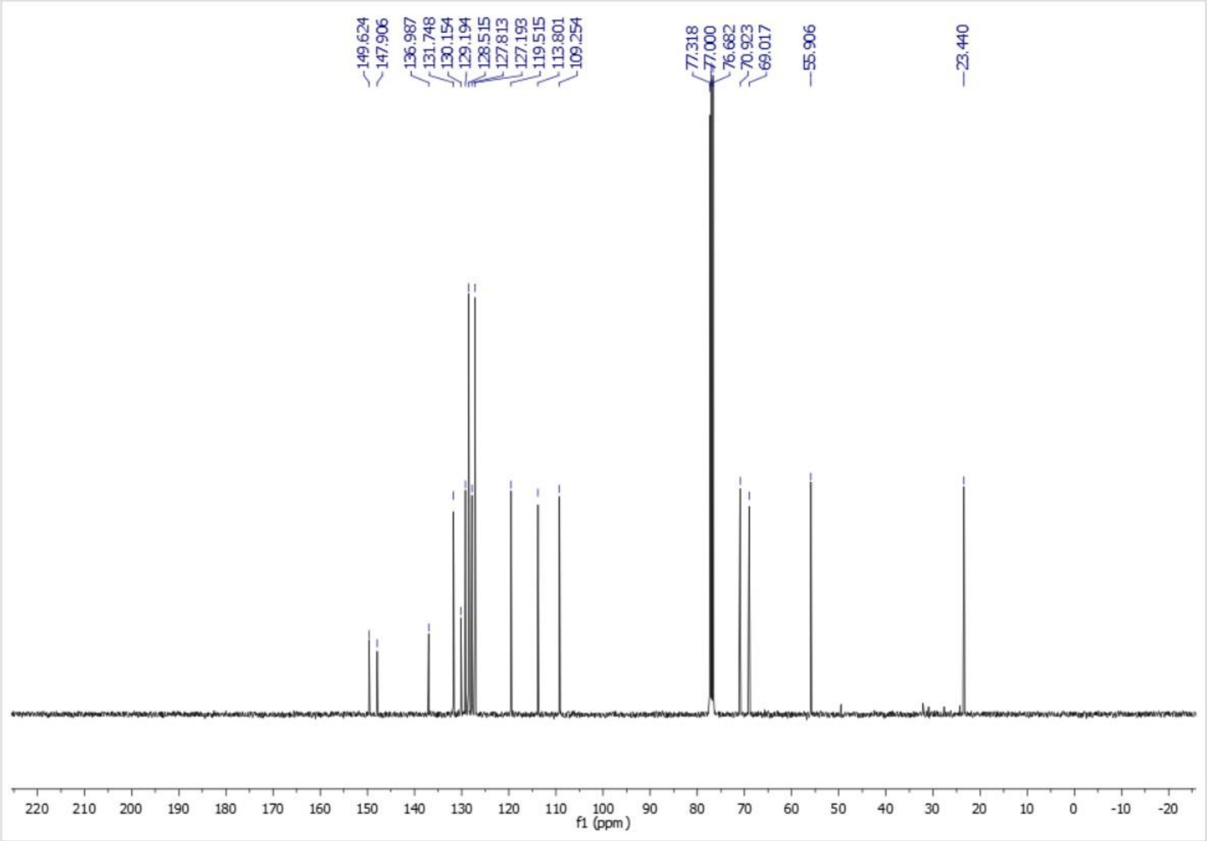

**Figure S14.** $^{13}$C NMR spectrum of ($E$)-4-(4'-benzyloxy-3'-methoxyphenyl)but-3-en-2-ol **(4)**

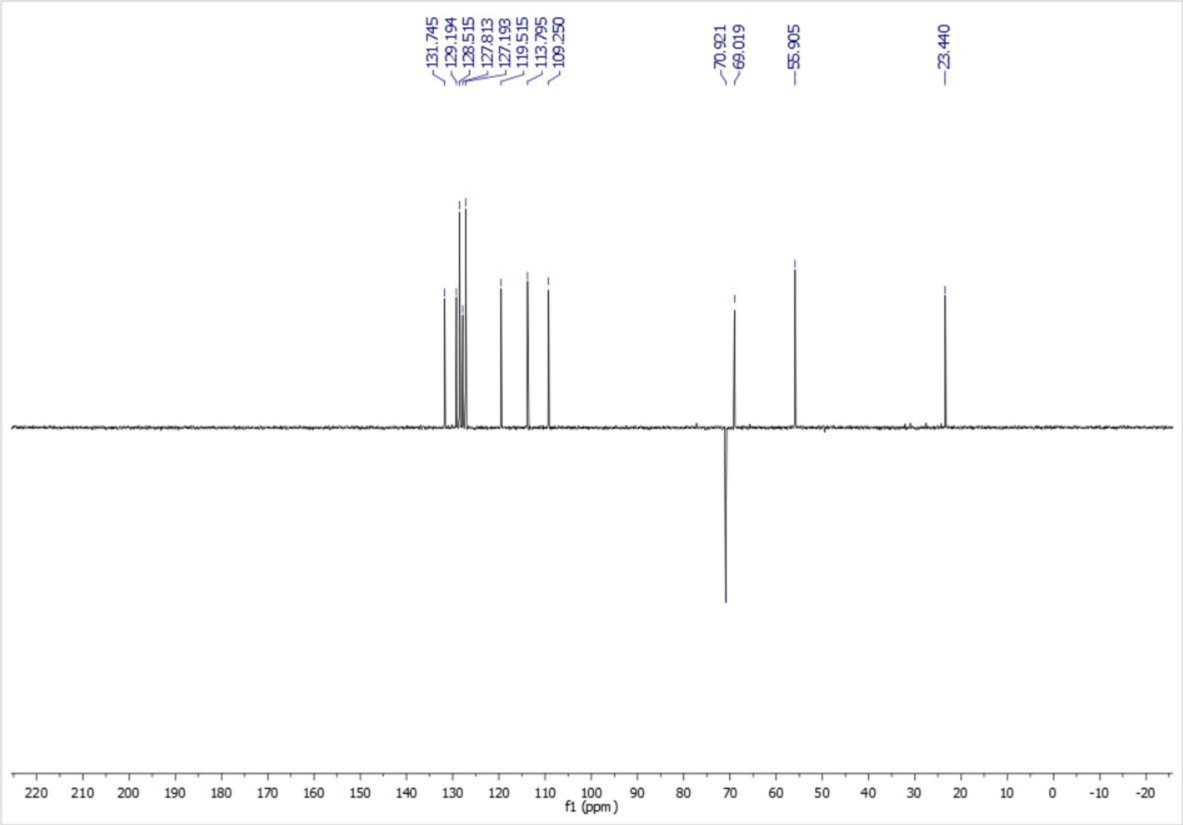


**Figure S15.** DEPT 135 NMR spectrum of (*E*)-4-(4'-benzyloxy-3'-methoxyphenyl)but-3-en-2-ol **(4)**

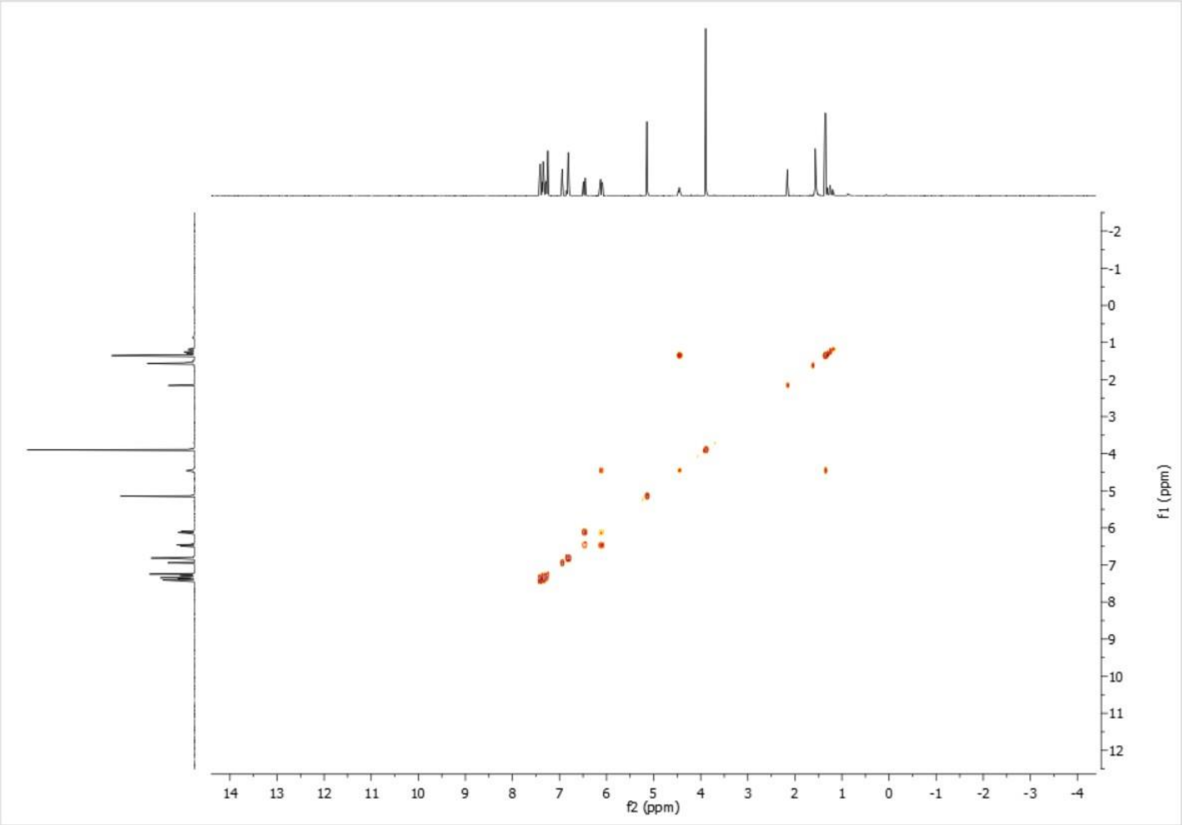


**Figure S16.** COSY spectrum of (*E*)-4-(4'-benzyloxy-3'-methoxyphenyl)but-3-en-2-ol **(4)**

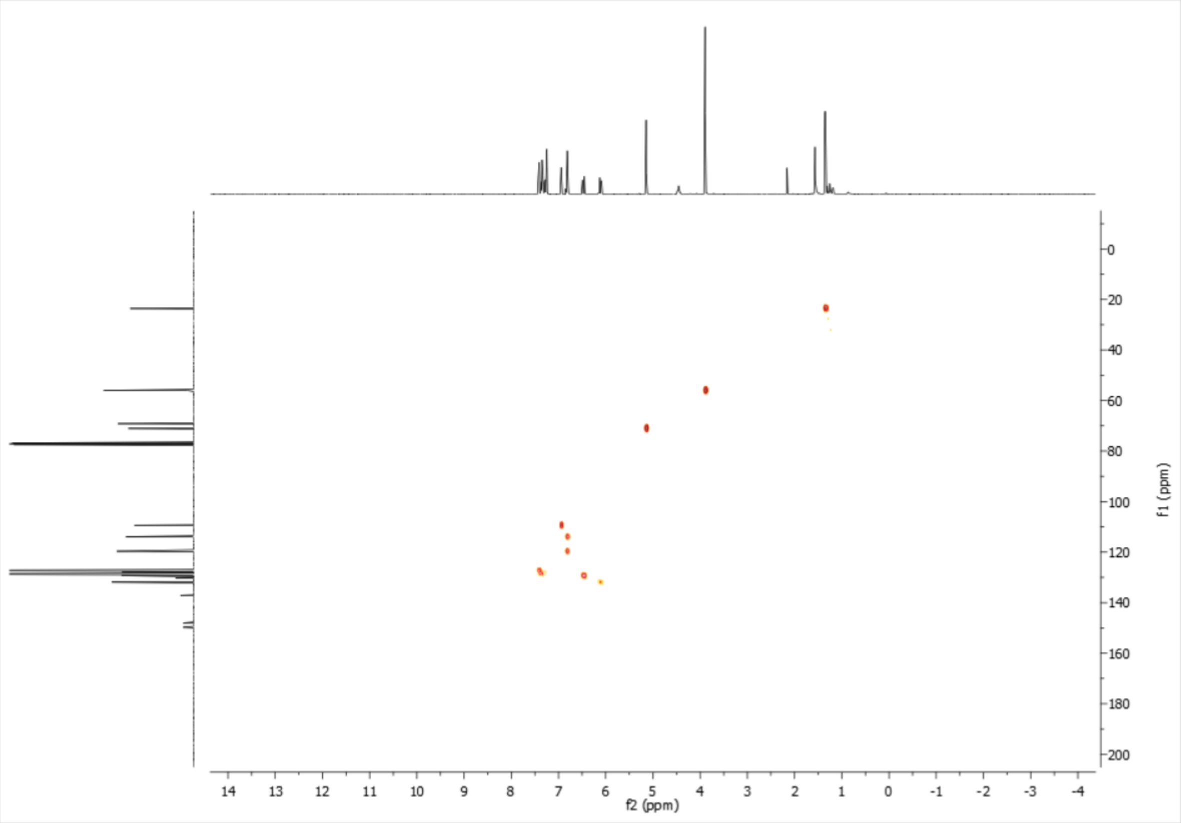


**Figure S17.** HMQC spectrum of (*E*)-4-(4'-benzyloxy-3'-methoxyphenyl)but-3-en-2-ol **(4)**

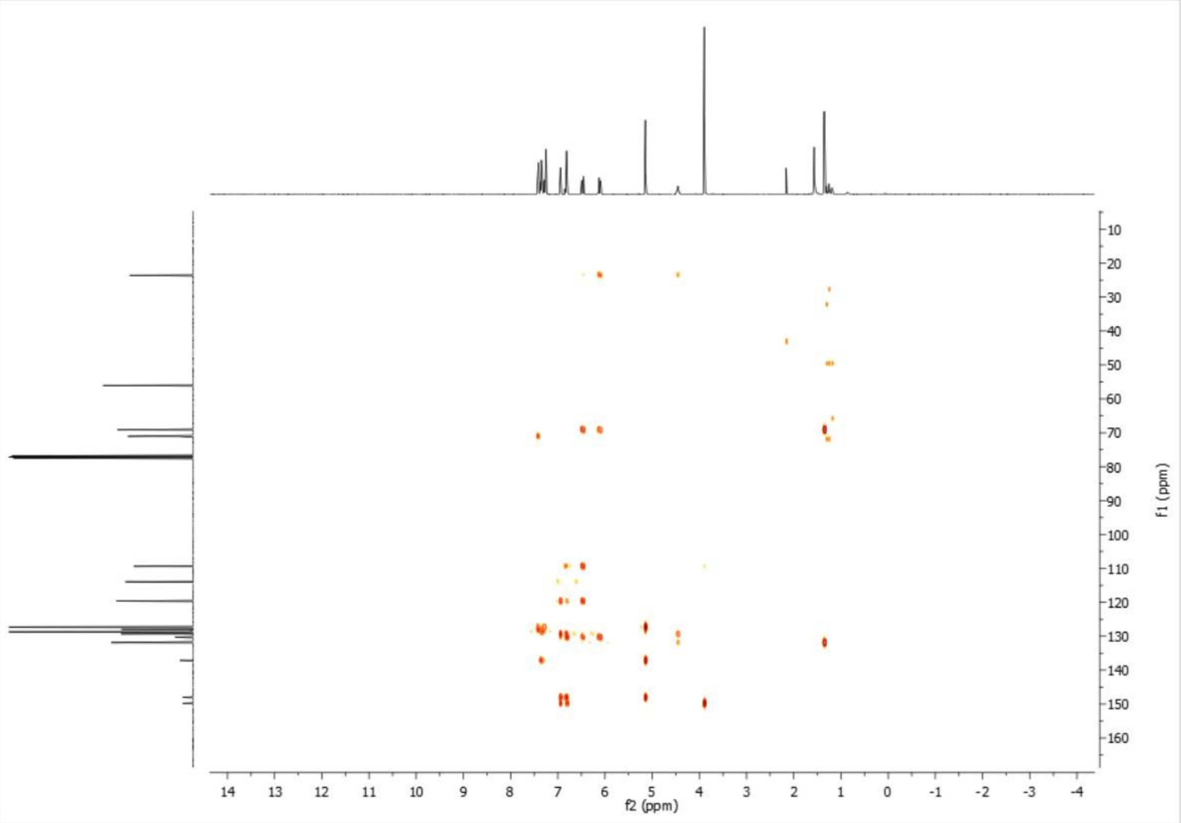


**Figure S18.** HMBC spectrum of (*E*)-4-(4'-benzyloxy-3'-methoxyphenyl)but-3-en-2-ol **(4)**

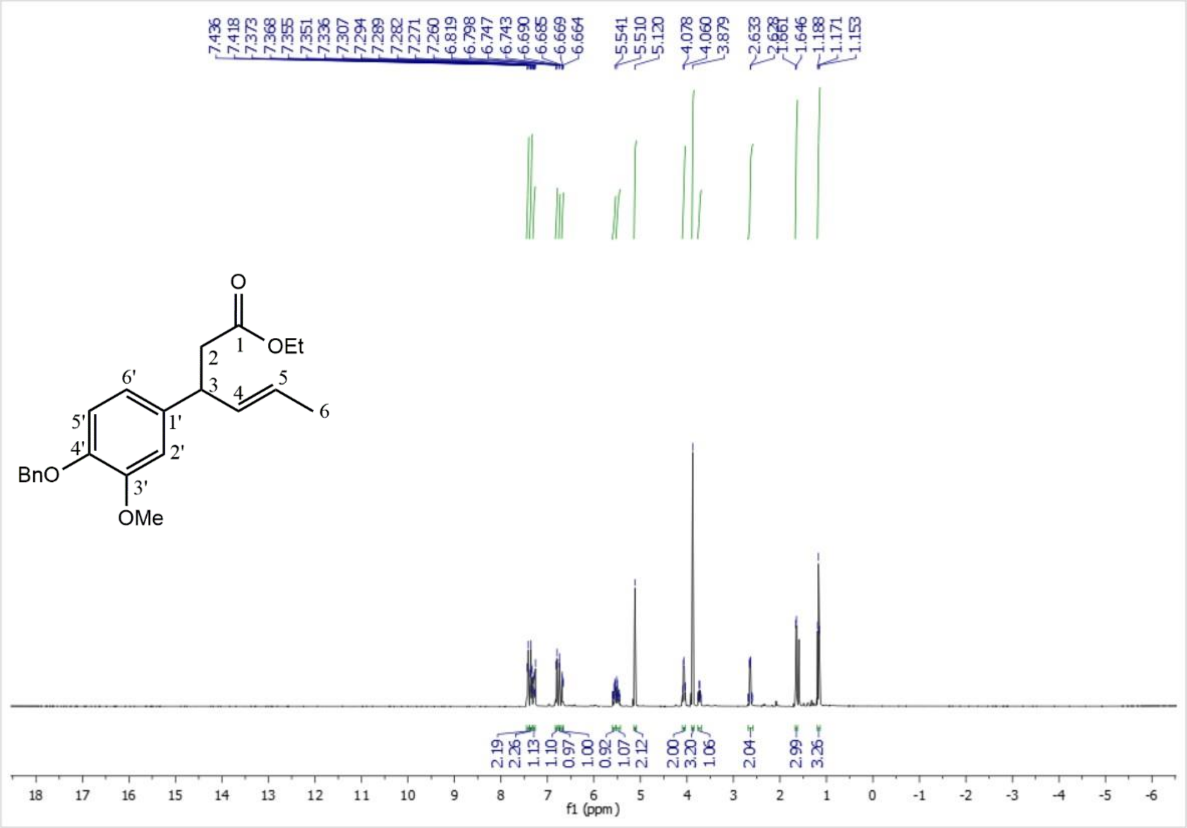

**Figure S19.** $^{1}$H NMR spectrum of (*E*)-3-(4'-benzyloxy-3'-methoxyphenyl)hex-4-enoate **(5)**

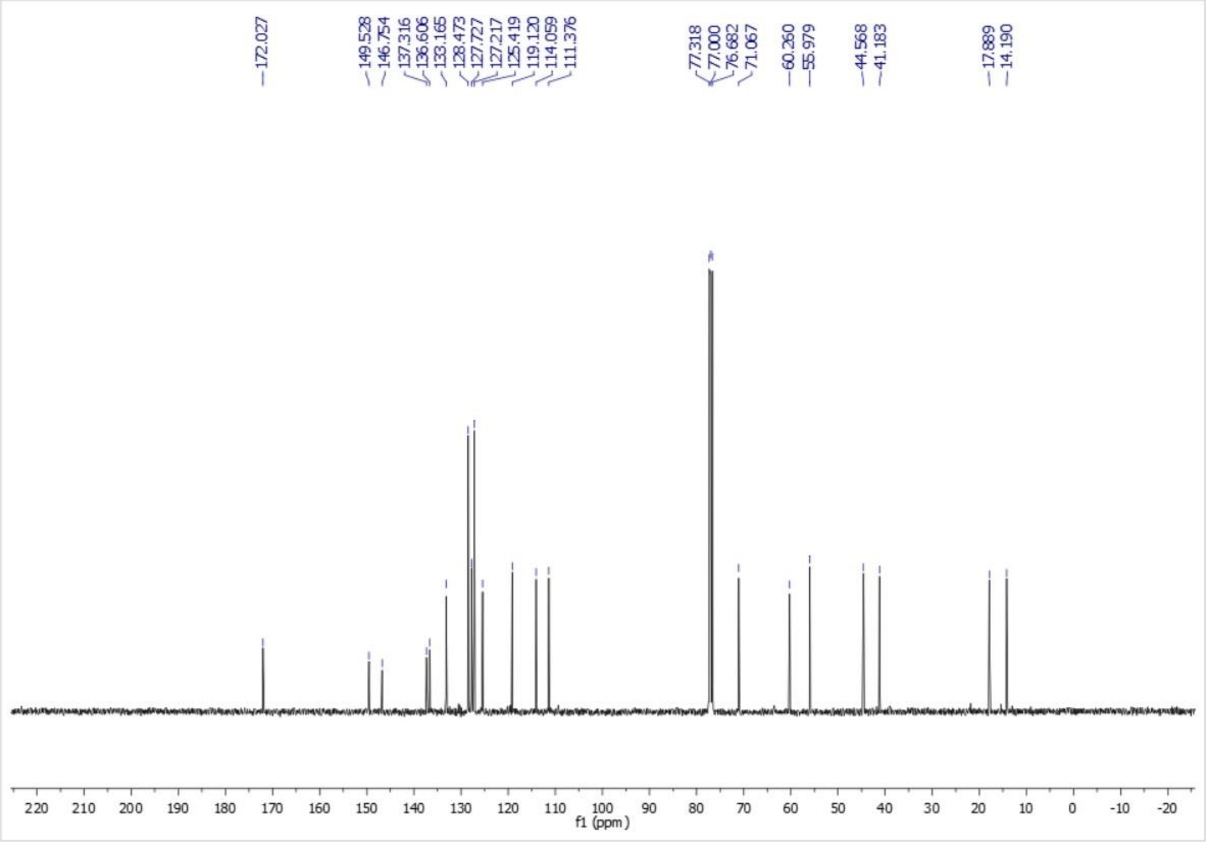

**Figure S20.** $^{13}$C NMR spectrum of (*E*)-3-(4'-benzyloxy-3'-methoxyphenyl)hex-4-enoate **(5)**

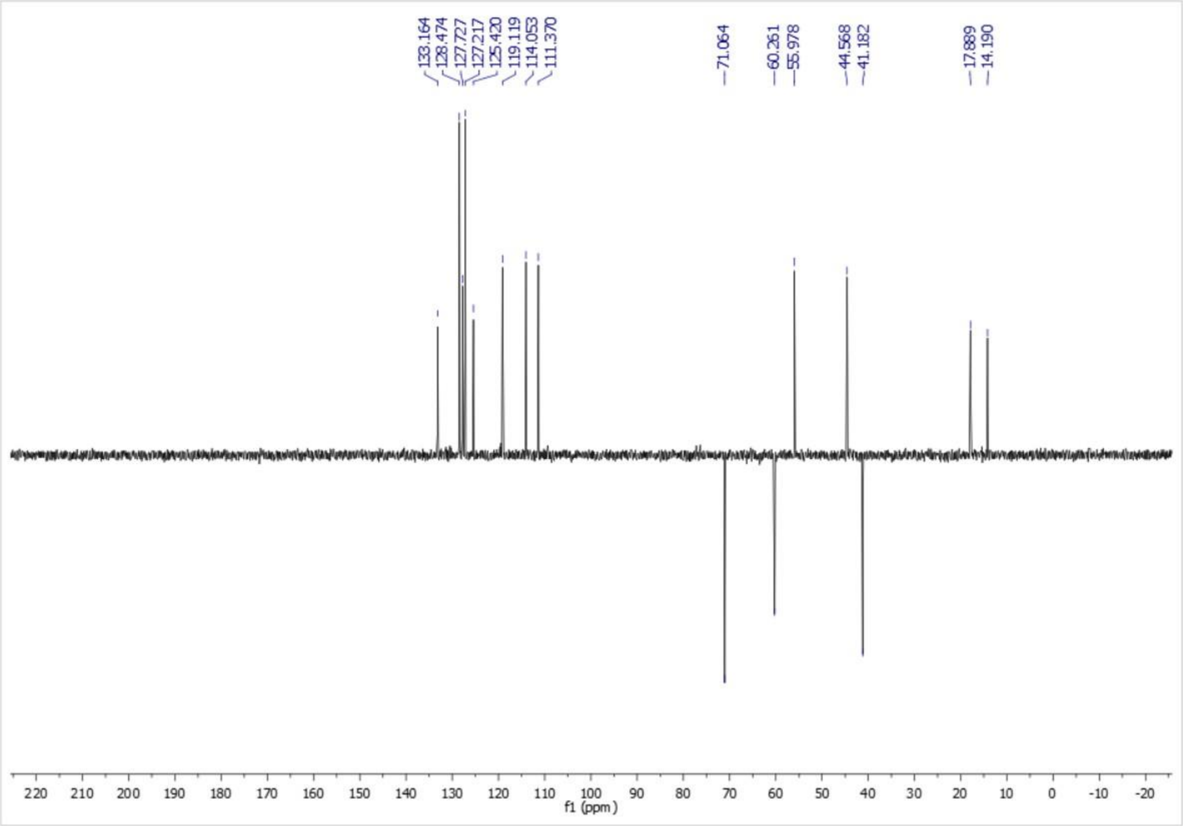


**Figure S21.** DEPT 135 NMR spectrum of (*E*)-3-(4'-benzyloxy-3'-methoxyphenyl)hex-4-enoate **(5)**

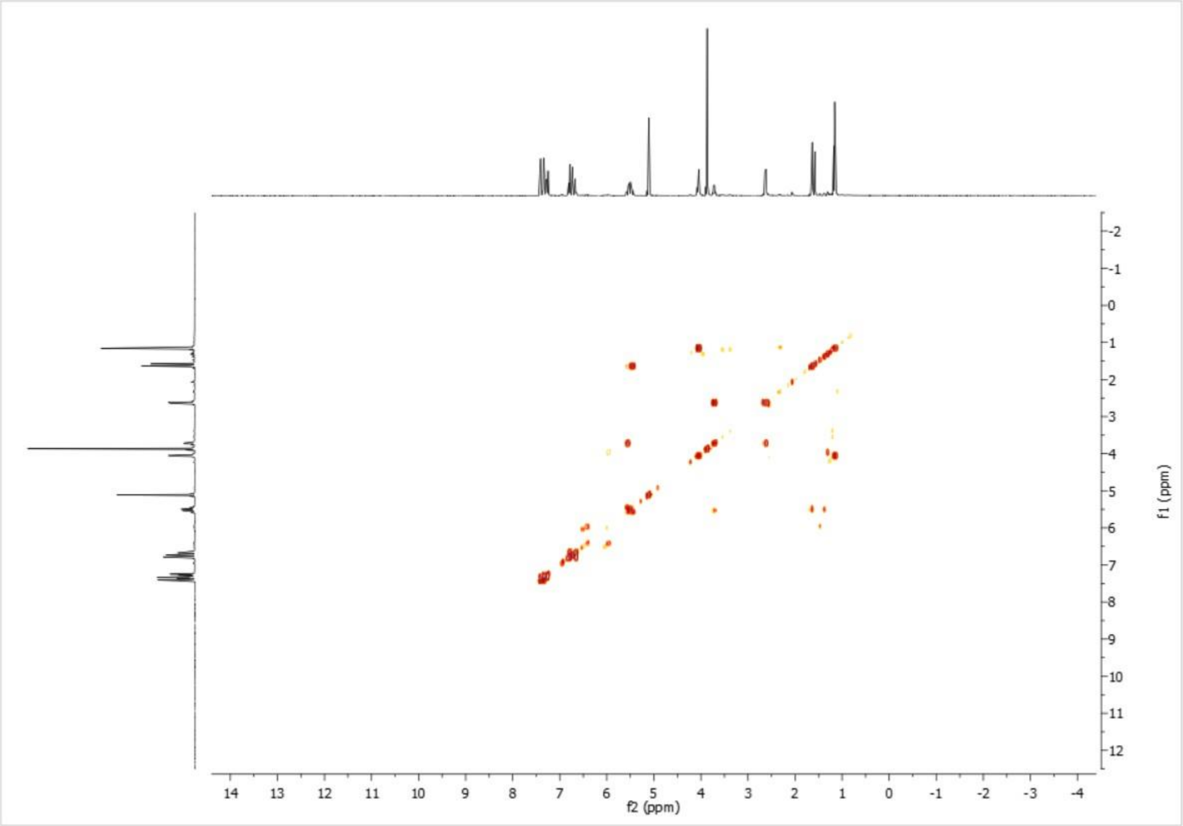


**Figure S22.** COSY spectrum of (*E*)-3-(4'-benzyloxy-3'-methoxyphenyl)hex-4-enoate **(5)**

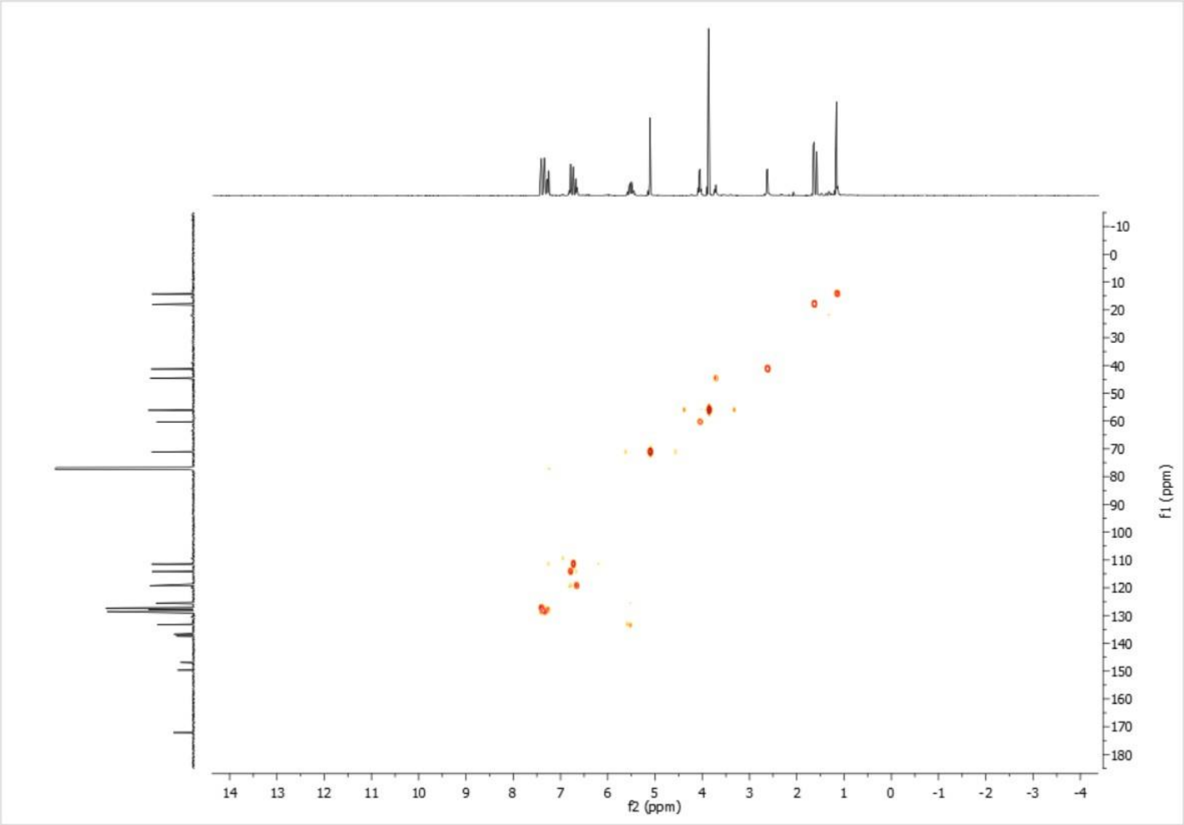


**Figure S23.** HMQC spectrum of (*E*)-3-(4'-benzyloxy-3'-methoxyphenyl)hex-4-enoate **(5)**

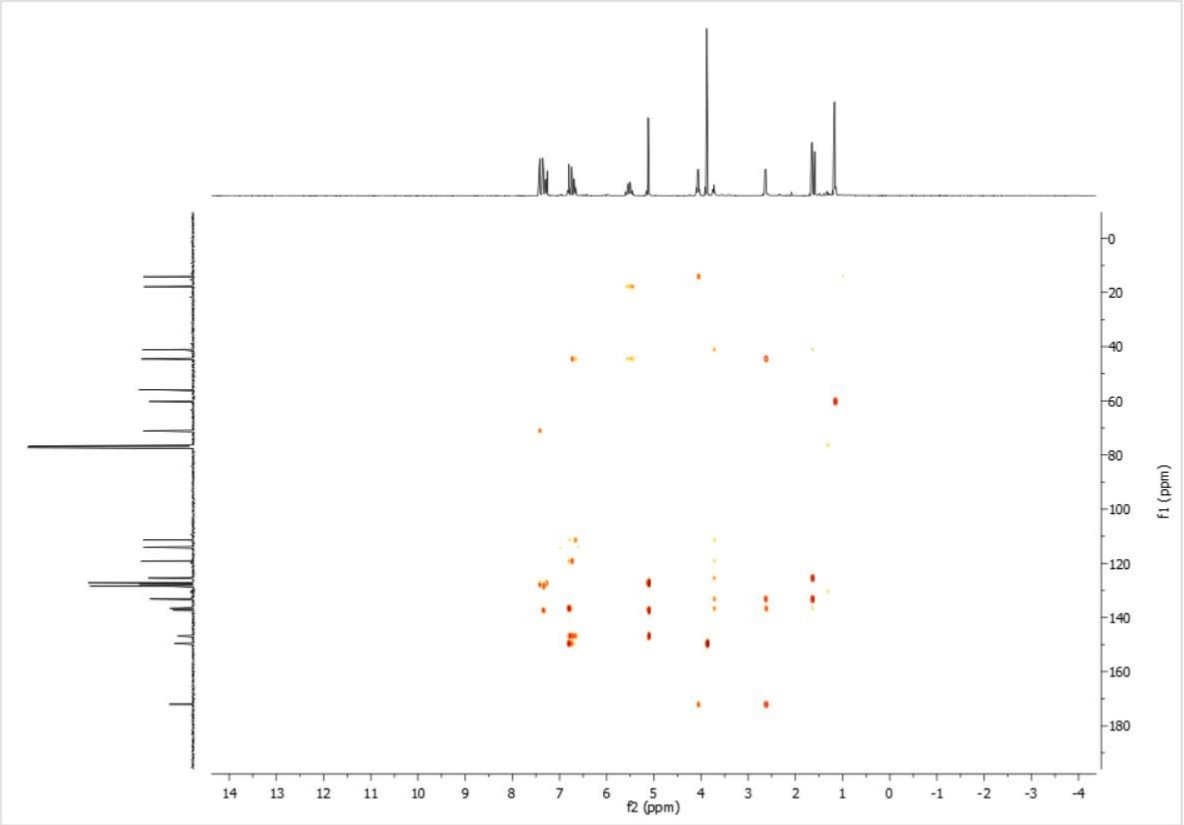


**Figure S24.** HMBC spectrum of (*E*)-3-(4'-benzyloxy-3'-methoxyphenyl)hex-4-enoate **(5)**

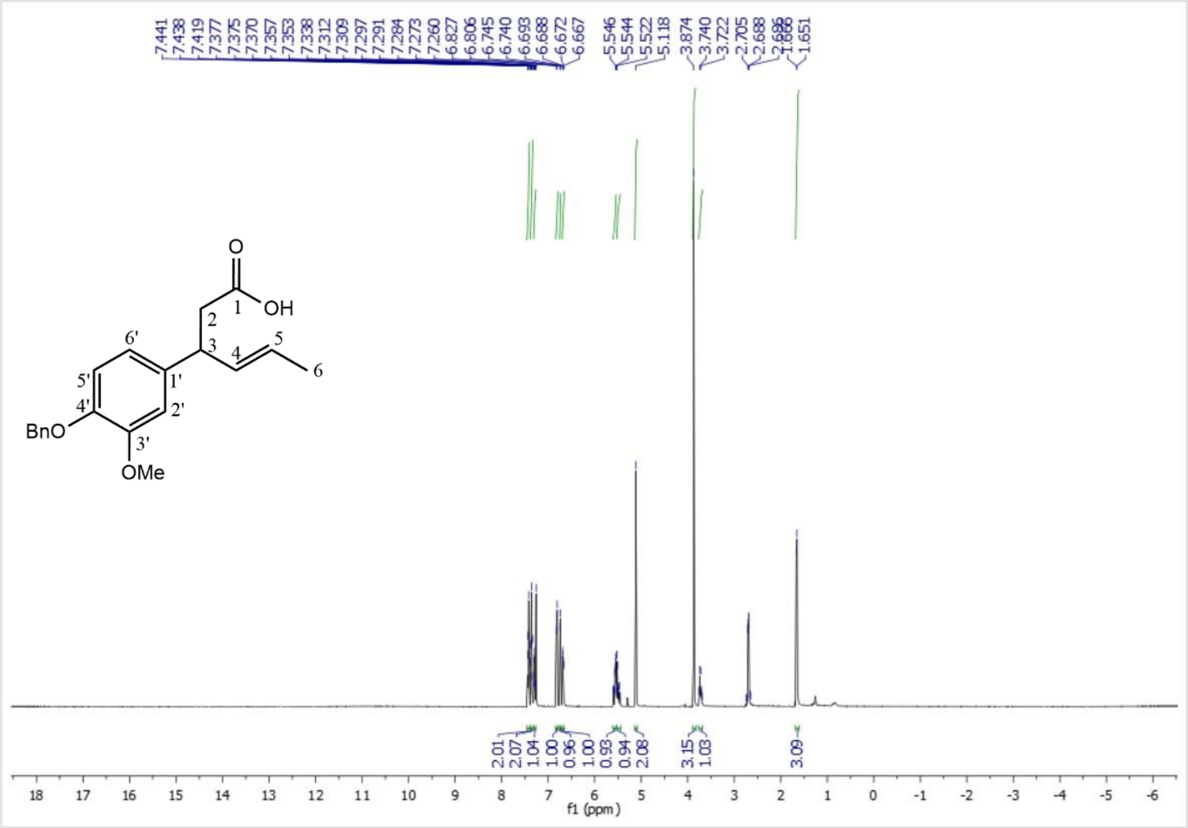

**Figure S25.** $^{1}$H NMR spectrum of (*E*)-3-(4'-benzyloxy-3'-methoxyphenyl)hex-4-enoic acid **(6)**

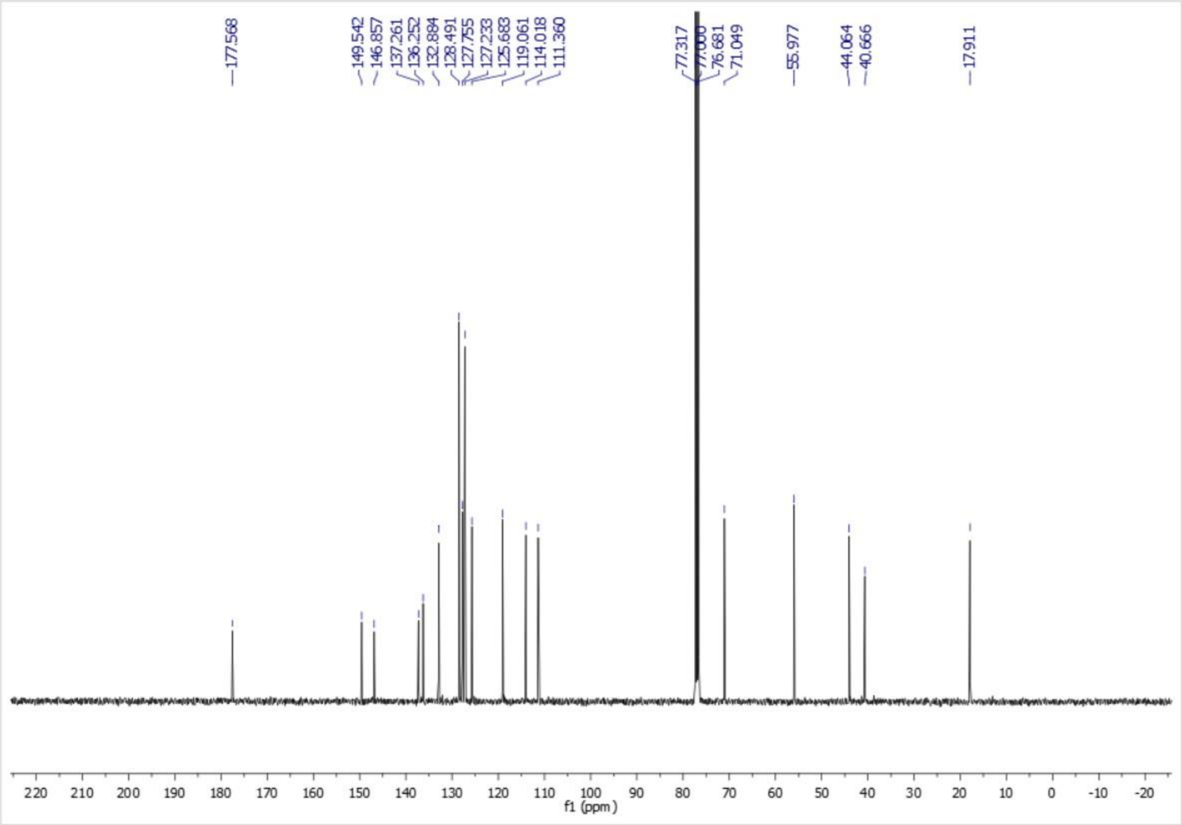

**Figure S26.** $^{13}$C NMR spectrum of (*E*)-3-(4'-benzyloxy-3'-methoxyphenyl)hex-4-enoic acid **(6)**

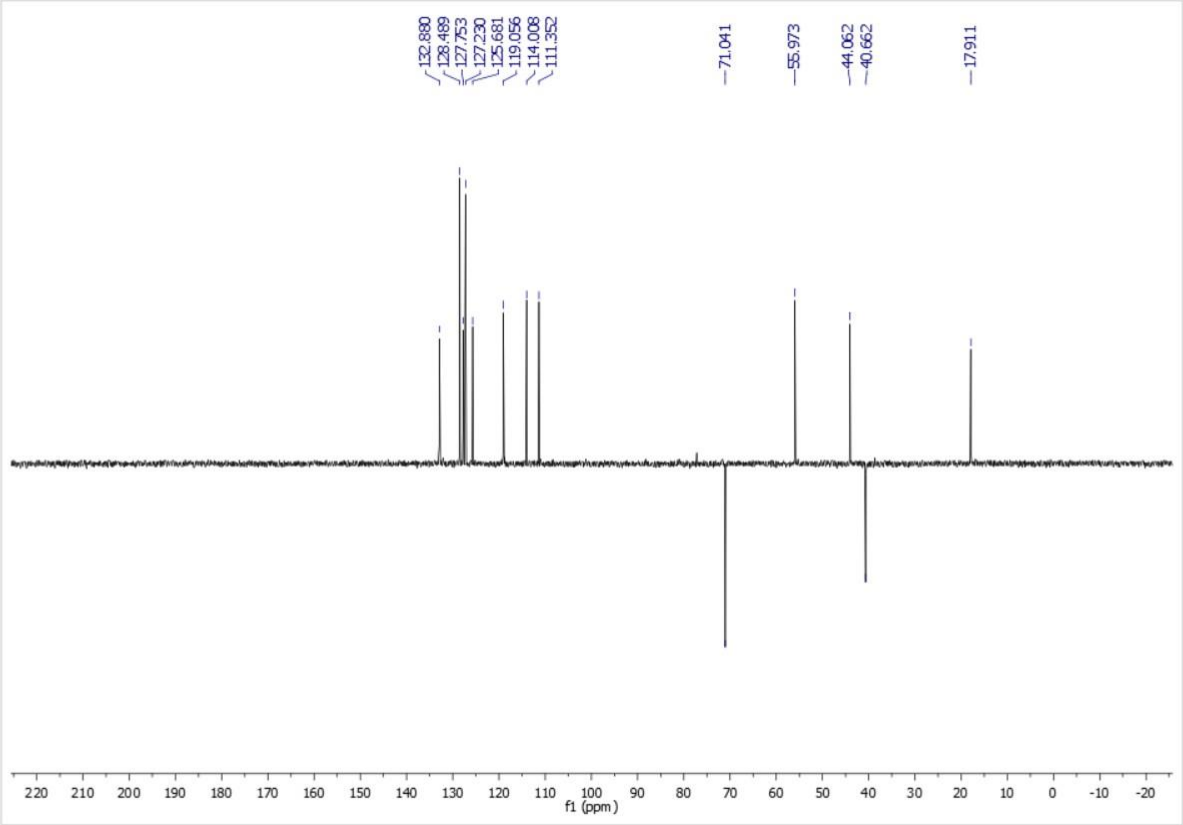


**Figure S27.** DEPT 135 NMR spectrum of (*E*)-3-(4'-benzyloxy-3'-methoxyphenyl)hex-4-enoic acid **(6)**

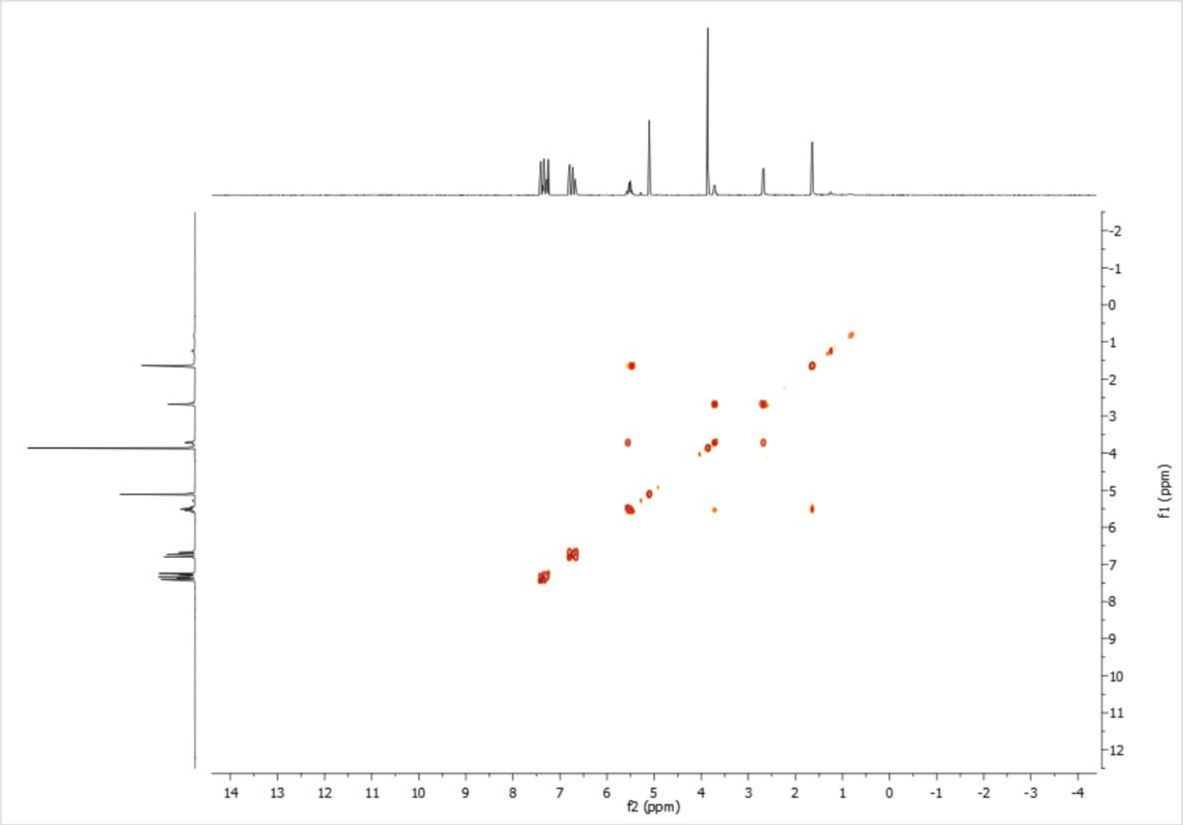


**Figure S28.** COSY spectrum of (*E*)-3-(4'-benzyloxy-3'-methoxyphenyl)hex-4-enoic acid **(6)**

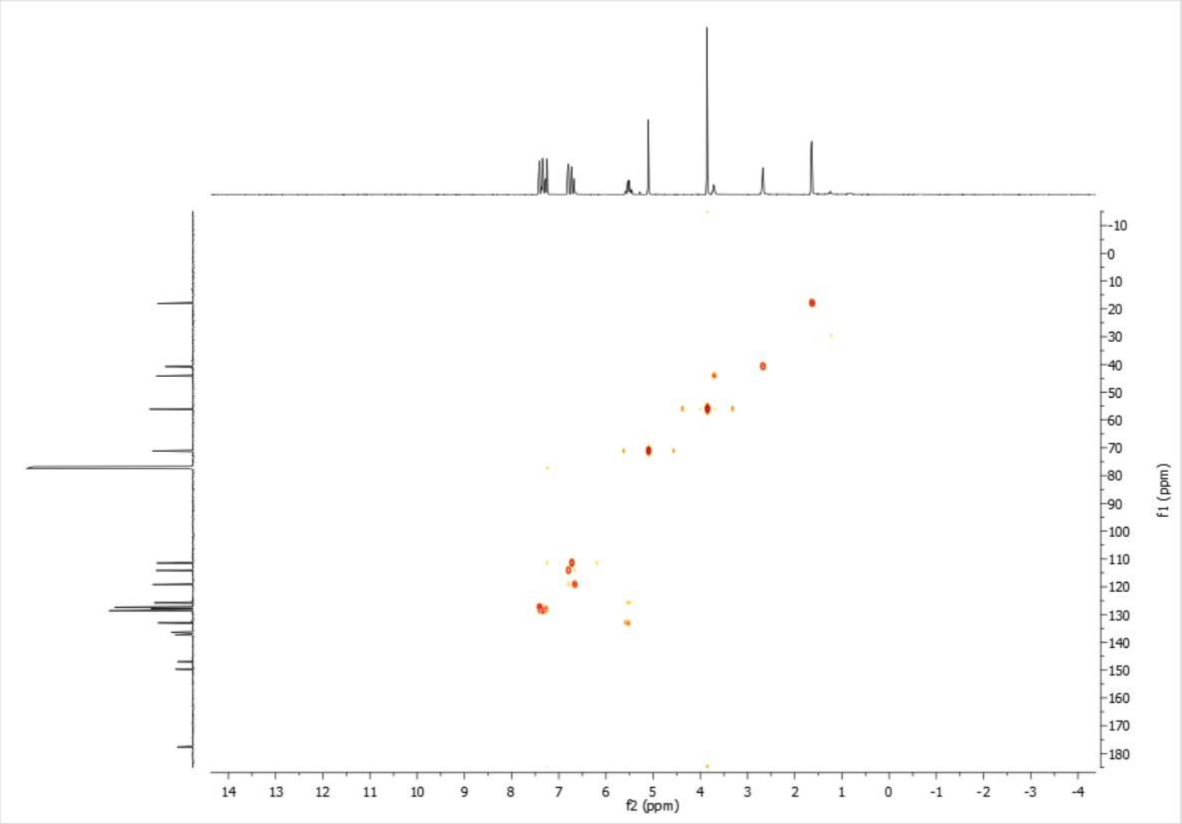


**Figure S29.** HMQC spectrum of (*E*)-3-(4'-benzyloxy-3'-methoxyphenyl)hex-4-enoic acid **(6)**

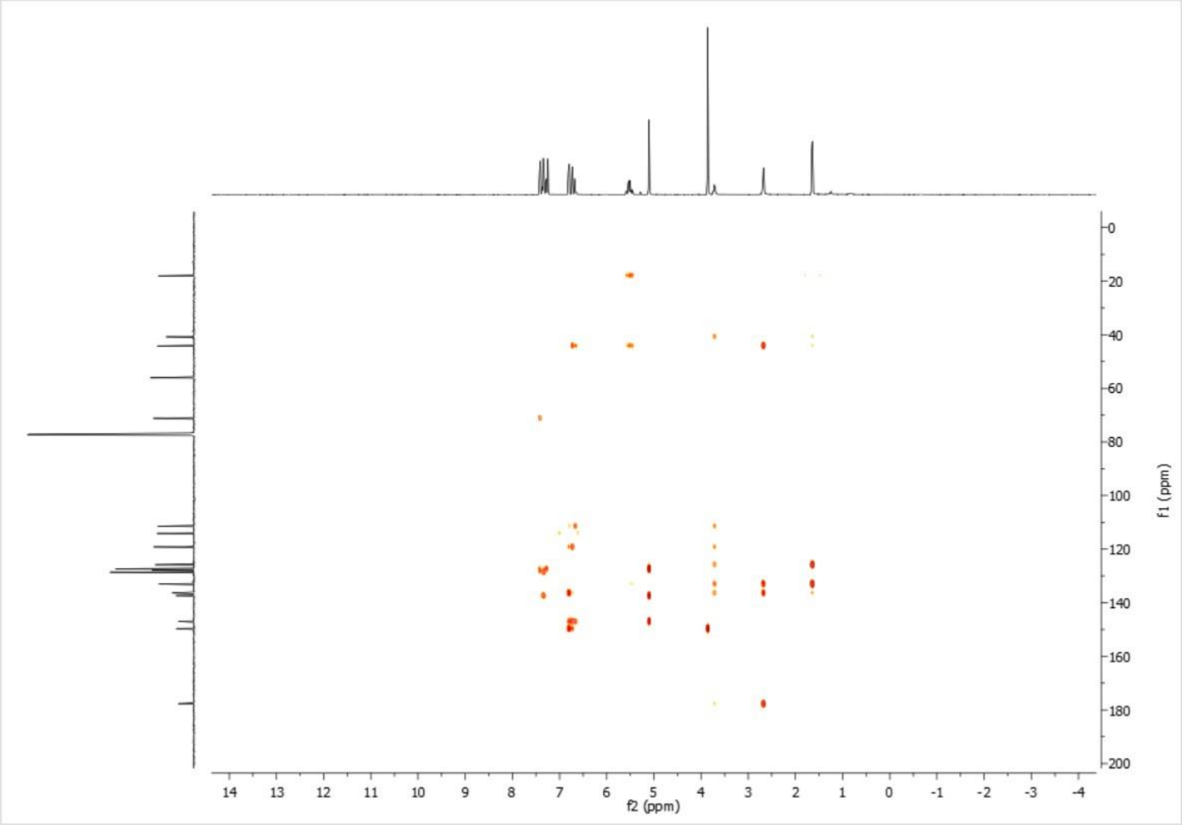


**Figure S30.** HMBC spectrum of (*E*)-3-(4'-benzyloxy-3'-methoxyphenyl)hex-4-enoic acid **(6)**

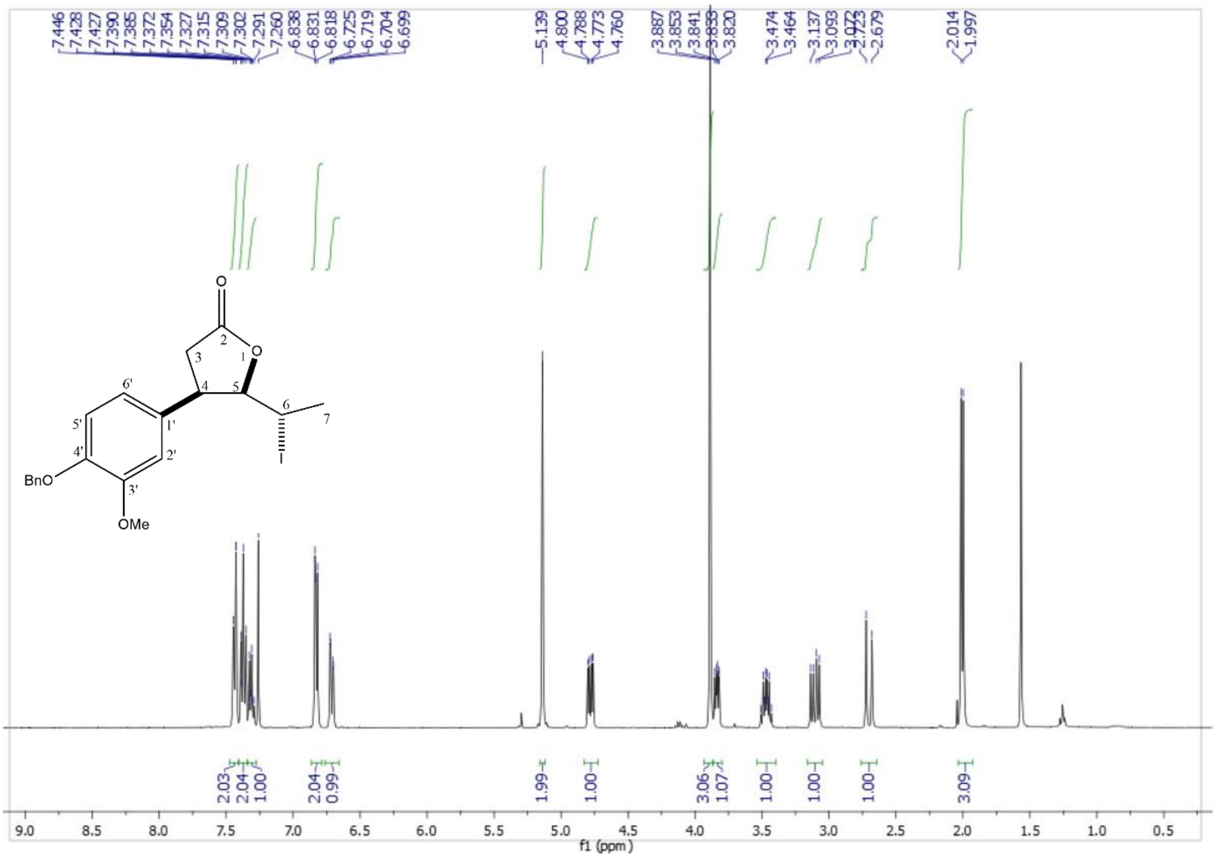

**Figure S31.** $^1$H NMR spectrum of *cis*-4-(4'-benzyloxy-3'-methoxyphenyl)-5-(1-iodoethyl)dihydrofuran-2-one **(7a)**

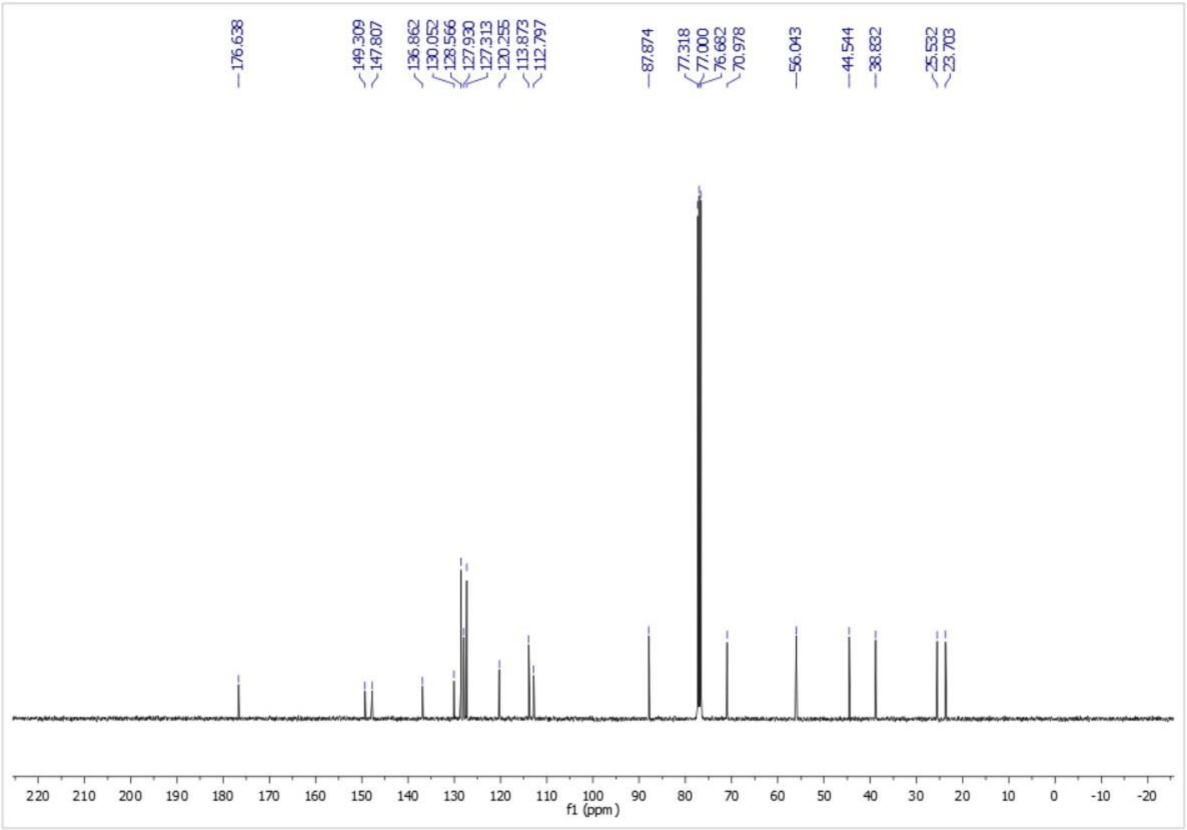

**Figure S32.** $^{13}$C NMR spectrum of *cis*-4-(4'-benzyloxy-3'-methoxyphenyl)-5-(1-iodoethyl)dihydrofuran-2-one **(7a)**

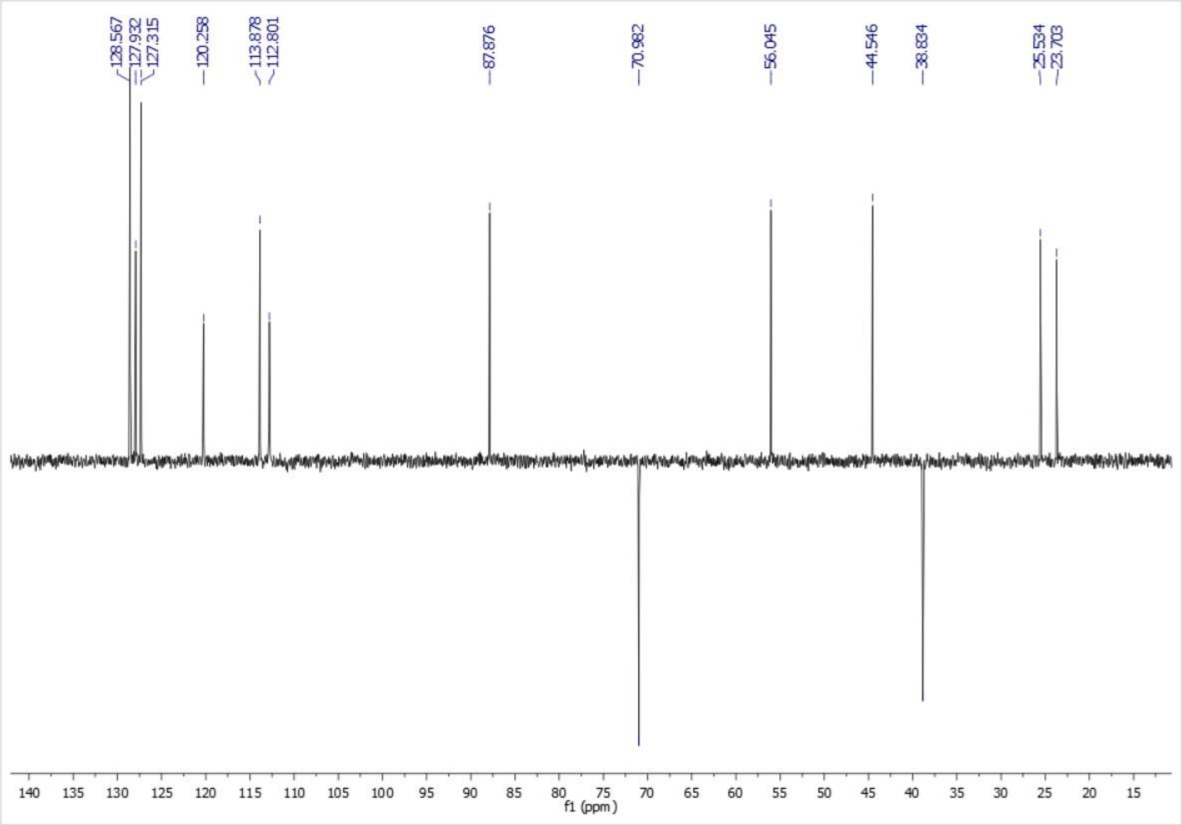


**Figure S33.** DEPT NMR spectrum of *cis*-4-(4'-benzyloxy-3'-methoxyphenyl)-5-(1-iodoethyl)dihydrofuran-2-one **(7a)**

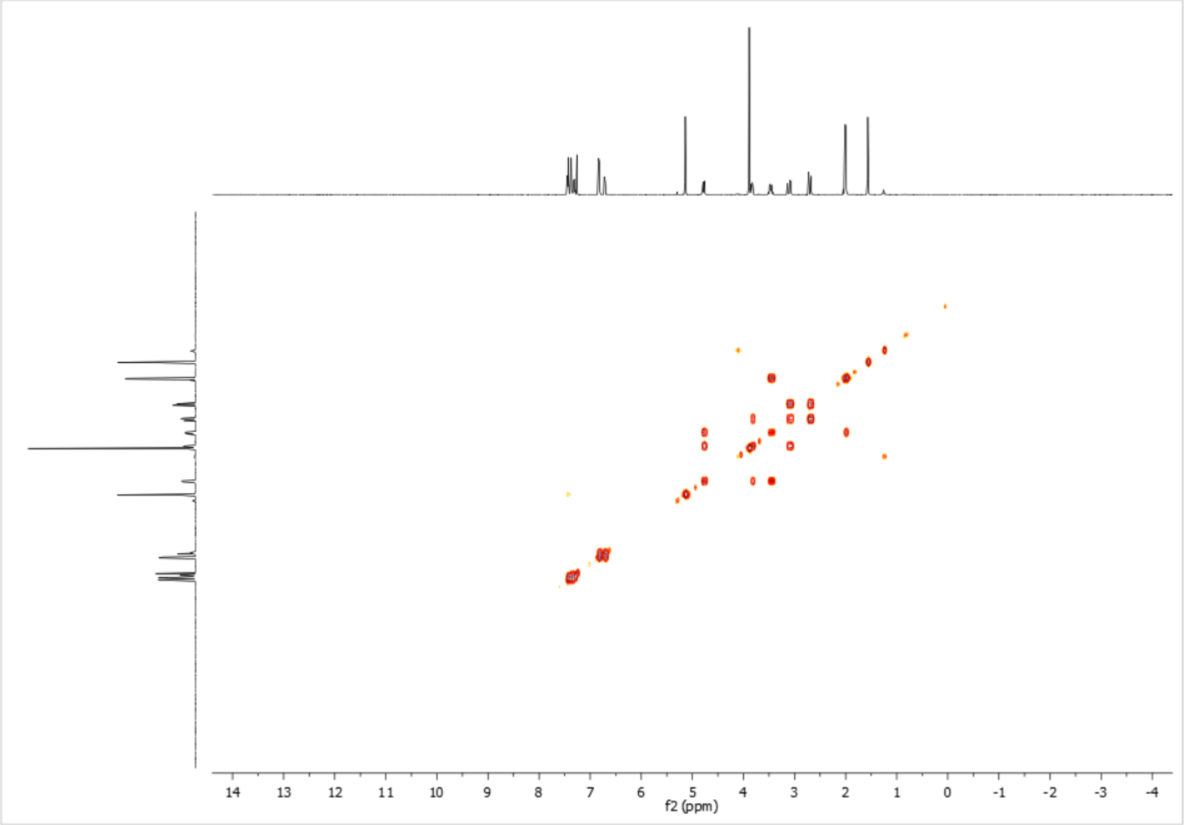


**Figure S34.** COSY spectrum of *cis*-4-(4'-benzyloxy-3'-methoxyphenyl)-5-(1-iodoethyl)dihydrofuran-2-one **(7a)**

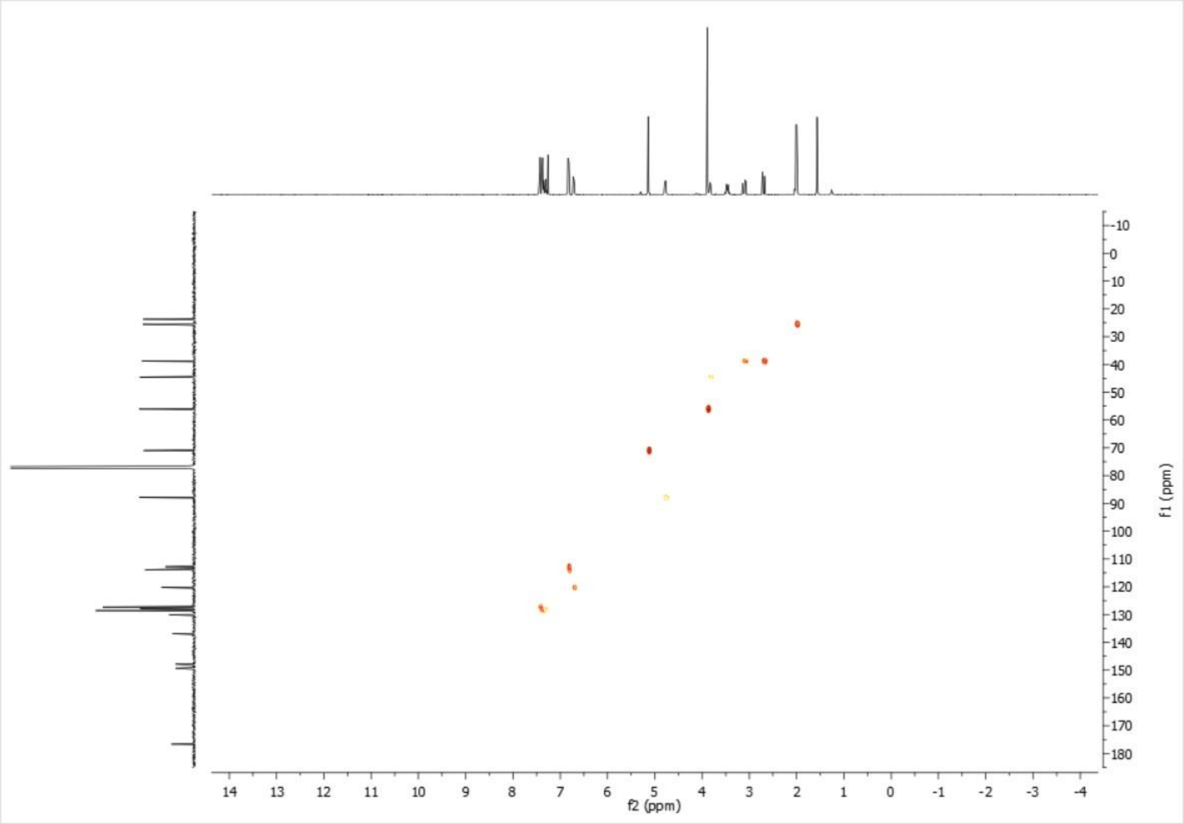


**Figure S35.** HMQC spectrum of *cis*-4-(4'-benzyloxy-3'-methoxyphenyl)-5-(1-iodoethyl)dihydrofuran-2-one **(7a)**

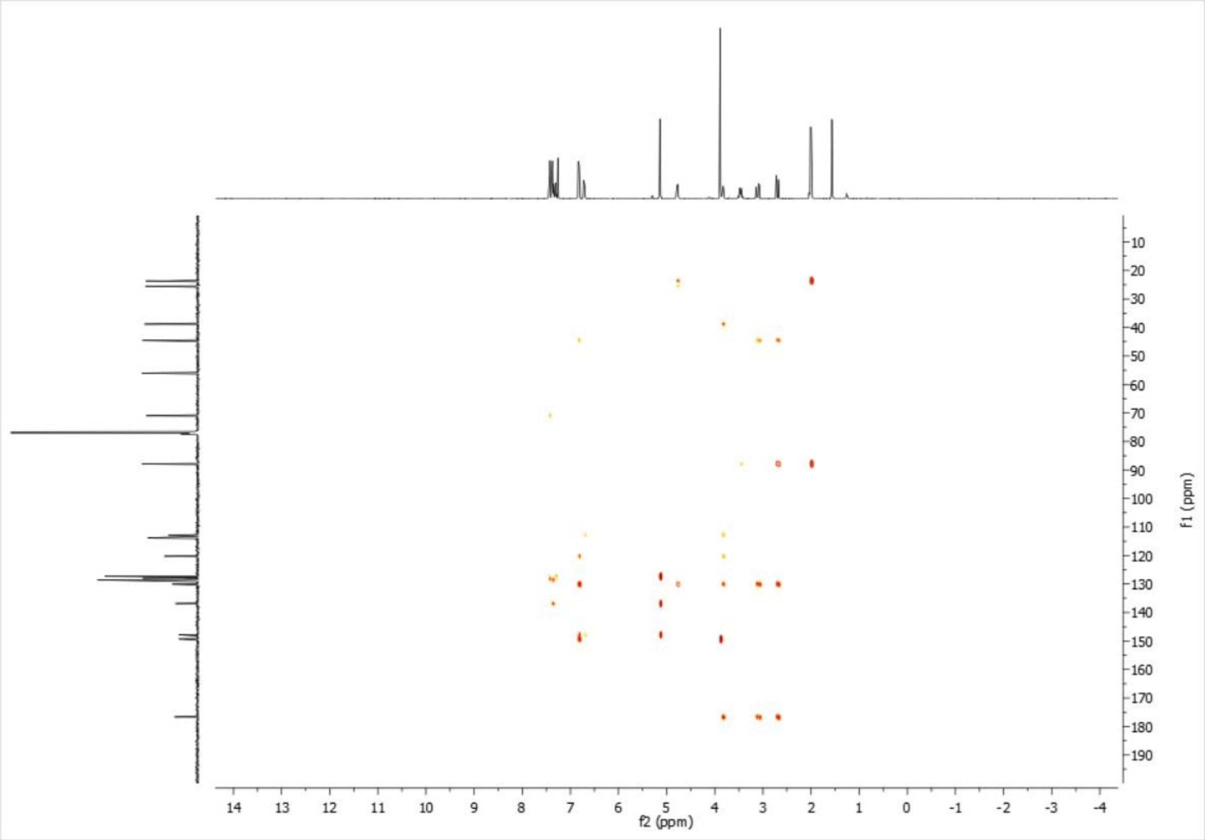


**Figure S36.** HMBC spectrum of *cis*-4-(4'-benzyloxy-3'-methoxyphenyl)-5-(1-iodoethyl)dihydrofuran-2-one **(7a)**

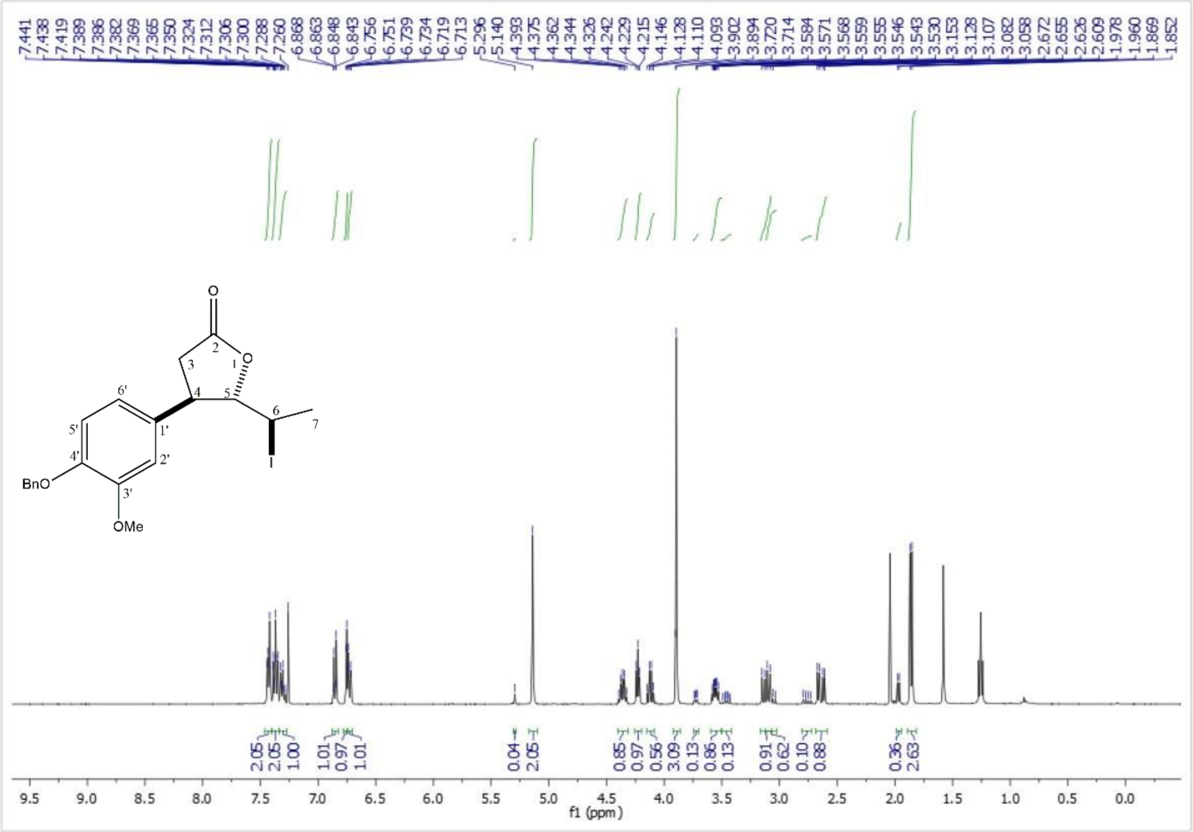

**Figure S37.** $^1$H NMR spectrum of *trans*-4-(4'-benzyloxy-3'-methoxyphenyl)-5-(1-iodoethyl)dihydrofuran-2-one **(7b)**

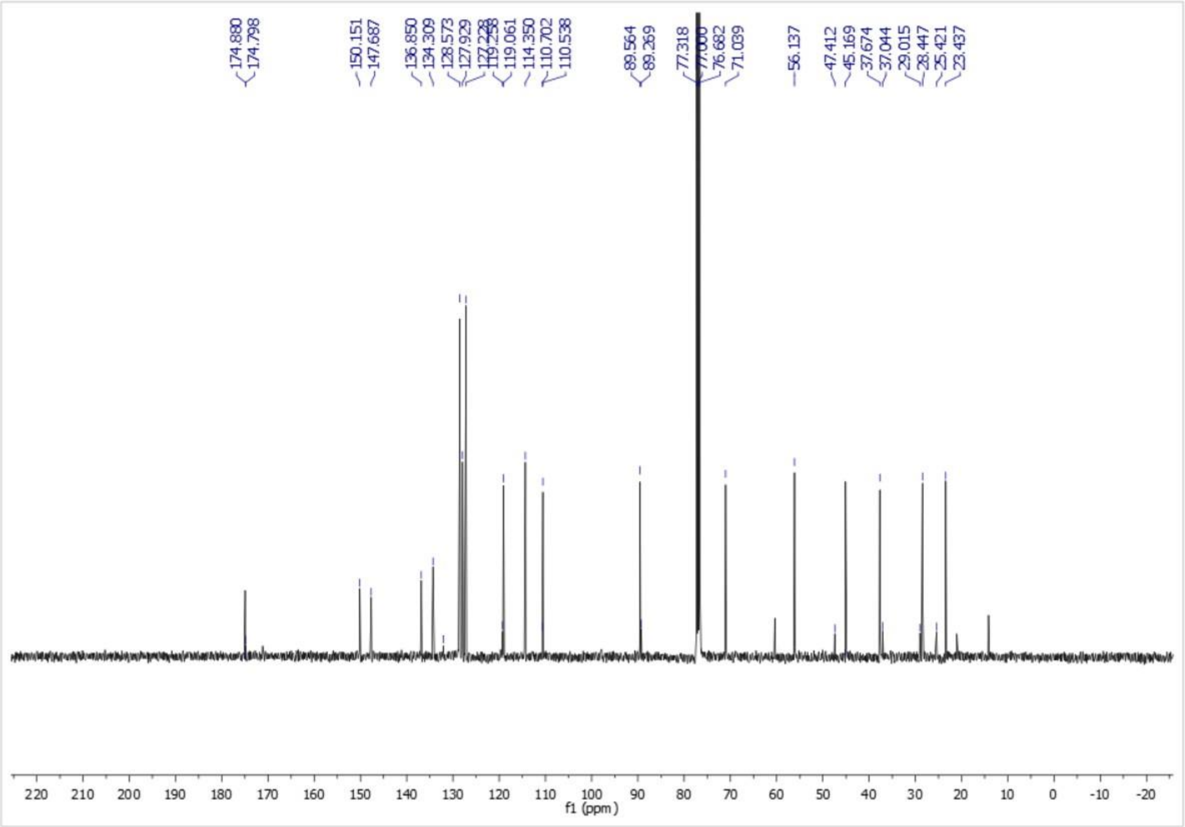

**Figure S38.** $^{13}$C NMR spectrum of *trans*-4-(4'-benzyloxy-3'-methoxyphenyl)-5-(1-iodoethyl)dihydrofuran-2-one **(7b)**

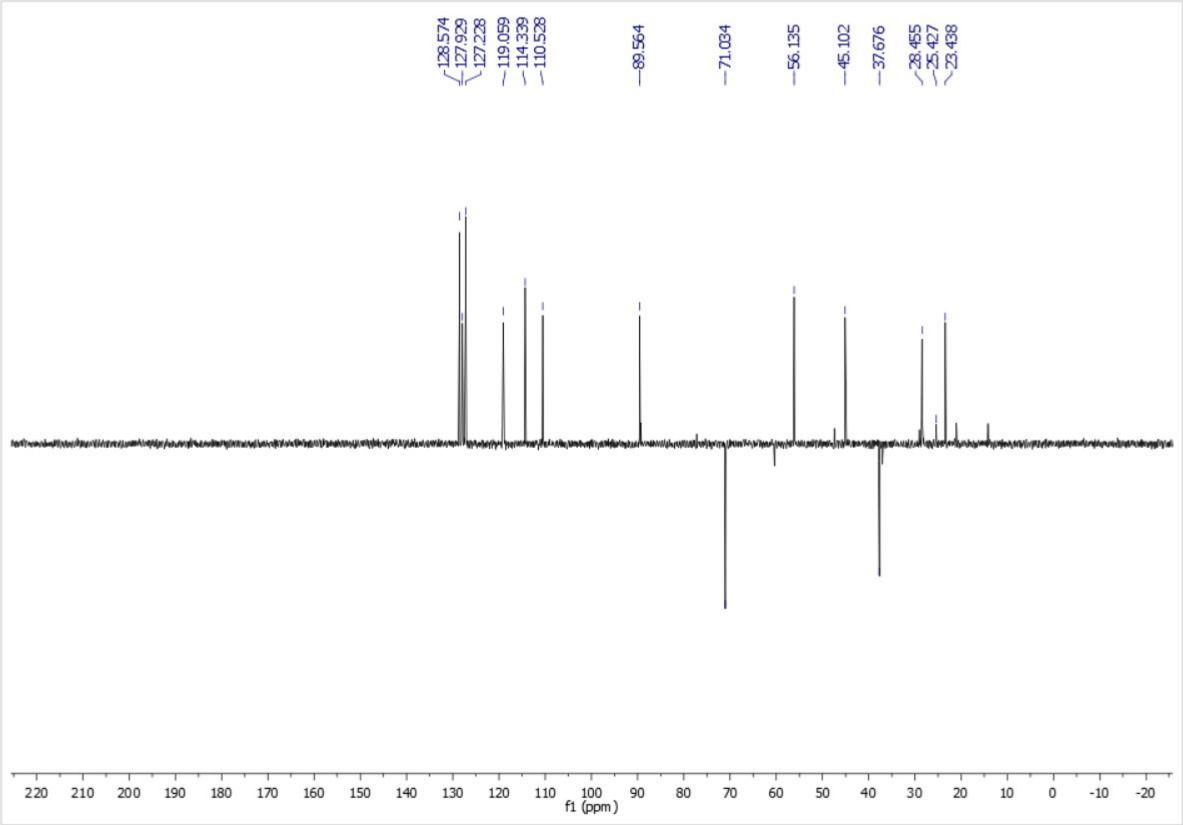


**Figure S39.** DEPT 135 NMR spectrum of *trans*-4-(4'-benzyloxy-3'-methoxyphenyl)-5-(1-iodoethyl)dihydrofuran-2-one **(7b)**

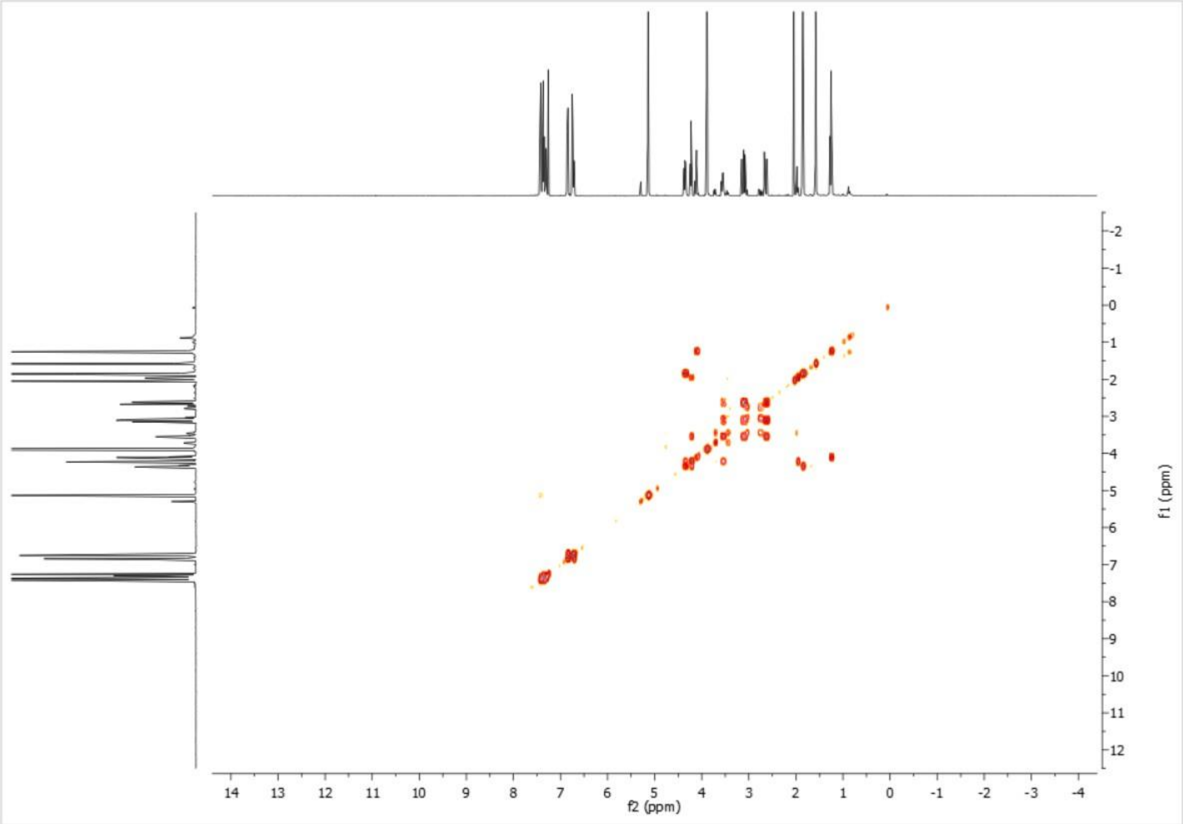


**Figure S40.** COSY spectrum of *trans*-4-(4'-benzyloxy-3'-methoxyphenyl)-5-(1-iodoethyl)dihydrofuran-2-one **(7b)**

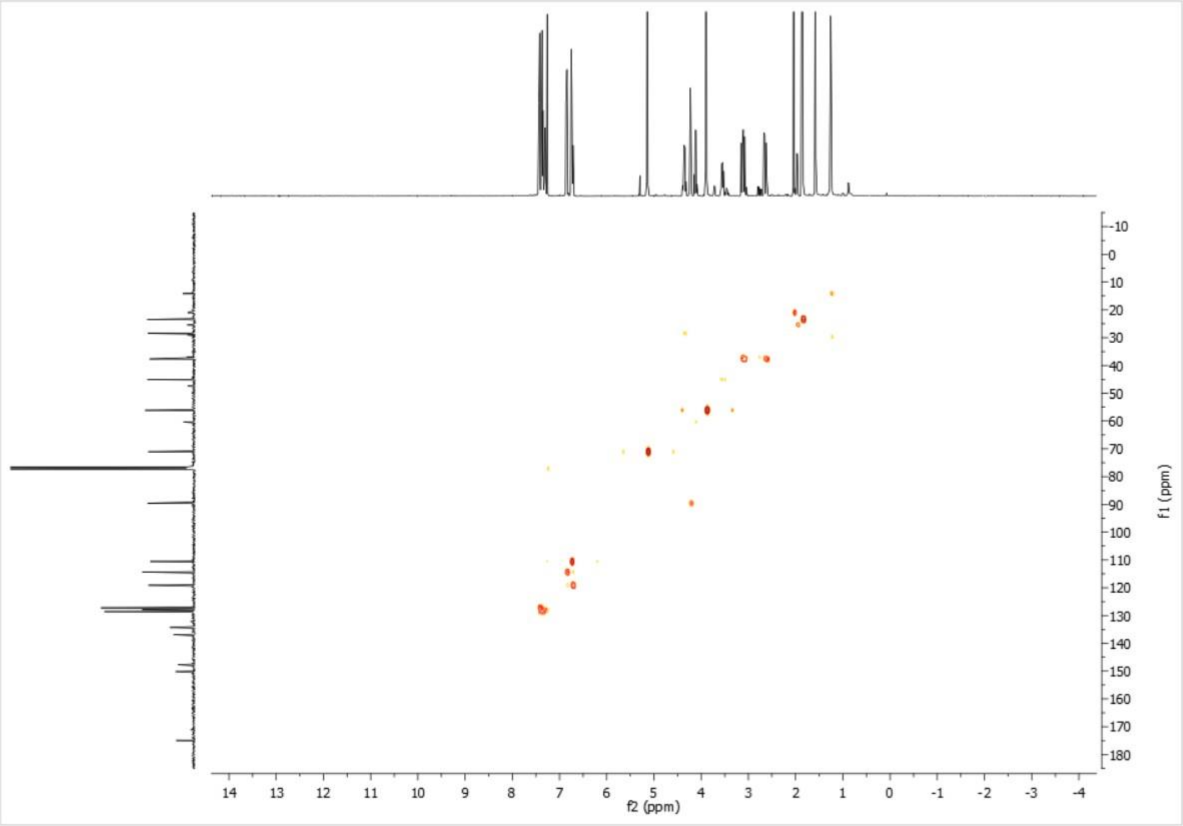


**Figure S41.** HMQC spectrum of *trans*-4-(4'-benzyloxy-3'-methoxyphenyl)-5-(1-iodoethyl)dihydrofuran-2-one **(7b)**

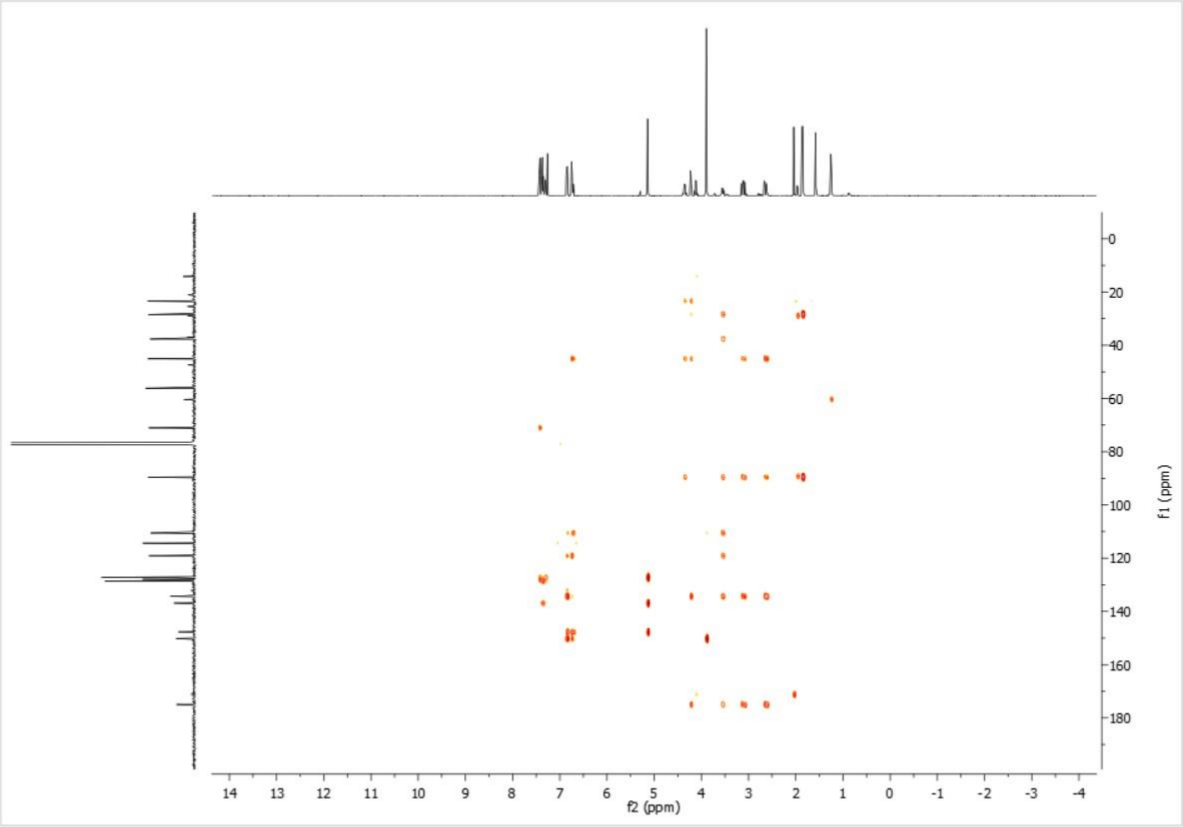


**Figure S42.** HMBC spectrum of *trans*-4-(4'-benzyloxy-3'-methoxyphenyl)-5-(1-iodoethyl)dihydrofuran-2-one **(7b)**

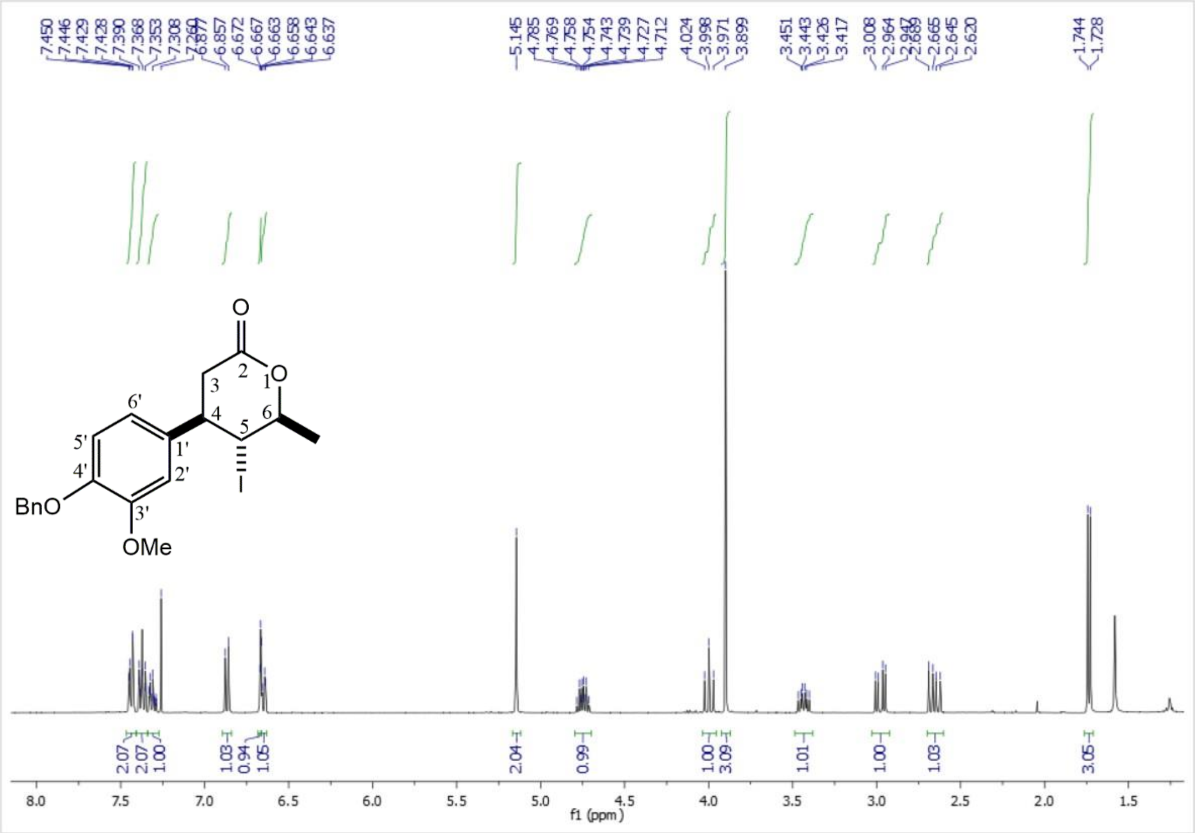


**Figure S43.** $^{1}H$ NMR spectrum of 4-*r*-(4'-benzyloxy-3'-methoxyphenyl)-5-*t*-iodo-6-*c*-methyltetrahydropyran-2-one **(7c)**

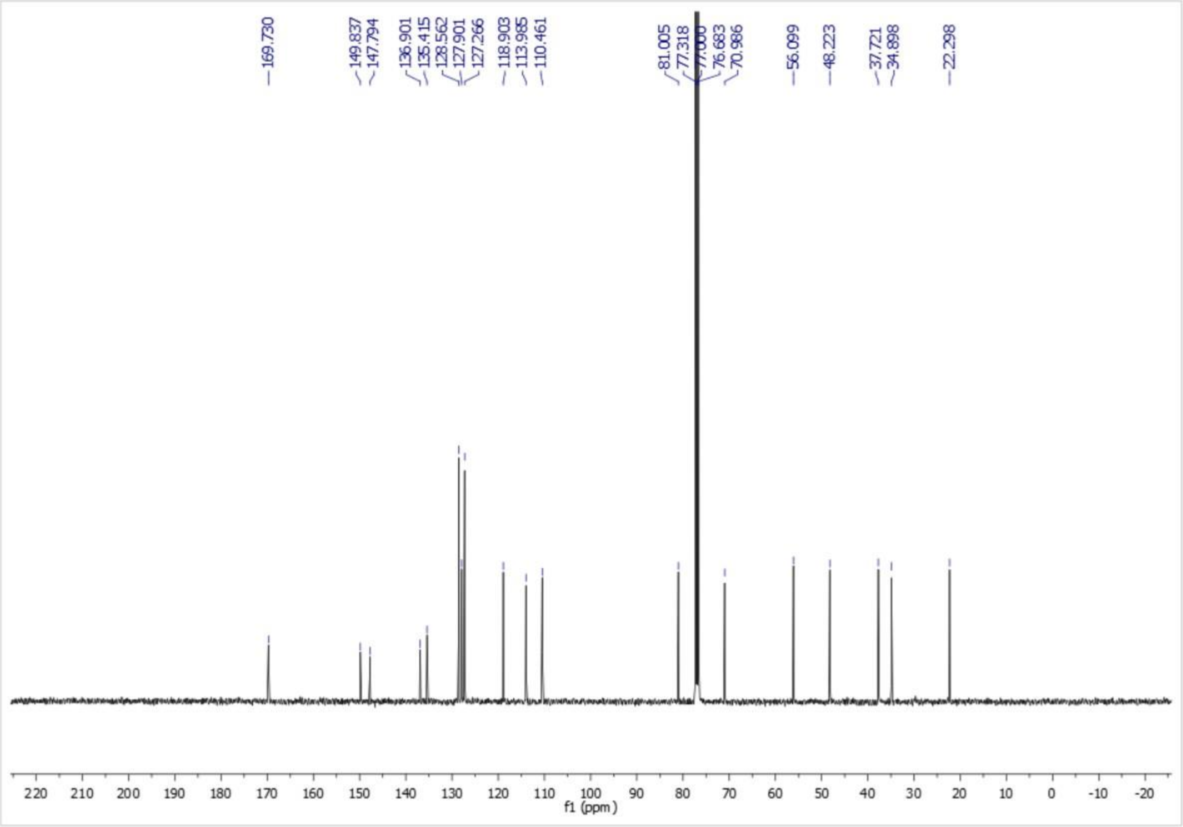


**Figure S44.** $^{13}C$ NMR spectrum of 4-*r*-(4'-benzyloxy-3'-methoxyphenyl)-5-*t*-iodo-6-*c*-methyltetrahydropyran-2-one **(7c)**

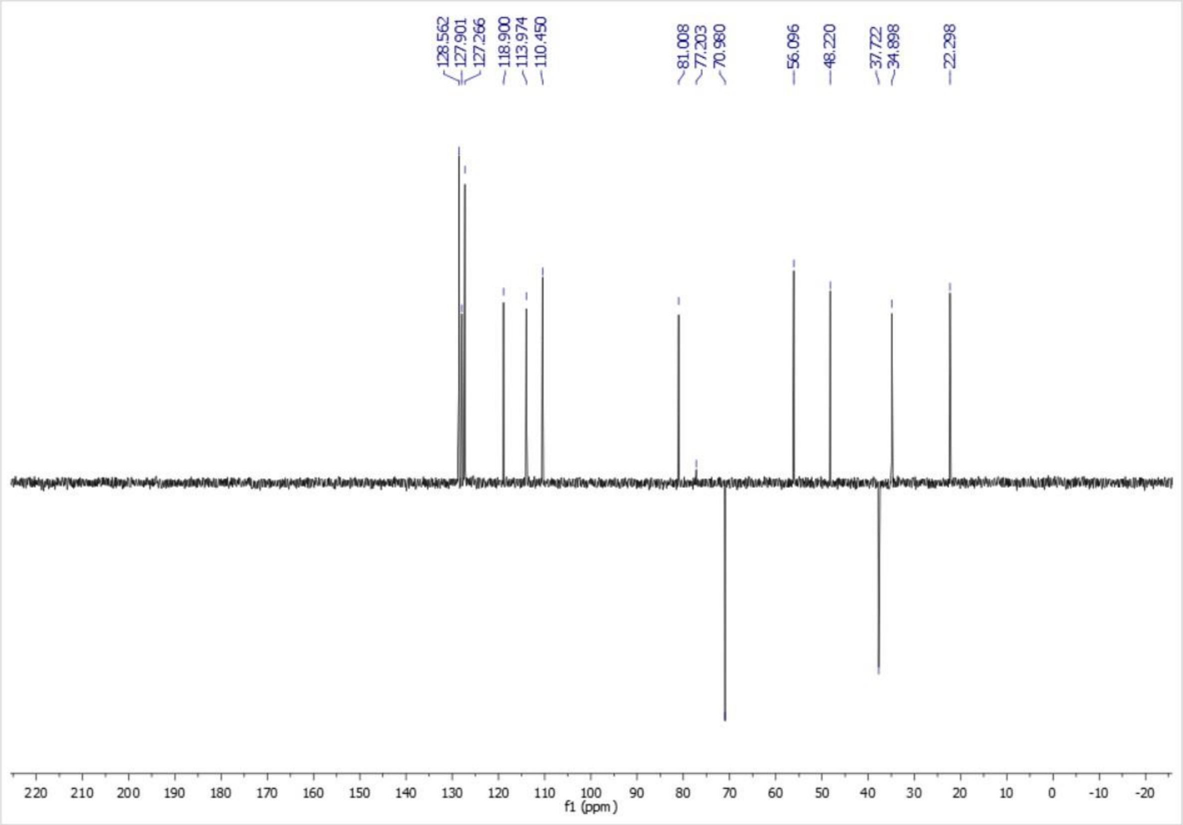


**Figure S45.** DEPT 135 NMR spectrum of 4-*r*-(4'-benzyloxy-3'-methoxyphenyl)-5-*t*-iodo-6-*c*-methyltetrahydropyran-2-one **(7c)**

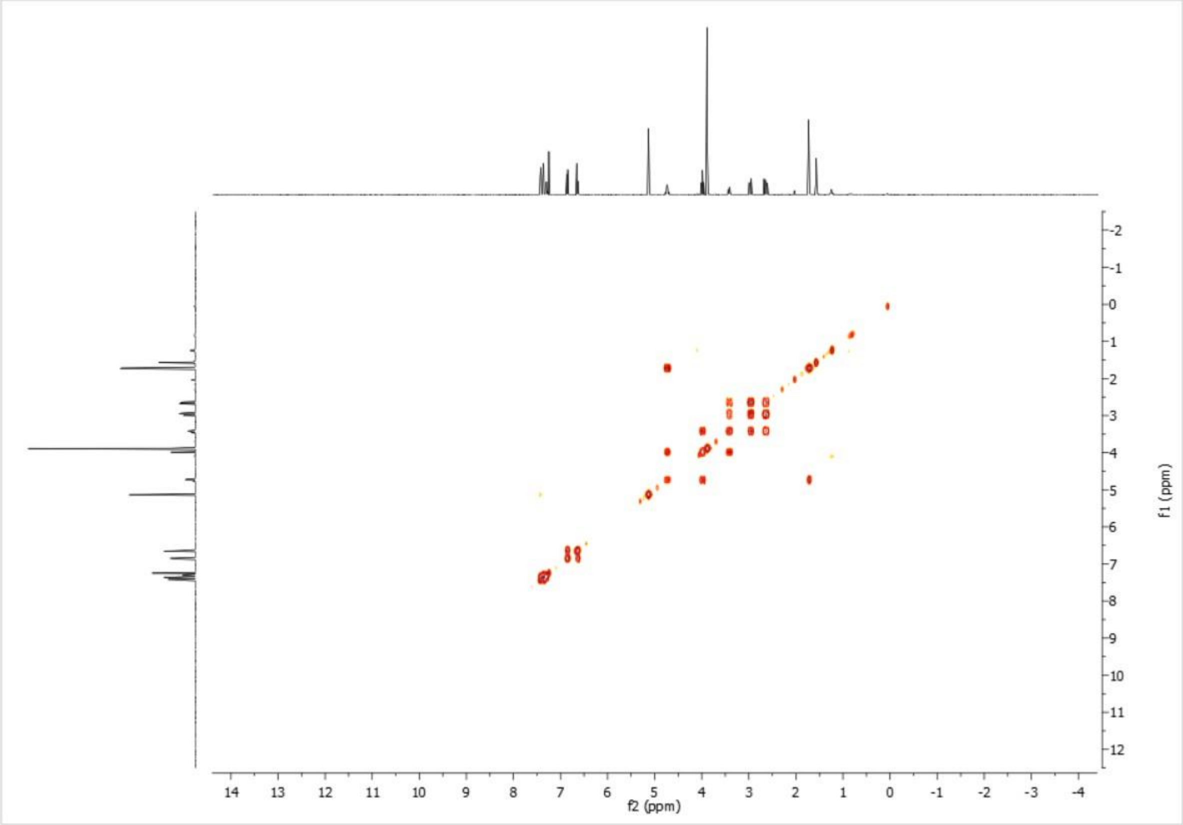


**Figure S46.** COSY spectrum of 4-*r*-(4'-benzyloxy-3'-methoxyphenyl)-5-*t*-iodo-6-*c*-methyltetrahydropyran-2-one **(7c)**

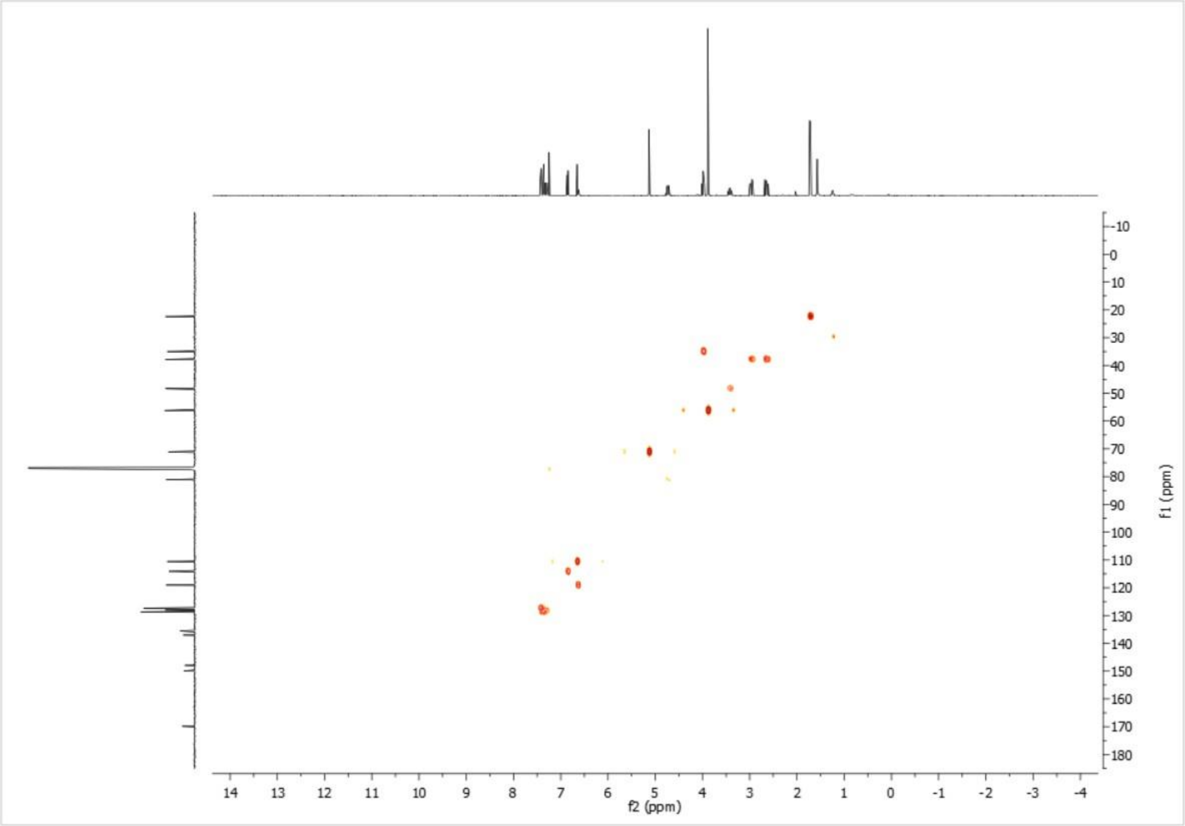


**Figure S47.** HMQC spectrum of 4-*r*-(4'-benzyloxy-3'-methoxyphenyl)-5-*t*-iodo-6-*c*-methyltetrahydropyran-2-one **(7c)**

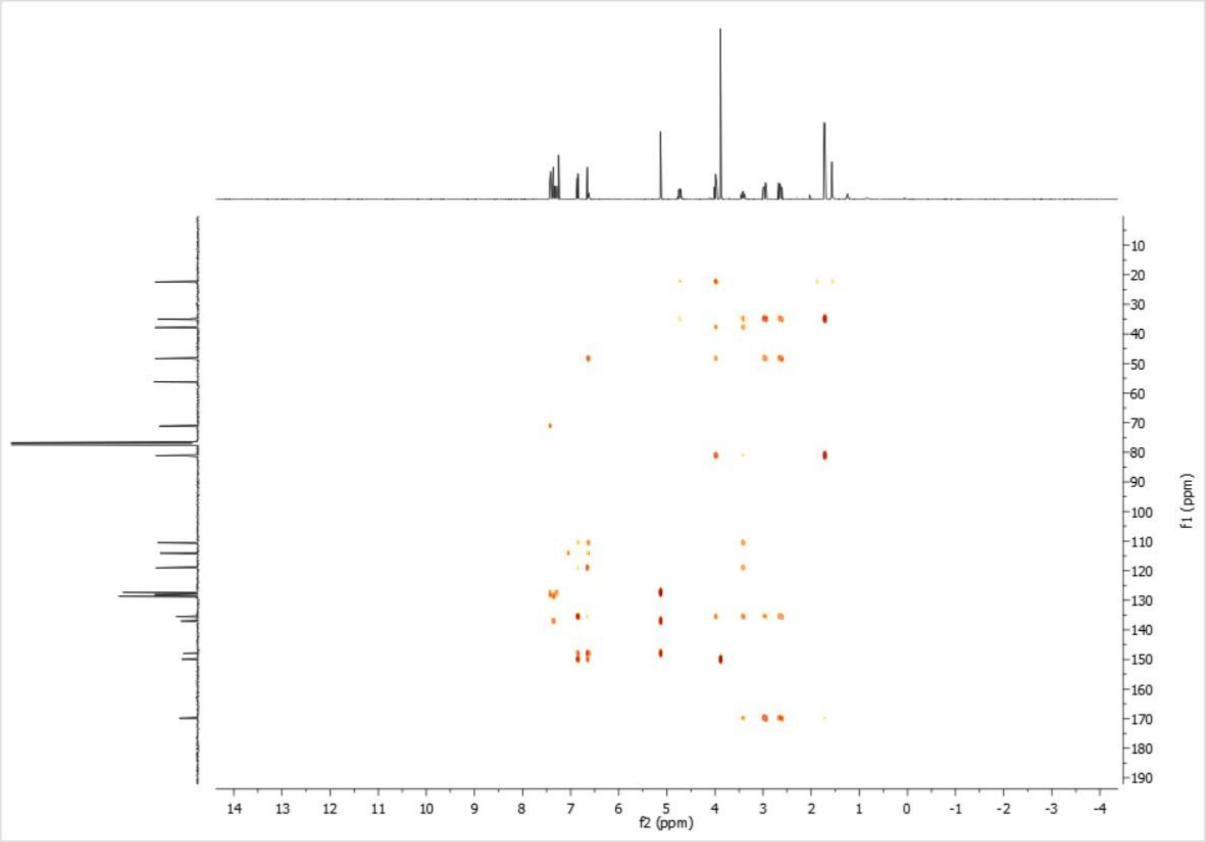


**Figure S48.** HMBC spectrum of 4-*r*-(4'-benzyloxy-3'-methoxyphenyl)-5-*t*-iodo-6-*c*-methyltetrahydropyran-2-one **(7c)**

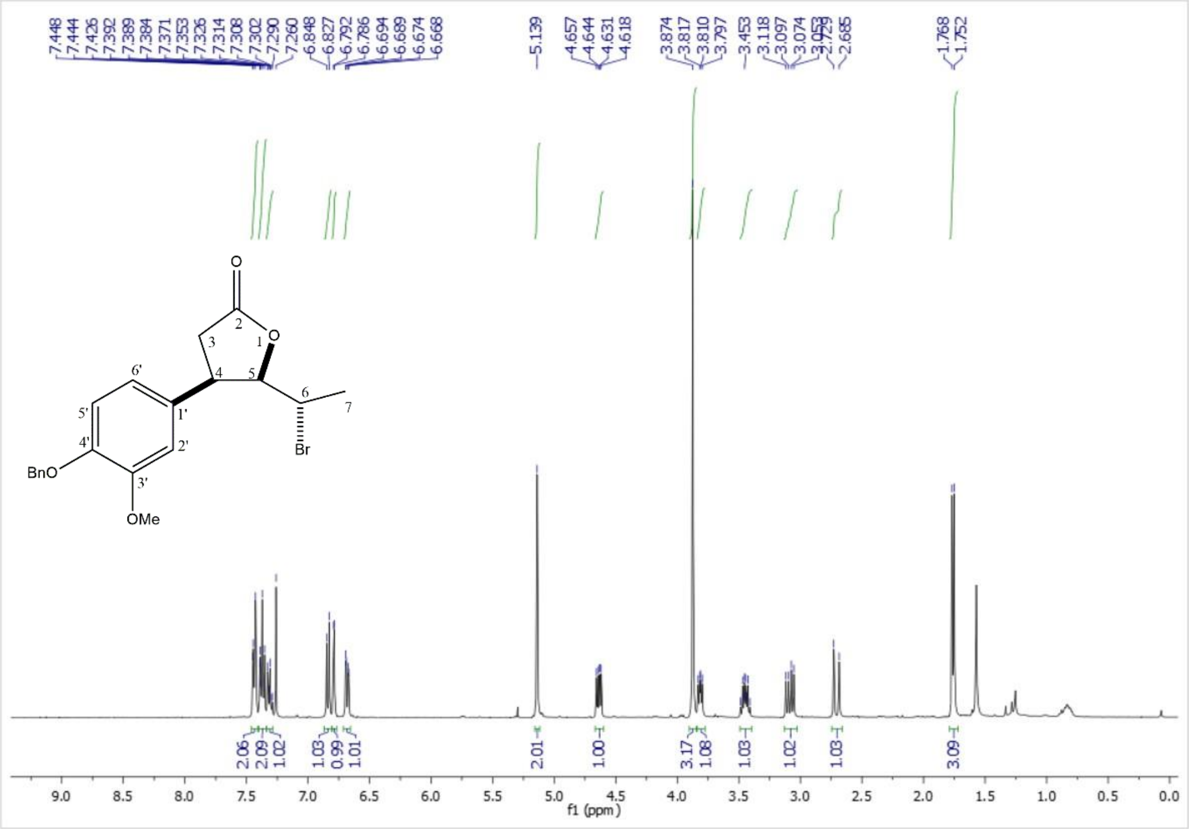

**Figure S49.** $^{1}H$ NMR spectrum of *cis*-4-(4'-benzyloxy-3'-methoxyphenyl)-5-(1-bromoethyl)dihydrofuran-2-one **(8a)**

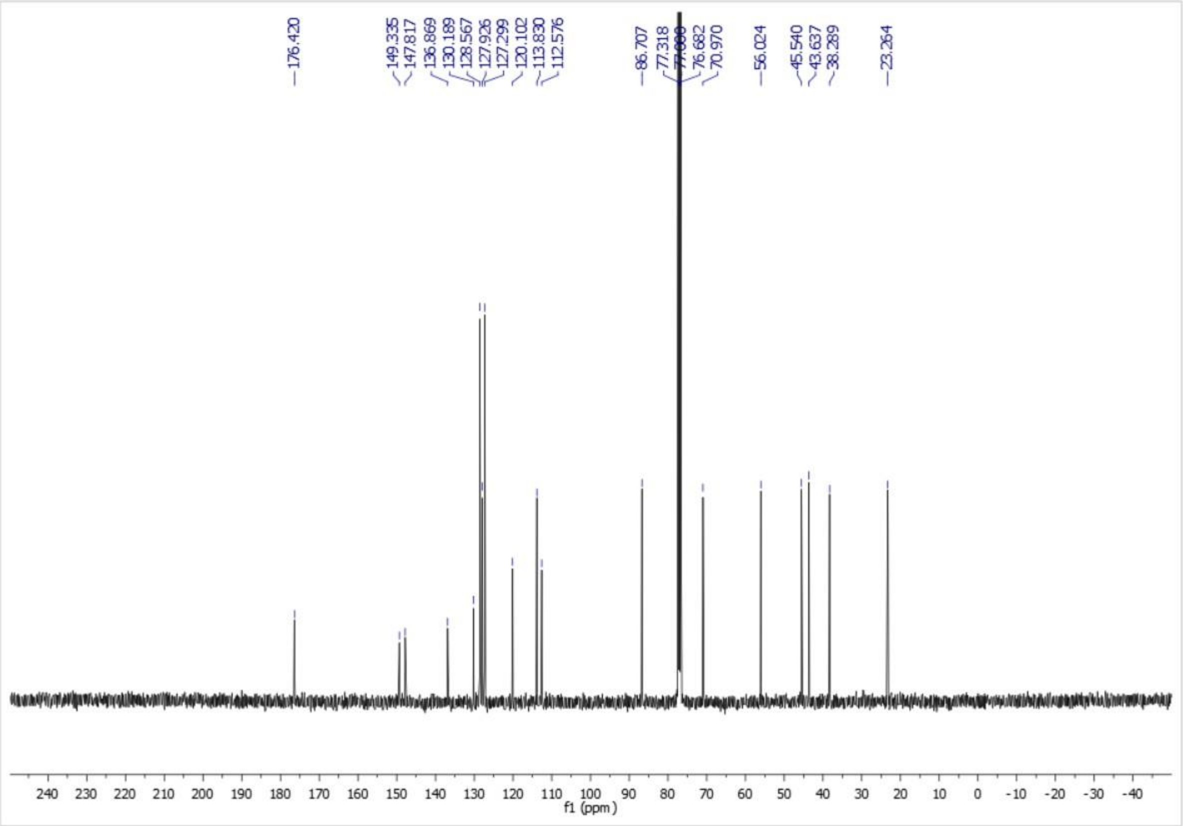

**Figure S50.** $^{13}C$ NMR spectrum of *cis*-4-(4'-benzyloxy-3'-methoxyphenyl)-5-(1-bromoethyl)dihydrofuran-2-one **(8a)**

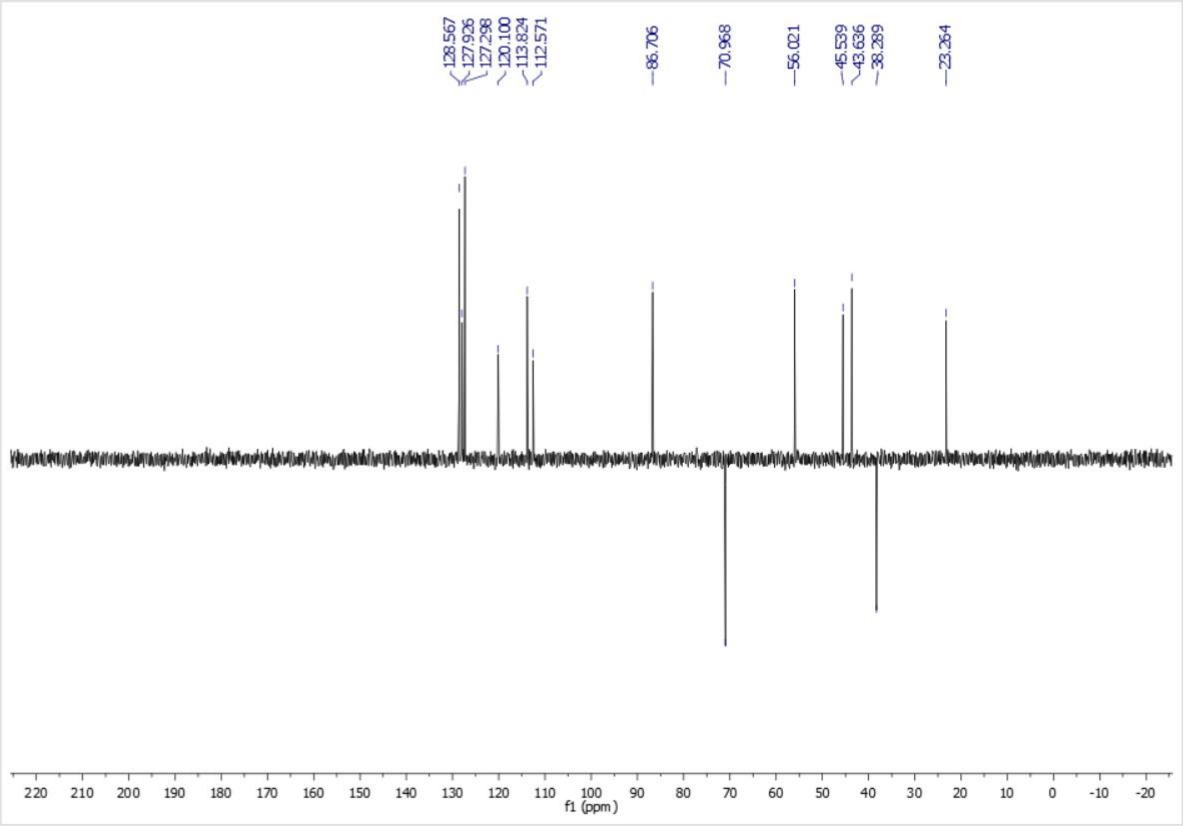


**Figure S51.** DEPT 135 NMR spectrum of *cis*-4-(4'-benzyloxy-3'-methoxyphenyl)-5-(1-bromoethyl)dihydrofuran-2-one **(8a)**

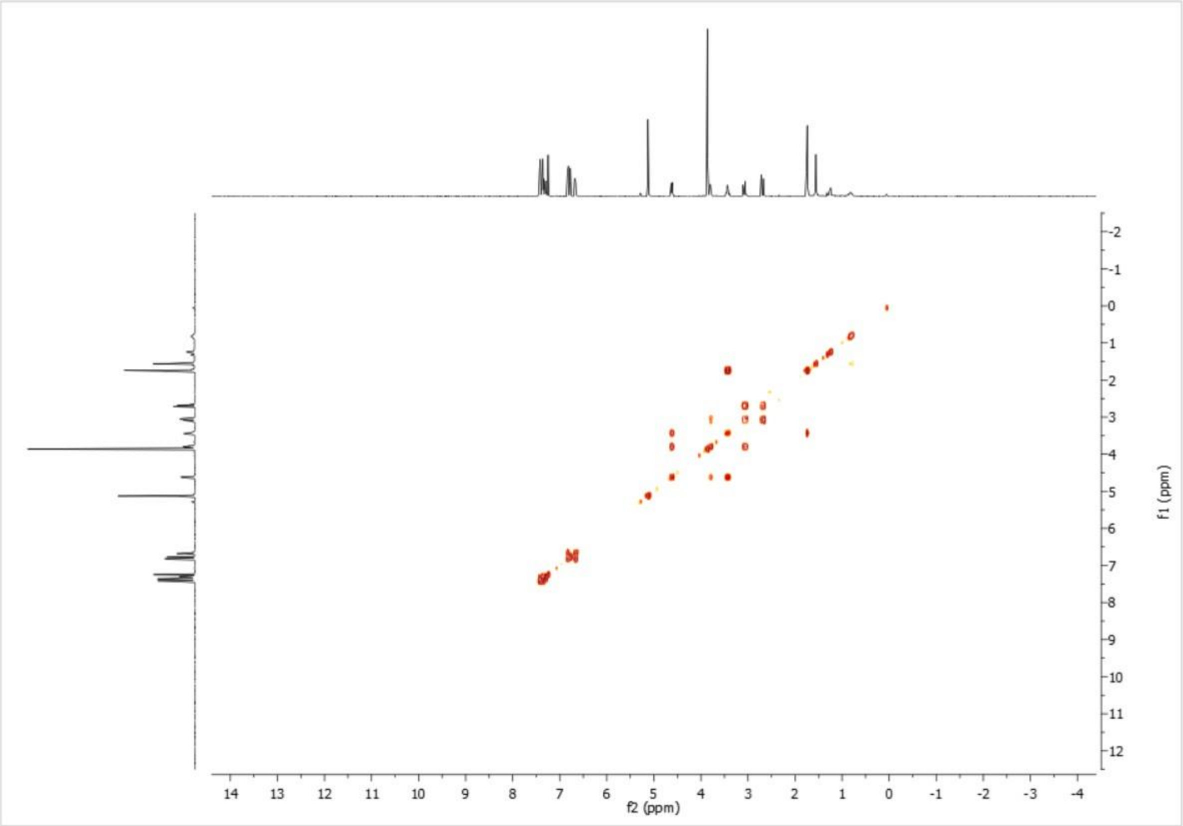


**Figure S52.** COSY spectrum of *cis*-4-(4'-benzyloxy-3'-methoxyphenyl)-5-(1-bromoethyl)dihydrofuran-2-one **(8a)**

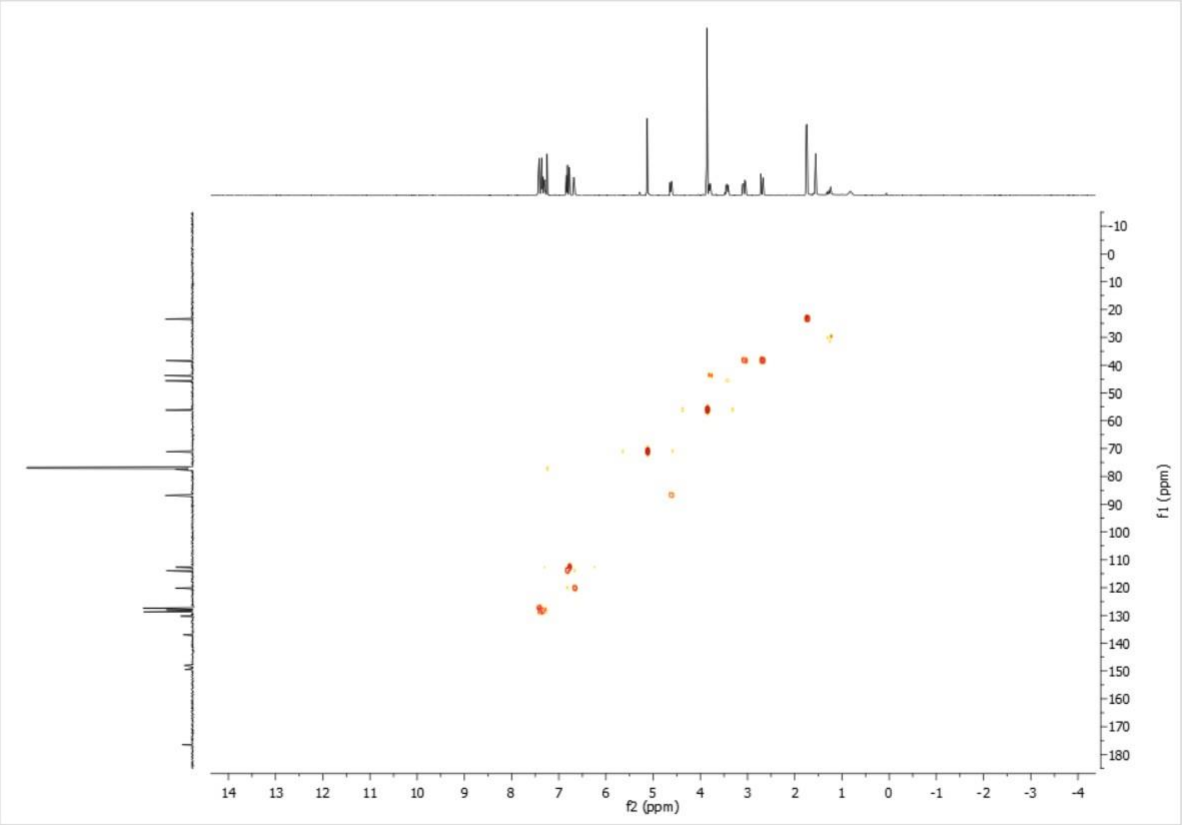


**Figure S53.** HMQC spectrum of *cis*-4-(4'-benzyloxy-3'-methoxyphenyl)-5-(1-bromoethyl)dihydrofuran-2-one **(8a)**

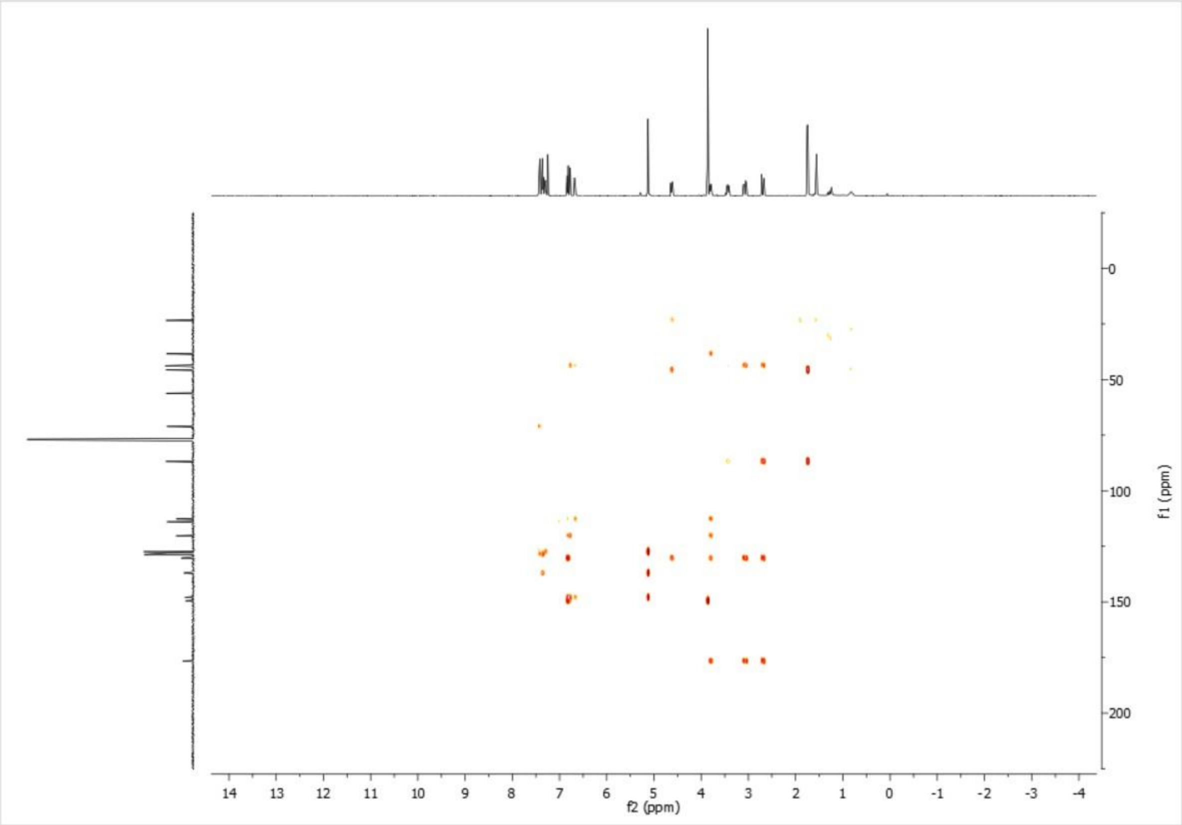


**Figure S54.** HMBC spectrum of *cis*-4-(4'-benzyloxy-3'-methoxyphenyl)-5-(1-bromoethyl)dihydrofuran-2-one **(8a)**

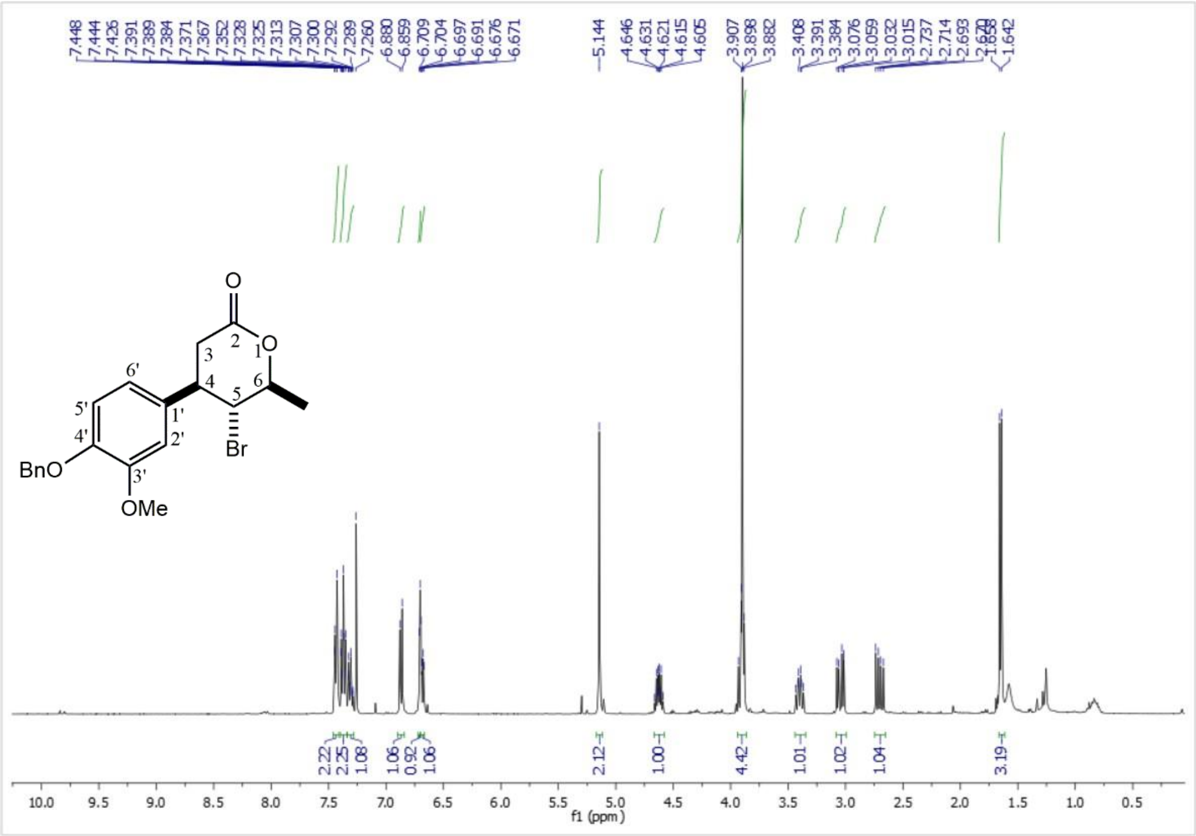


**Figure S55.** $^{1}$H NMR spectrum of 4-*r*-(4'-benzyloxy-3'-methoxyphenyl)-5-*t*-bromo-6-*c*-methyltetrahydropyran-2-one **(8b)**

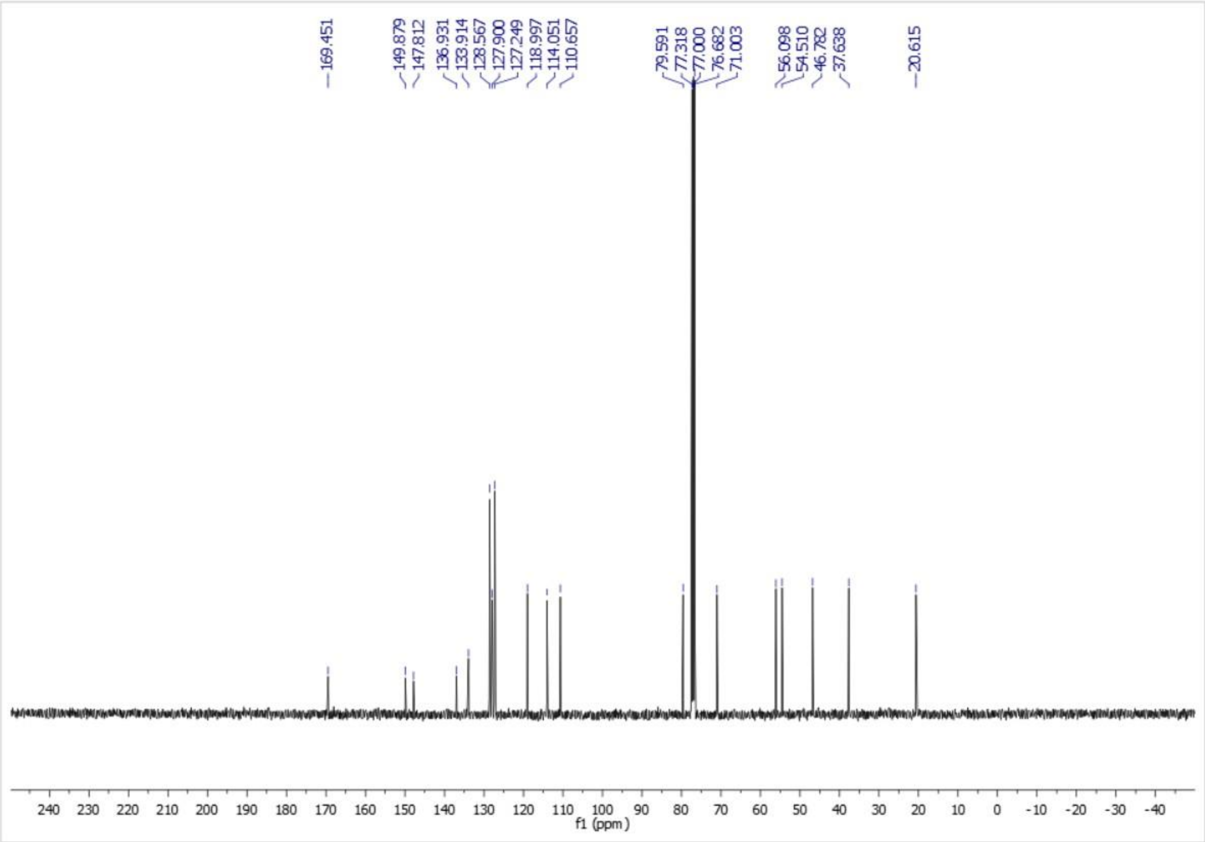


**Figure S56.** $^{13}$C NMR spectrum of 4-*r*-(4'-benzyloxy-3'-methoxyphenyl)-5-*t*-bromo-6-*c*-methyltetrahydropyran-2-one **(8b)**

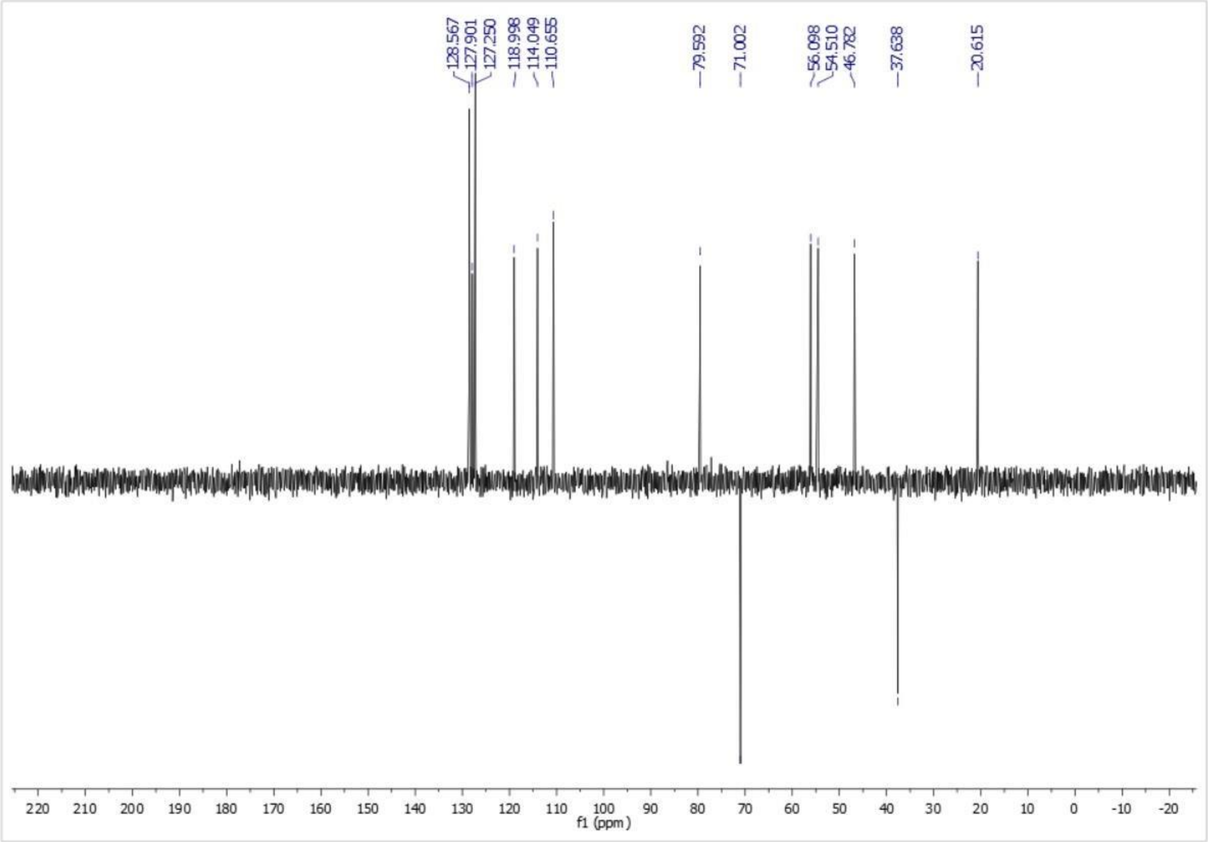


**Figure S57.** DEPT 135 NMR spectrum of 4-*r*-(4'-benzyloxy-3'-methoxyphenyl)-5-*t*-bromo-6-*c*-methyltetrahydropyran-2-one **(8b)**

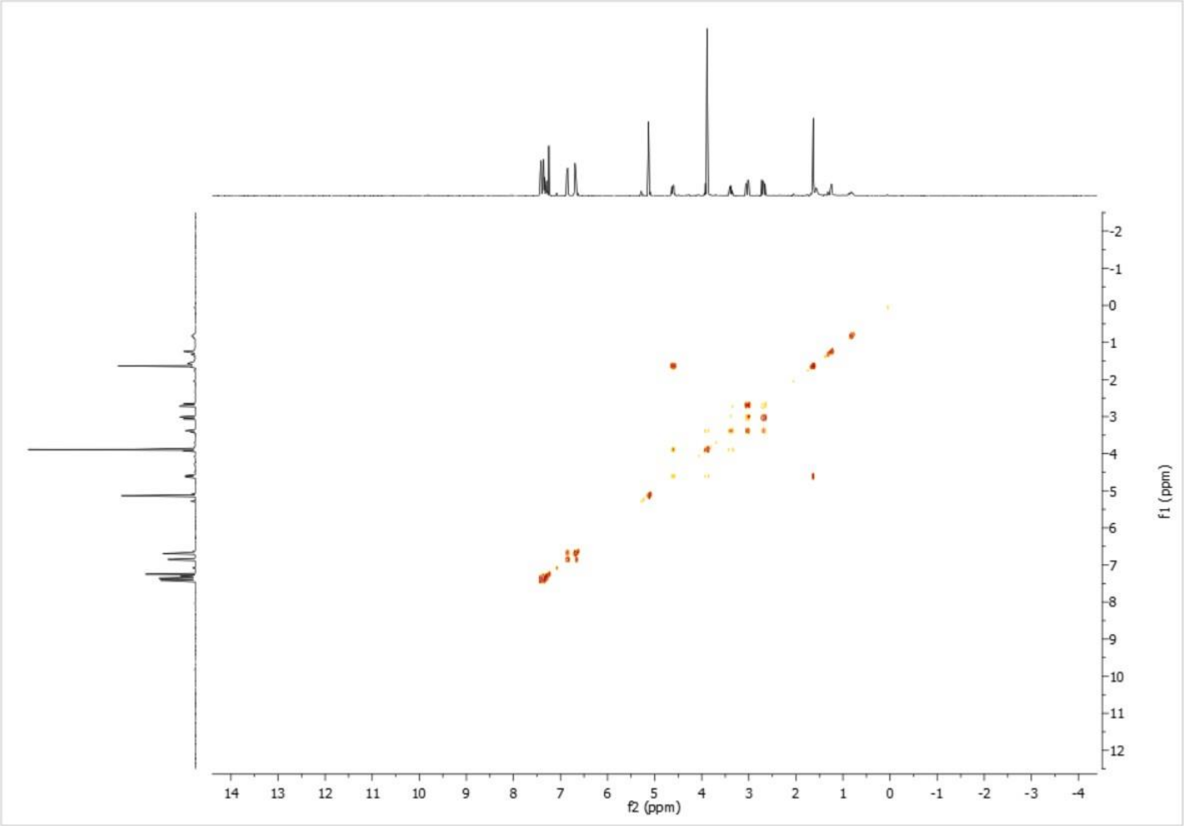


**Figure S58.** COSY spectrum of 4-*r*-(4'-benzyloxy-3'-methoxyphenyl)-5-*t*-bromo-6-*c*-methyltetrahydropyran-2-one **(8b)**

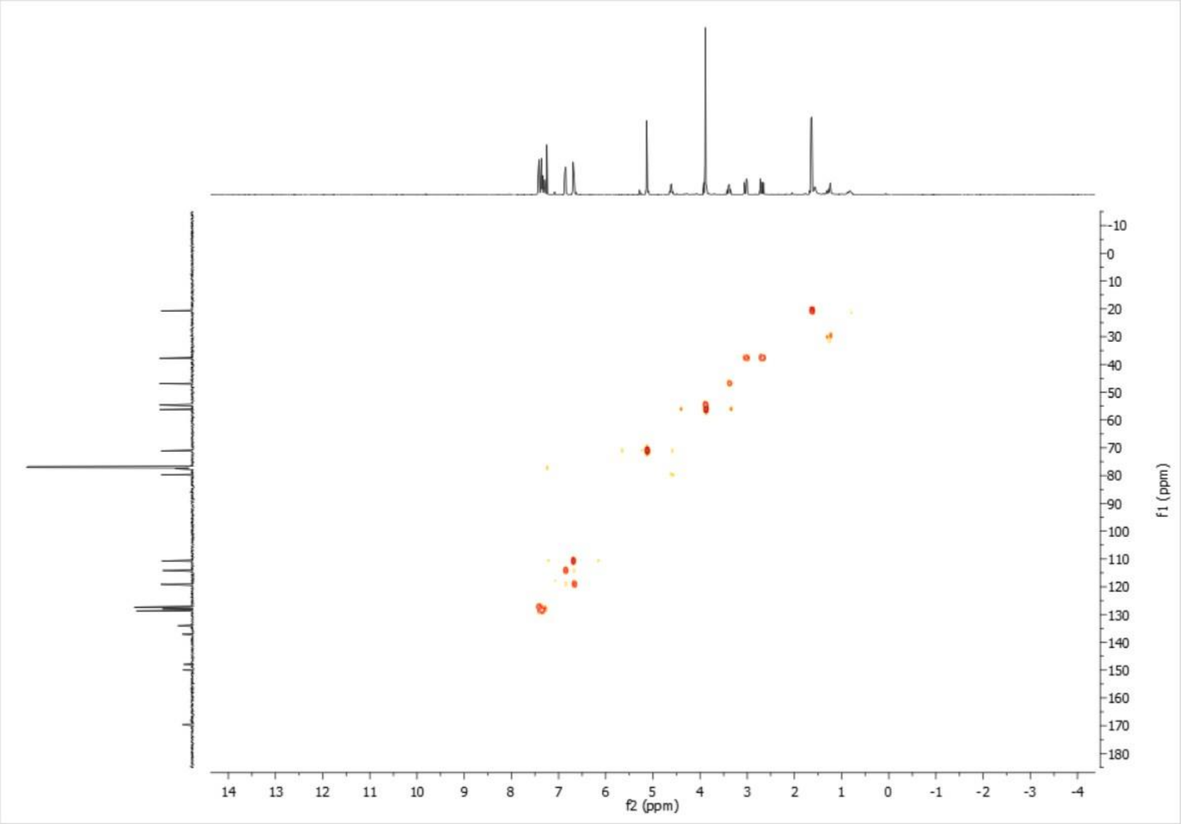


**Figure S59.** HMQC spectrum of 4-*r*-(4'-benzyloxy-3'-methoxyphenyl)-5-*t*-bromo-6-*c*-methyltetrahydropyran-2-one **(8b)**

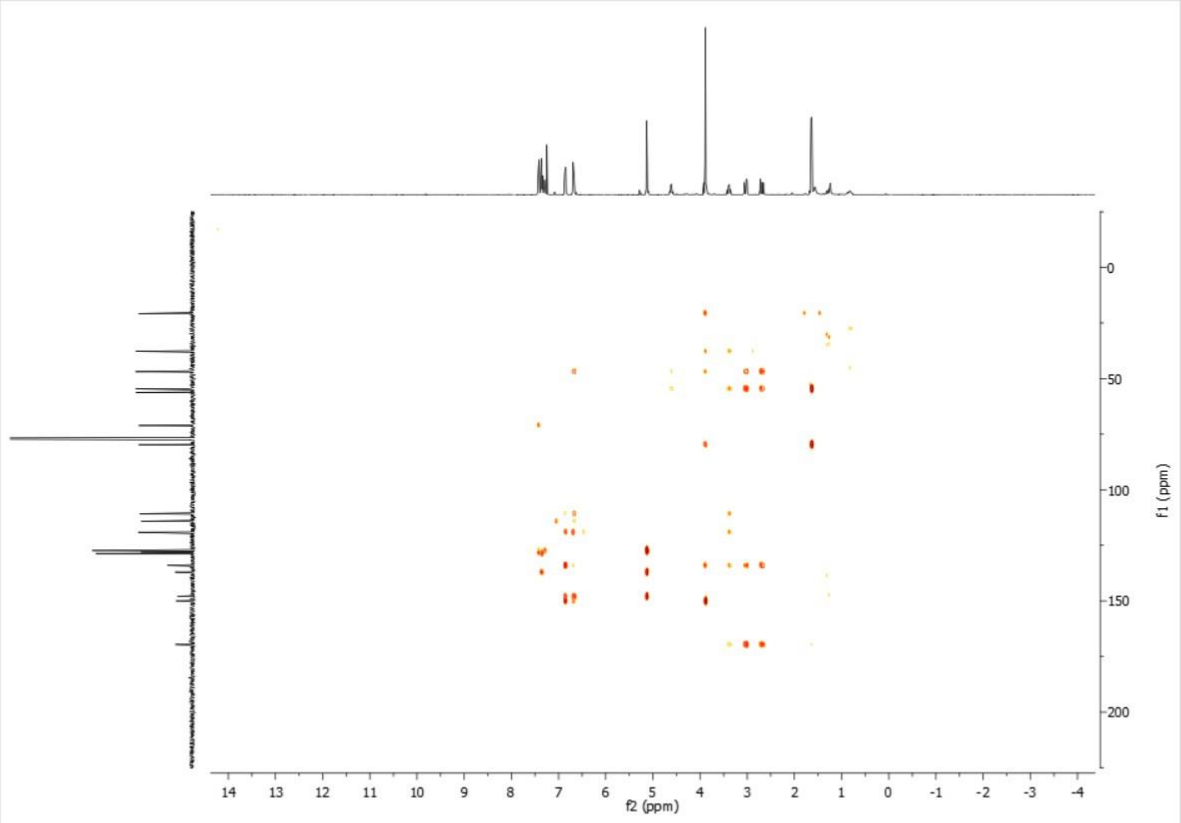


**Figure S60.** HMBC spectrum of 4-*r*-(4'-benzyloxy-3'-methoxyphenyl)-5-*t*-bromo-6-*c*-methyltetrahydropyran-2-one **(8b)**

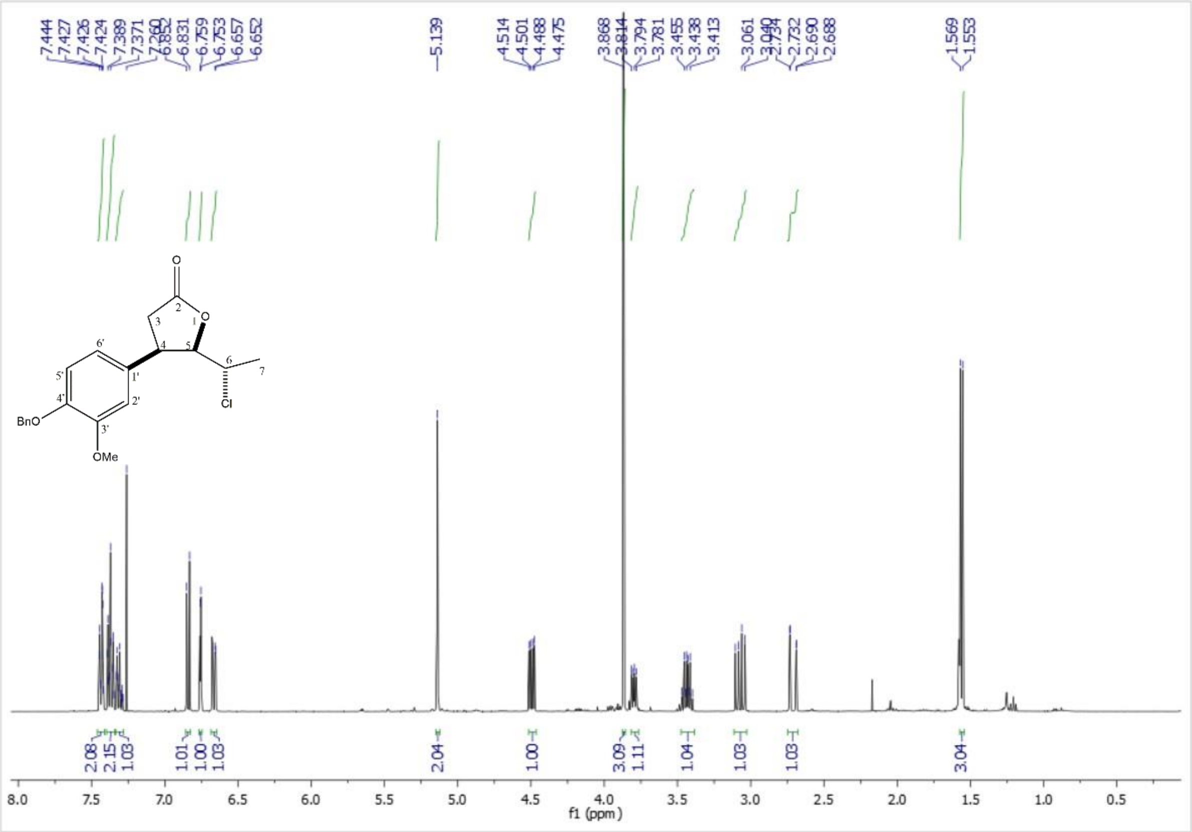

**Figure S61.** $^{1}$H NMR spectrum of *cis*-4-(4'-benzyloxy-3'-methoxyphenyl)-5-(1-chloroethyl)dihydrofuran-2-one **(9a)**

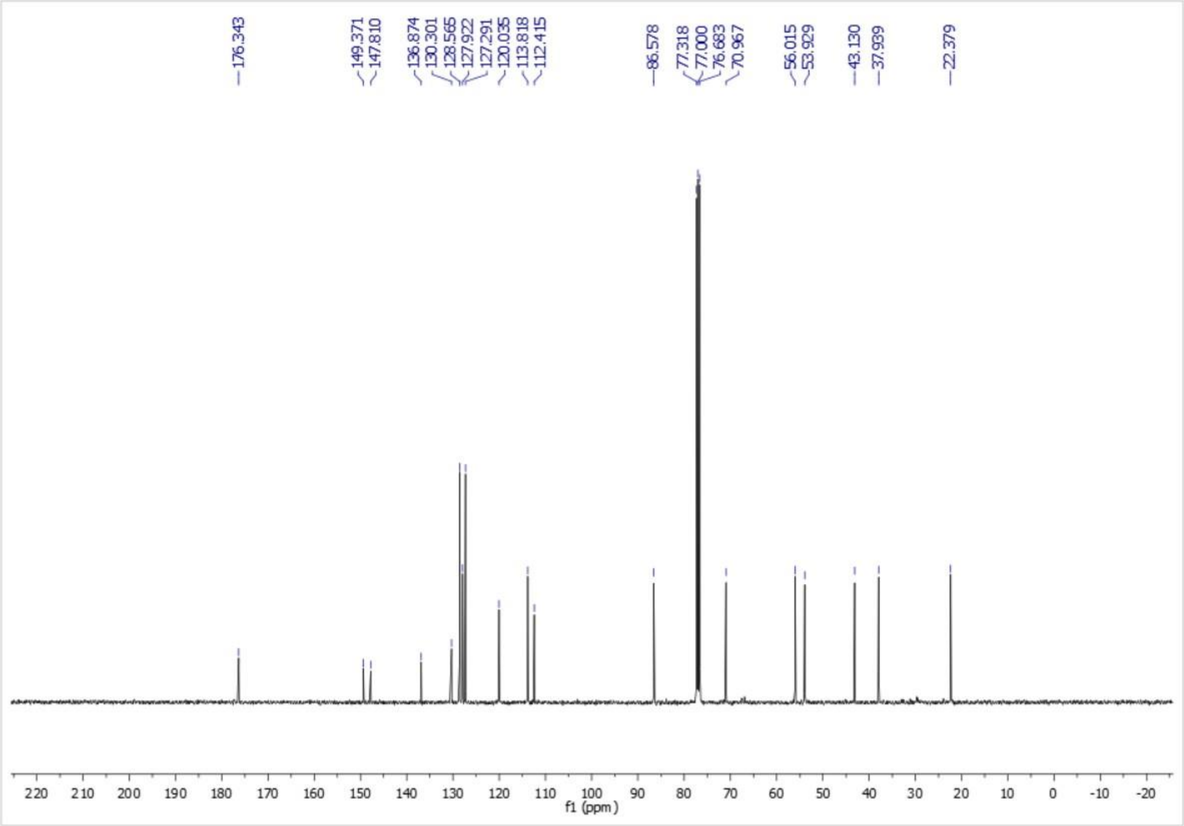

**Figure S62.** $^{13}$C NMR spectrum of *cis*-4-(4'-benzyloxy-3'-methoxyphenyl)-5-(1-chloroethyl)dihydrofuran-2-one **(9a)**

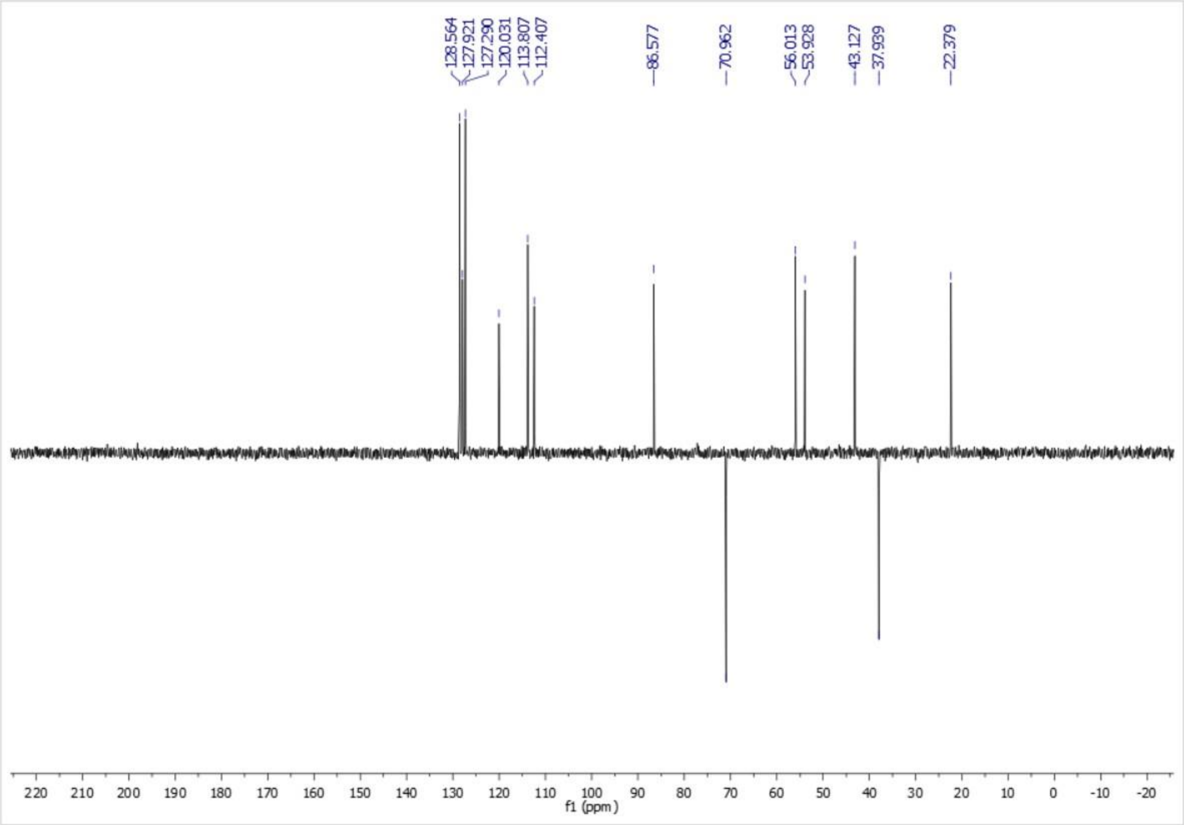


**Figure S63.** DEPT 135 NMR spectrum of *cis*-4-(4'-benzyloxy-3'-methoxyphenyl)-5-(1-chloroethyl)dihydrofuran-2-one **(9a)**

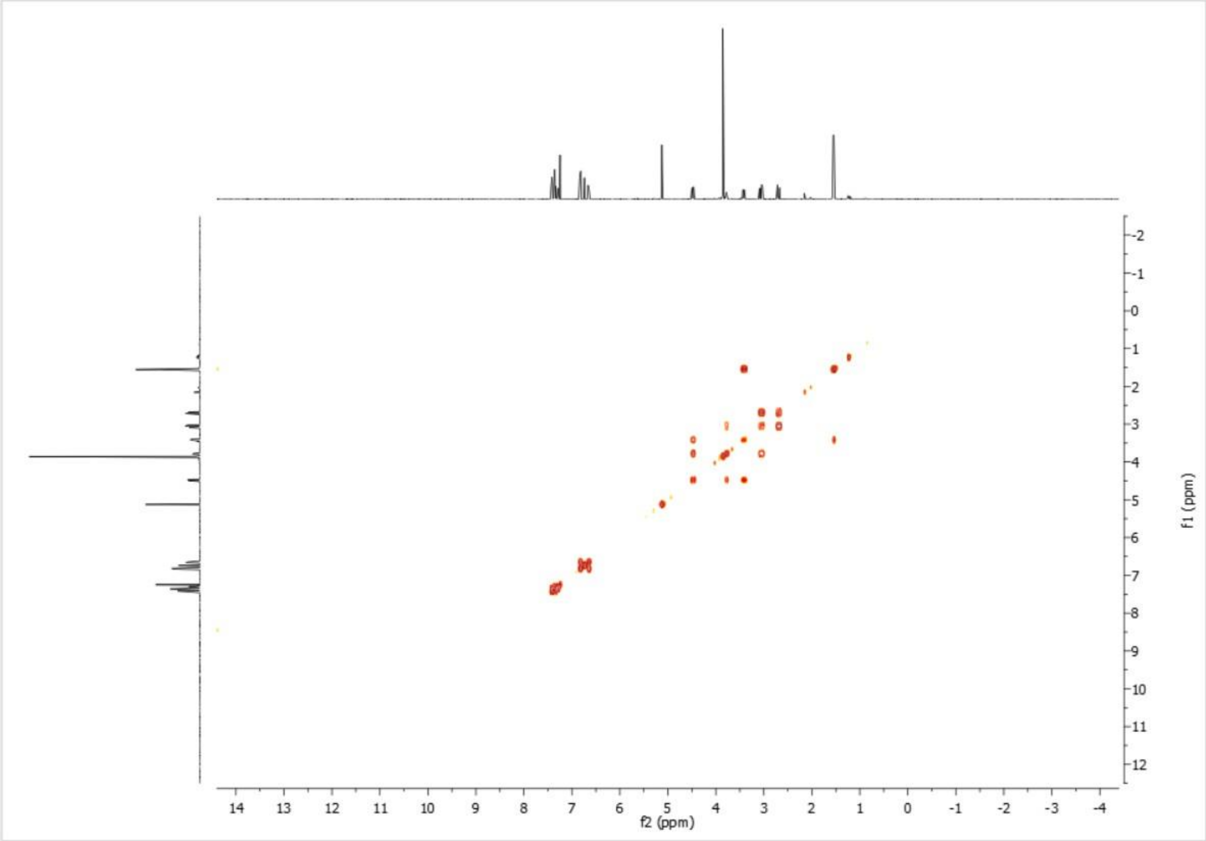


**Figure S64.** COSY spectrum of *cis*-4-(4'-benzyloxy-3'-methoxyphenyl)-5-(1-chloroethyl)dihydrofuran-2-one **(9a)**

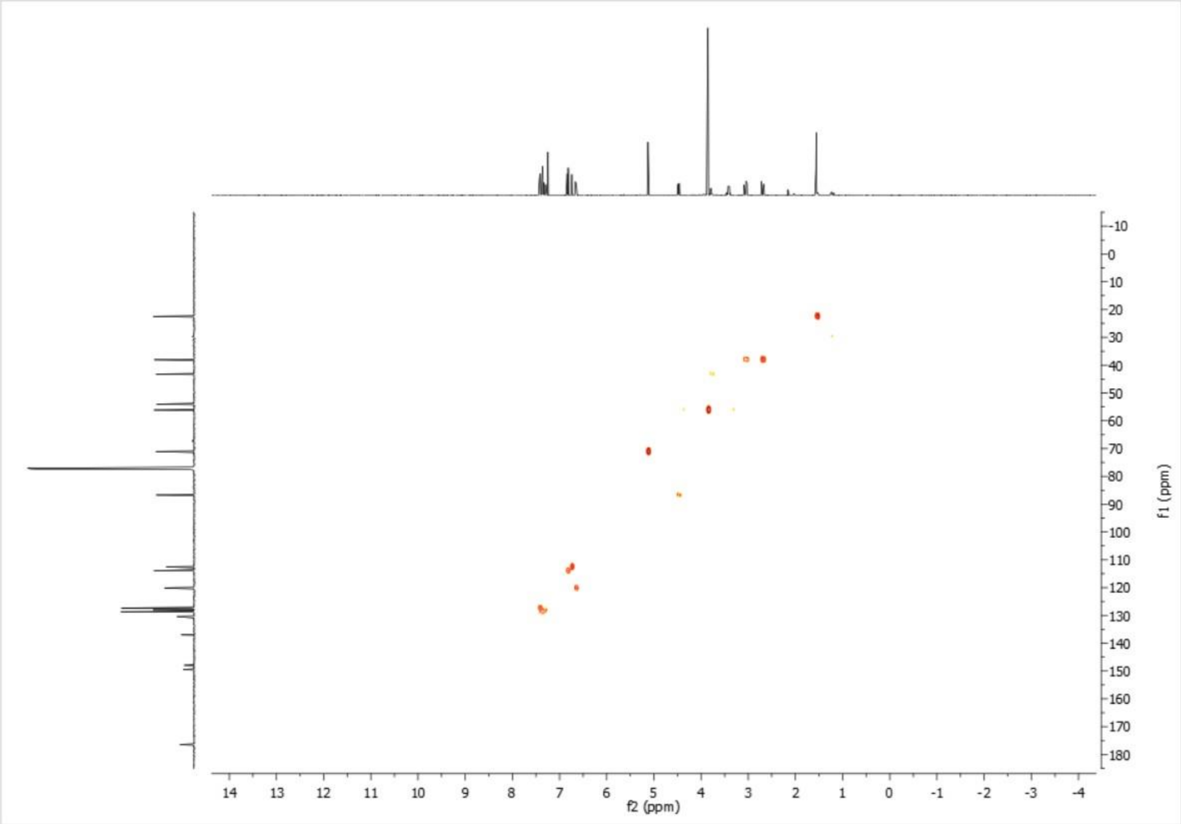


**Figure S65.** HMQC spectrum of *cis*-4-(4'-benzyloxy-3'-methoxyphenyl)-5-(1-chloroethyl)dihydrofuran-2-one **(9a)**

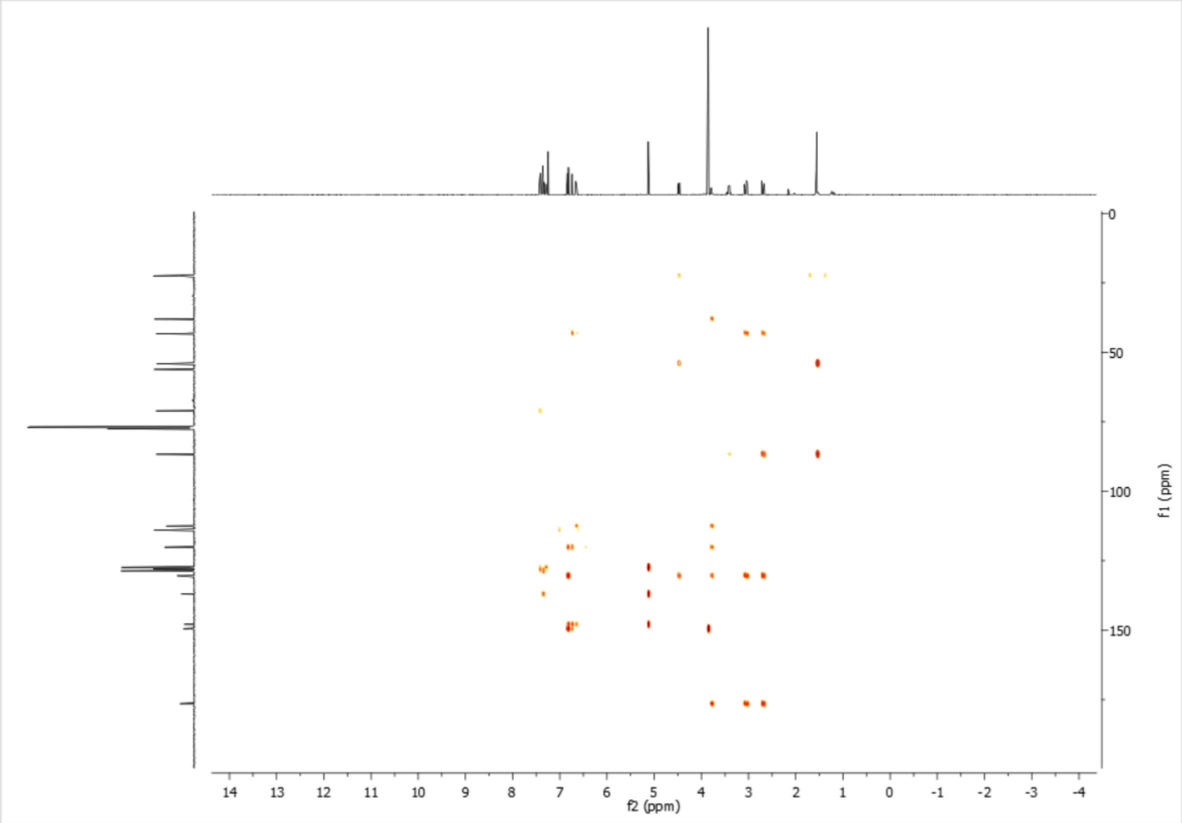


**Figure S66.** HMBC spectrum of *cis*-4-(4'-benzyloxy-3'-methoxyphenyl)-5-(1-chloroethyl)dihydrofuran-2-one **(9a)**

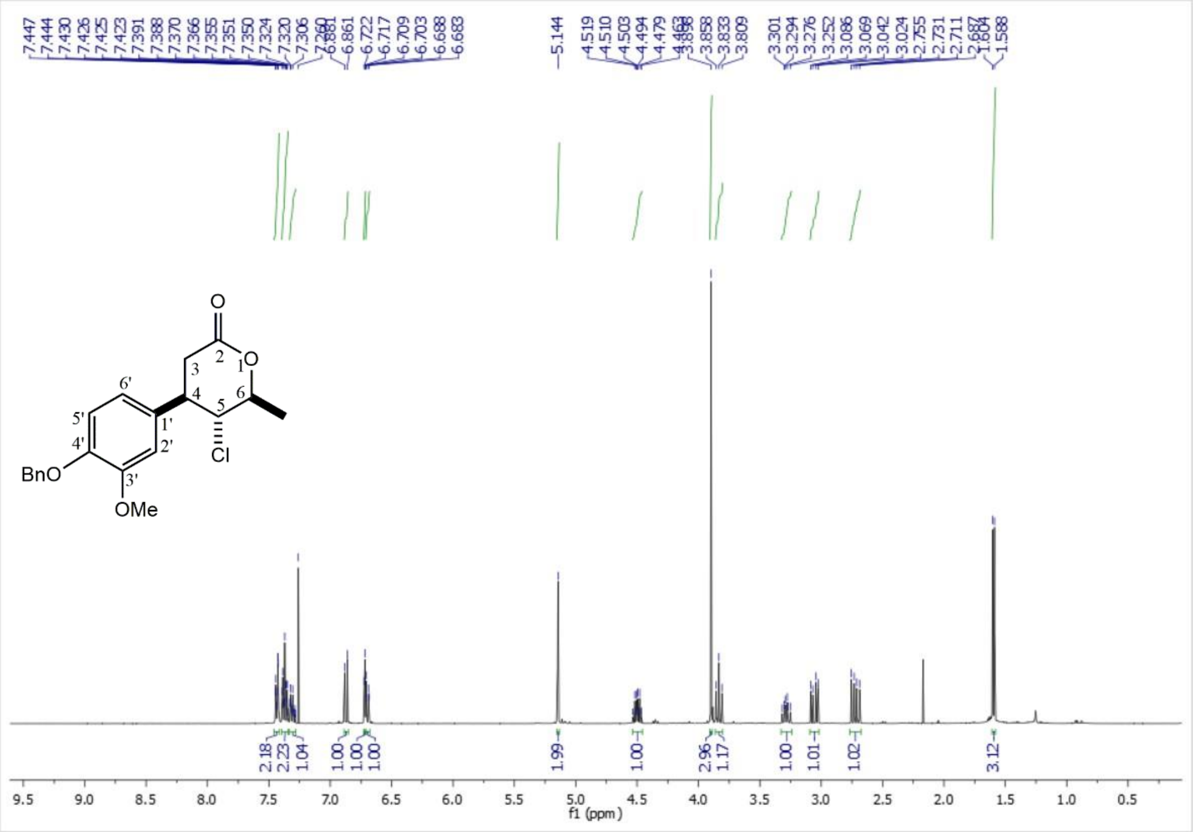

**Figure S67.** $^{1}$H NMR spectrum of 4-*r*-(4'-benzyloxy-3'-methoxyphenyl)-5-*t*-chloro-6-*c*-methyltetrahydropyran-2-one **(9b)**

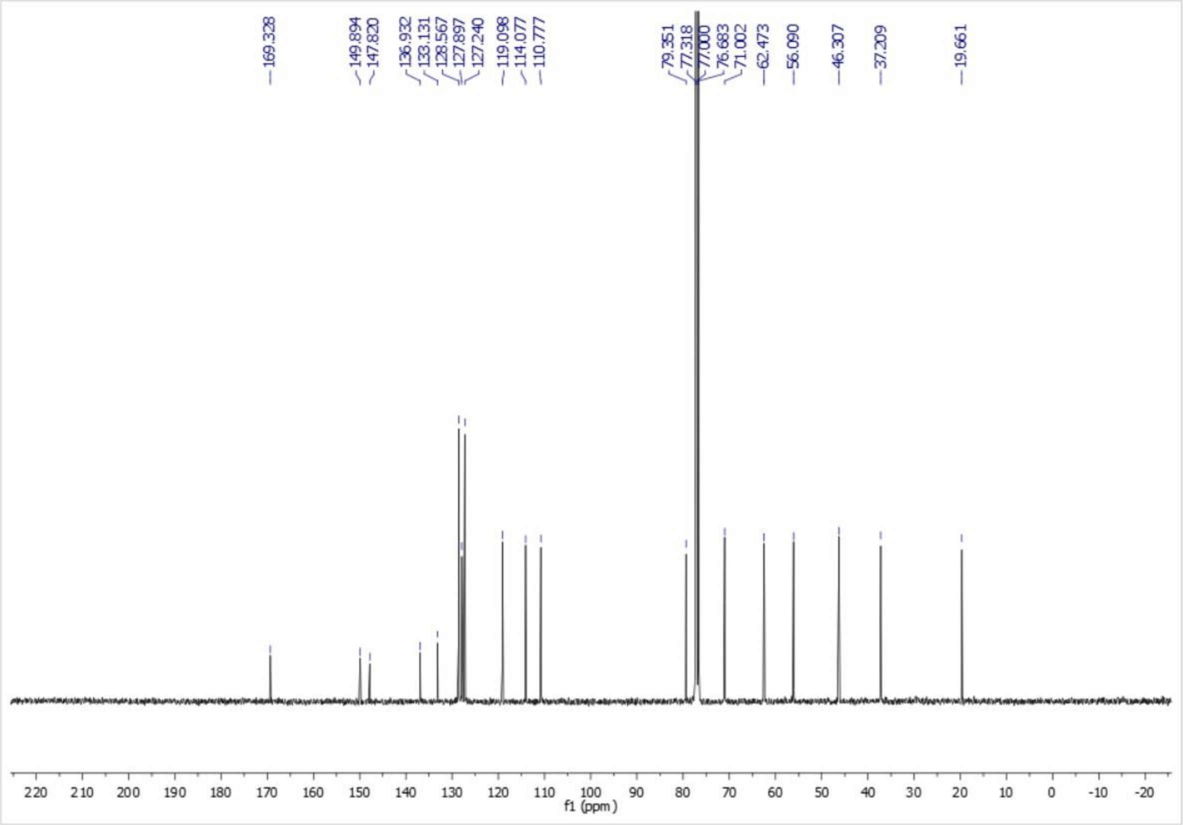


**Figure S68.** $^{13}$CNMR spectrum of 4-*r*-(4'-benzyloxy-3'-methoxyphenyl)-5-*t*-chloro-6-*c*-methyltetrahydropyran-2-one **(9b)**

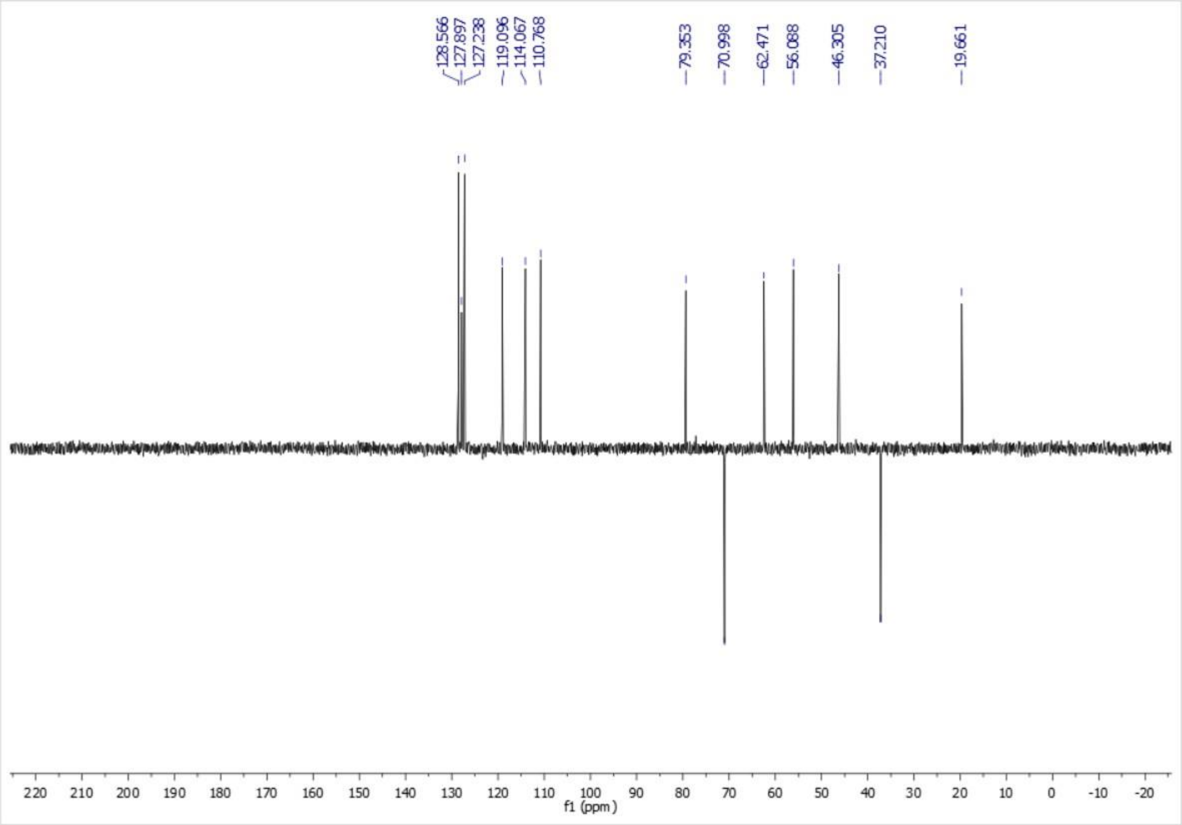


**Figure S69.** DEPT 135 NMR spectrum of 4-*r*-(4'-benzyloxy-3'-methoxyphenyl)-5-*t*-chloro-6-*c*-methyltetrahydropyran-2-one **(9b)**

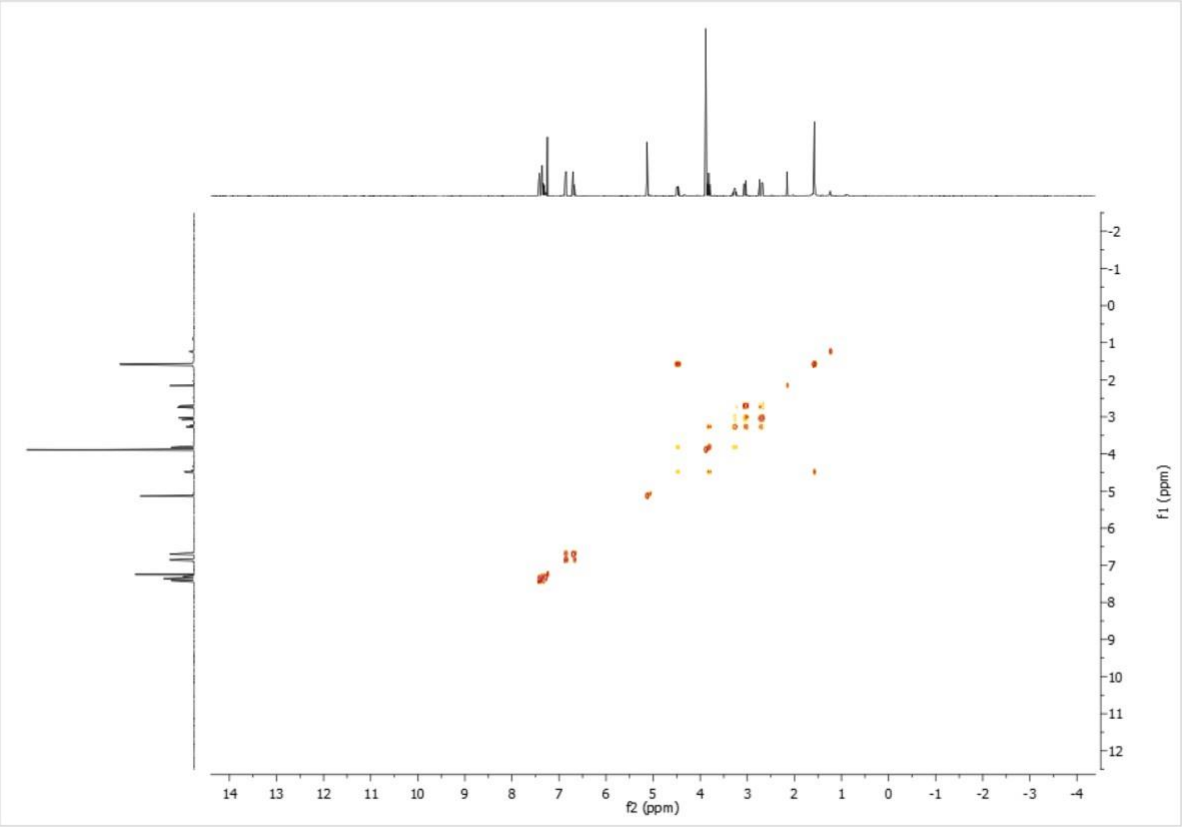


**Figure S70.** COSY spectrum of 4-*r*-(4'-benzyloxy-3'-methoxyphenyl)-5-*t*-chloro-6-*c*-methyltetrahydropyran-2-one **(9b)**

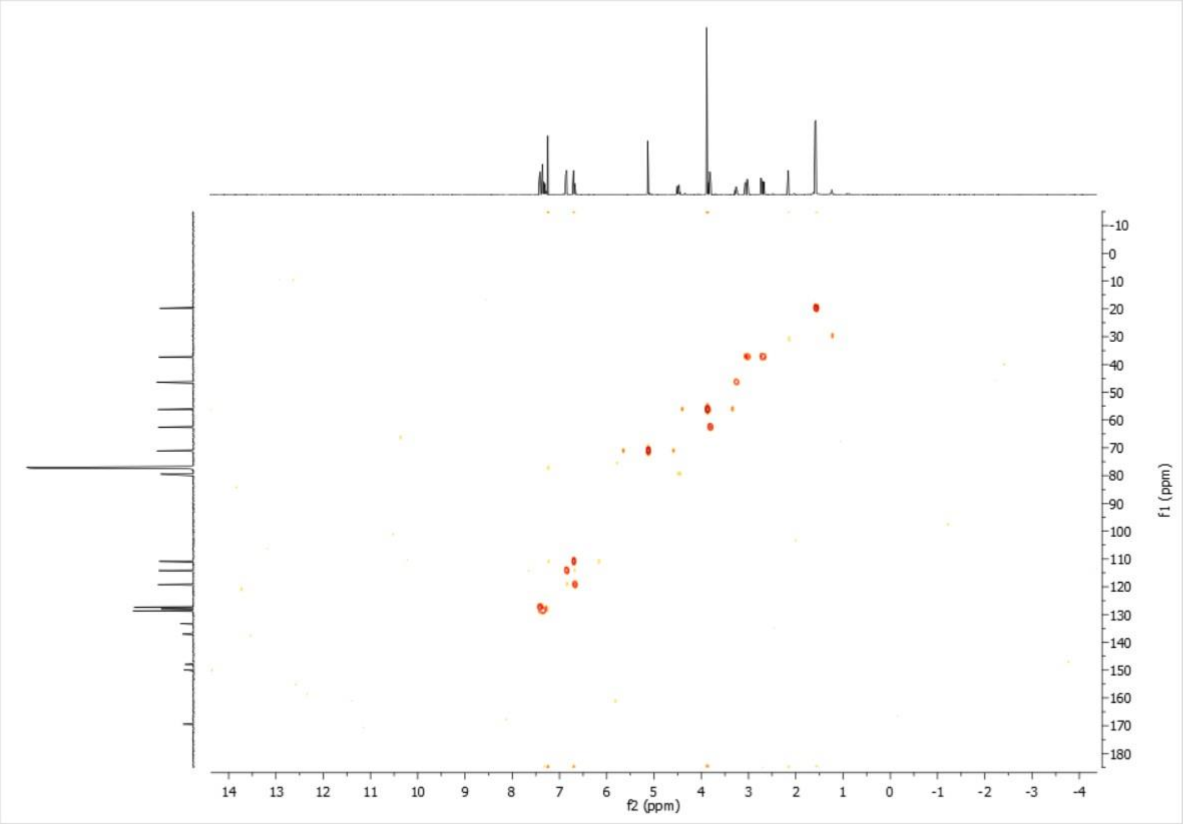


**Figure S71.** HMQC spectrum of 4-*r*-(4'-benzyloxy-3'-methoxyphenyl)-5-*t*-chloro-6-*c*-methyltetrahydropyran-2-one **(9b)**

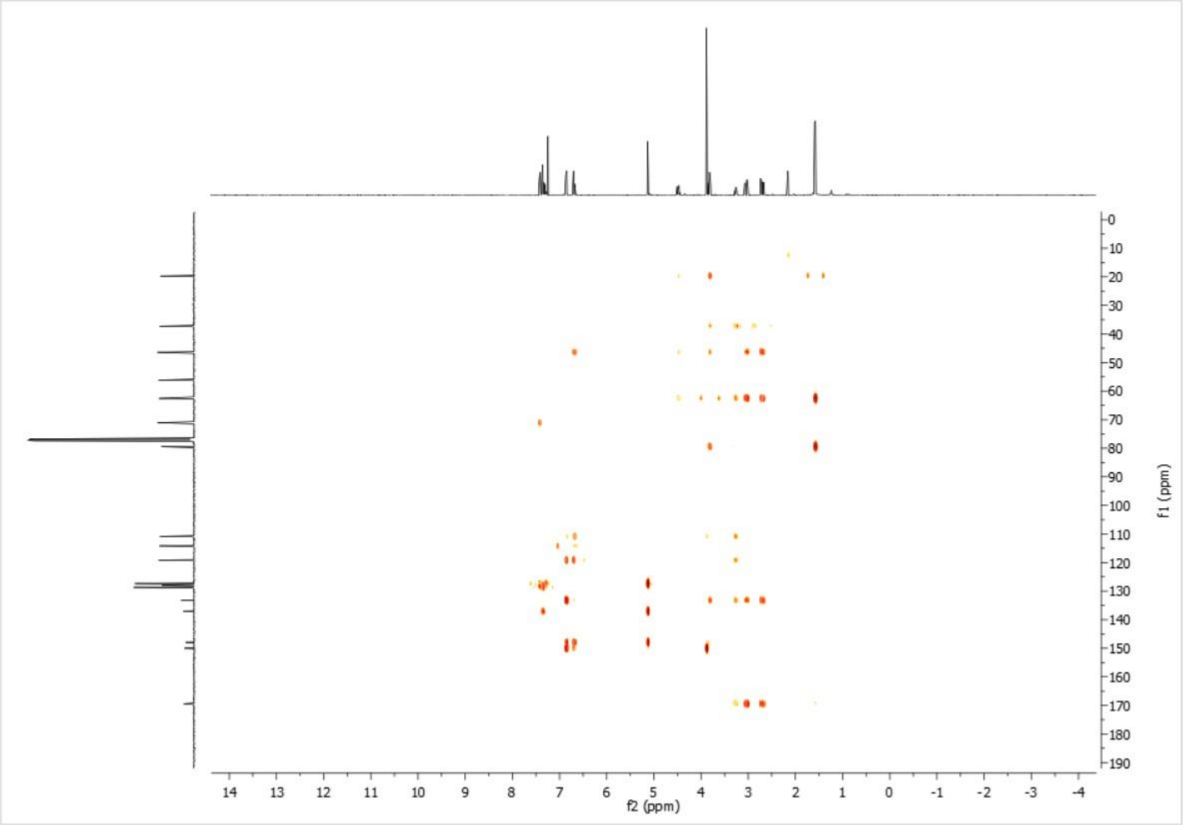


**Figure S72.** HMBC spectrum of 4-*r*-(4'-benzyloxy-3'-methoxyphenyl)-5-*t*-chloro-6-*c*-methyltetrahydropyran-2-one **(9b)**

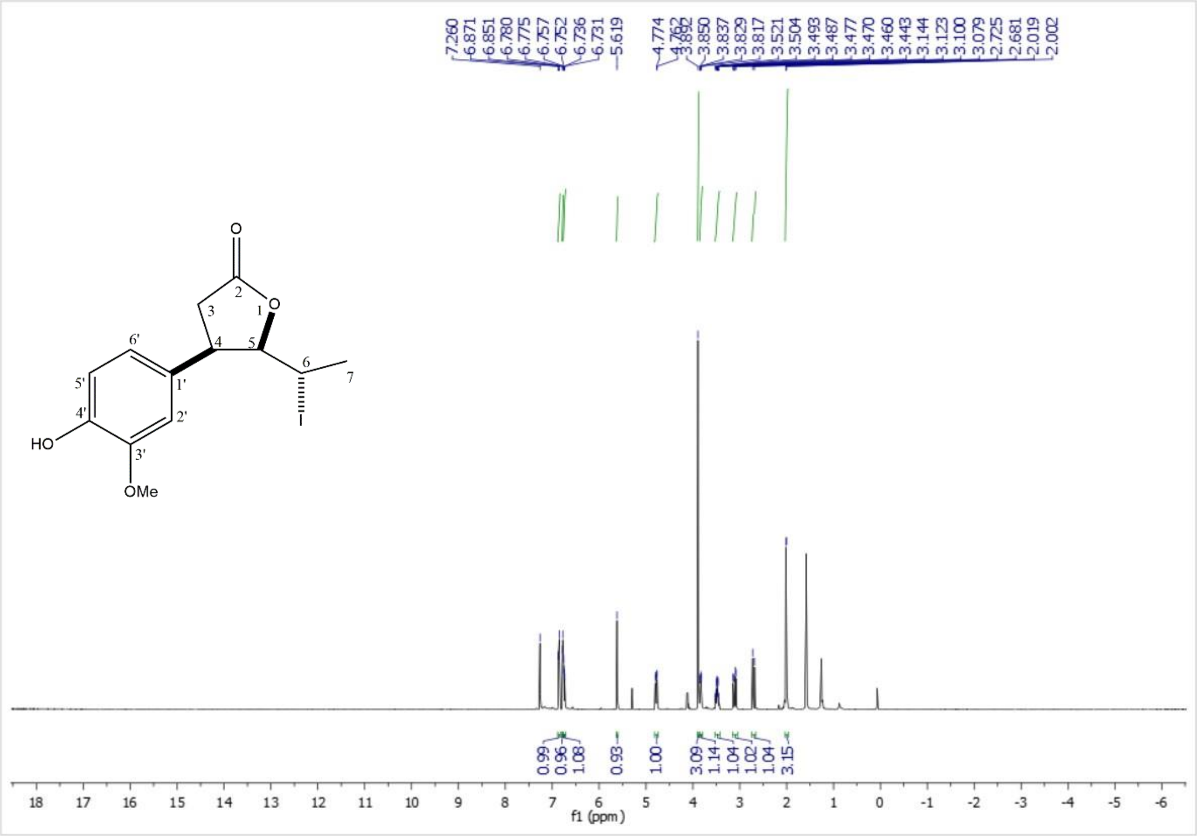


**Figure S73.** $^{1}$H NMR spectrum of *cis*-4-(4'-hydroxy-3'-methoxyphenyl)-5-(1-iodoethyl)dihydrofuran-2-one **(10a)**

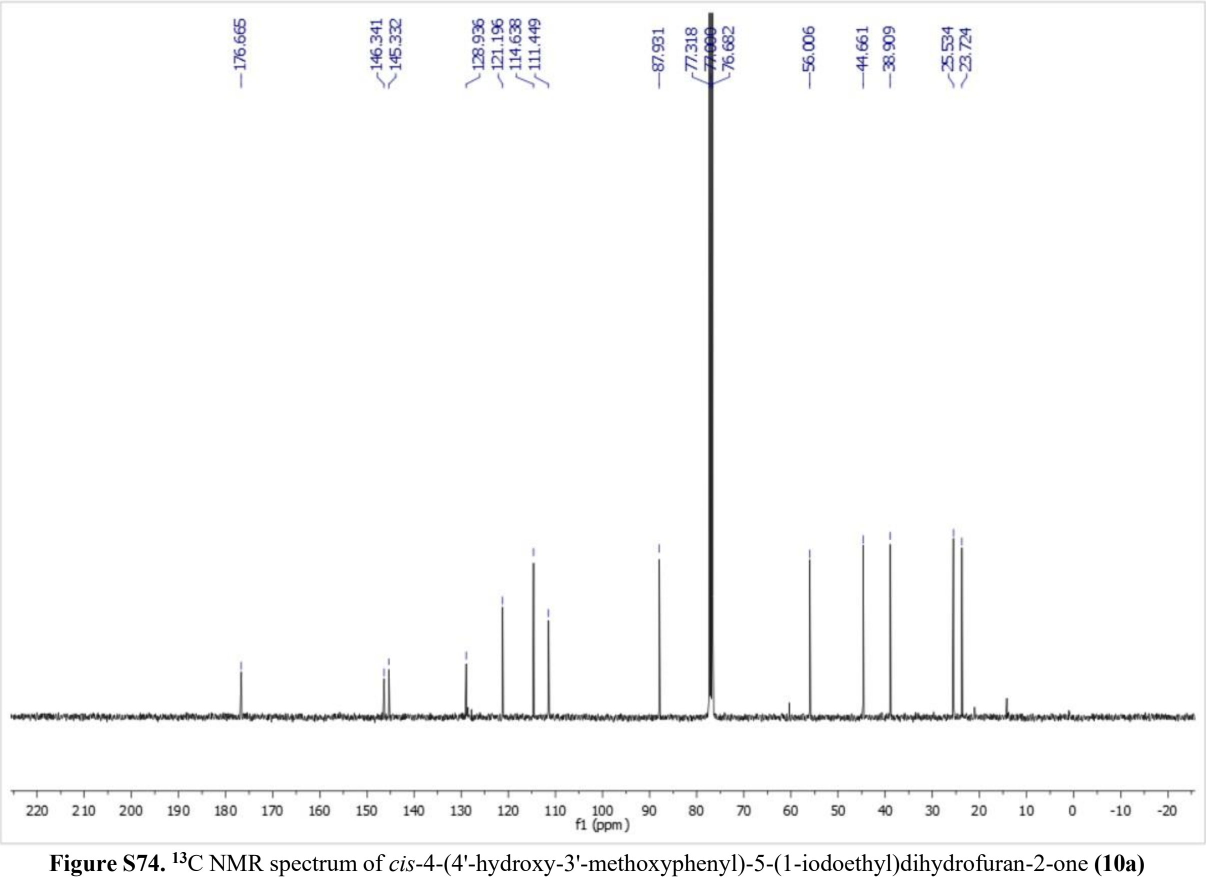


**Figure S74.** $^{13}$C NMR spectrum of *cis*-4-(4'-hydroxy-3'-methoxyphenyl)-5-(1-iodoethyl)dihydrofuran-2-one **(10a)**

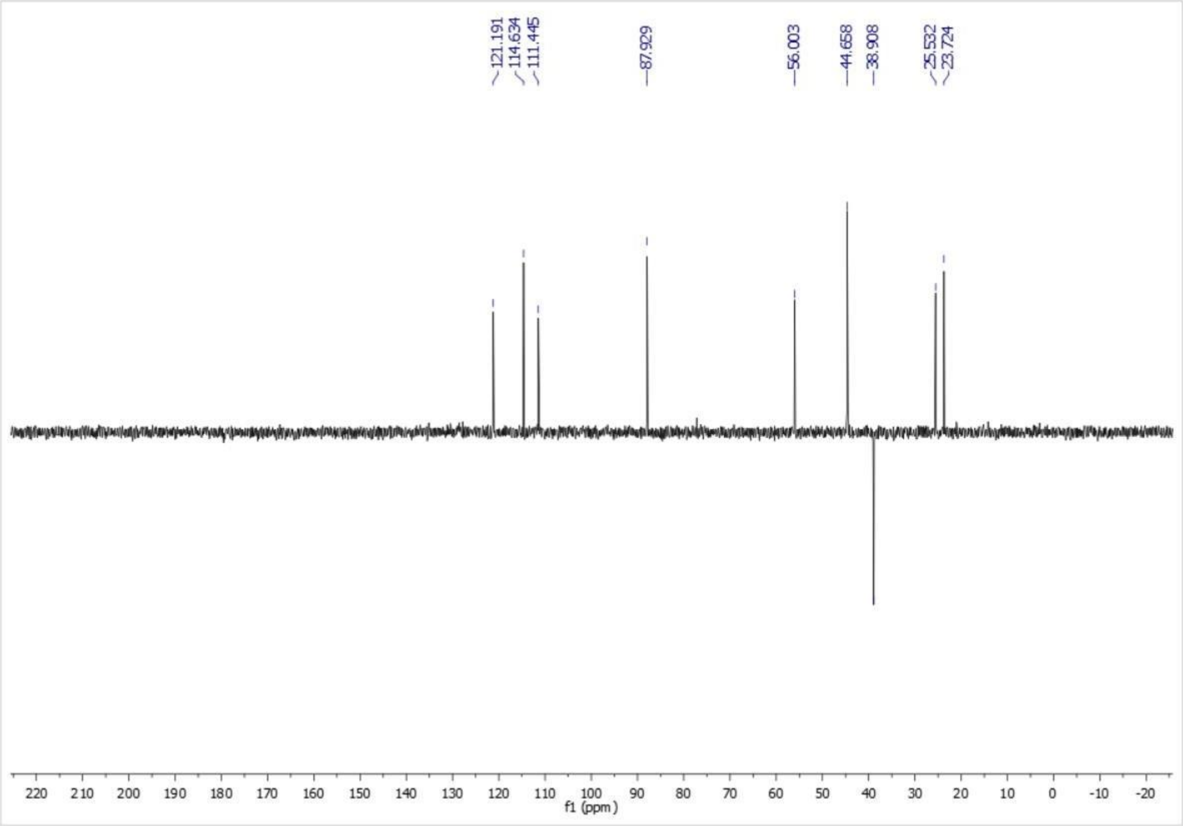


**Figure S75.** DEPT 135 NMR spectrum of *cis*-4-(4'-hydroxy-3'-methoxyphenyl)-5-(1-iodoethyl)dihydrofuran-2-one **(10a)**

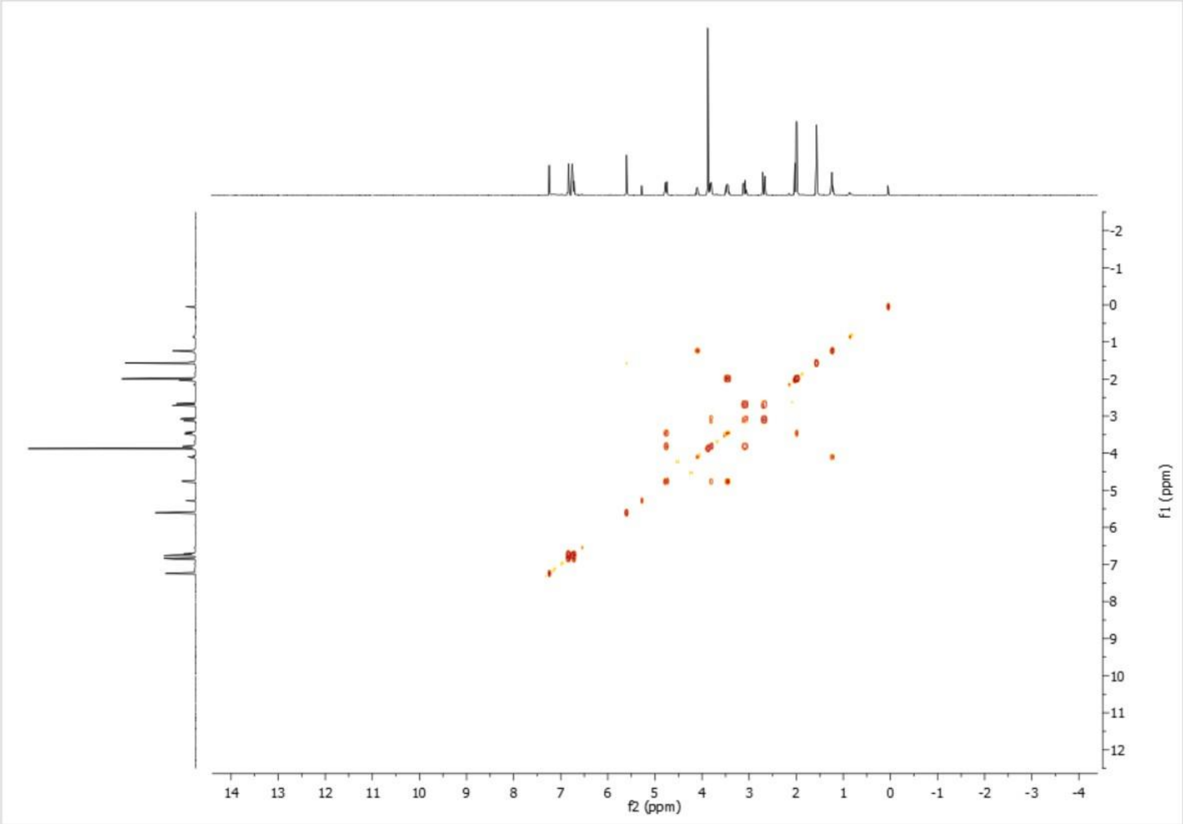


**Figure S76.** COSY spectrum of *cis*-4-(4'-hydroxy-3'-methoxyphenyl)-5-(1-iodoethyl)dihydrofuran-2-one **(10a)**

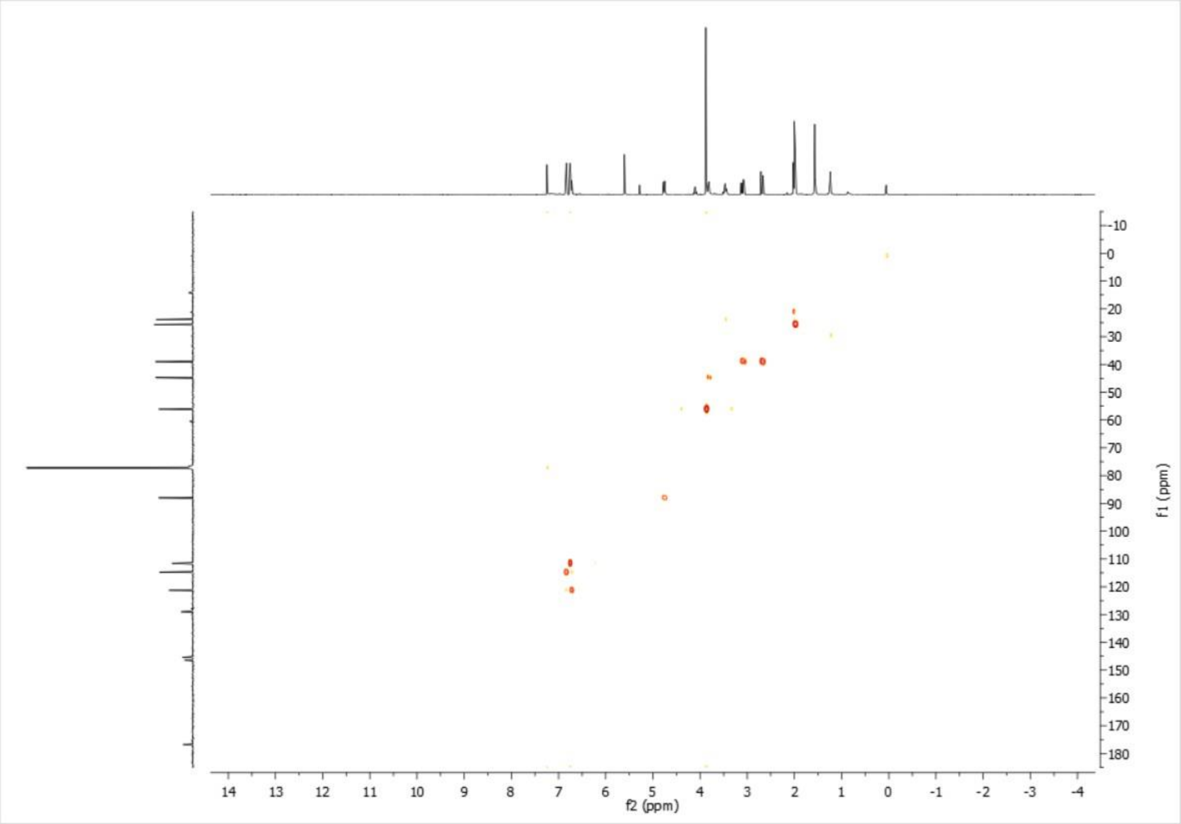


**Figure S77.** HMQC spectrum of *cis*-4-(4'-hydroxy-3'-methoxyphenyl)-5-(1-iodoethyl)dihydrofuran-2-one **(10a)**

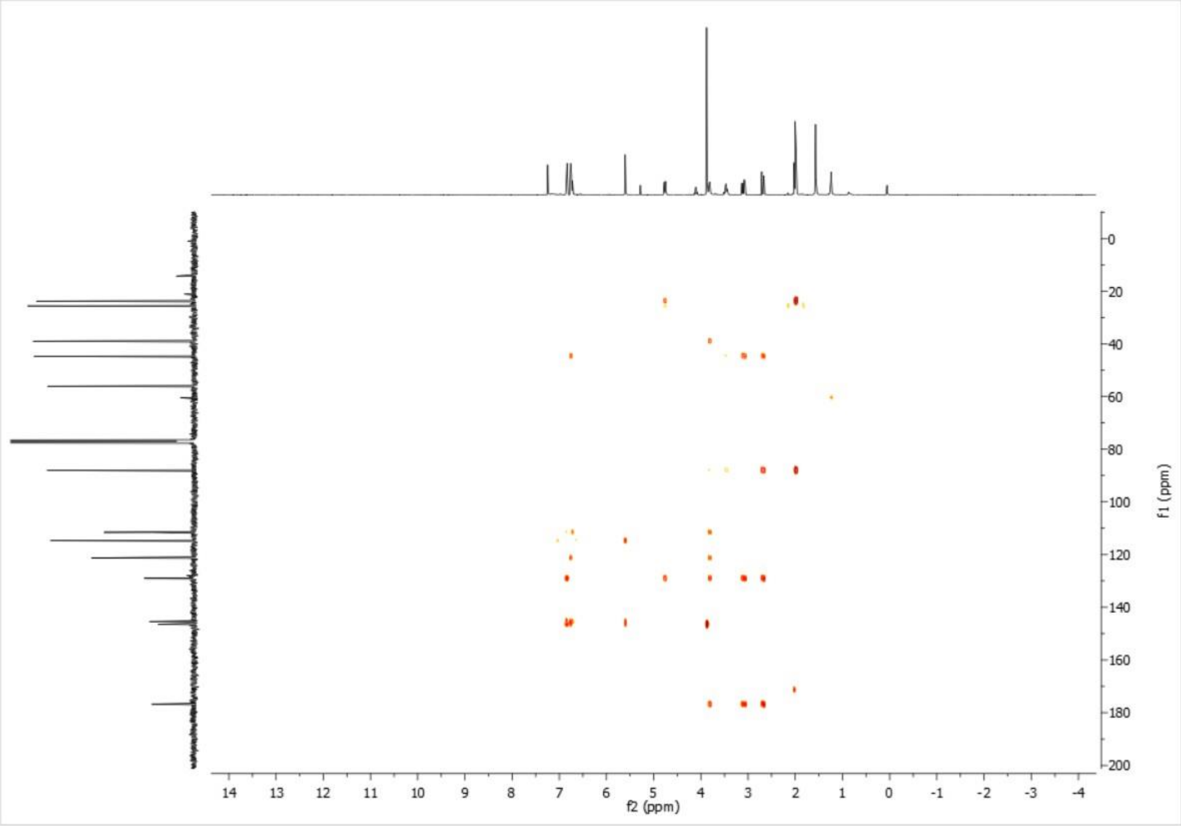


**Figure S78.** HMBC spectrum of *cis*-4-(4'-hydroxy-3'-methoxyphenyl)-5-(1-iodoethyl)dihydrofuran-2-one **(10a)**

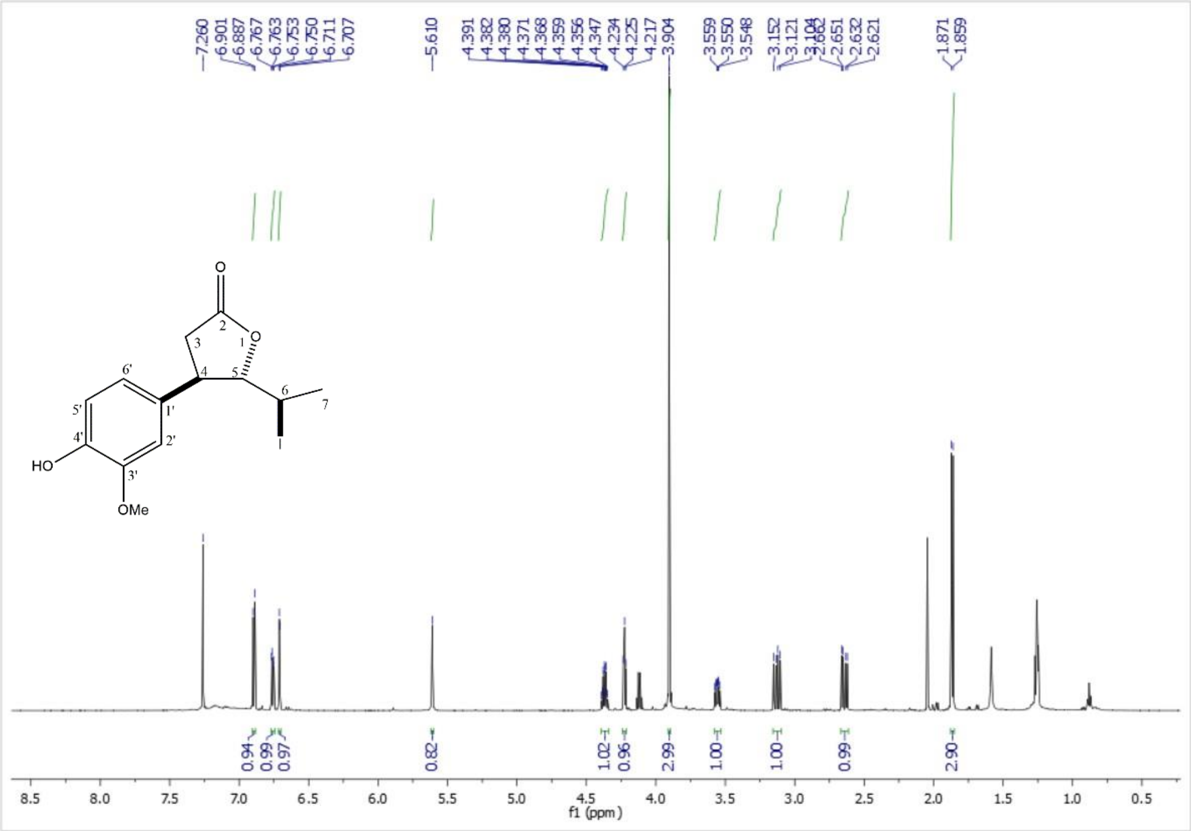


**Figure S79.** $^{1}$H NMR spectrum of *trans*-4-(4'-hydroxy-3'-methoxyphenyl)-5-(1-iodoethyl)dihydrofuran-2-one **(10b)**

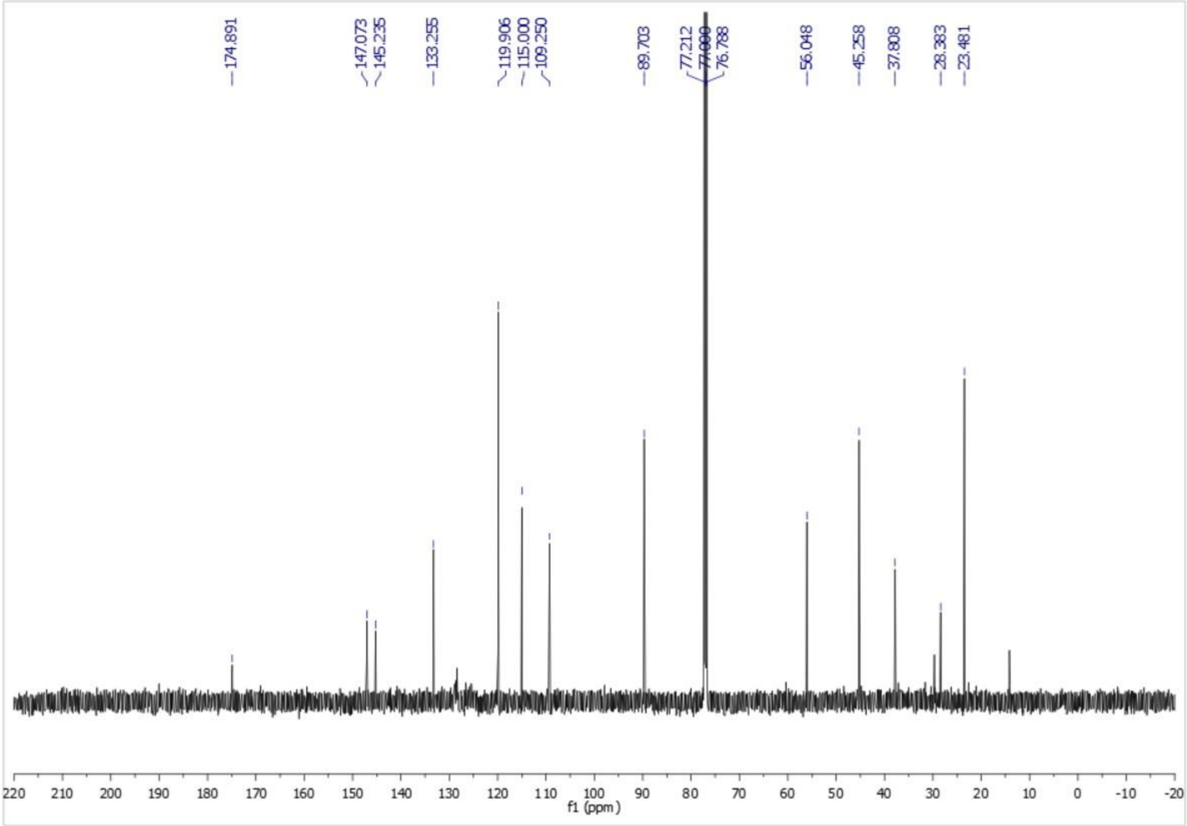


**Figure S80.** $^{13}$C NMR spectrum of *trans*-4-(4'-hydroxy-3'-methoxyphenyl)-5-(1-iodoethyl)dihydrofuran-2-one **(10b)**

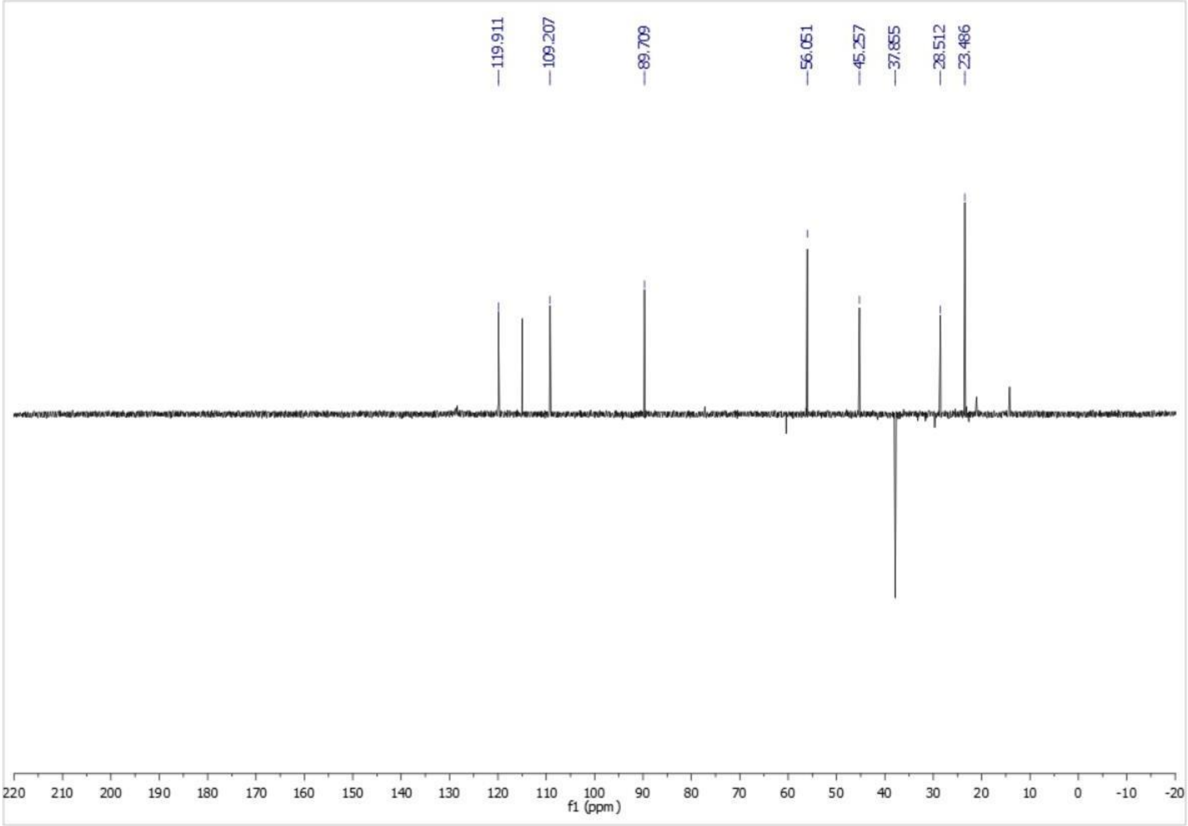


**Figure S81.** DEPT 135 NMR spectrum of *trans*-4-(4'-hydroxy-3'-methoxyphenyl)-5-(1-iodoethyl)dihydrofuran-2-one **(10b)**

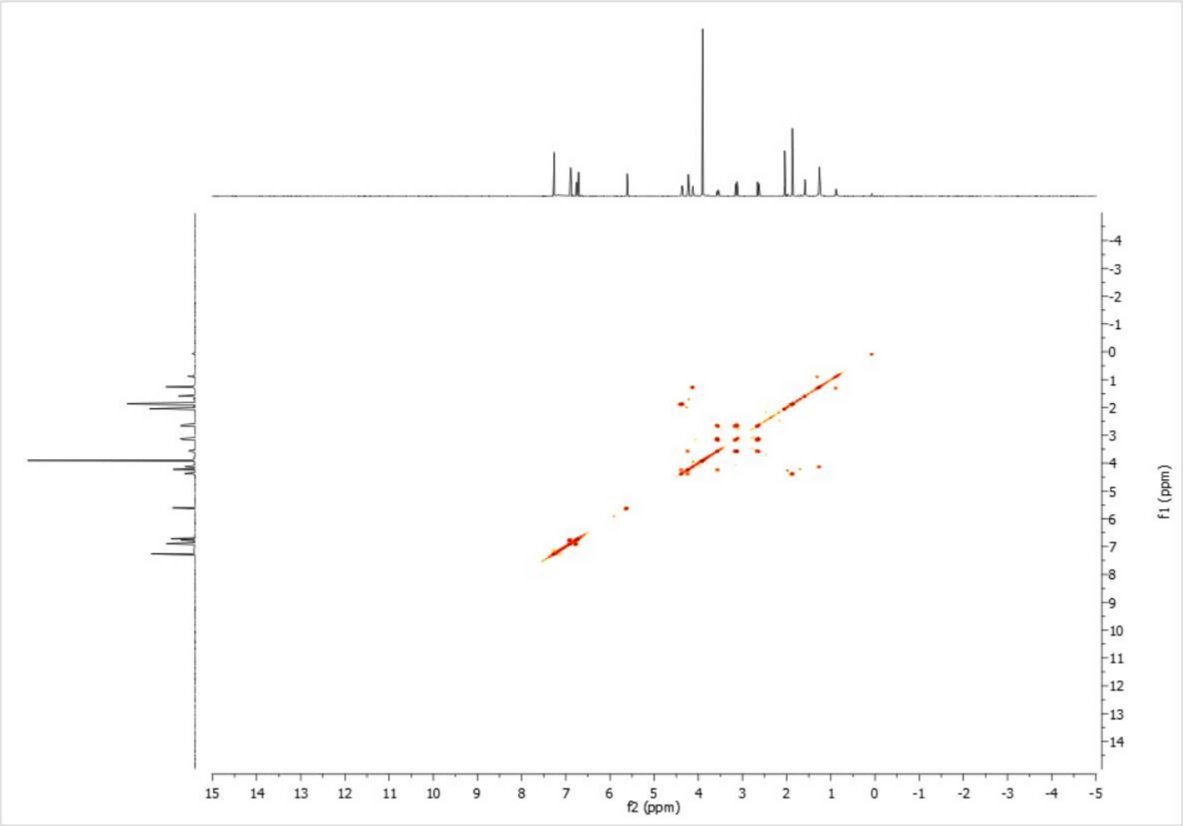


**Figure S82.** COSY spectrum of *trans*-4-(4'-hydroxy-3'-methoxyphenyl)-5-(1-iodoethyl)dihydrofuran-2-one **(10b)**

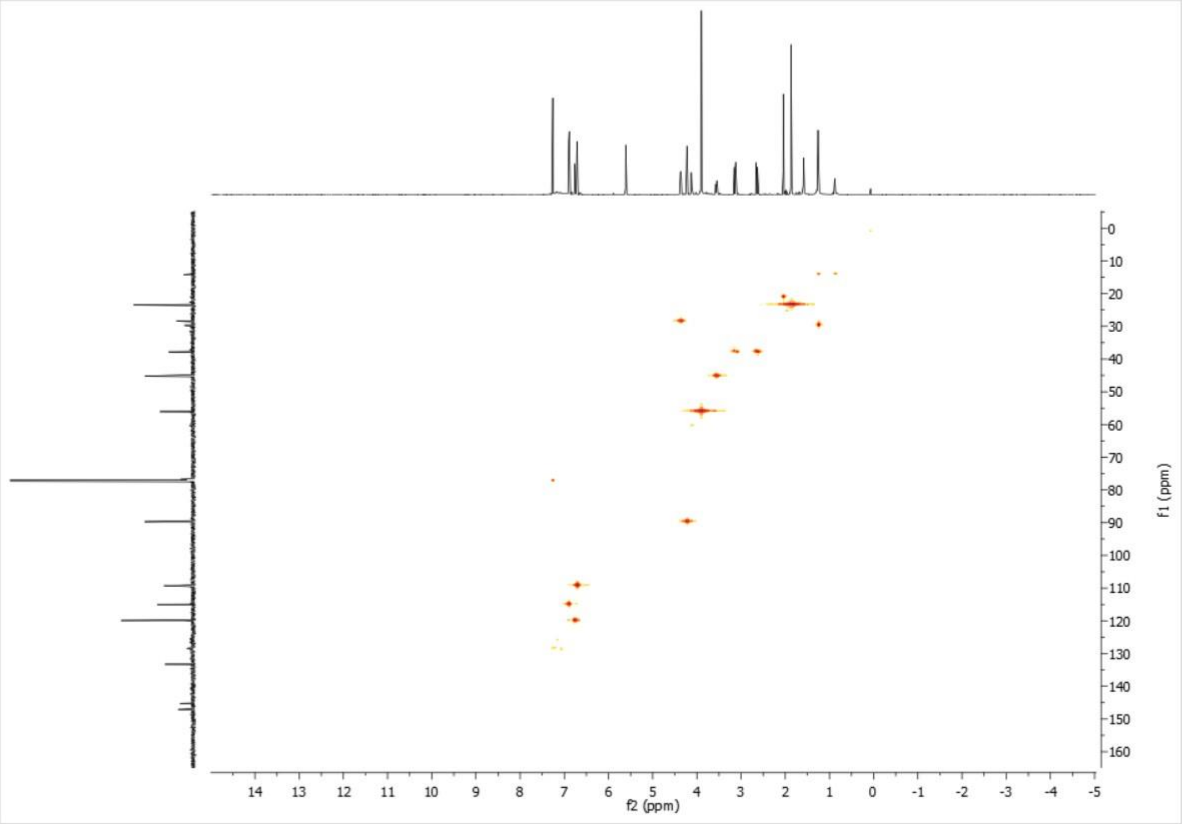


**Figure S83.** HMQC spectrum of *trans*-4-(4'-hydroxy-3'-methoxyphenyl)-5-(1-iodoethyl)dihydrofuran-2-one **(10b)**

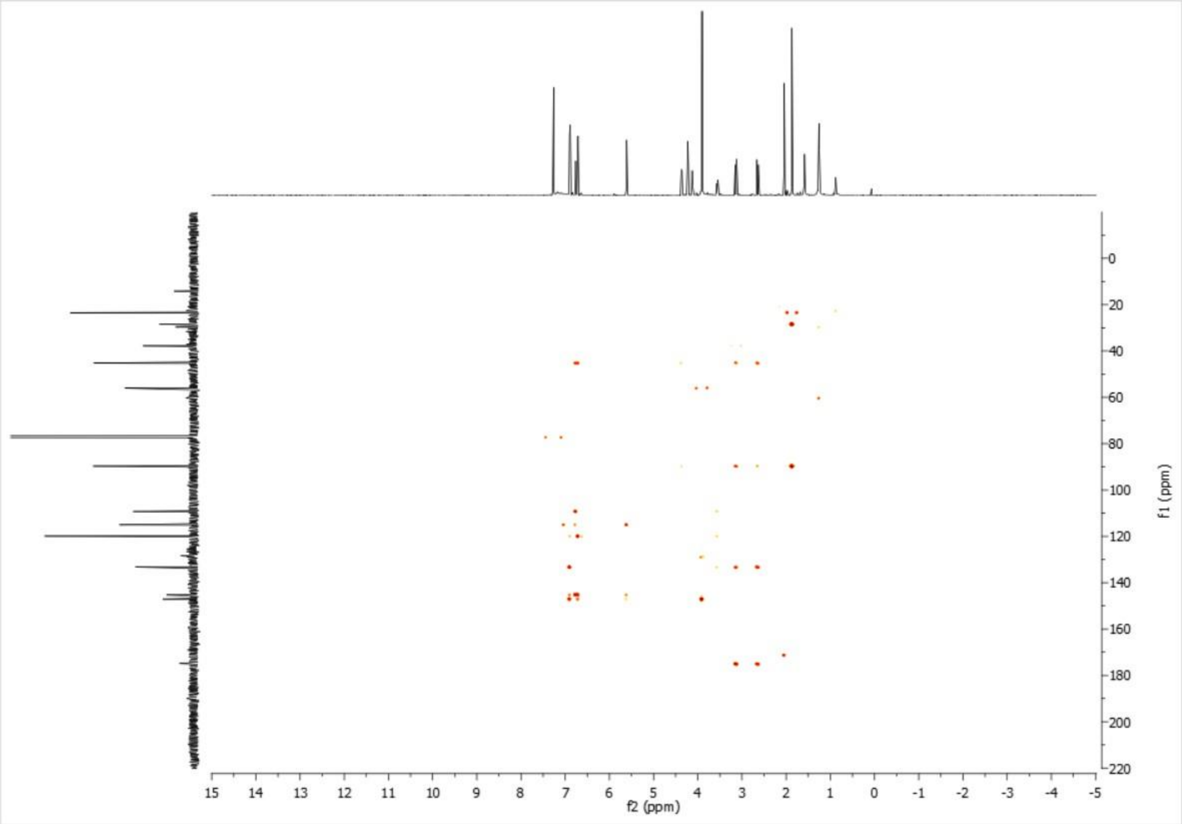


**Figure S84.** HMBC spectrum of *trans*-4-(4'-hydroxy-3'-methoxyphenyl)-5-(1-iodoethyl)dihydrofuran-2-one **(10b)**

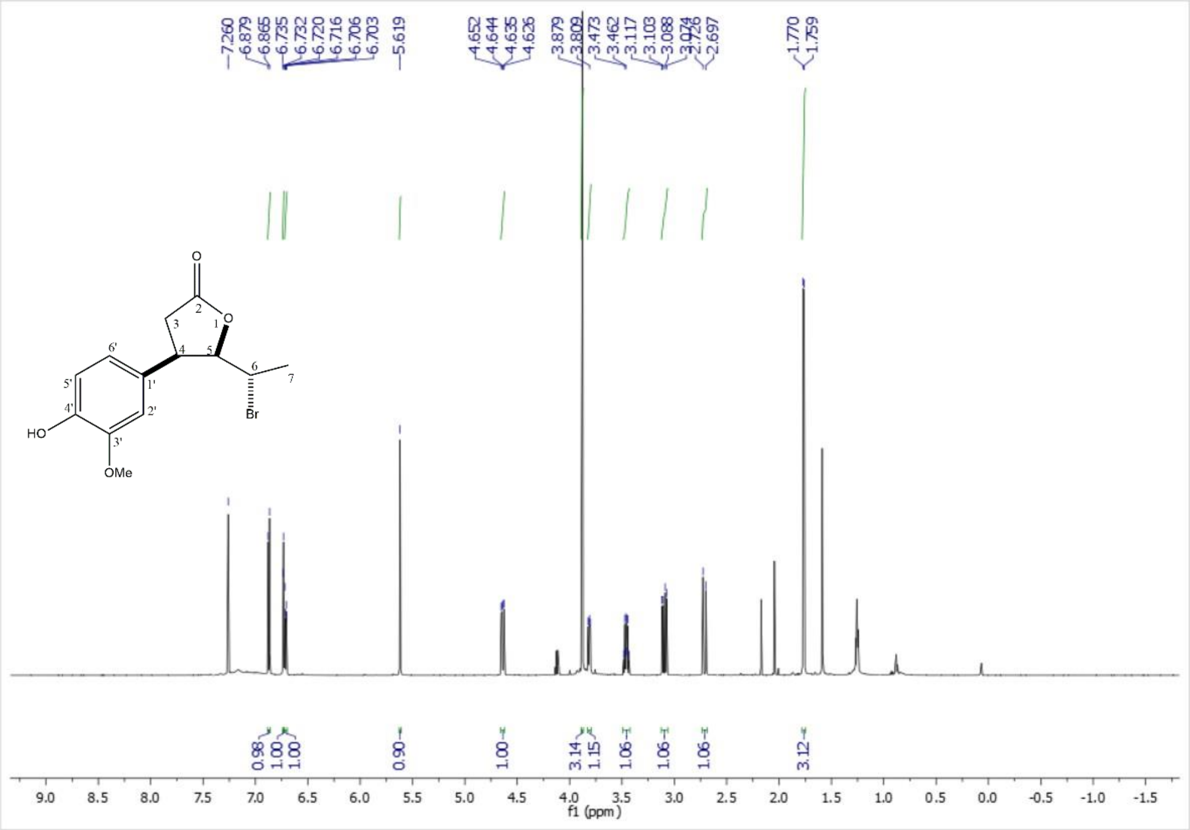


**Figure S85.** $^{1}H$ NMR spectrum of *cis*-5-(1-bromoethyl)-4-(4'-hydroxy-3'-methoxyphenyl)dihydrofuran-2-one **(11a)**

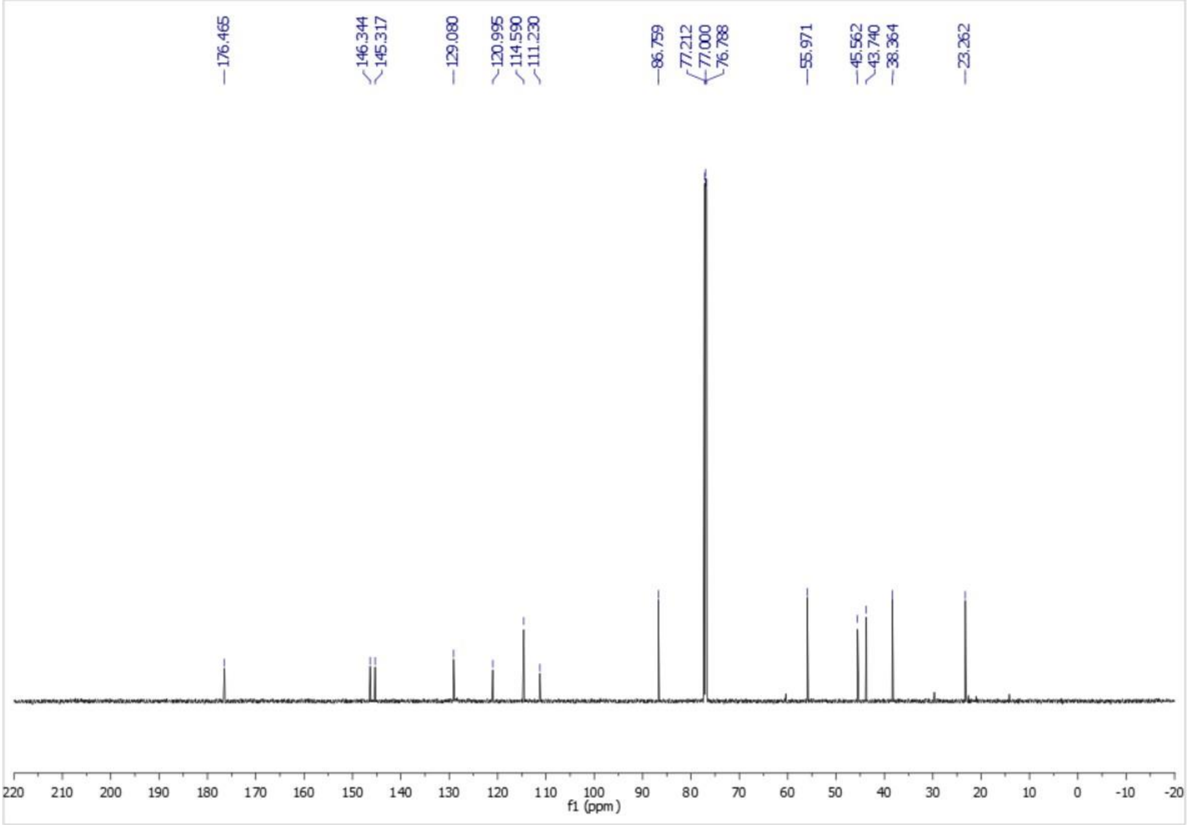


**Figure S86.** $^{13}C$ NMR spectrum of *cis*-5-(1-bromoethyl)-4-(4'-hydroxy-3'-methoxyphenyl)dihydrofuran-2-one **(11a)**

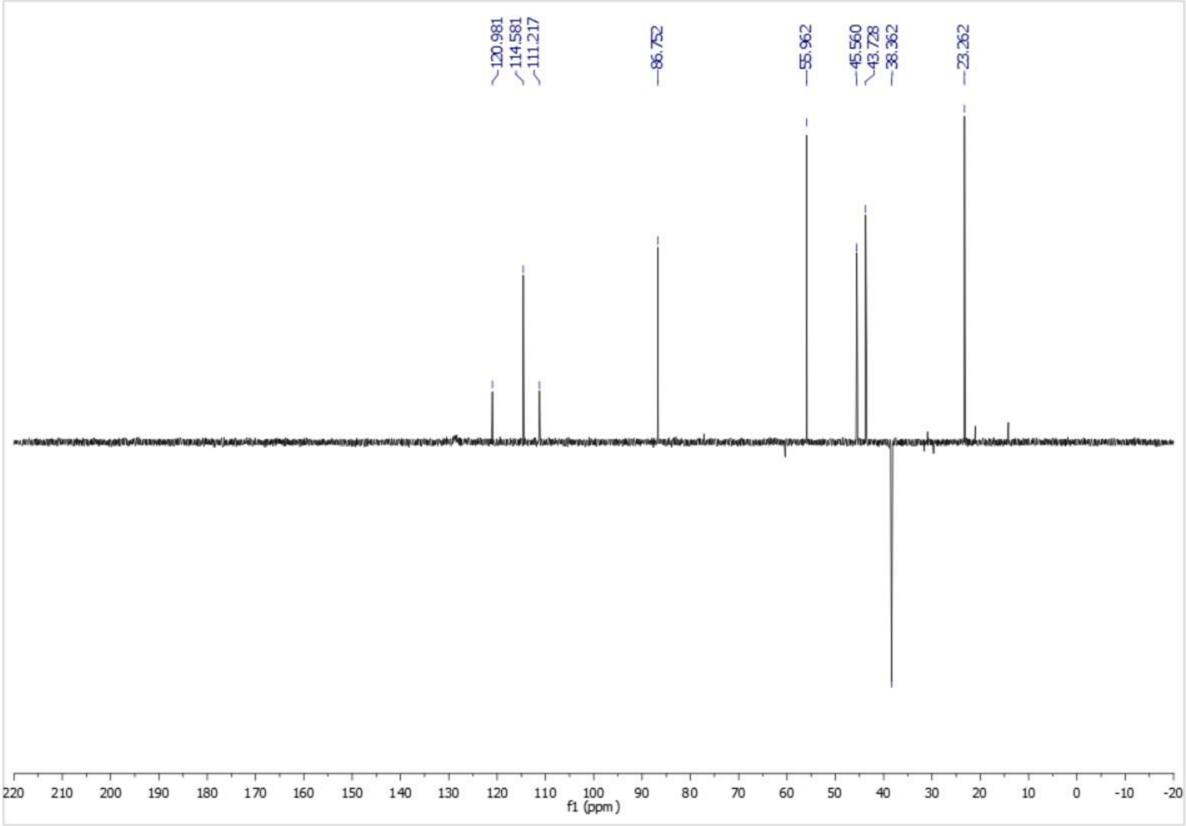


**Figure S87.** DEPT 135 NMR spectrum of *cis*-5-(1-bromoethyl)-4-(4'-hydroxy-3'-methoxyphenyl)dihydrofuran-2-one **(11a)**

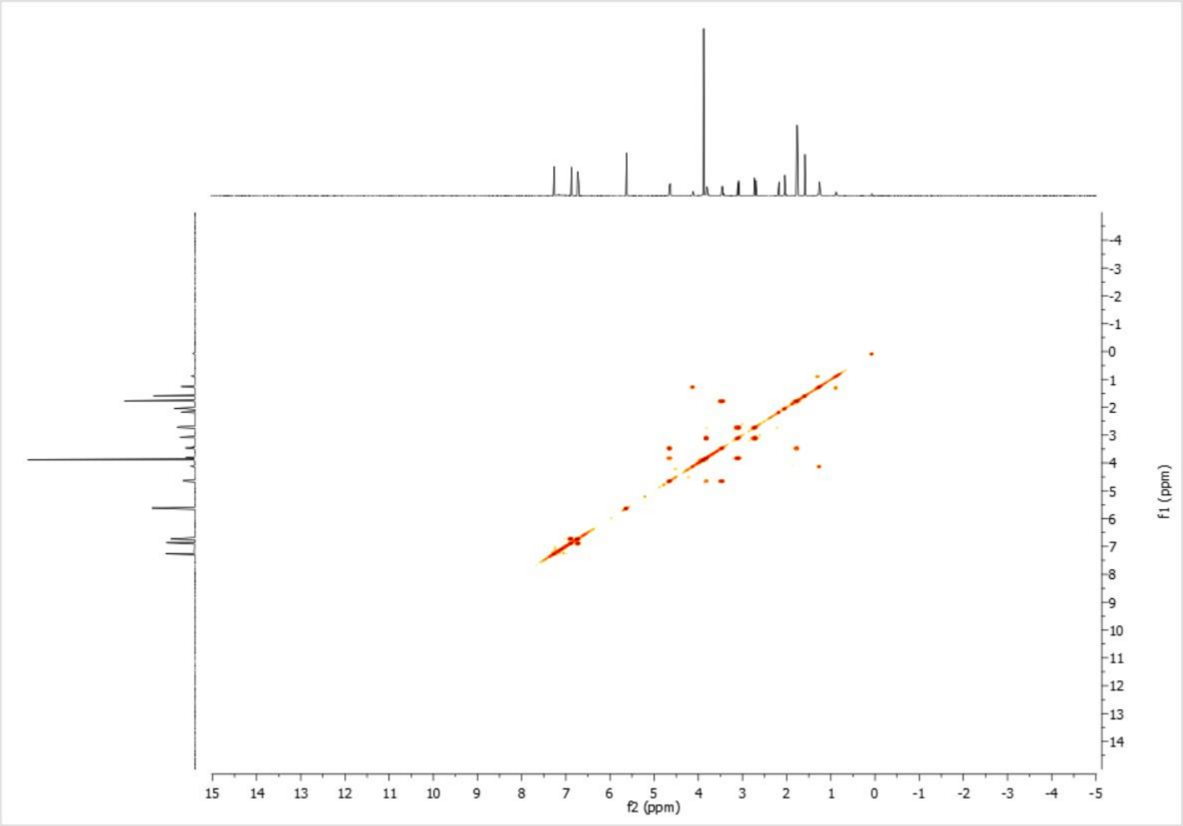


**Figure S88.** COSY spectrum of *cis*-5-(1-bromoethyl)-4-(4'-hydroxy-3'-methoxyphenyl)dihydrofuran-2-one **(11a)**

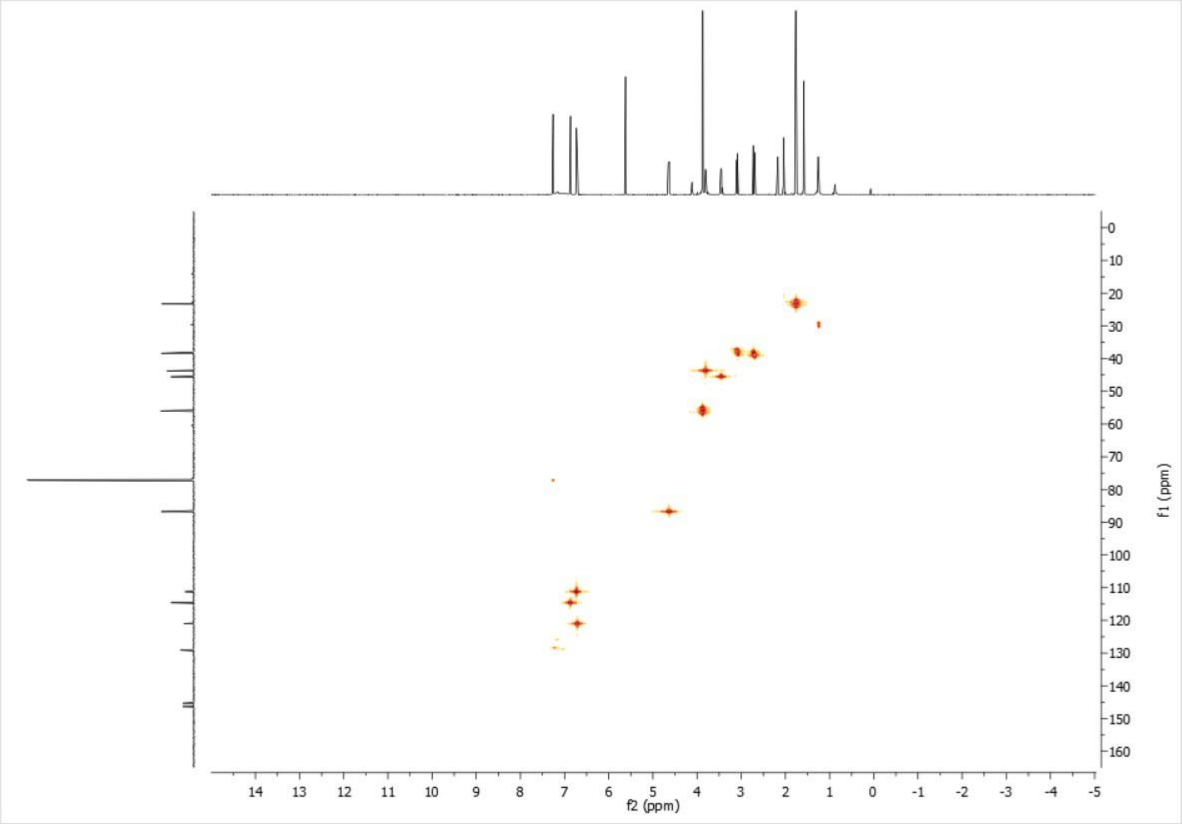


**Figure S89.** HMQC spectrum of *cis*-5-(1-bromoethyl)-4-(4'-hydroxy-3'-methoxyphenyl)dihydrofuran-2-one **(11a)**

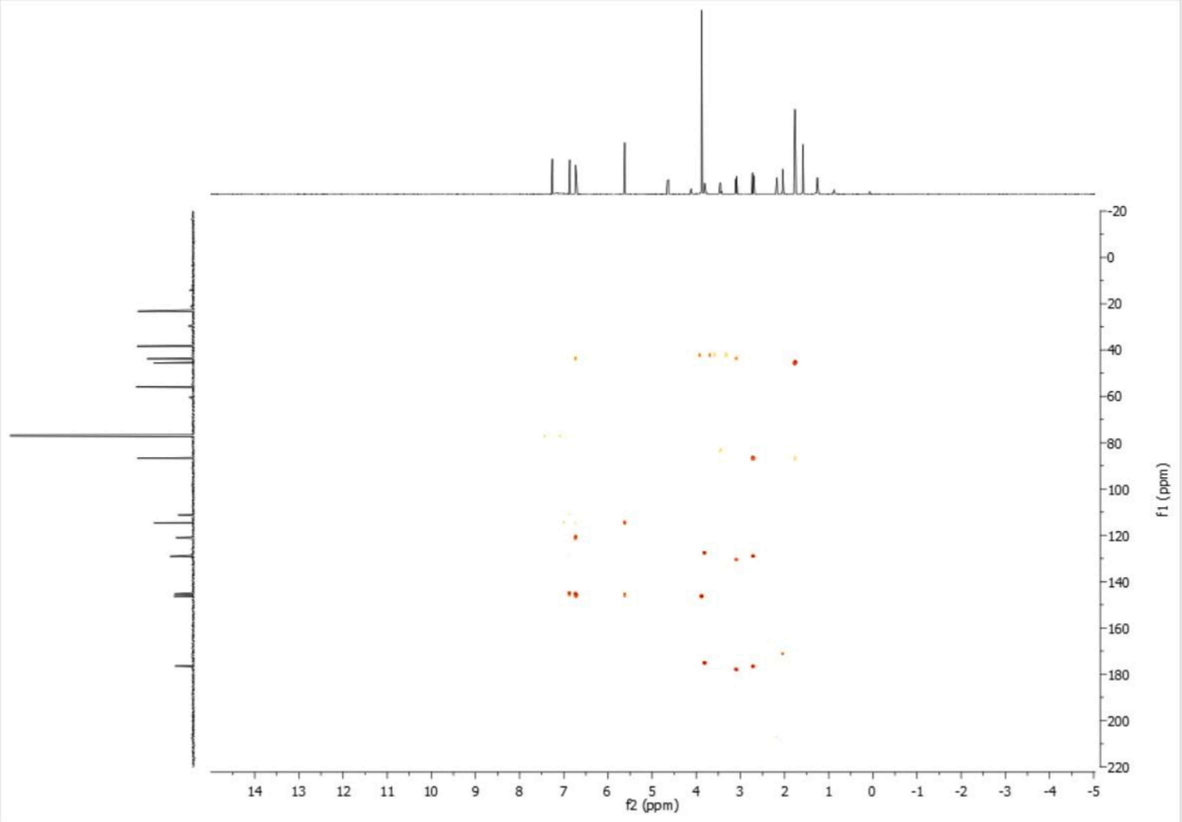


**Figure S90.** HMBC spectrum of *cis*-5-(1-bromoethyl)-4-(4'-hydroxy-3'-methoxyphenyl)dihydrofuran-2-one **(11a)**

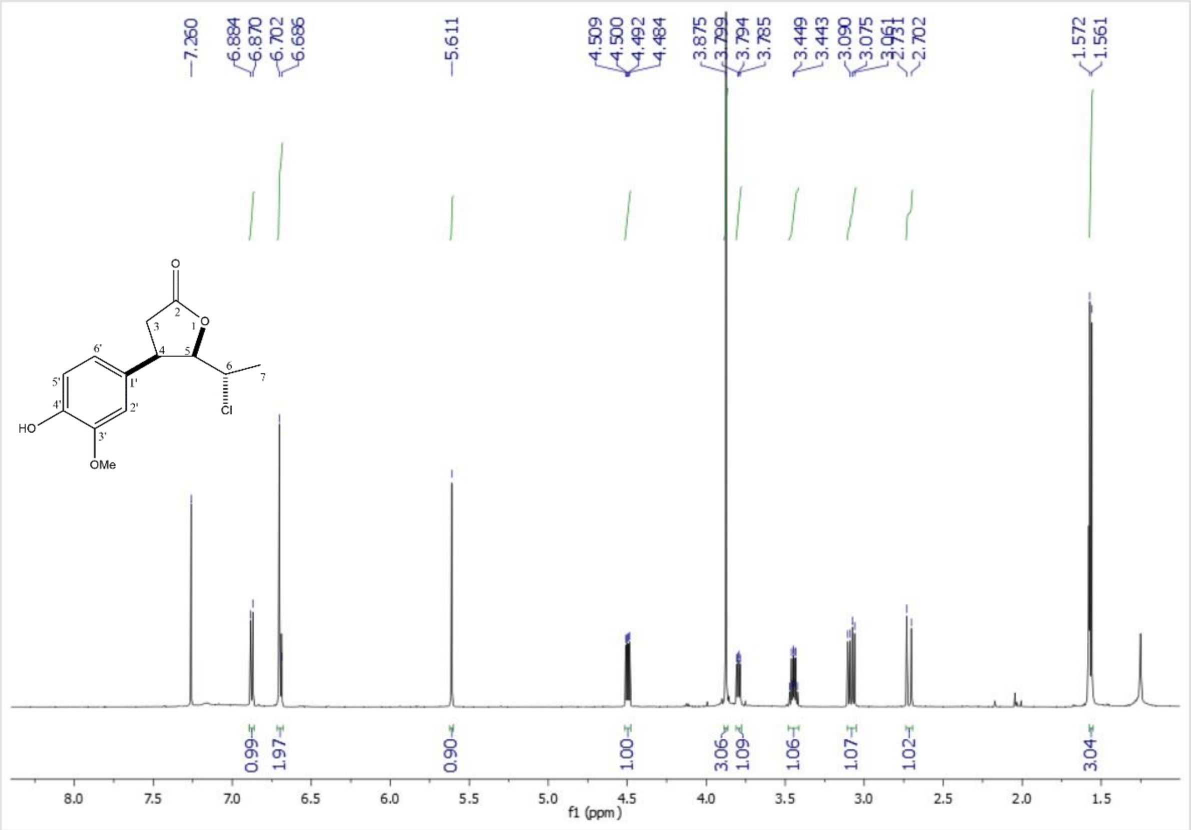


**Figure S91.** $^{1}$H NMR spectrum of *cis*-5-(1-chloroethyl)-4-(4'-hydroxy-3'-methoxyphenyl)dihydrofuran-2-one **(12a)**

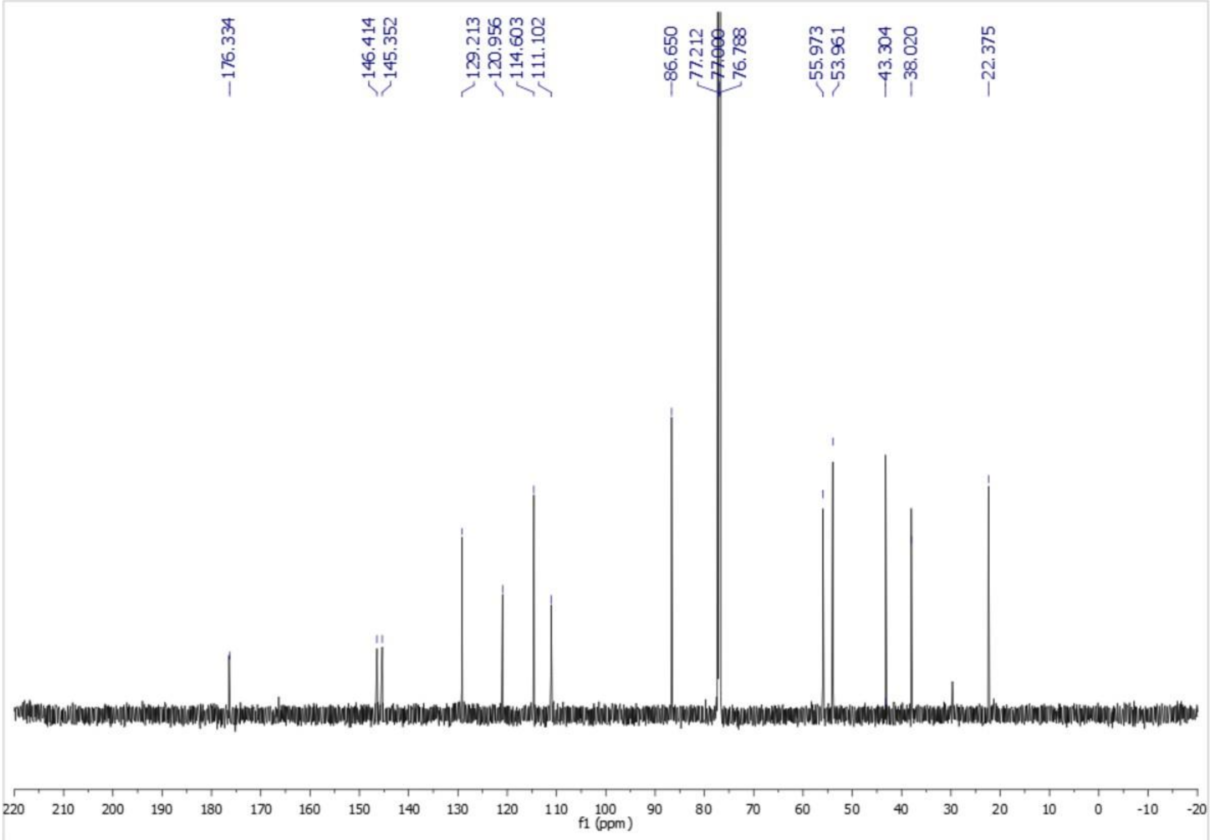


**Figure S92.** $^{13}$C NMR spectrum of *cis*-5-(1-chloroethyl)-4-(4'-hydroxy-3'-methoxyphenyl)dihydrofuran-2-one **(12a)**

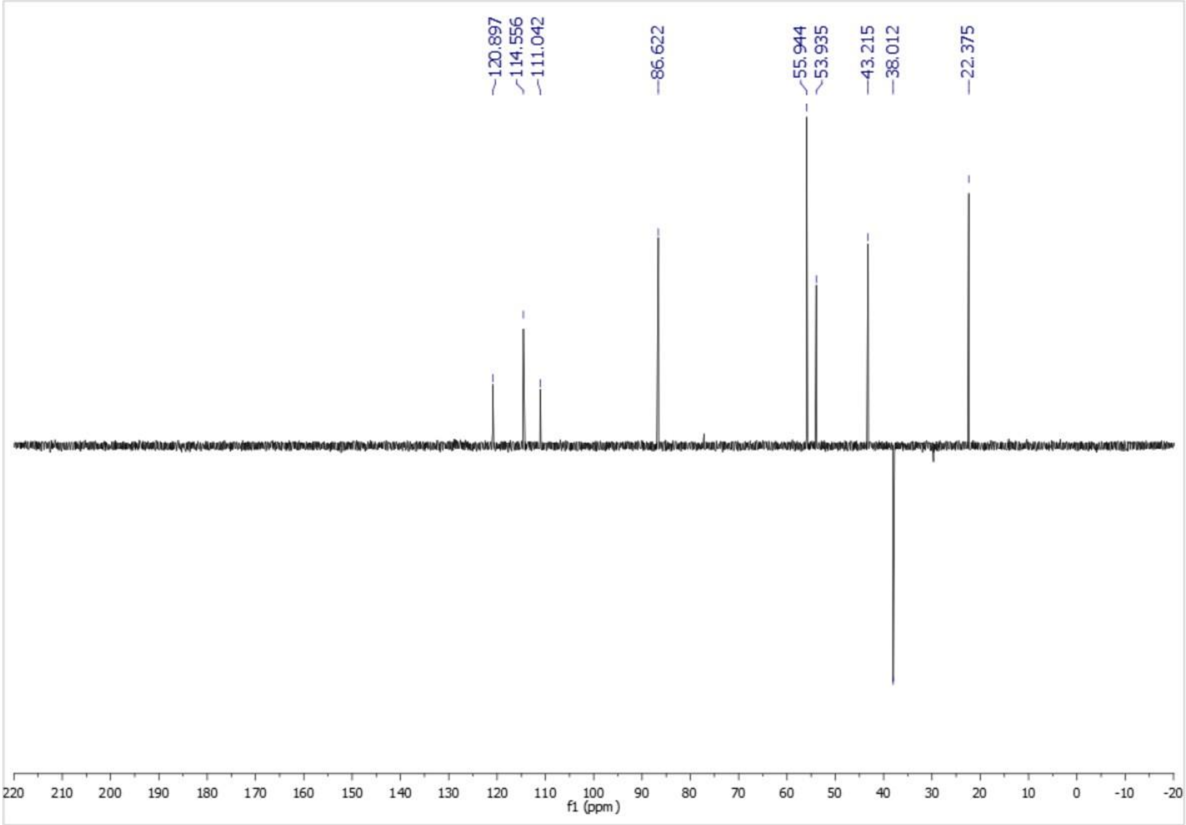


**Figure S93.** DEPT 135 NMR spectrum of *cis*-5-(1-chloroethyl)-4-(4'-hydroxy-3'-methoxyphenyl)dihydrofuran-2-one **(12a)**

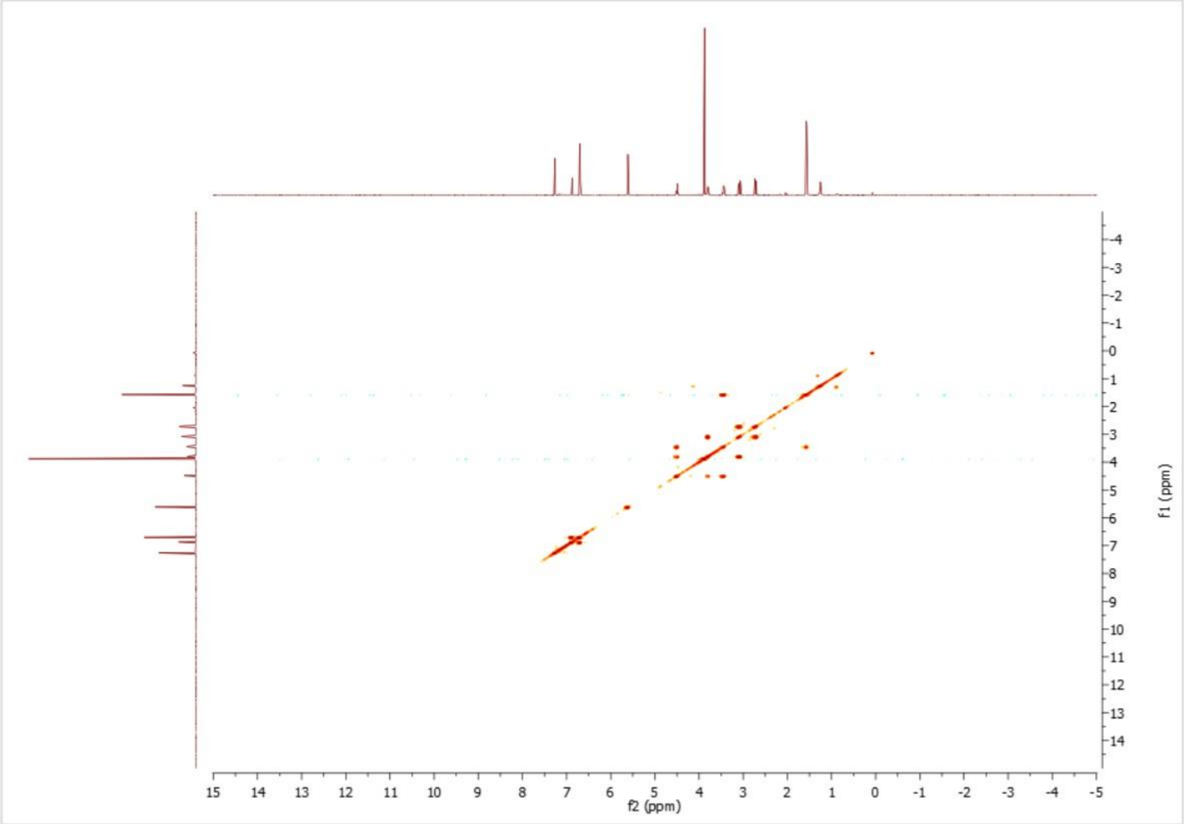


**Figure S94.** COSY spectrum of *cis*-5-(1-chloroethyl)-4-(4'-hydroxy-3'-methoxyphenyl)dihydrofuran-2-one **(12a)**

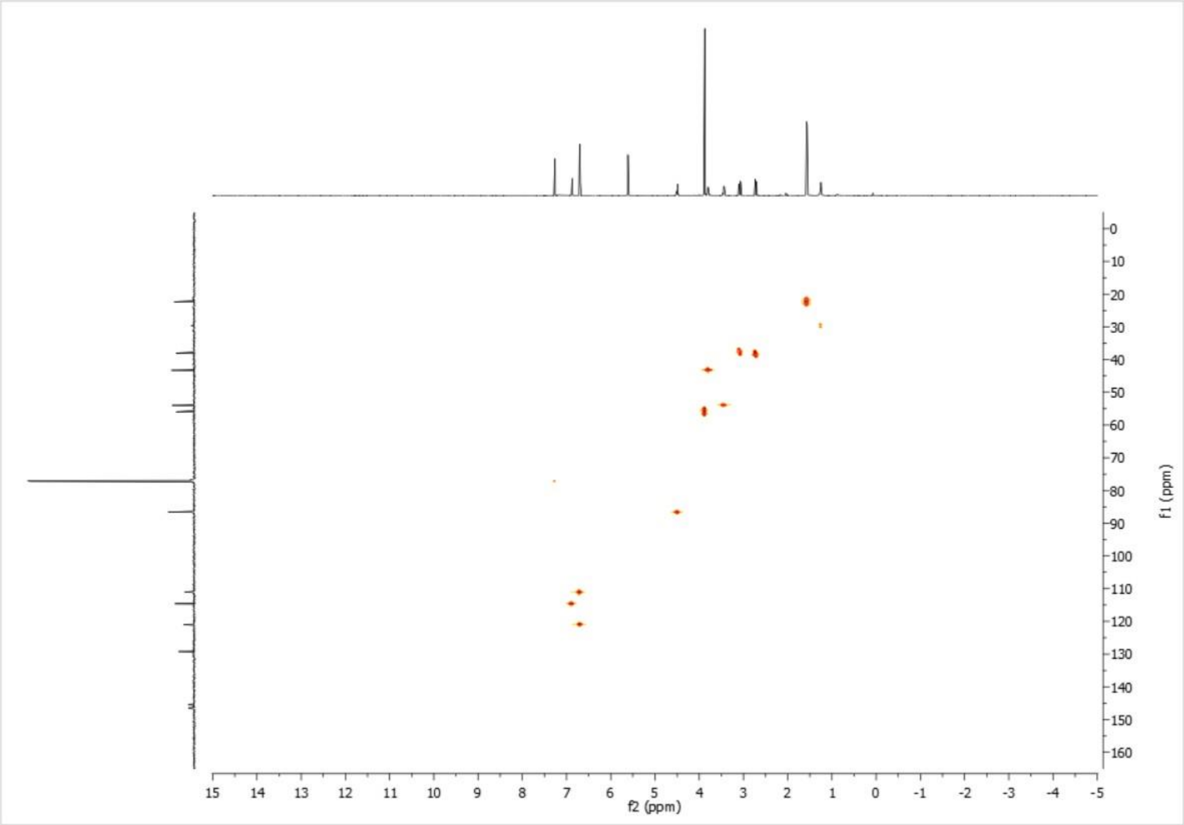


**Figure S95.** HMQC spectrum of *cis*-5-(1-chloroethyl)-4-(4'-hydroxy-3'-methoxyphenyl)dihydrofuran-2-one **(12a)**

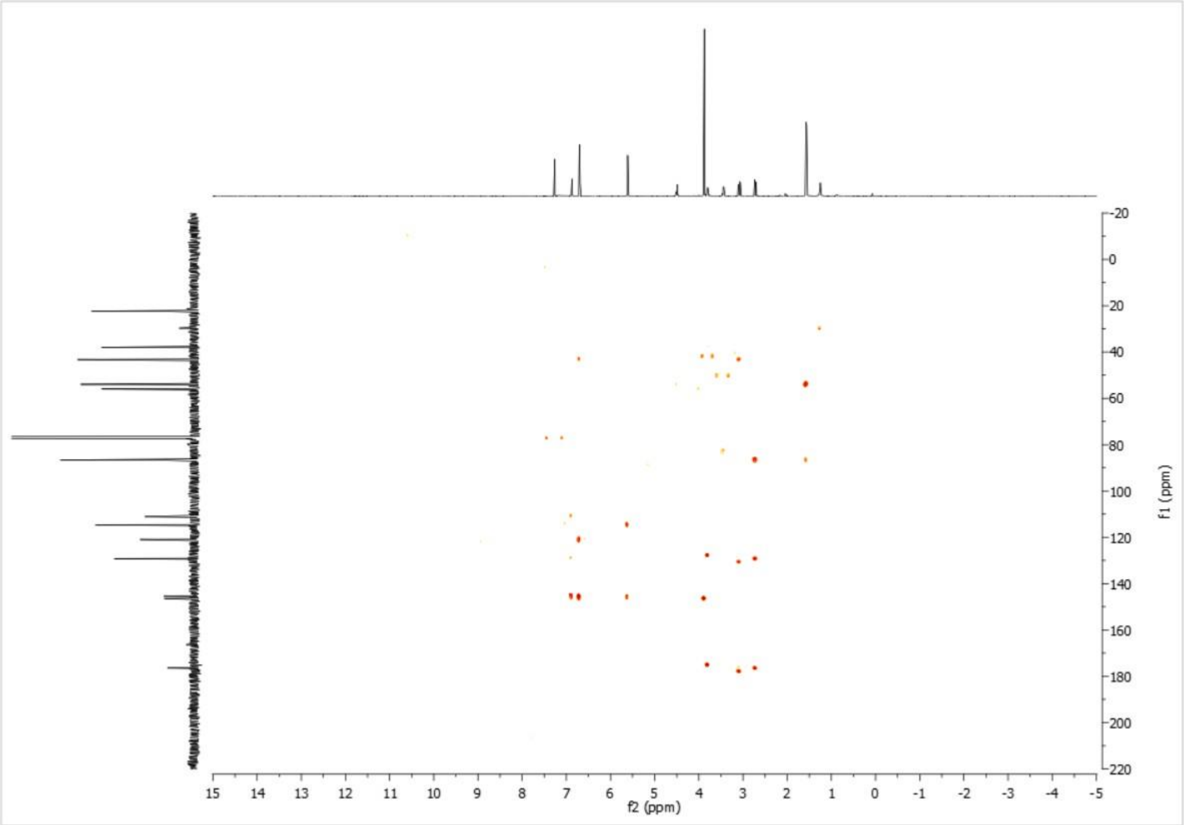


**Figure S96.** HMBC spectrum of *cis*-5-(1-chloroethyl)-4-(4'-hydroxy-3'-methoxyphenyl)dihydrofuran-2-one **(12a)**

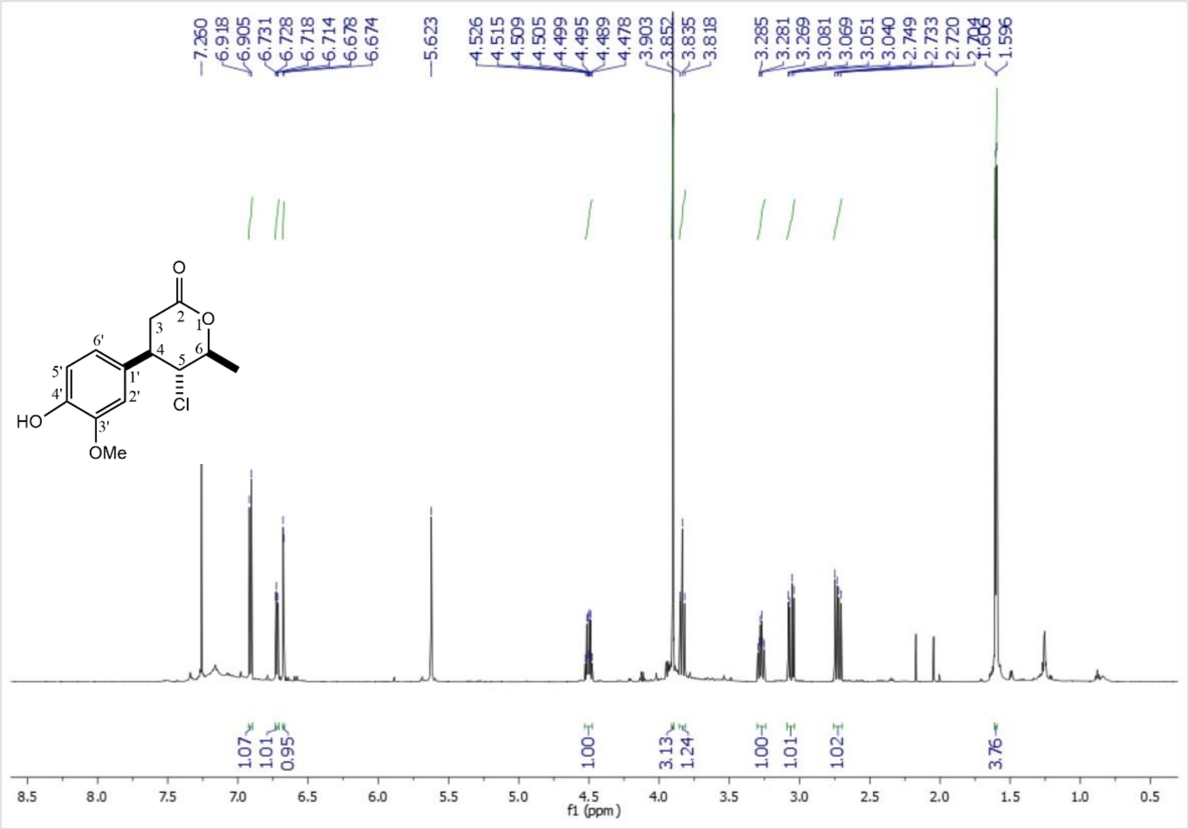

**Figure S97.** $^{1}H$ NMR spectrum of 5-*t*-chloro-4-*r*-(4'-hydroxy-3'-methoxyphenyl)-6-*c*-methyltetrahydropyran-2-one **(12b)**

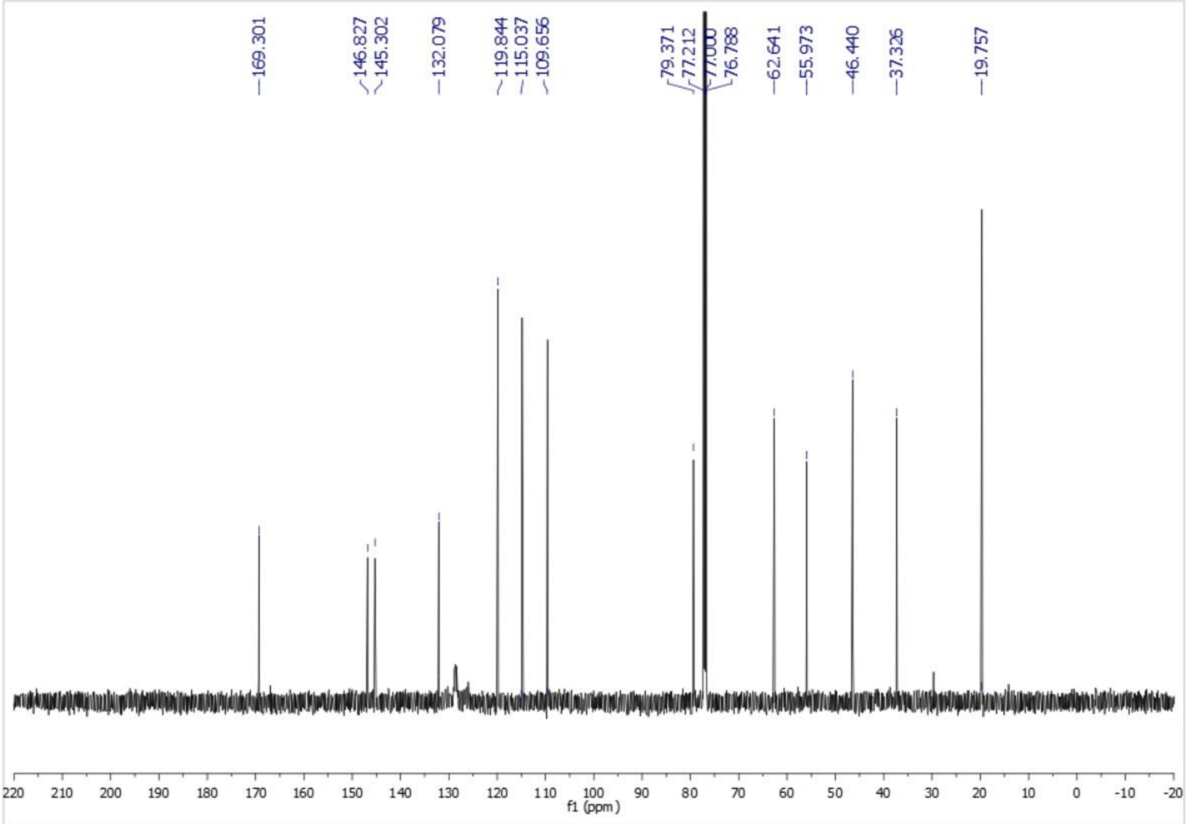

**Figure S98.** $^{13}C$ NMR spectrum of 5-*t*-chloro-4-*r*-(4'-hydroxy-3'-methoxyphenyl)-6-*c*-methyltetrahydropyran-2-one **(12b)**

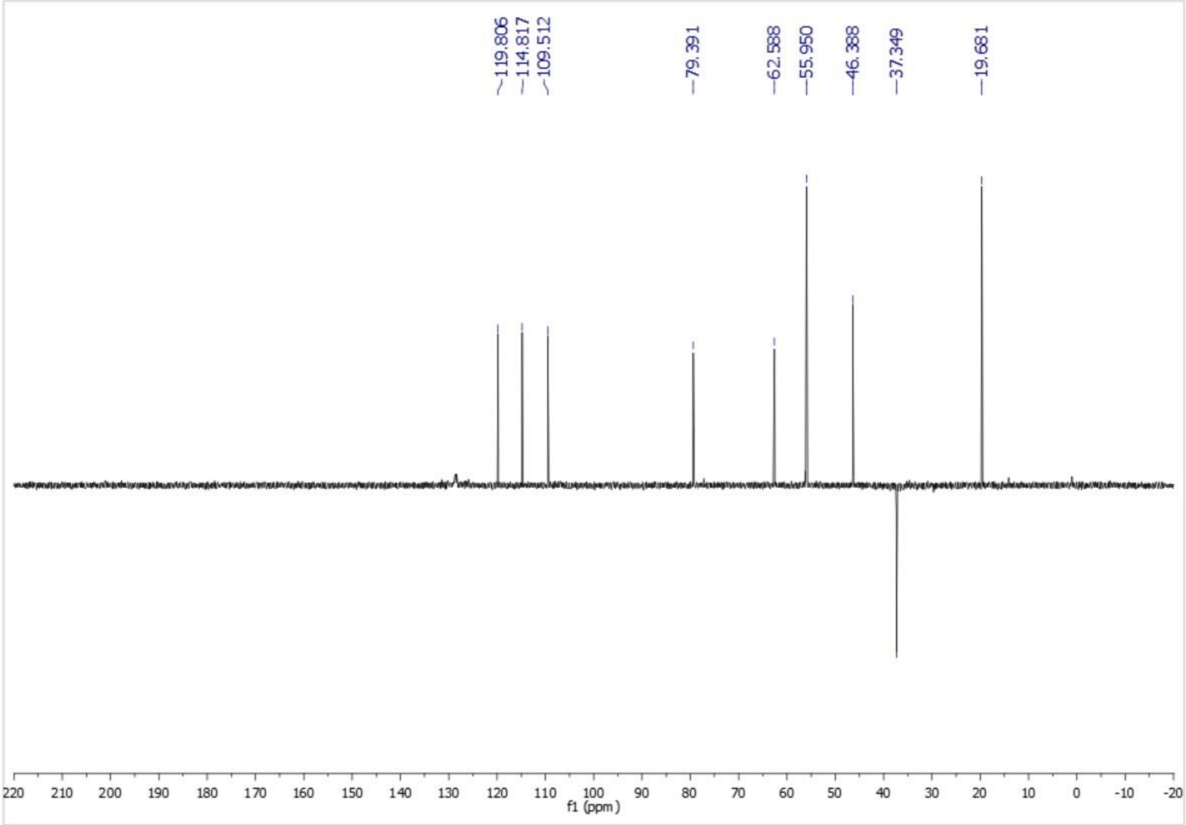


**Figure S99.** DEPT 135 NMR spectrum of 5-*t*-chloro-4-*r*-(4'-hydroxy-3'-methoxyphenyl)-6-*c*-methyltetrahydropyran-2-one **(12b)**

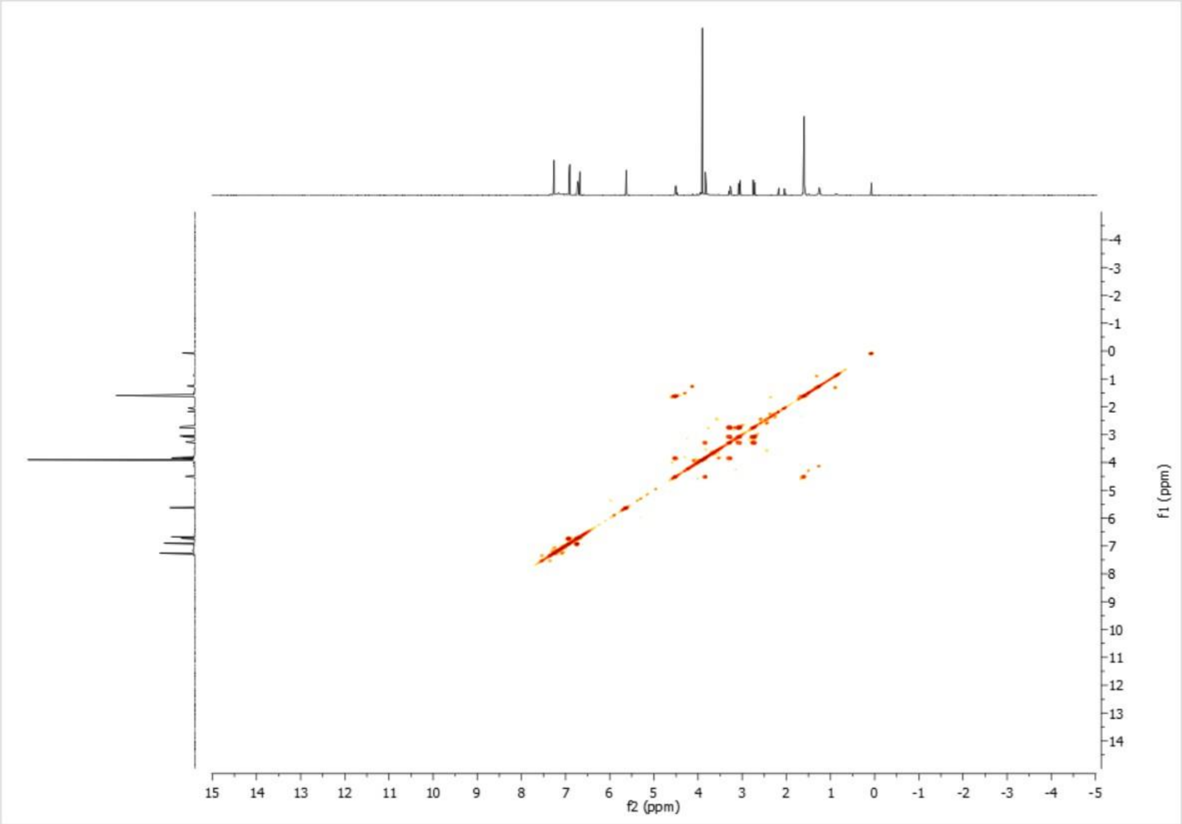


**Figure S100.** COSY spectrum of 5-*t*-chloro-4-*r*-(4'-hydroxy-3'-methoxyphenyl)-6-*c*-methyltetrahydropyran-2-one **(12b)**

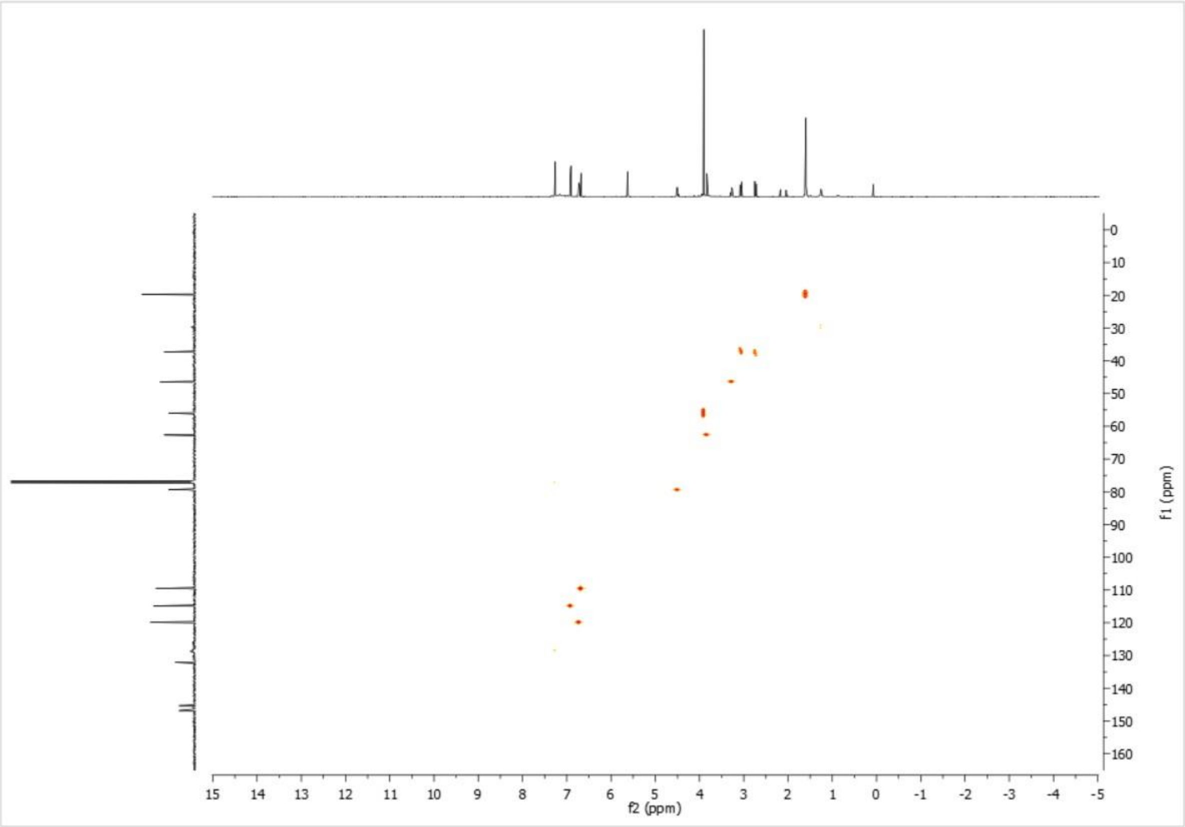


**Figure S101.** HMQC spectrum of 5-*t*-chloro-4-*r*-(4'-hydroxy-3'-methoxyphenyl)-6-*c*-methyltetrahydropyran-2-one **(12b)**

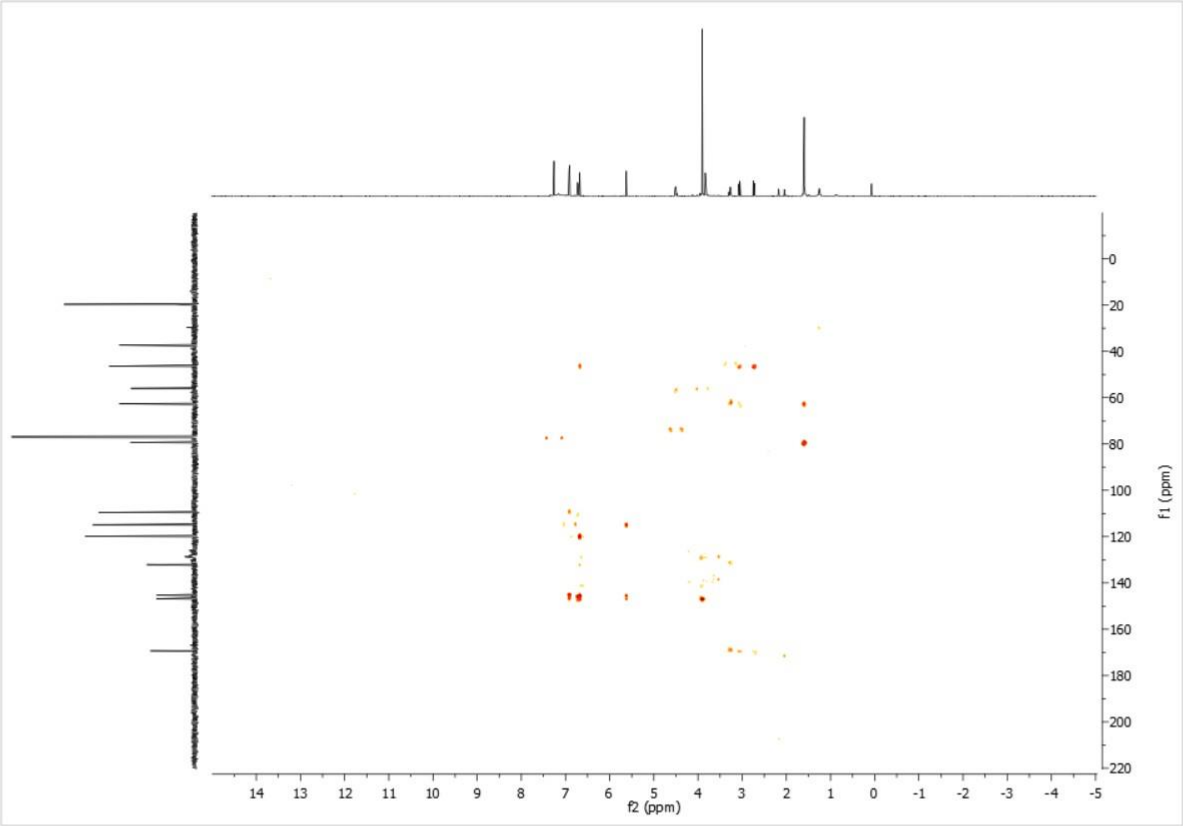


**Figure S102.** HMBC spectrum of 5-*t*-chloro-4-*r*-(4'-hydroxy-3'-methoxyphenyl)-6-*c*-methyltetrahydropyran-2-one **(12b)**

# IR SPECTRA:

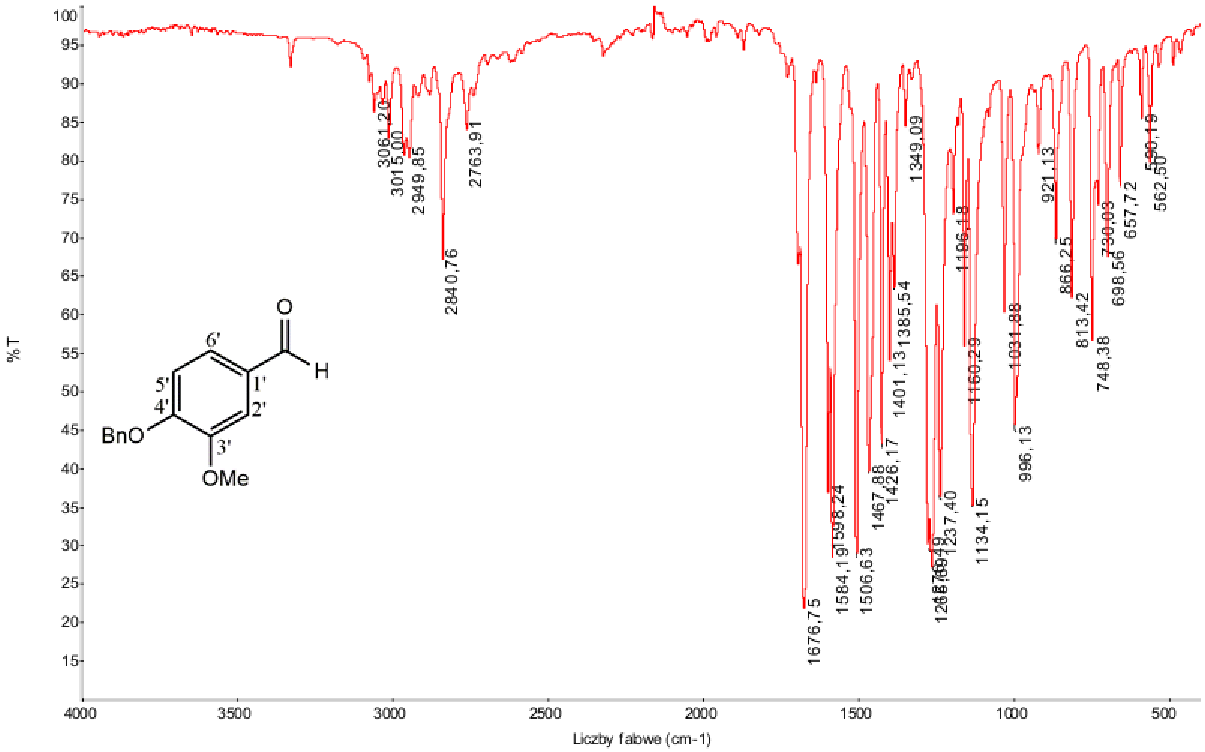


**Figure S103.** IR spectrum of benzylvanillin **(2)**

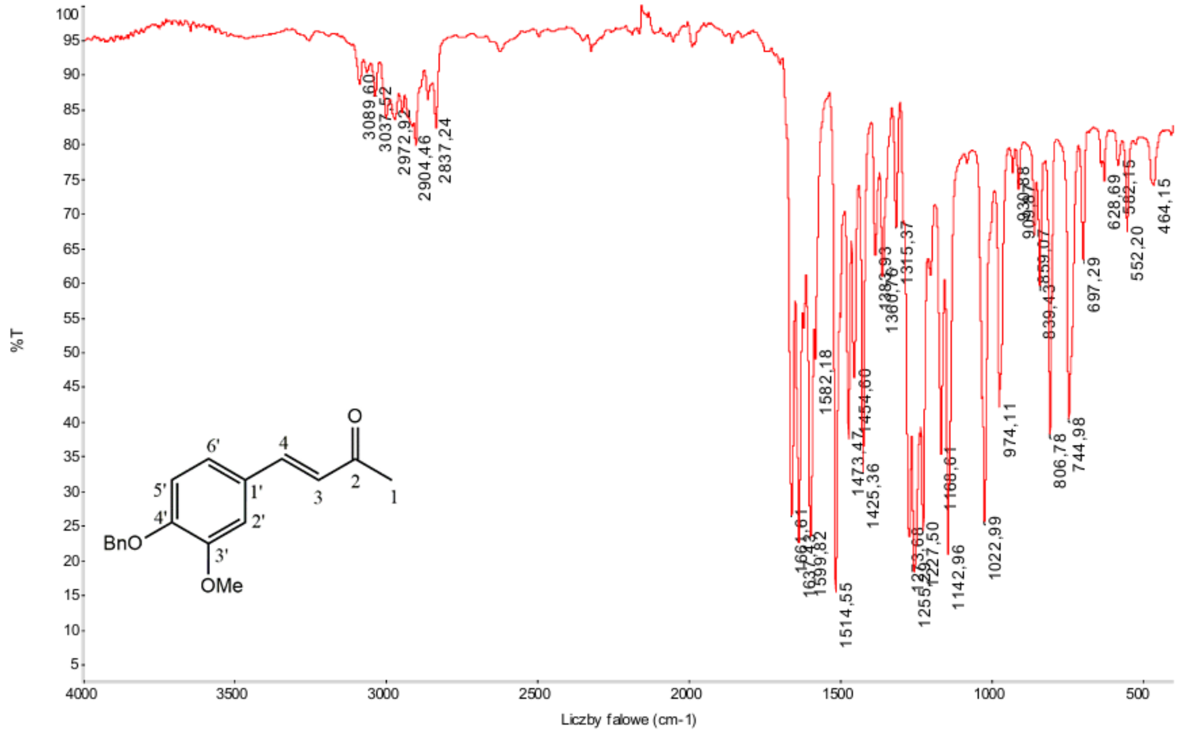


**Figure S104.** IR spectrum of (*E*)-4-(4'-(benzyloxy-3'-methoxyphenyl)but-3-en-2-one **(3)**

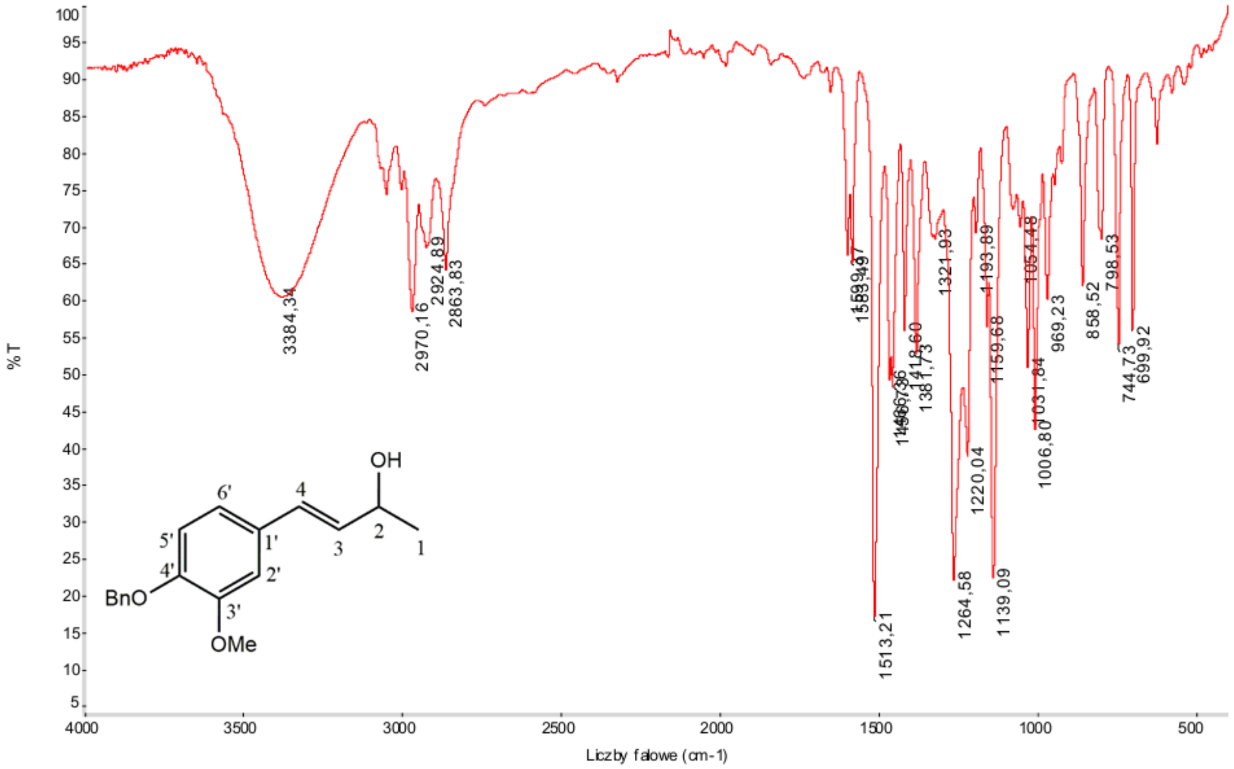


**Figure S105.** IR spectrum of (*E*)-4-(4'-benzyloxy-3'-methoxyphenyl)but-3-en-2-ol **(4)**

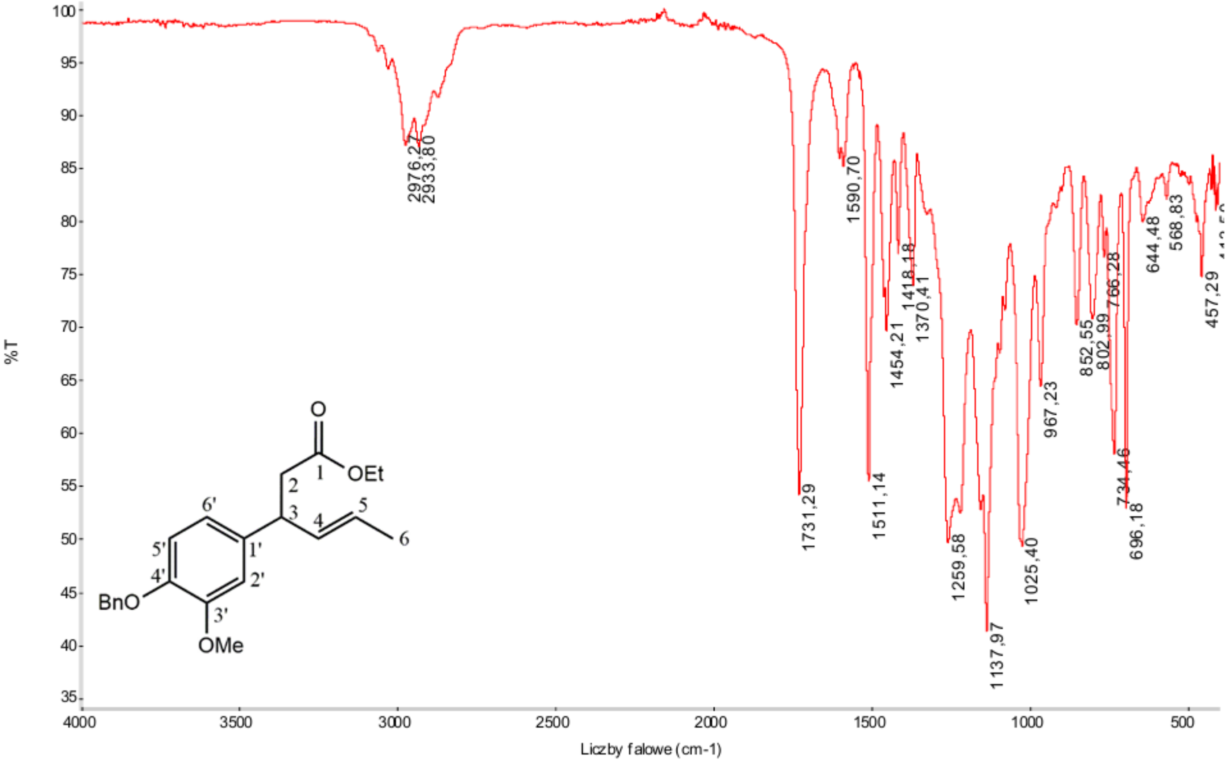


**Figure S106.** IR spectrum of (*E*)-3-(4'-benzyloxy-3'-methoxyphenyl)hex-4-enoate **(5)**

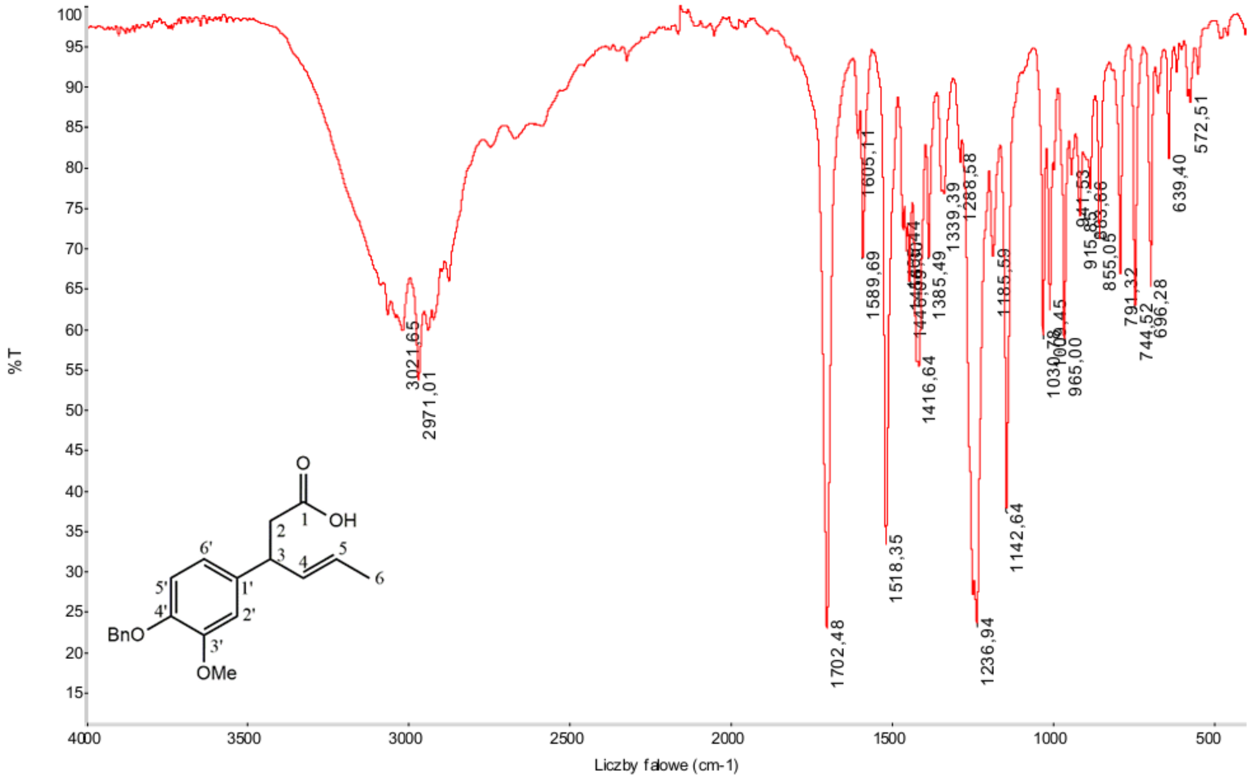

**Figure S107.** IR spectrum of (*E*)-3-(4'-benzyloxy-3'-methoxyphenyl)hex-4-enoic acid **(6)**

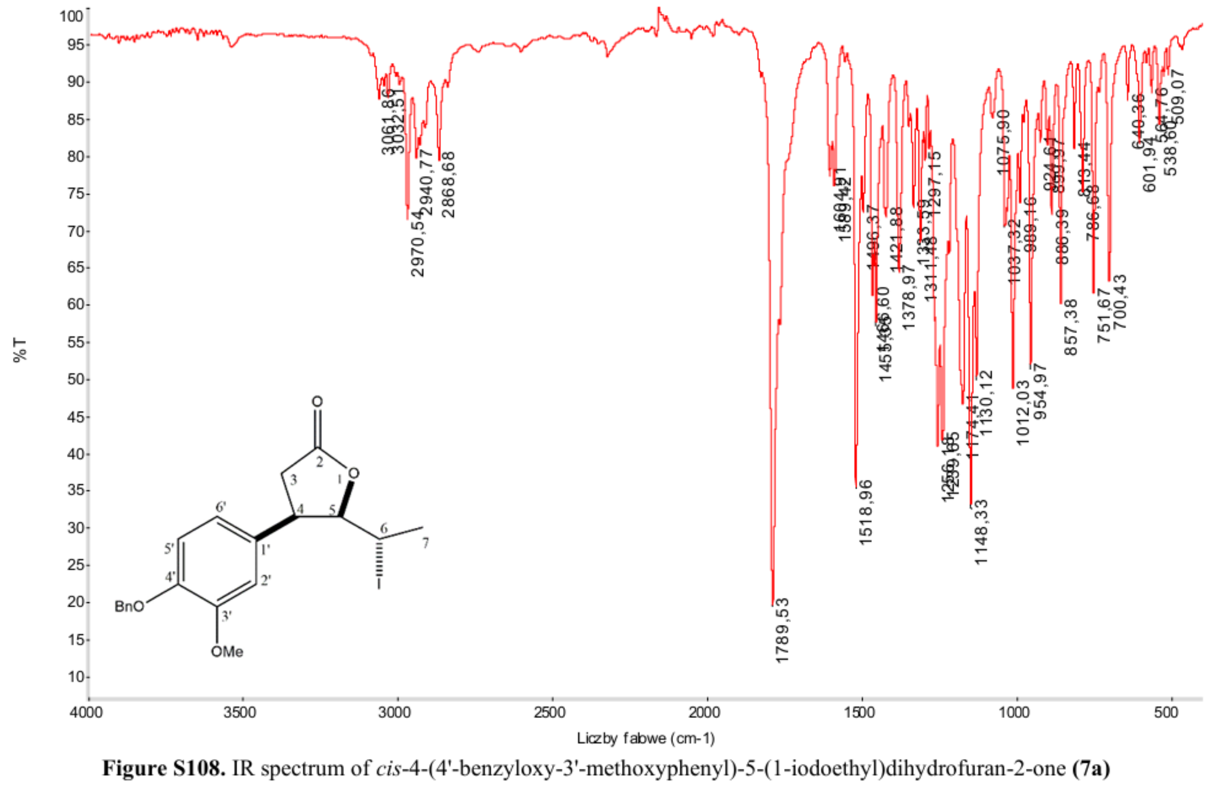

**Figure S108.** IR spectrum of *cis*-4-(4'-benzyloxy-3'-methoxyphenyl)-5-(1-iodoethyl)dihydrofuran-2-one **(7a)**

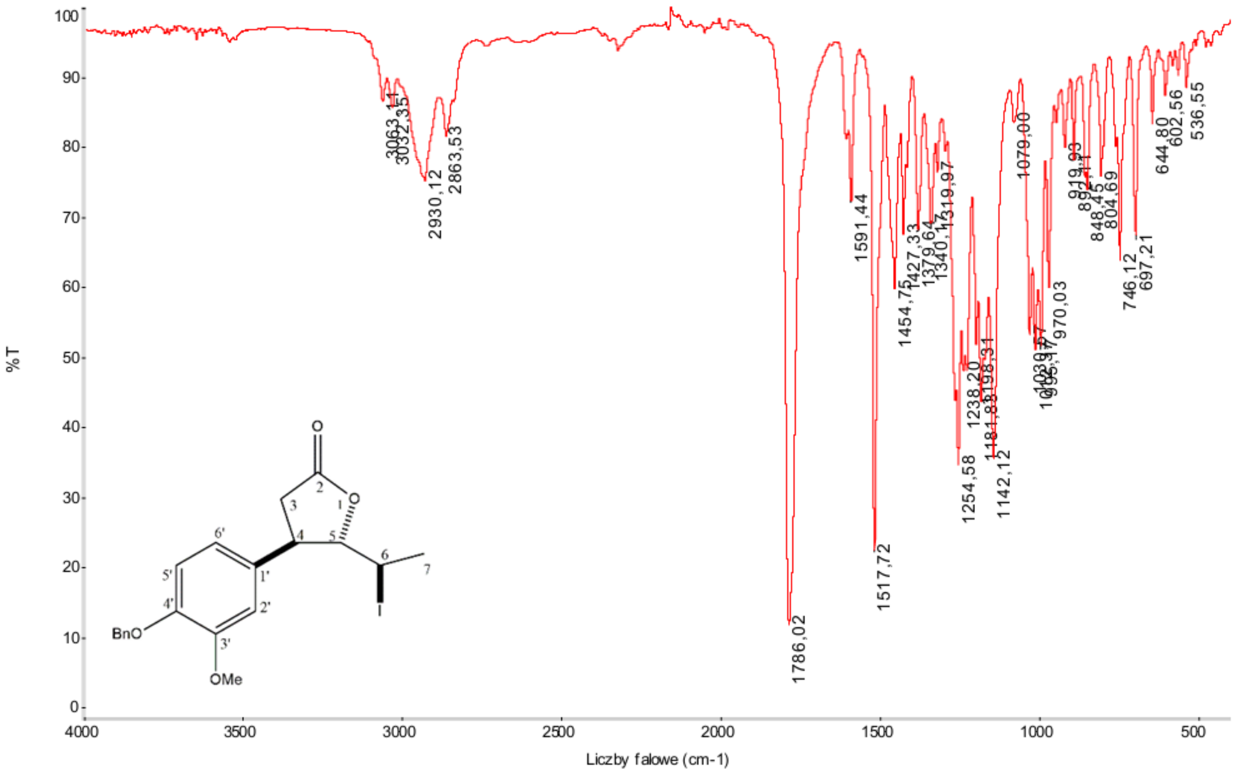


**Figure S109.** IR spectrum of *trans*-4-(4'-benzyloxy-3'-methoxyphenyl)-5-(1-iodoethyl)dihydrofuran-2-one **(7b)**

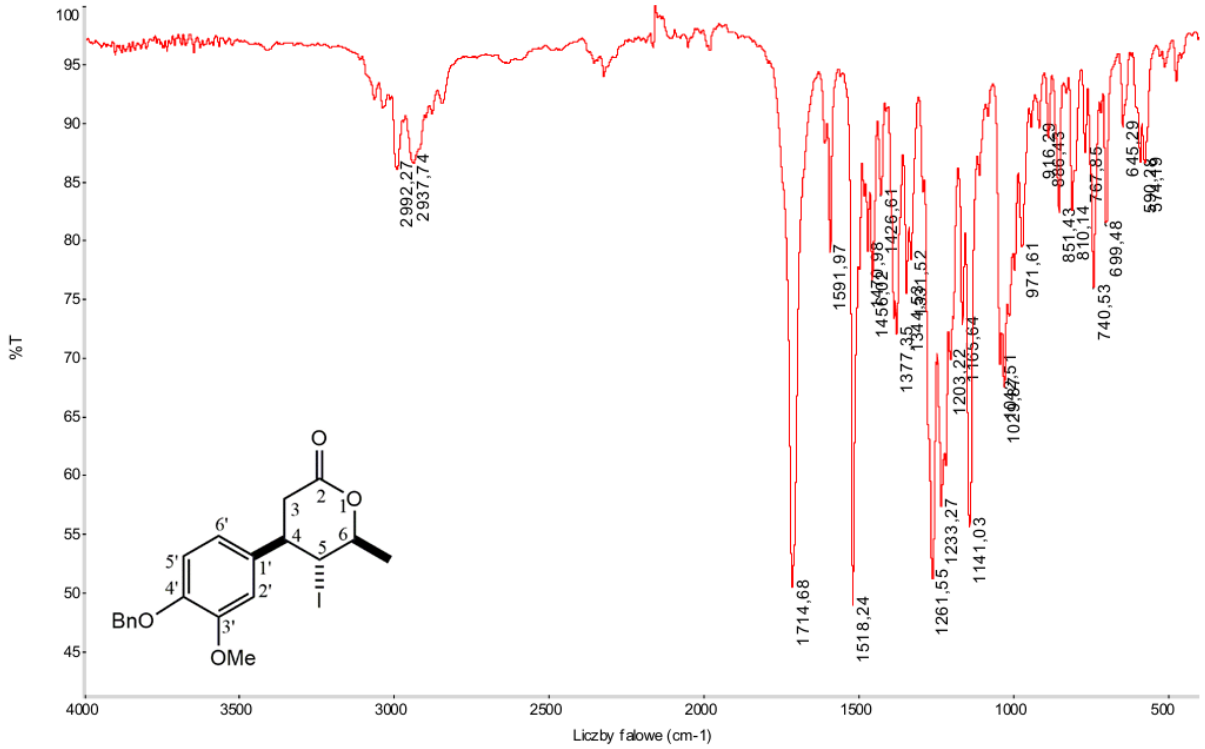


**Figure S110.** IR spectrum of 4-*r*-(4'-benzyloxy-3'-methoxyphenyl)-5-*t*-iodo-6-*c*-methyltetrahydropyran-2-one **(7c)**

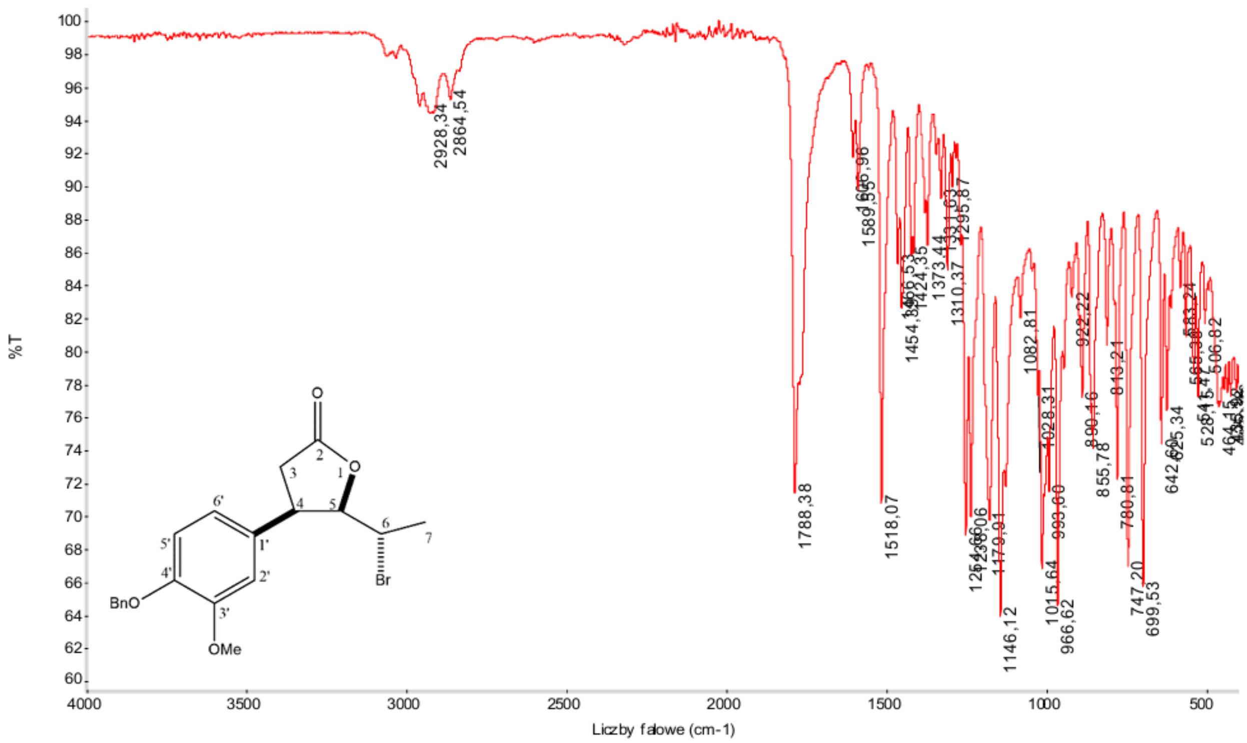


**Figure S111.** IR spectrum of *cis*-4-(4'-benzyloxy-3'-methoxyphenyl)-5-(1-bromoethyl)dihydrofuran-2-one **(8a)**

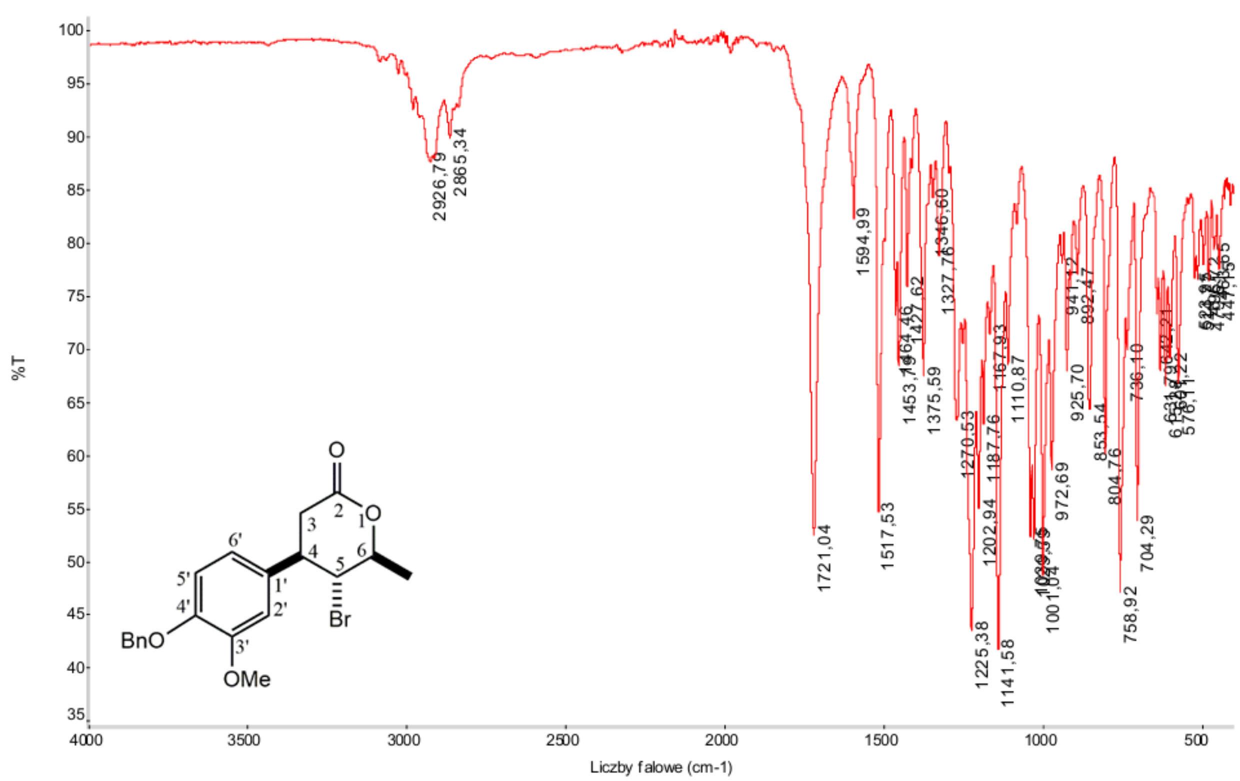


**Figure S112.** IR spectrum of 4-*r*-(4'-benzyloxy-3'-methoxyphenyl)-5-*t*-bromo-6-*c*-methyltetrahydropyran-2-one **(8b)**

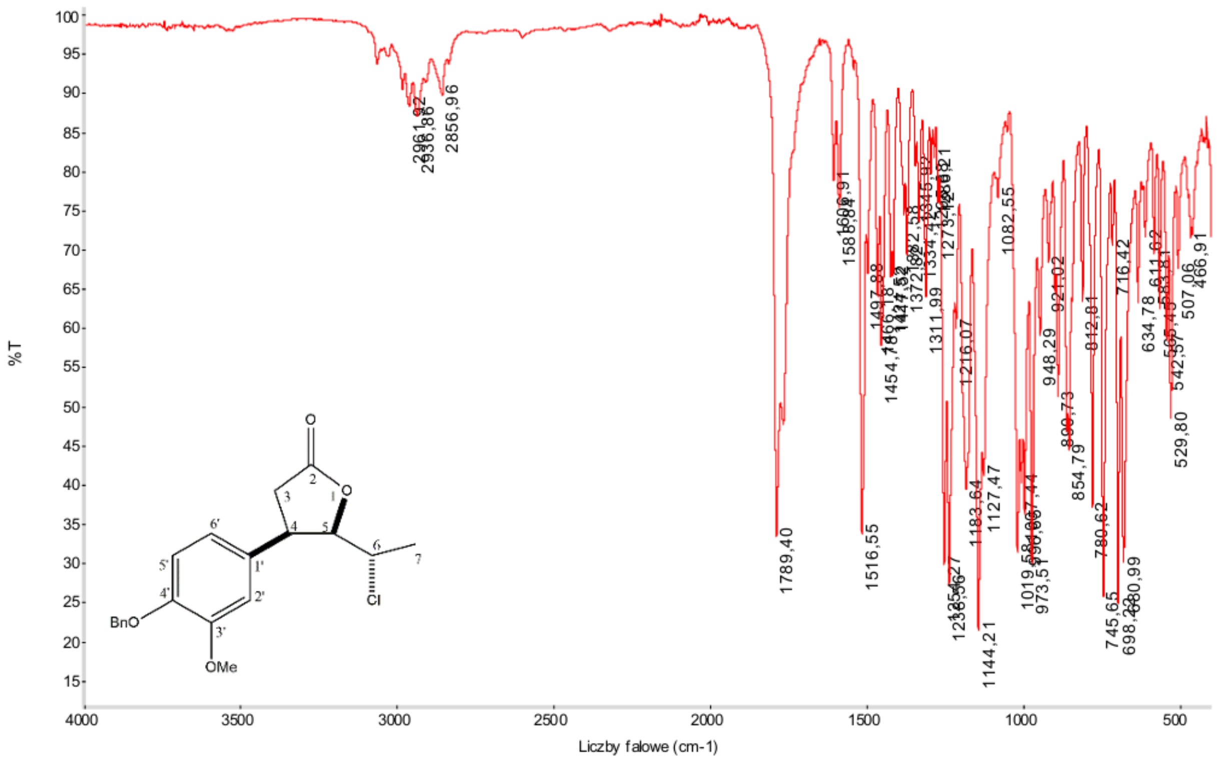

**Figure S113.** IR spectrum of *cis*-4-(4'-benzyloxy-3'-methoxyphenyl)-5-(1-chloroethyl)dihydrofuran-2-one **(9a)**

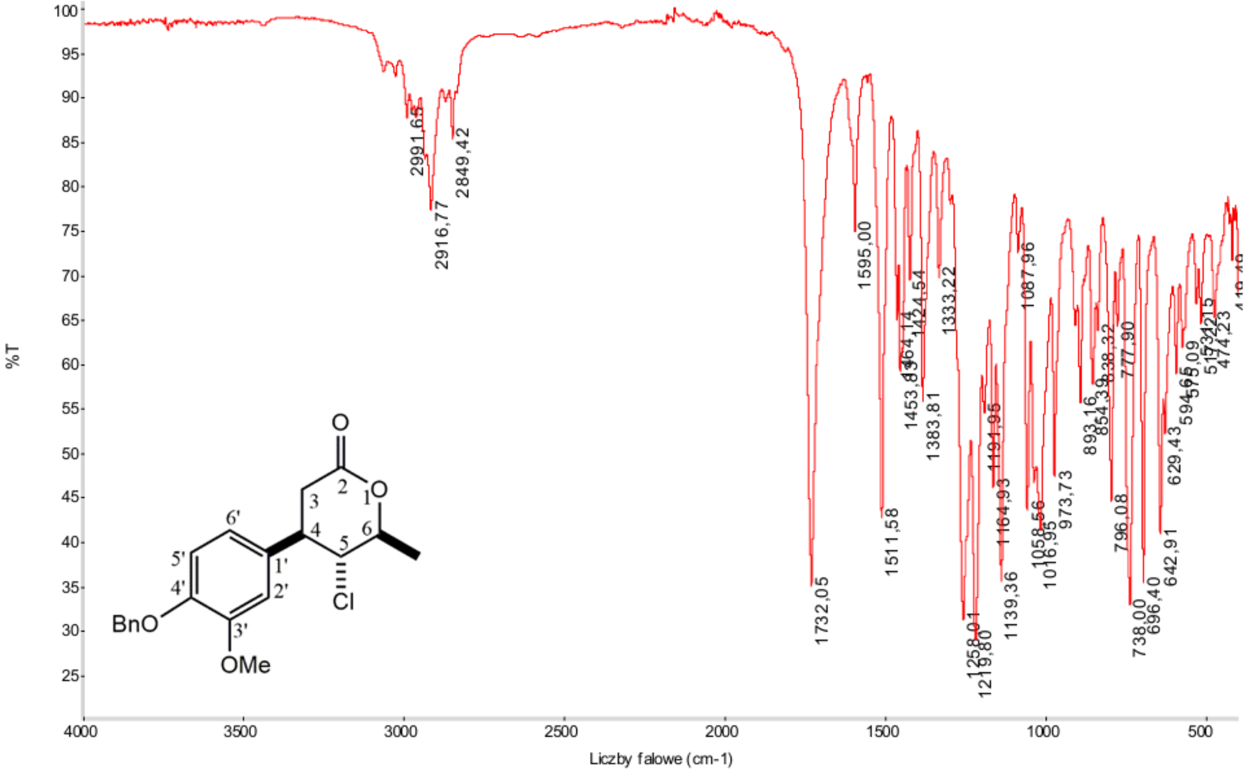

**Figure S114.** IR spectrum of 4-*r*-(4'-benzyloxy-3'-methoxyphenyl)-5-*t*-chloro-6-*c*-methyltetrahydropyran-2-one **(9b)**

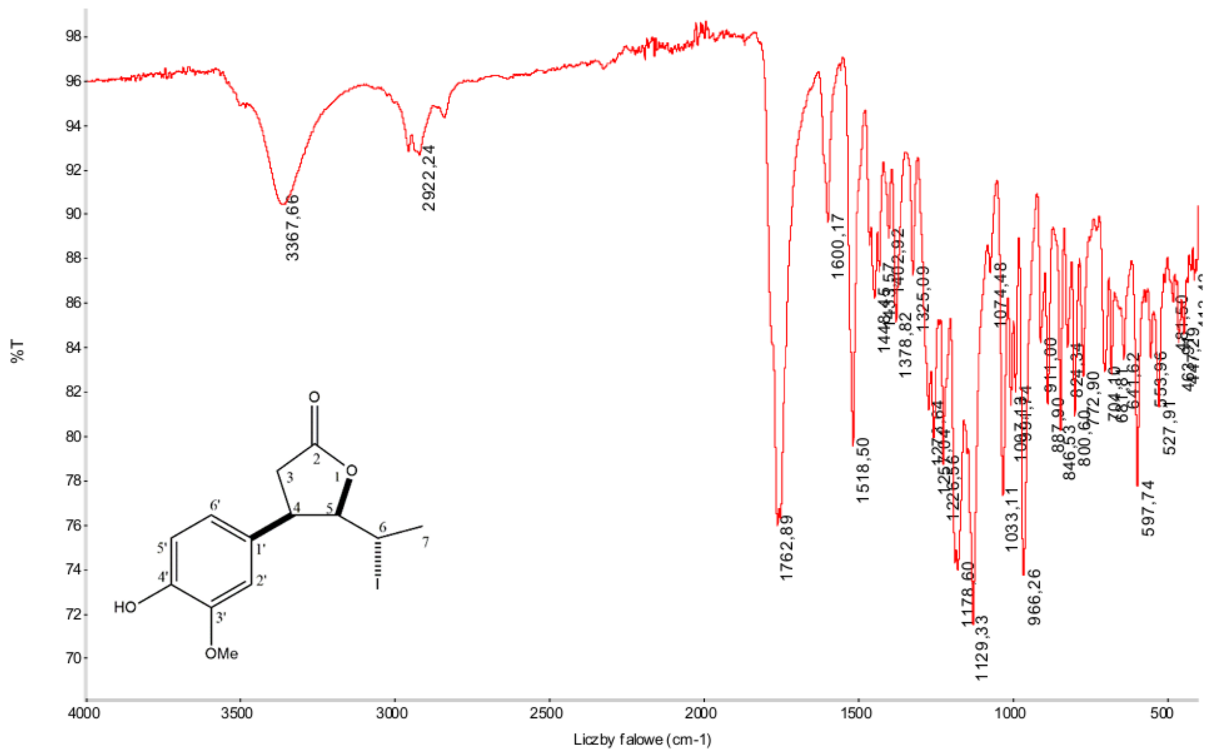

**Figure S115.** IR spectrum of *cis*-4-(4'-hydroxy-3'-methoxyphenyl)-5-(1-iodoethyl)dihydrofuran-2-one **(10a)**

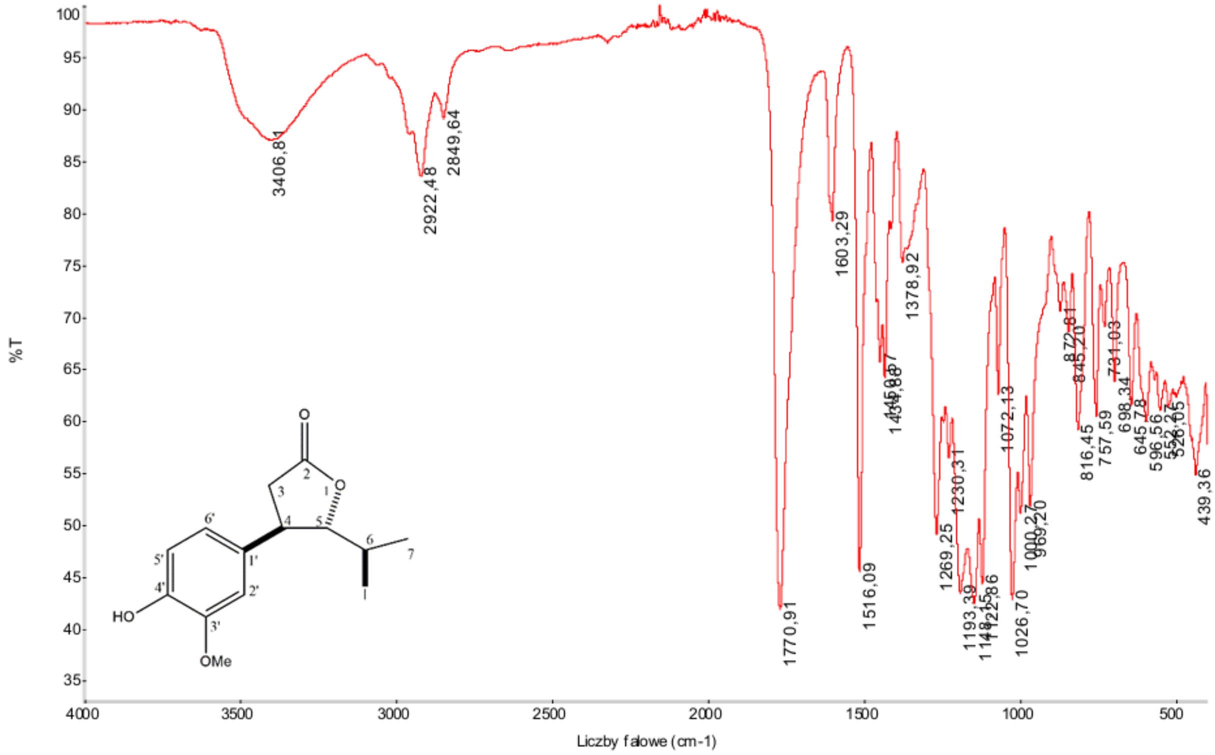

**Figure S116.** IR spectrum of *trans*-4-(4'-hydroxy-3'-methoxyphenyl)-5-(1-iodoethyl)dihydrofuran-2-one **(10b)**

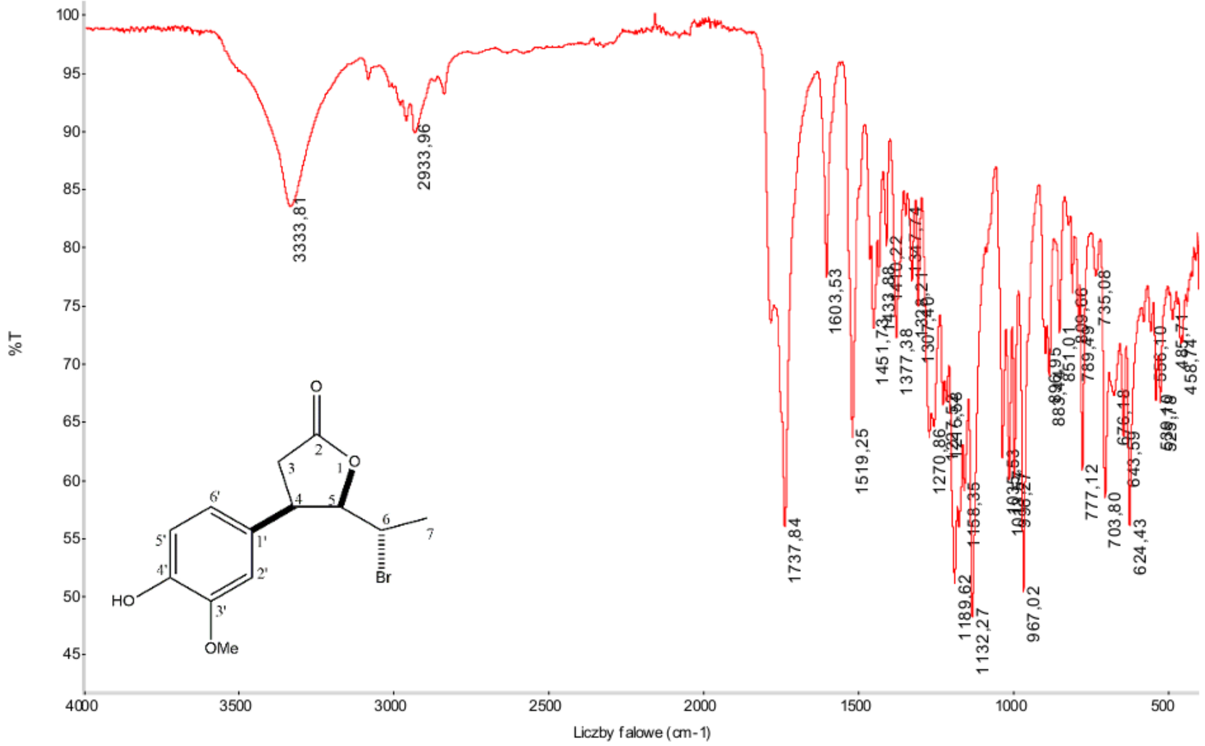


**Figure S117.** IR spectrum of *cis*-5-(1-bromoethyl)-4-(4'-hydroxy-3'-methoxyphenyl)dihydrofuran-2-one **(11a)**

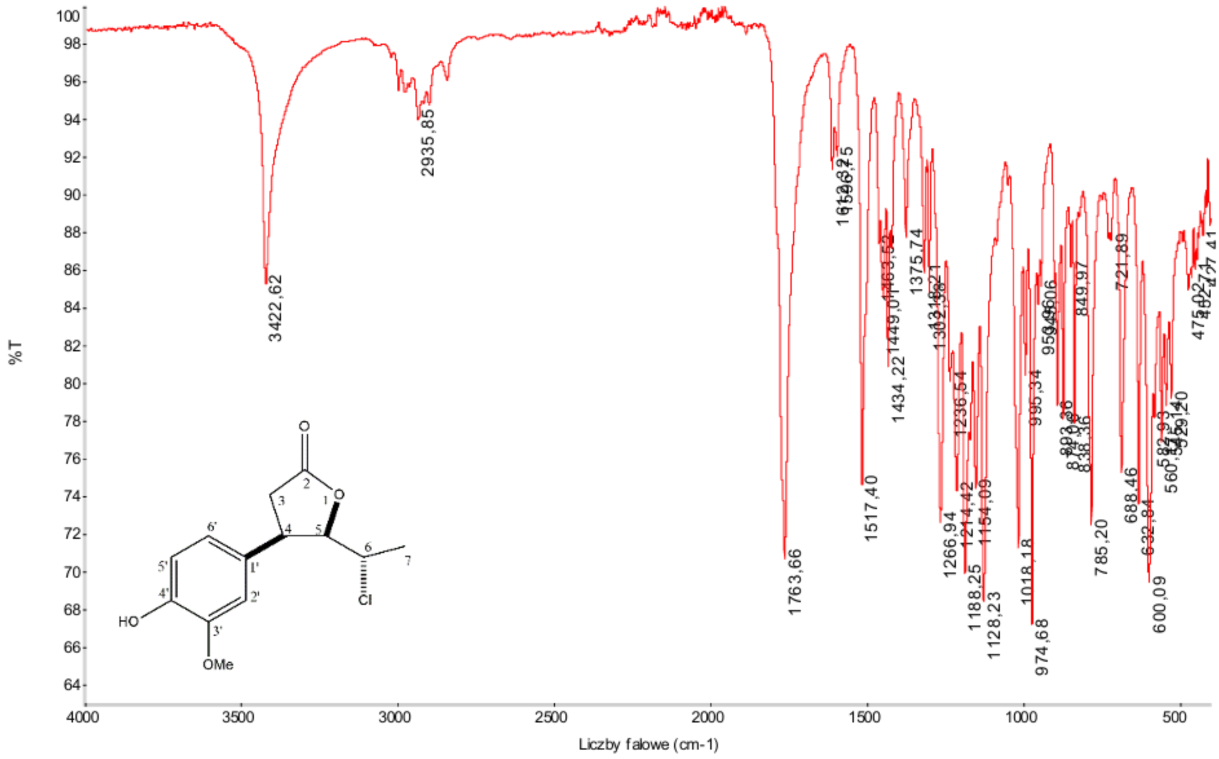


**Figure S118.** IR spectrum of *cis*-5-(1-chloroethyl)-4-(4'-hydroxy-3'-methoxyphenyl)dihydrofuran-2-one **(12a)**

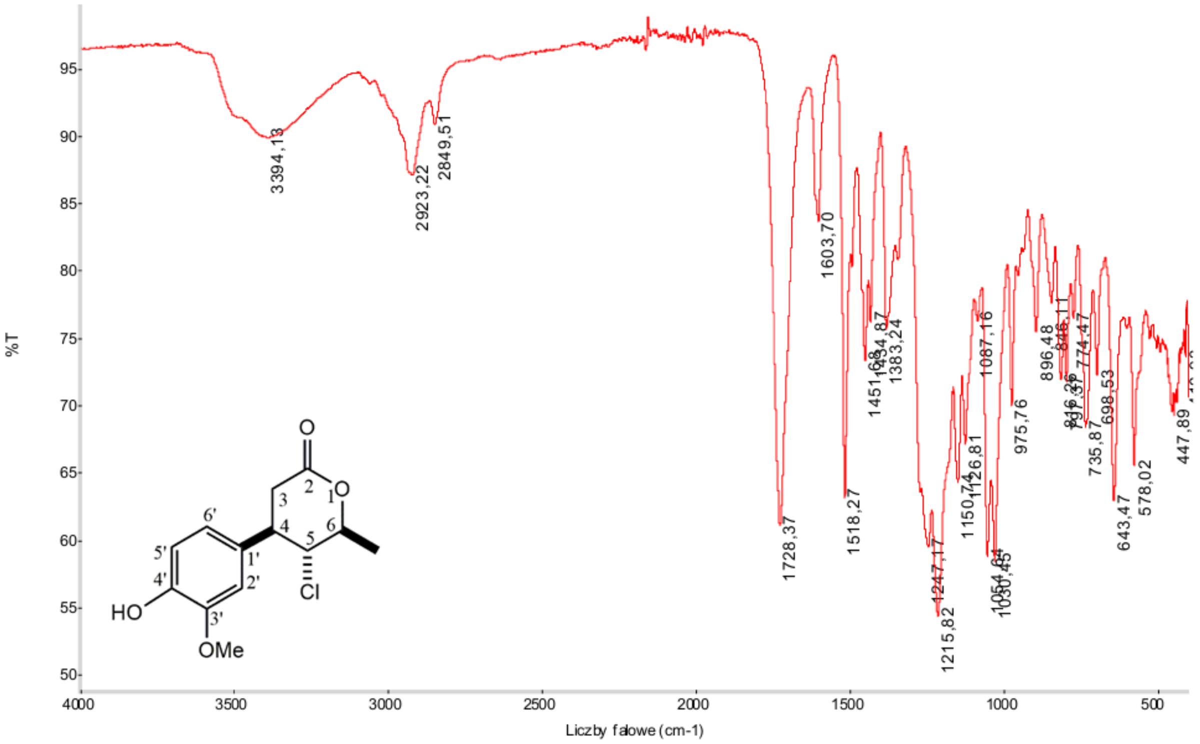


**Figure S119.** IR spectrum of 5-*t*-chloro-4-*r*-(4'-hydroxy-3'-methoxyphenyl)-6-*c*-methyltetrahydropyran-2-one **(12b)**

# HRMS SPECTRA:

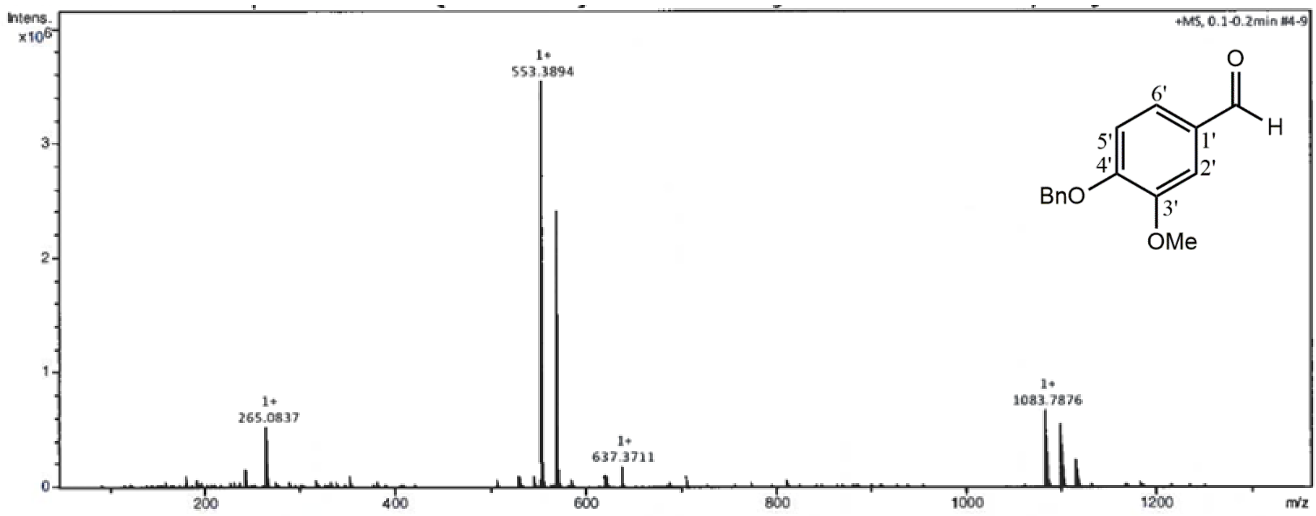


**Figure S120.** HRMS spectrum of benzylvanillin **(2)**

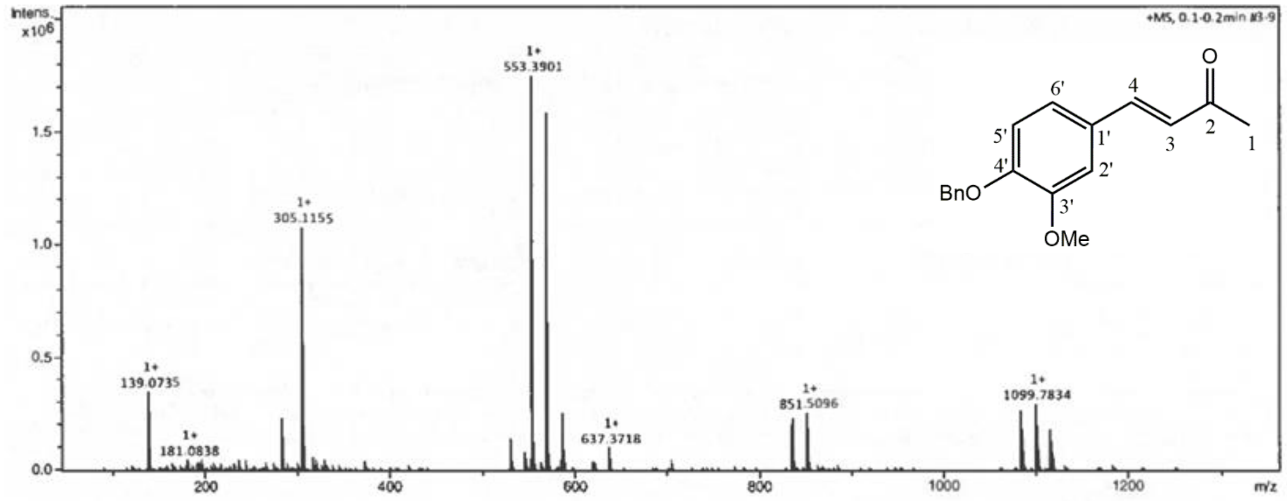


**Figure S121.** HRMS spectrum of (*E*)-4-(4'-(benzyloxy-3'-methoxyphenyl)but-3-en-2-one **(3)**

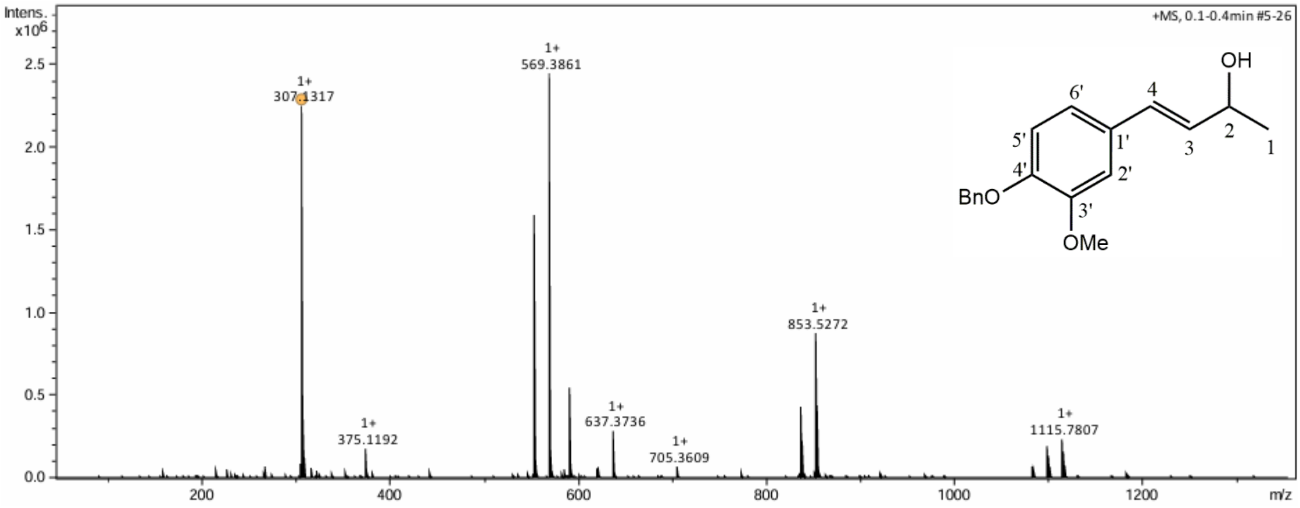


**Figure S122.** HRMS spectrum of (*E*)-4-(4'-benzyloxy-3'-methoxyphenyl)but-3-en-2-ol **(4)**

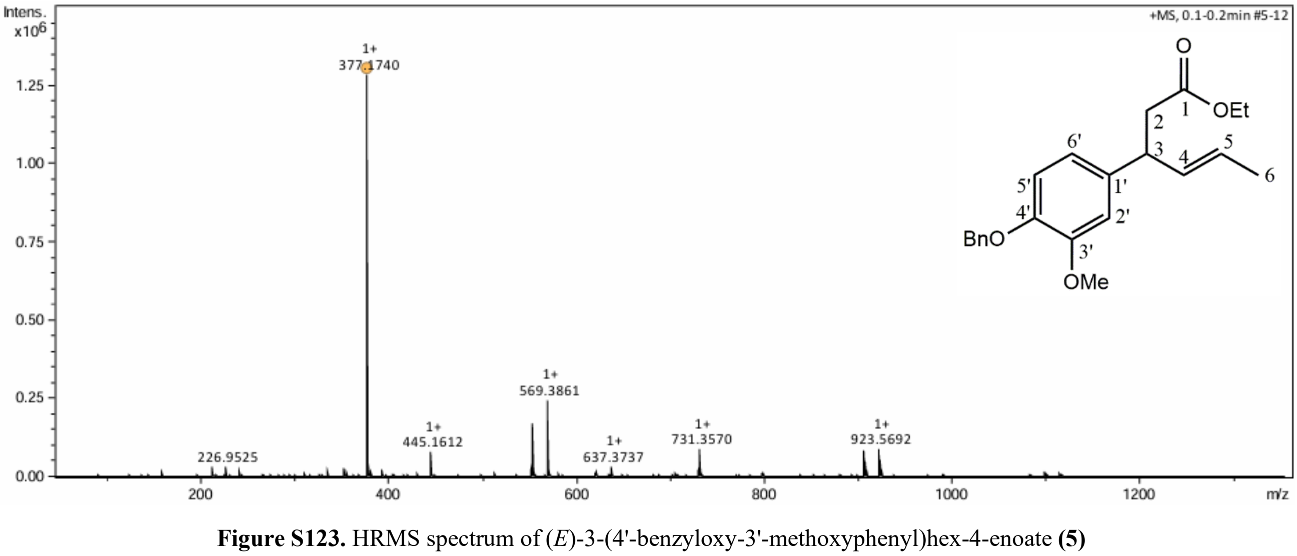


**Figure S123.** HRMS spectrum of (*E*)-3-(4'-benzyloxy-3'-methoxyphenyl)hex-4-enoate **(5)**

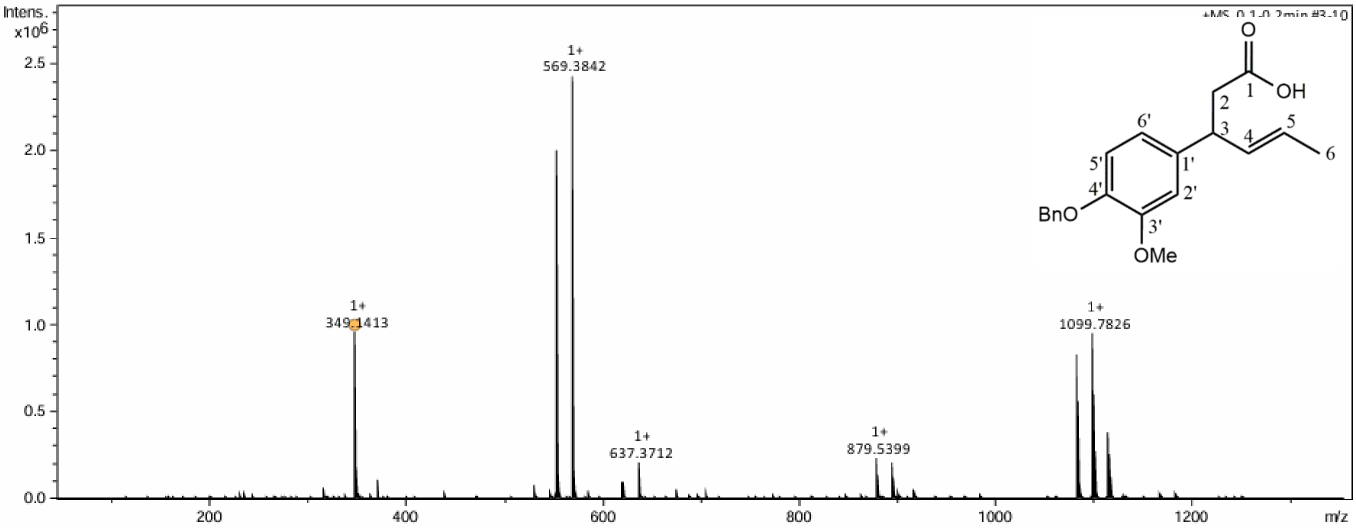


**Figure S124.** HRMS spectrum of (*E*)-3-(4'-benzyloxy-3'-methoxyphenyl)hex-4-enoic acid **(6)**

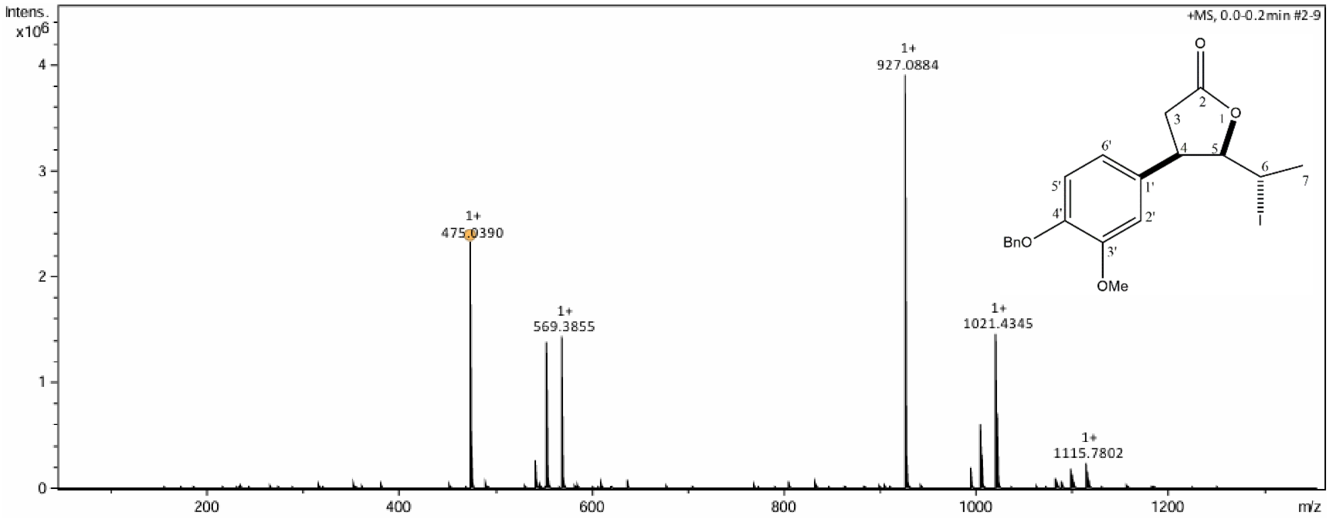


**Figure S125.** HRMS spectrum of *cis*-4-(4'-benzyloxy-3'-methoxyphenyl)-5-(1-iodoethyl)dihydrofuran-2-one **(7a)**

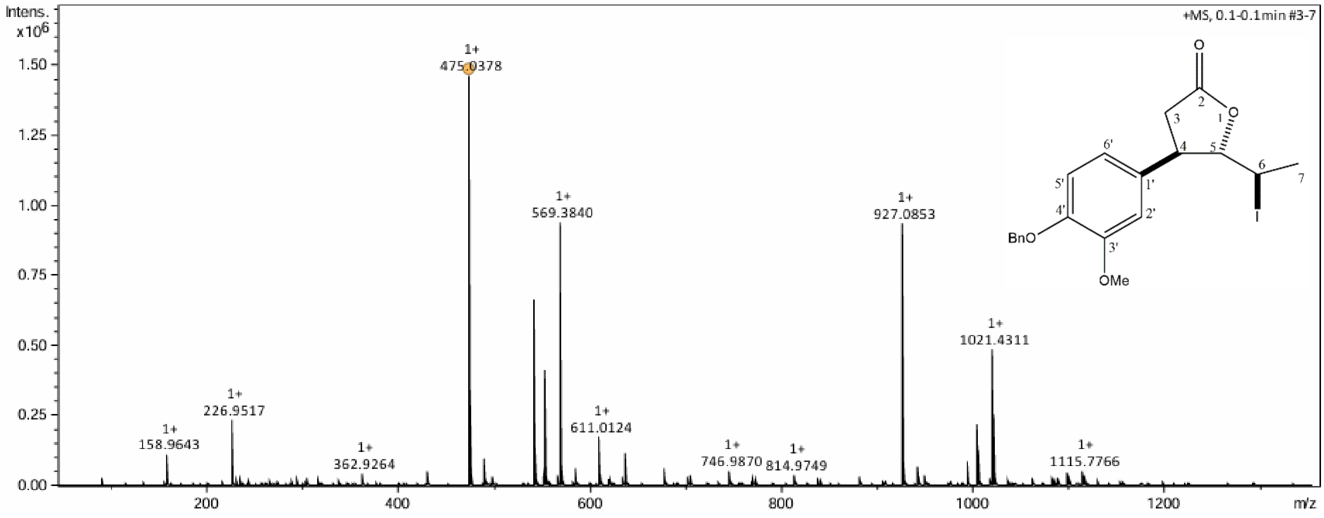

**Figure S126.** HRMS spectrum of *trans*-4-(4'-benzyloxy-3'-methoxyphenyl)-5-(1-iodoethyl)dihydrofuran-2-one **(7b)**

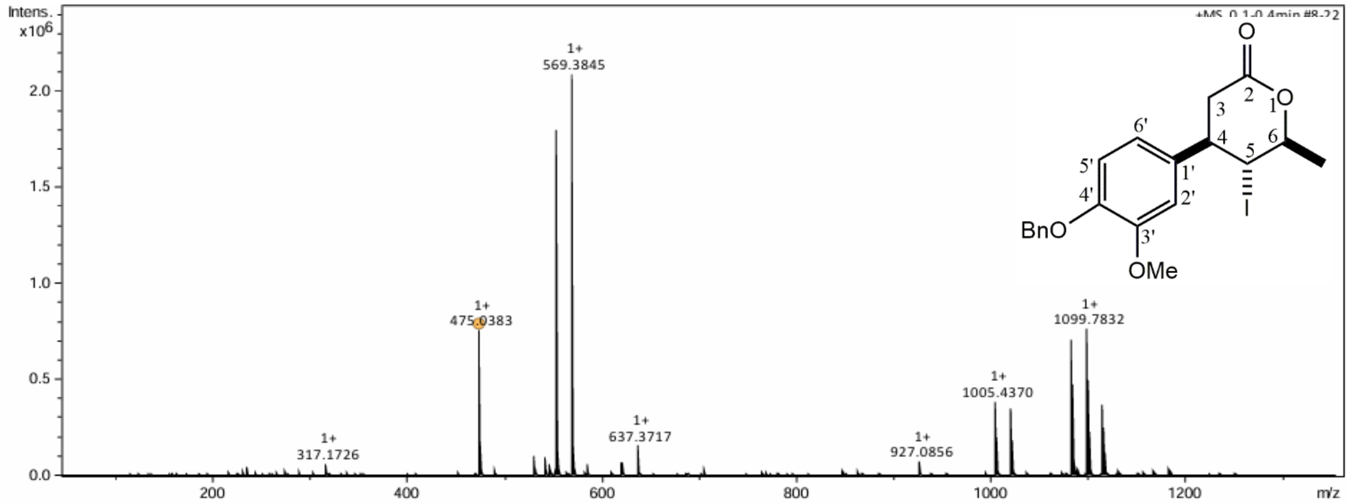


**Figure S127.** HRMS spectrum of 4-*r*-(4'-benzyloxy-3'-methoxyphenyl)-5-*t*-iodo-6-*c*-methyltetrahydropyran-2-one **(7c)**

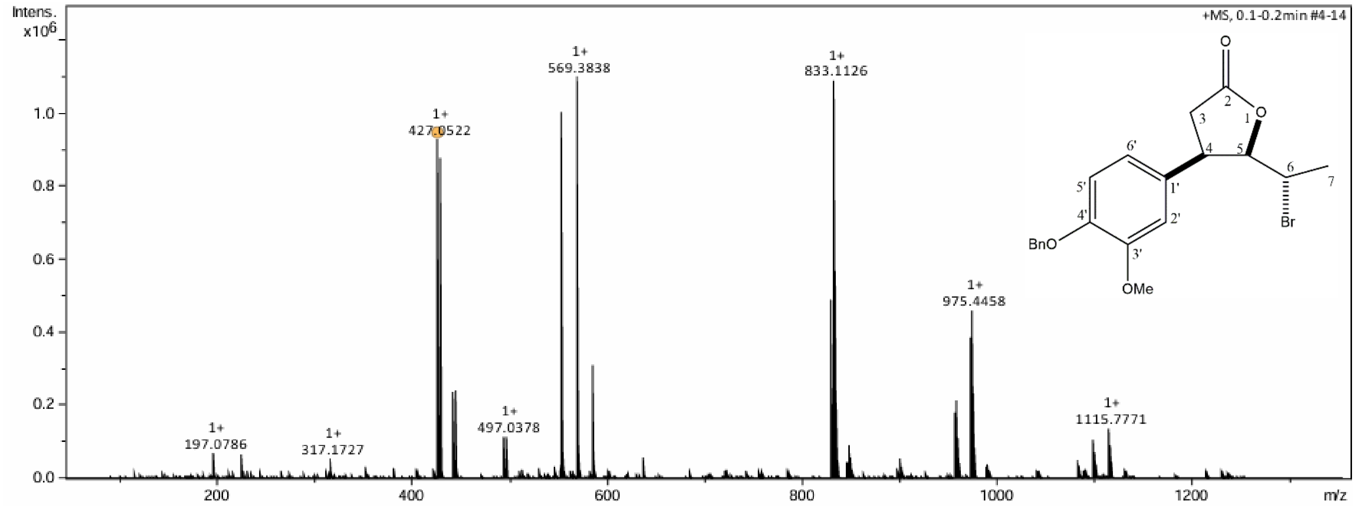


**Figure S128.** HRMS spectrum of *cis*-4-(4'-benzyloxy-3'-methoxyphenyl)-5-(1-bromoethyl)dihydrofuran-2-one **(8a)**

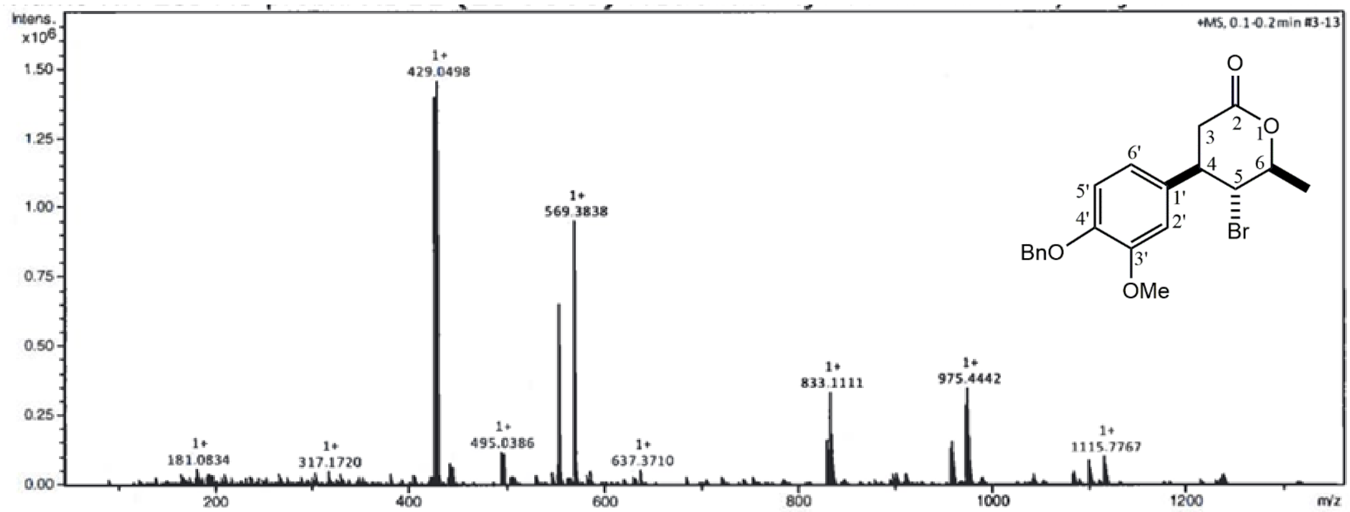

**Figure S129.** HRMS spectrum of 4-*r*-(4'-benzyloxy-3'-methoxyphenyl)-5-*t*-bromo-6-*c*-methyltetrahydropyran-2-one **(8b)**

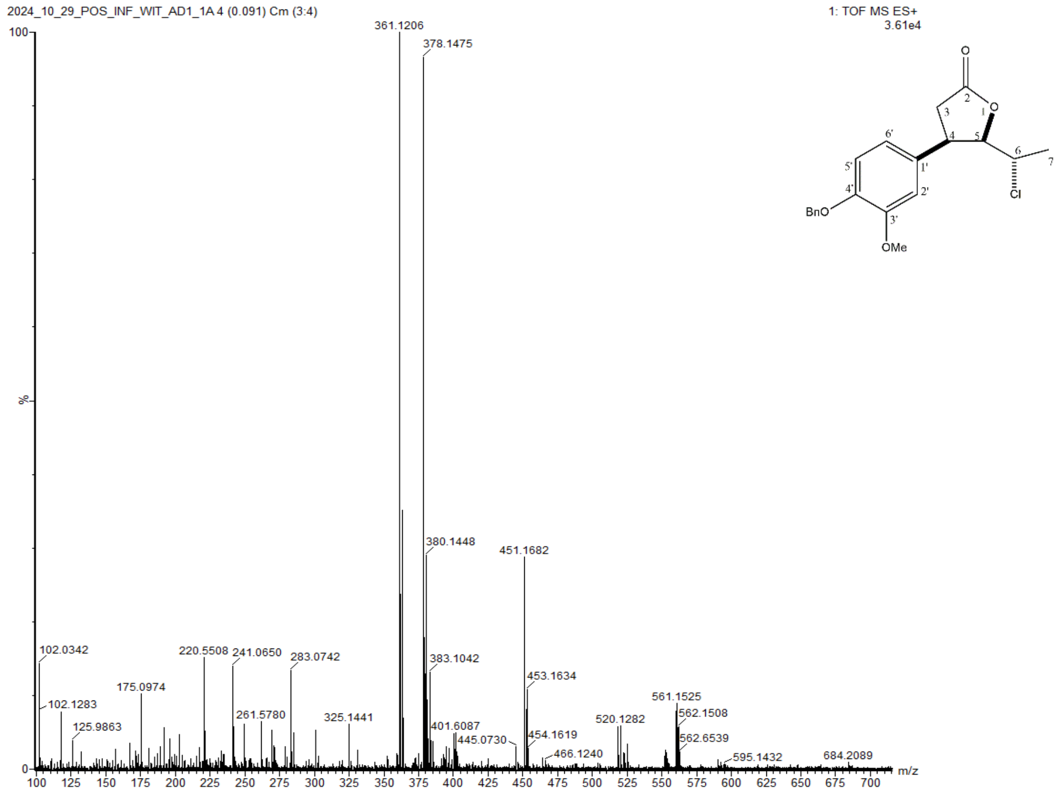


**Figure S130.** HRMS spectrum of *cis*-4-(4'-benzyloxy-3'-methoxyphenyl)-5-(1-chloroethyl)dihydrofuran-2-one **(9a)**

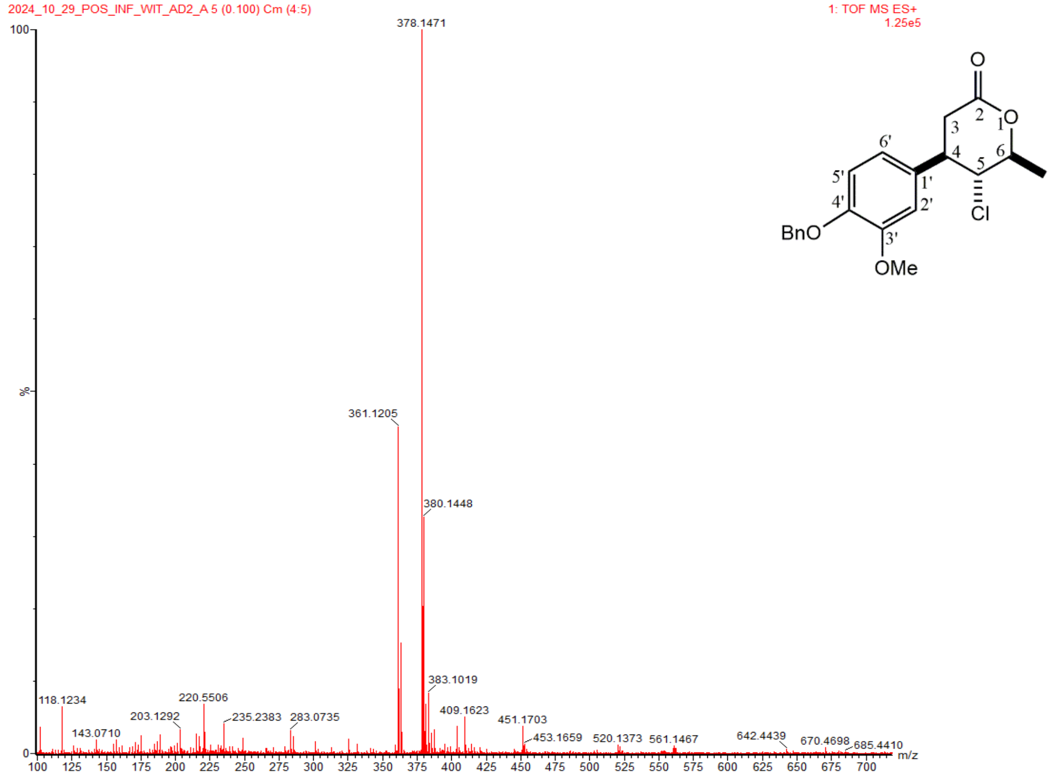


**Figure S131.** HRMS spectrum of 4-*r*-(4'-benzyloxy-3'-methoxyphenyl)-5-*t*-chloro-6-*c*-methyltetrahydropyran-2-one **(9b)**

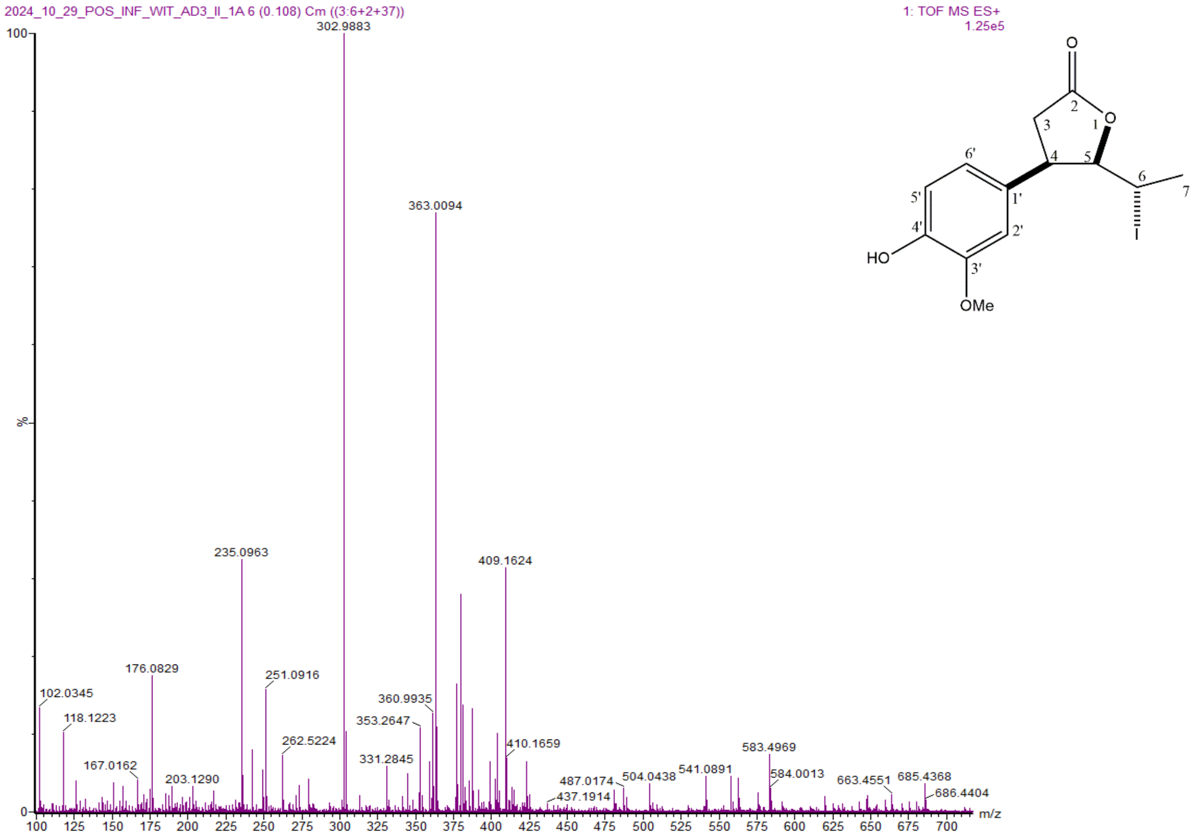


**Figure S132.** HRMS spectrum of *cis*-4-(4'-hydroxy-3'-methoxyphenyl)-5-(1-iodoethyl)dihydrofuran-2-one **(10a)**

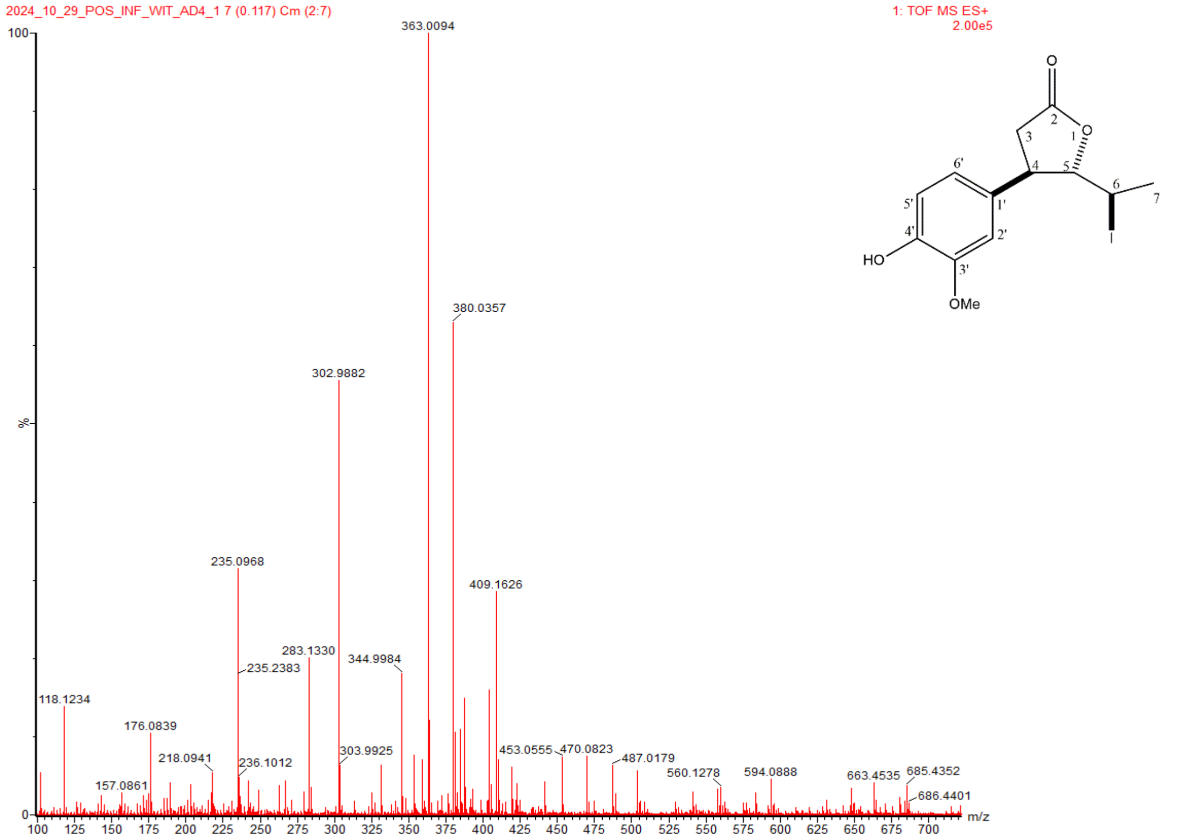

**Figure S133.** HRMS spectrum of *trans*-4-(4'-hydroxy-3'-methoxyphenyl)-5-(1-iodoethyl)dihydrofuran-2-one **(10b)**

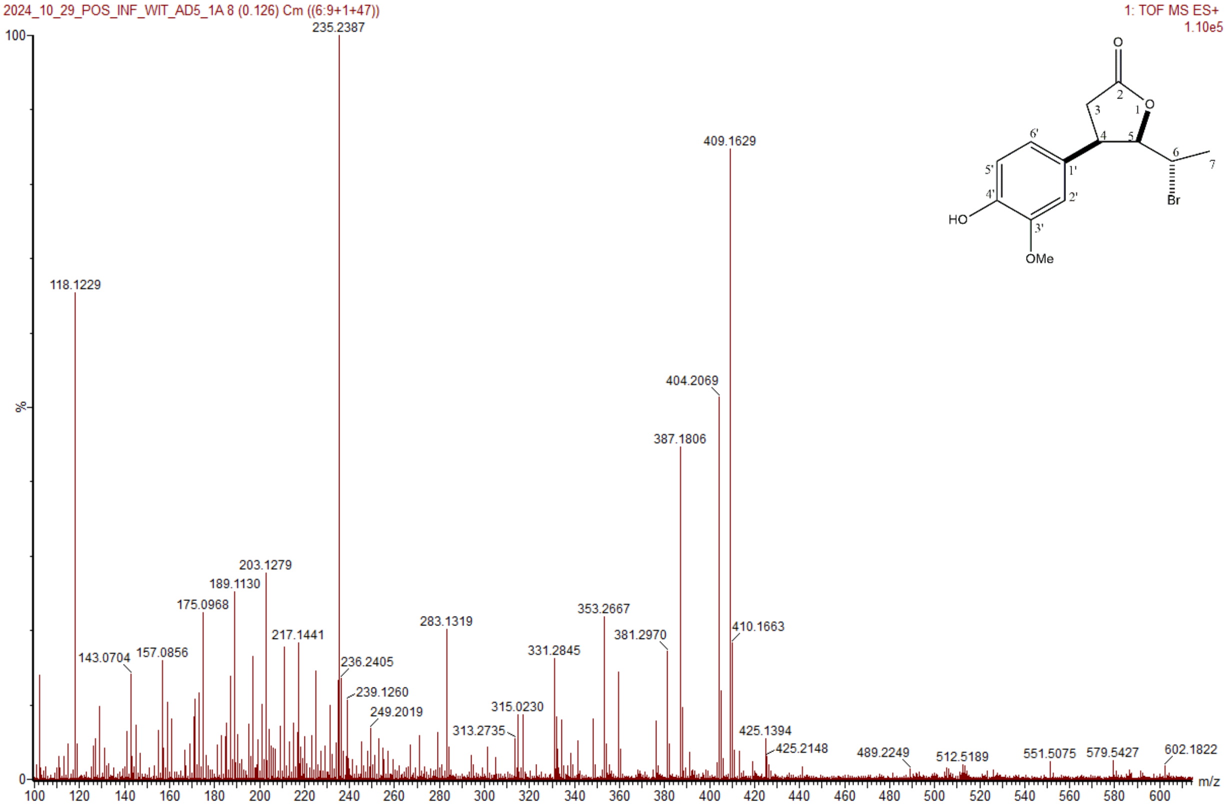


**Figure S134.** HRMS spectrum of *cis*-5-(1-bromoethyl)-4-(4'-hydroxy-3'-methoxyphenyl)dihydrofuran-2-one **(11a)**

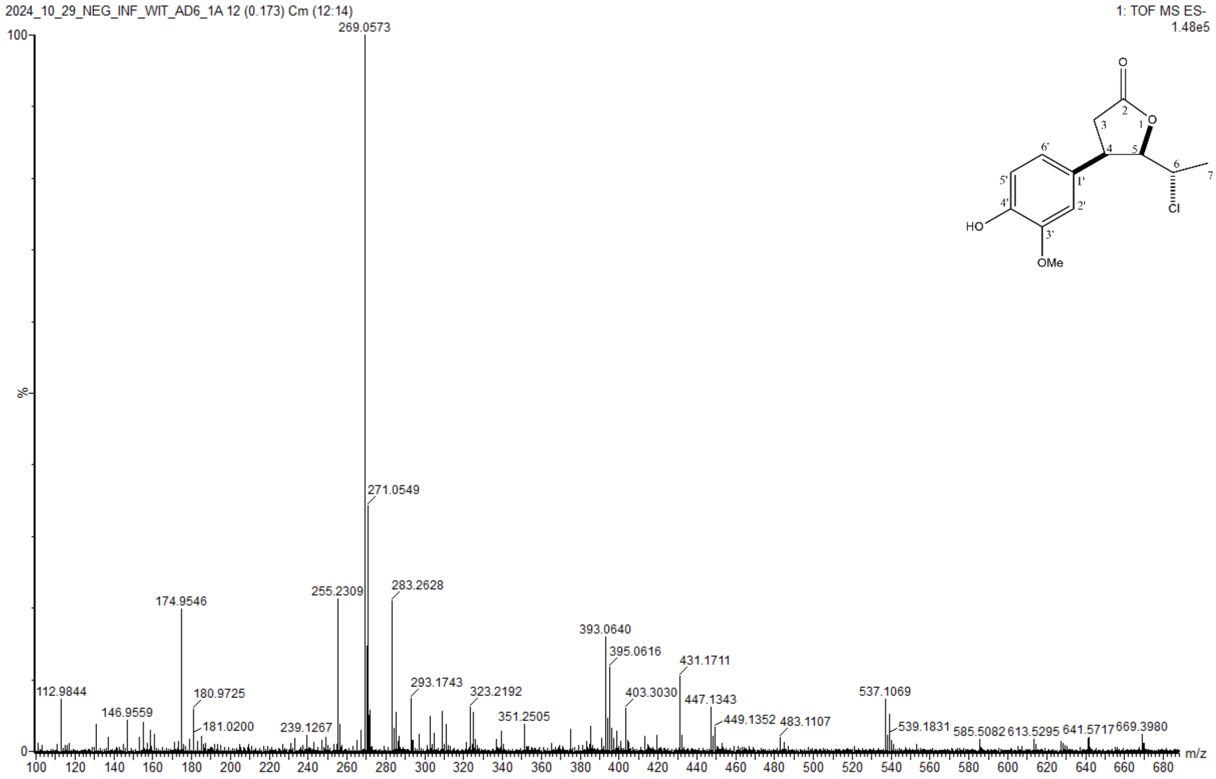


**Figure S135.** HRMS spectrum of *cis*-5-(1-chloroethyl)-4-(4'-hydroxy-3'-methoxyphenyl)dihydrofuran-2-one **(12a)**

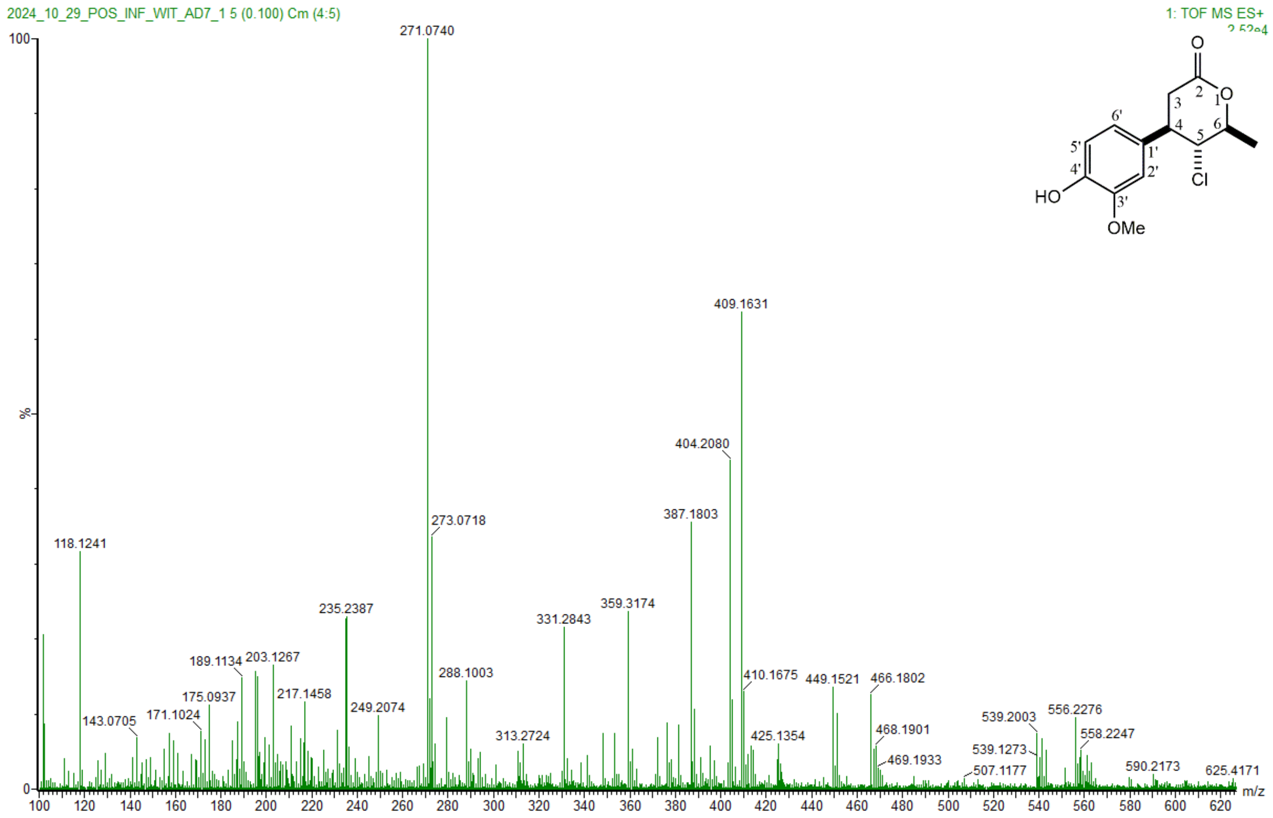


**Figure S136.** HRMS spectrum of 5-*t*-chloro-4-*r*-(4'-hydroxy-3'-methoxyphenyl)-6-*c*-methyltetrahydropyran-2-one **(12b)**

Spectral data of benzylvanillin (**2**)

$^{1}$H NMR (400 MHz, $CD_3OD$): δ 3.90 (s, 3H, $-OCH_3$), 5.21 (s, 2H, $-OCH_2Ph$), 7.17 (d, *J* = 8.2 Hz, 1H, H-5′), 7.32 (m, 1H, H-4′′), 7.35 – 7.40 (m, 2H, H-3′′, H-5′′), 7.43 – 7.47 (m, 3H, H-2′, H-2′′ and H-6′′), 7.49 (dd, *J* = 8.2 and 1.9 Hz, 1H, H-6′), 9.80 (s, 1H, -CHO); $^{13}$C NMR (100 MHz, $CD_3OD$): δ 56.44 ($-OCH_3$), 71.82 ($-OCH_2Ph$), 111.00 (C-2), 113.91 (C-5), 127.49 (C-6), 128.69 (C-2′,C-6′), 129.17 (C-4′), 129.59 (C-3′,C-5′), 131.71 (C-1), 137.84 (C-1′), 151.50 (C-3), 155.33 (C-4), 192.94 (-CHO); IR (ATR): $\nu_{max}$ = 2840, 1676, 1584, 1506, 1262, 1134, 748 $cm^{-1}$; HRMS (ESI): *m*/*z* calcd for $C_{15}H_{14}O_3$ $[M+Na]^+$: 265.0835; found: 265.0837.

Spectral data of (*E*)-4-(4'-(benzyloxy-3'-methoxyphenyl)but-3-en-2-one (**3**)

$^{1}$H NMR (400 MHz, $CDCl_3$): δ 2.36 (s, 3H, $CH_3$-1), 3.92 (s, 3H, $-OCH_3$), 5.19 (s, 2H, $-OCH_2Ph$), 6.59 (d, *J* = 16.2 Hz, 1H, H-3), 6.88 (d, *J* = 8.3 Hz, 1H, H-5′), 7.05 (dd, *J* = 8.3 Hz and 2.0 Hz, 1H, H-6′), 7.09 (d, *J* = 2.0 Hz, 1H, H-2′), 7.31 (m, 1H, H-4′′), 7.35-7.40 (m, 2H, H-3′′, H-5′′), 7.41-7.45 (m, 2H, H-2′′, H-6′′), 7.44 (d, *J* = 16.2 Hz, H-4); $^{13}$C NMR (100 MHz, $CDCl_3$): δ 27.31 (C-1), 55.95 ($-OCH_3$), 70.78 ($-OCH_2Ph$), 110.12 (C-2′), 113.34 (C-5′), 122.74 (C-6′), 125.31 (C-3), 127.16 (C-2′′,C-6′′), 127.61 (C-1′), 128.01 (C-4′′), 128.61 (C-3′′, C-5′′), 136.44 (C-1′′), 143.46 (C-4), 149.76 (C-3′), 150.43 (C-4′), 198.35 (C-2); IR (ATR): $\nu_{max}$ = 1661, 1637, 1514, 1255, 1142, 974, 839, 806, 744, 697 $cm^{-1}$; HRMS (ESI): *m*/*z* calcd for $C_{18}H_{18}O_3$ $[M+Na]^+$: 305.1148; found: 305.1155.
